# Supplementary material for: Genomic epidemiology of SARS-CoV-2 variants during the first two years of the pandemic in Colombia
Source: Commun Med (Lond). 2023 Jul 13;3:97. doi: 10.1038/s43856-023-00328-3 (PMC10344885; doi:10.1038/s43856-023-00328-3)
Supplement: Supplementary file 1 — Supplementary information [file 43856_2023_328_MOESM1_ESM.pdf]

1 Supplementary methods

In-house protocol for SARS-CoV-2 diagnostic by RT-qPCR

A validated multiplex RT-qPCR in-house protocol targeting E. RdRP, N, and RNAseP genes were used for the detection of SARS-CoV-2 viral RNA from nasopharyngeal swabs. This protocol uses 4 TaqMan probes labeled with HEX, CalFluorRed 610, FAM, and Quasar 670 for the detection of E, N, RdRP, and RNAseP genes. The latter is used for the quality control of sample collection and RNA extraction procedures due to its suitability as a housekeeping gene. RT-qPCR reactions were carried out as follows: working solutions consisting of 100 µM stocks of each primer were prepared in TE (pH 8.0) buffer. Then, a reaction mix was prepared to a final concentration of 200nM for each forward and reverse primer and 100nM for each probe. NEB Lunascript RT master mix was used with the beforementioned primers by adding 10µl of 2X reaction mix, 1µl of Enzyme mix and 6.5µl of nuclease-free water to a final reaction volume of 25µl.

Nucleic acid amplification was carried out in a BioRad CFX96 Real-time thermal cycler, samples with Ct values lower than 36 were considered positive and those with Ct values between 20 and 30 were selected for sequencing.

2 Supplementary Figures

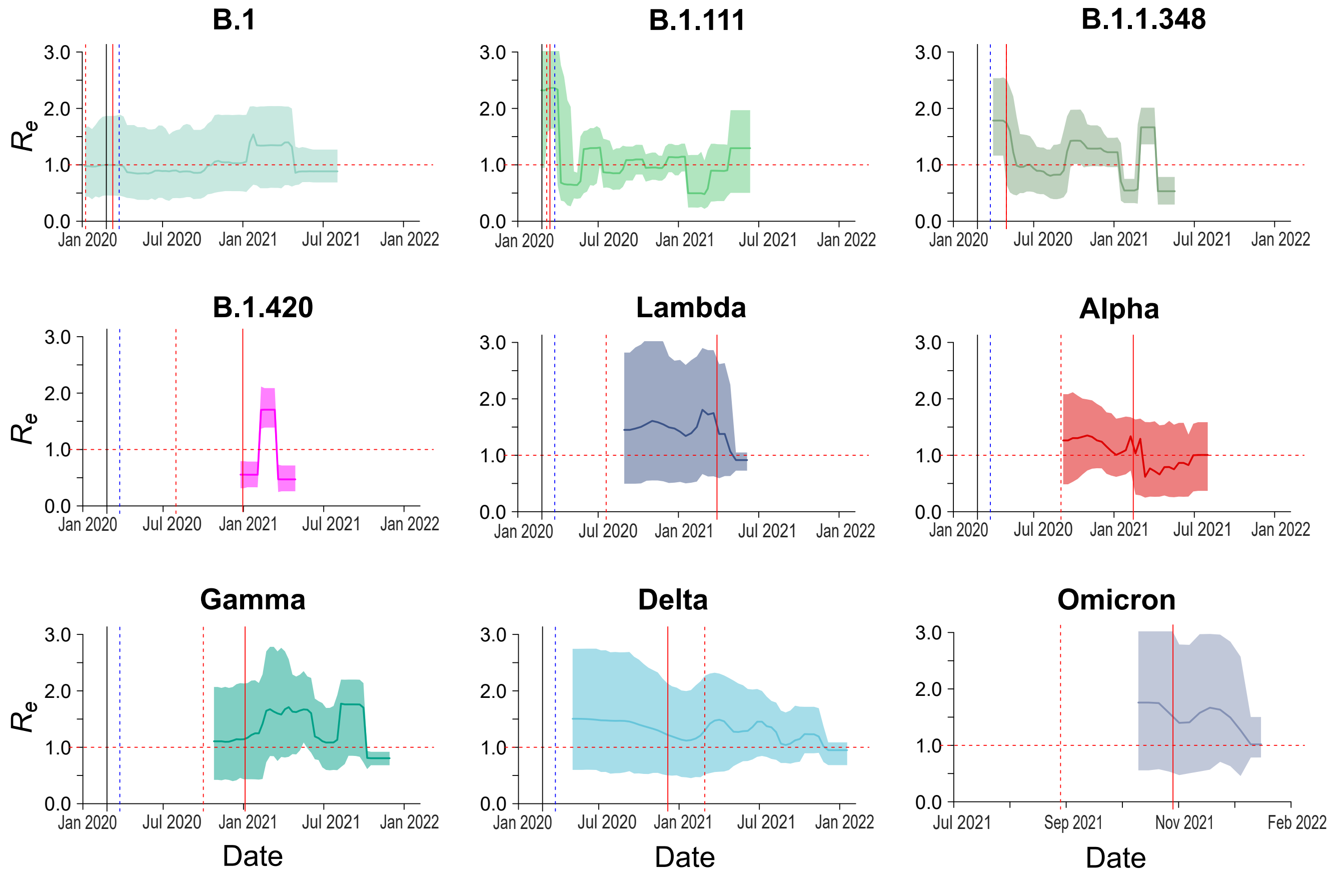

Supplementary Figure 1: Birth-death skyline (bdsky) analysis of nine COVID-19 variants circulated in Colombia. The most prevalent Pangolin COVID-19 global lineages during the first semester of the pandemic (B.1, B.1.111, B.1.1.348, B.1.420), one designated Variants of Interest (VOIs) (Lambda) and four SARS-CoV-2 variants of concern (VOCs: Alpha, Gamma, Delta, and Omicron). The median posterior estimate of the estimated effective reproductive number (R_e) over time is shown, with the 95% highest posterior density (HPD) interval in different colors. The horizontal red dotted line indicates the epidemic threshold (R_e =1). The black dotted line showed the first COVID-19 case that was identified in Colombia on February 26, 2020. The blue dotted line indicated the first National Lockdown in Colombia on March 25, 2020. The red dotted line showed the first sequence reported in https://cov-lineages.org/ for each variant. The continuous red line indicated the first Colombian sequence reported for each variant.

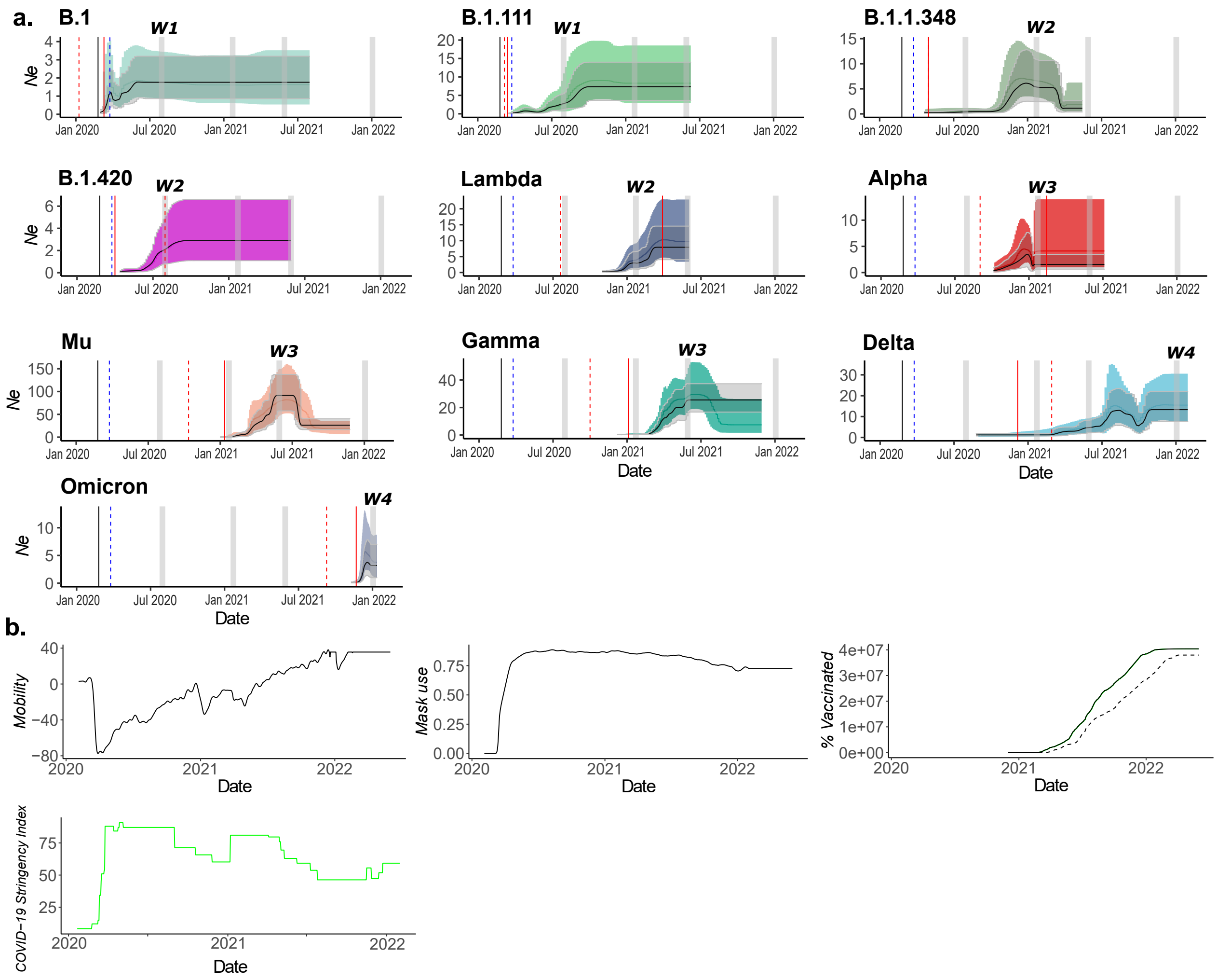

Supplementary Figure 2: Dynamic of SARS-CoV-2 in Colombia from March 2020 to February 2022. Effective population size (N_e) of a) The most prevalent Pangolin COVID-19 global lineages during the first semester of the pandemic (B.1, B.1.111, B.1.1.348, B.1.420), two designated Variants of Interest (VOIs) (Lambda and Mu) and four SARS-CoV-2 variants of concern (VOCs: Alpha, Gamma, Delta, and Omicron) which have circulated in Colombia using Bayesian skyline plot (in color) and BICEPS analysis (grey). Horizontal grey bars represent the four waves (W1, 2, 3, 4). N_e is shown on the y-axis, and the time in years is shown on the x-axis. The dark middle line indicates the median; lighter outer lines cover the 95%HPD intervals. The blue dotted line indicated the first National Lockdown in Colombia on March 25, 2020. The red dotted line showed the first sequence reported in https://cov-lineages.org/ for each variant. The continuous red line indicated the first Colombian sequence reported for each variant. b) Variables that could affect SARS-CoV-2 transmission (Mobility, Mask use, % vaccinated people, and COVID-19 Stringency index).

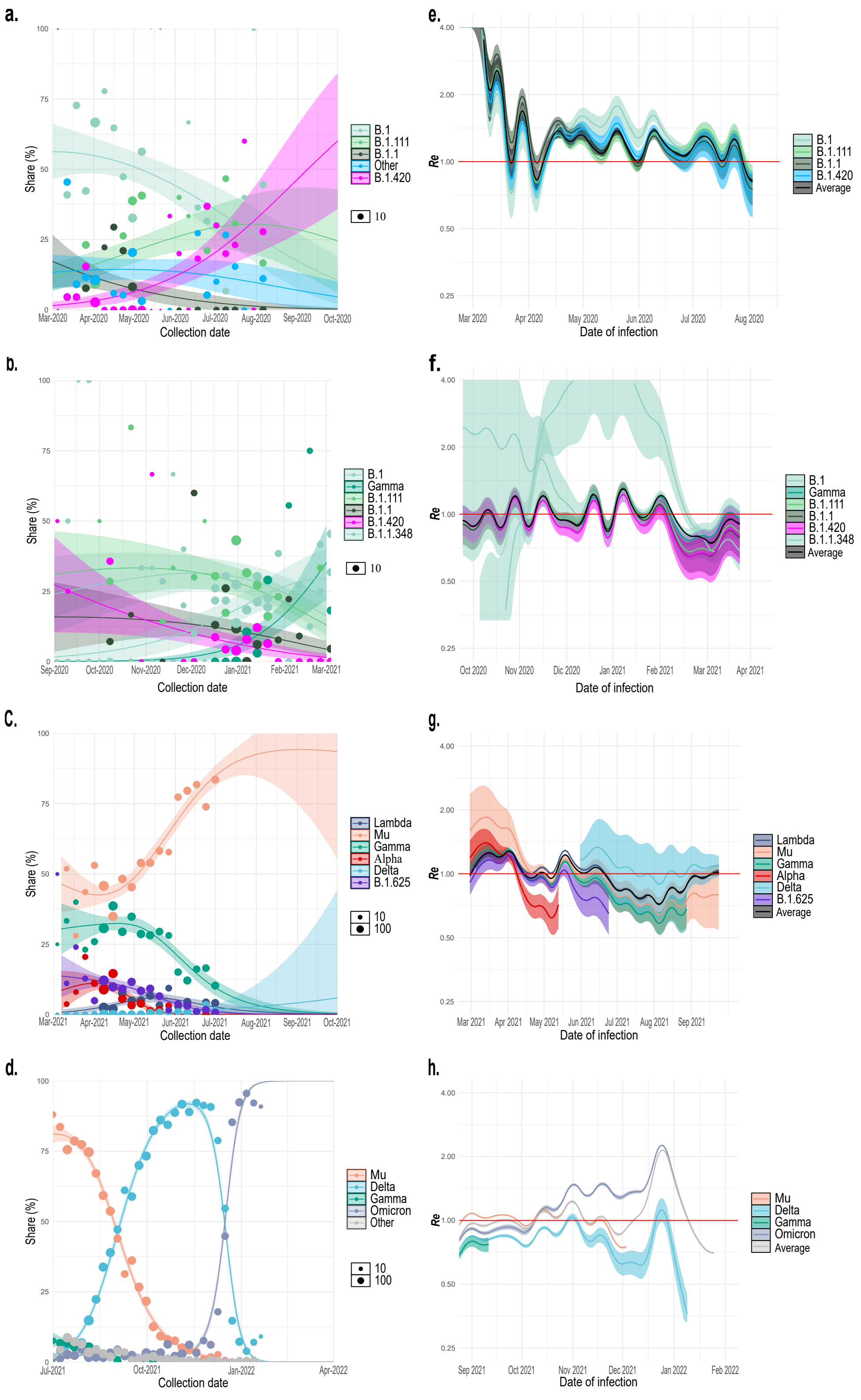

Supplementary Figure 3: Multinomial fit analysis for the four periods (waves) of the SARS-CoV-2 pandemic in Colombia. a) to d) show a reconstruction of the growth advantage dynamics of the circulating variants during the four pandemic periods inferred from the Global Initiative on Sharing All Influenza Data (GISAID) metadata and represented as share percentage. e) to h) show the R_e from each variant calculated from intrinsic growth rates as inferred from daily case data from World Health Organization (WHO).

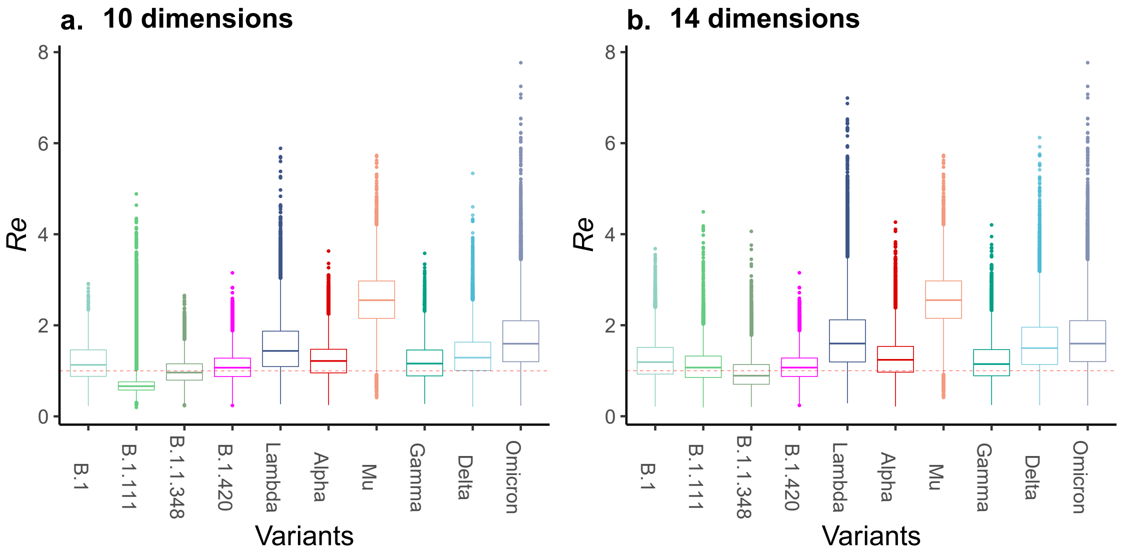

Supplementary Figure 4: Global R_e estimations of the most prevalent Pangolin COVID-19 global lineages, SARS-CoV-2 Variants of Interest and SARS-CoV-2 variant of concern (VOCIs) that have circulated in Colombia. The effective reproduction number (R_e) through time parameter was estimated using the Bayesian birth-death skyline model setting ten (a) and fourteen dimensions (b), showing congruence assuming different scenarios.

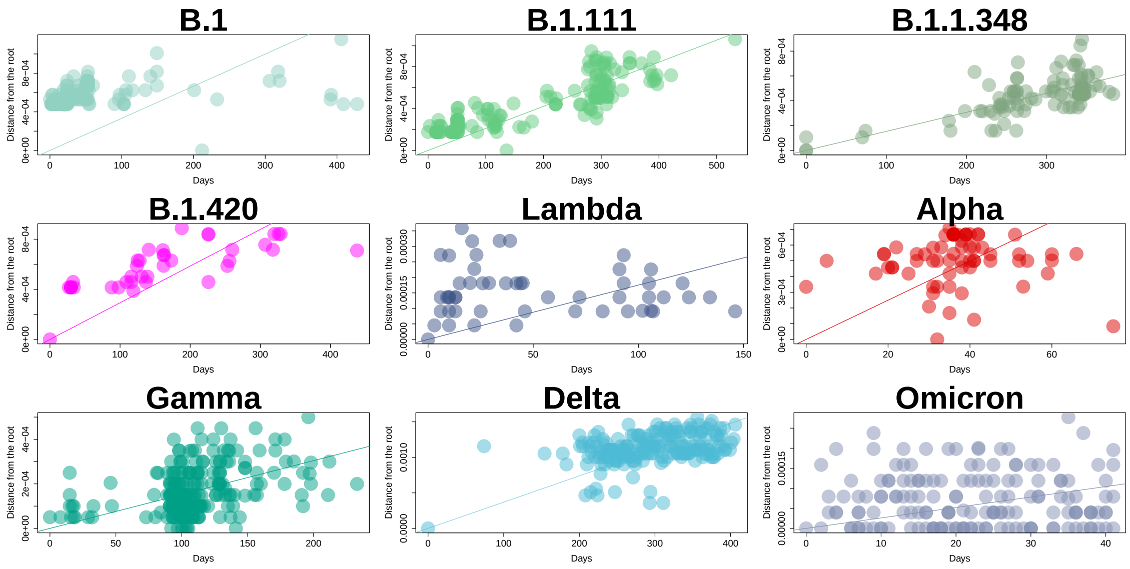

Supplementary Figure 5: Root-to-tip regression analysis of Colombian SARS-CoV-2 sequences down-sampling alignments showing the phylogenetic signal per variant.

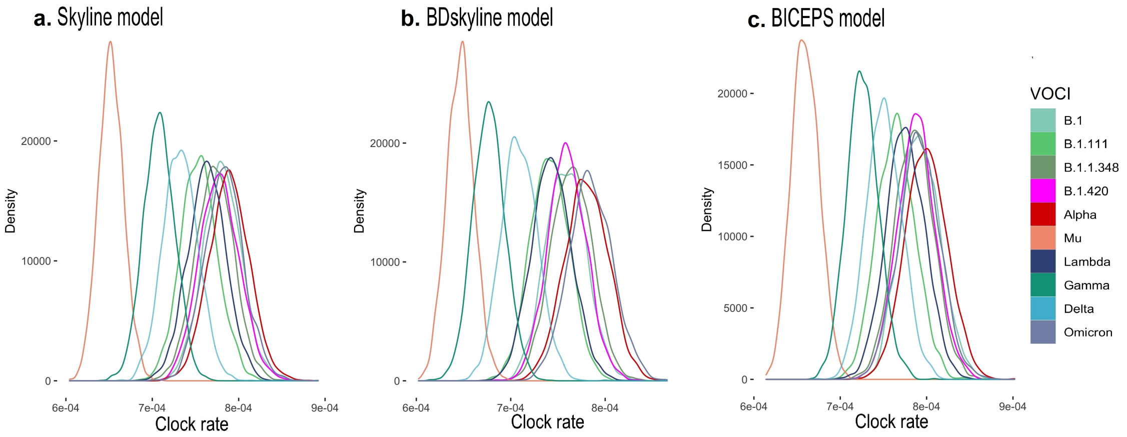

Supplementary Figure 6: Density plot of the posterior of the Clock rate parameter for ten variants' alignments (VOCIs). a) Coalescent Skyline model (Skyline), b) Birth-Death Skyline model (BDSkyline), c) Bayesian Integrated Coalescent Epoch PlotS (BICEPS).

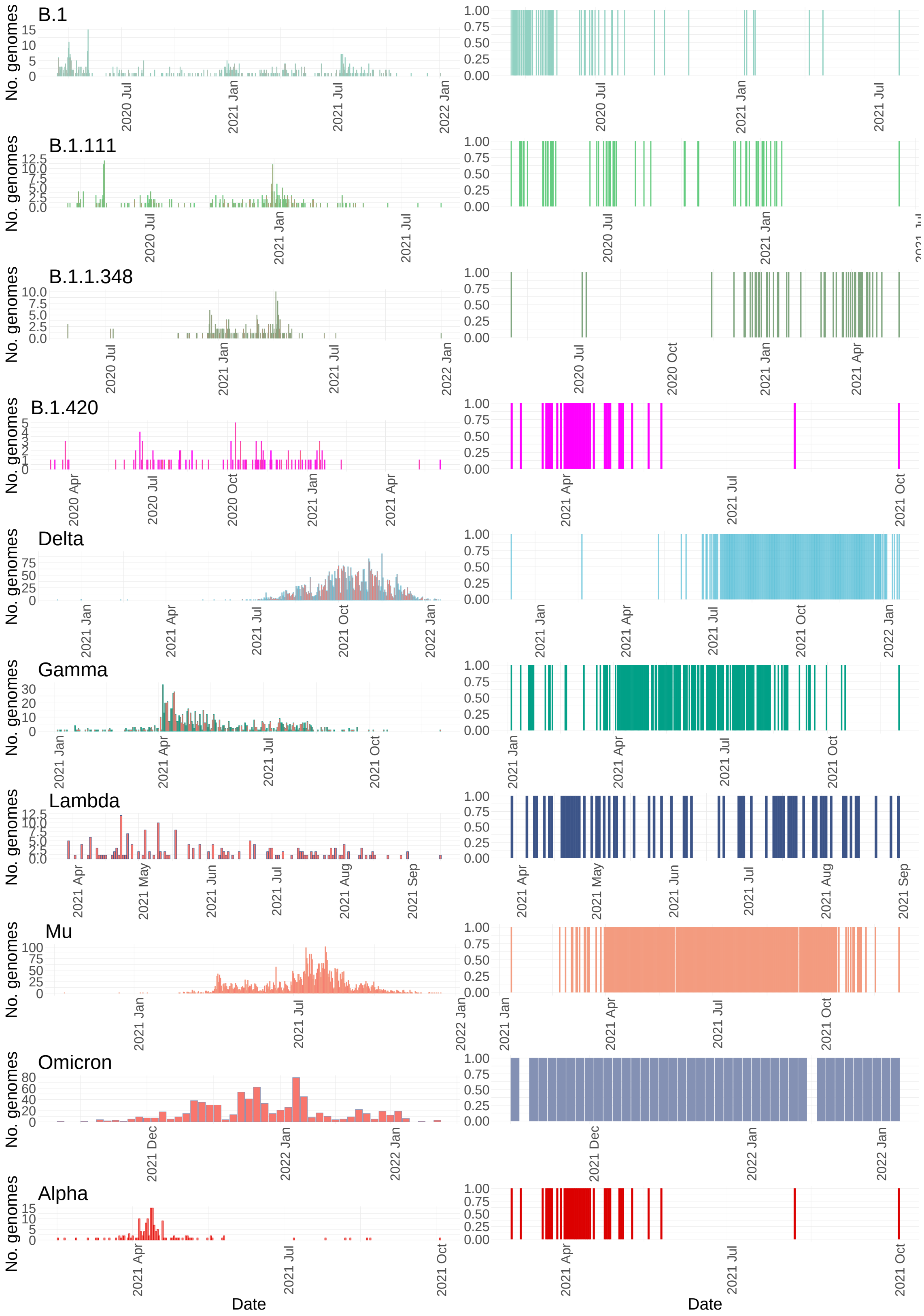

Supplementary Figure 7: Colombian SARS-CoV-2 sequences included in this study. The right panel shows the total of sequences available per variant collected for this study. The left panel shows the number of sequences used for the phylodynamic analysis after following the data curation pipeline.
