# Supplementary material for: Genomic epidemiology of SARS-CoV-2 variants during the first two years of the pandemic in Colombia
Source: Commun Med (Lond). 2023 Jul 13;3:97. doi: 10.1038/s43856-023-00328-3 (PMC10344885; doi:10.1038/s43856-023-00328-3)
Supplement: Supplementary file 2 — Description of Additional Supplementary Files [file 43856_2023_328_MOESM2_ESM.pdf]

Description of Additional Supplementary Files

File name: Supplementary Data 1
Description: Acknowledgment to the Authors and Laboratories. The following authors and laboratories are acknowledged for originating and submitting the SARS-CoV-2 genomes to GISAID, which were used as available sequences in this study.

File name: Supplementary Data 2
Description: Supplementary table that provides the access numbers of the novel SARS-CoV-2 sequences obtained from Colombia, which were included in this study. The current status is reported until June 2022. A total of 228 genome sequences were used in this study, with 124 genomes sequenced specifically for this research and 104 genomes downloaded from GISAID. The genomes with a sample collection date within the same time frame as the social protest are highlighted in gray.
