# Supplementary material for: Genomic epidemiology of SARS-CoV-2 variants during the first two years of the pandemic in Colombia
Source: Commun Med (Lond). 2023 Jul 13;3:97. doi: 10.1038/s43856-023-00328-3 (PMC10344885; doi:10.1038/s43856-023-00328-3)
Supplement: Supplementary file 3 — Supplementary Data 1 [file 43856_2023_328_MOESM3_ESM.pdf]

We gratefully acknowledge the following Authors from the Originating laboratories responsible for obtaining the specimens, as well as the Submitting laboratories where the genome data were generated and shared via GISAID, on which this research is based.

All Submitters of data may be contacted directly via www.gisaid.org

Authors are sorted alphabetically.

Accession ID	Originating Laboratory	Submitting Laboratory	Authors
EPI_ISL_3347173, EPI_ISL_3347174, EPI_ISL_3347175, EPI_ISL_3347176, EPI_ISL_3347177, EPI_ISL_3347178, EPI_ISL_3347179, EPI_ISL_3347180, EPI_ISL_3347181, EPI_ISL_3347182, EPI_ISL_3347183, EPI_ISL_3347184, EPI_ISL_3347185, EPI_ISL_3347186, EPI_ISL_3347187, EPI_ISL_3347188, EPI_ISL_3347189, EPI_ISL_3347190, EPI_ISL_3347191, EPI_ISL_3347192, EPI_ISL_3347193, EPI_ISL_3347194, EPI_ISL_3347195, EPI_ISL_3347196, EPI_ISL_3347197, EPI_ISL_3347198, EPI_ISL_3347199, EPI_ISL_3347200, EPI_ISL_3347201, EPI_ISL_3347202, EPI_ISL_3347203, EPI_ISL_3347204, EPI_ISL_3347205, EPI_ISL_3347206, EPI_ISL_3347207, EPI_ISL_3347208			
see above	ADILAB	Laboratorio Departamental de Salud Publica de Antioquia	Andres F. Cardona-Rios; Gloria Isabel Escobar; Idabely Betancur Ortiz; Juan P. Hernandez-Ortiz; Maria Stella López
EPI_ISL_1960045, EPI_ISL_1960056, EPI_ISL_2621212, EPI_ISL_2621213, EPI_ISL_2621214, EPI_ISL_2621215, EPI_ISL_2621216, EPI_ISL_2621217, EPI_ISL_2621218, EPI_ISL_2621219, EPI_ISL_2621220, EPI_ISL_2621221, EPI_ISL_2651233, EPI_ISL_2651234, EPI_ISL_3276535, EPI_ISL_3276536, EPI_ISL_3276537, EPI_ISL_3276538, EPI_ISL_3276539, EPI_ISL_3276540, EPI_ISL_3276541, EPI_ISL_3276542, EPI_ISL_3276543, EPI_ISL_3276544, EPI_ISL_3276545, EPI_ISL_3276549, EPI_ISL_3276550			
see above	ADILAB	Universidad Nacional de Colombia - Laboratorio Genómico One Health	Andres F. Cardona-Rios; Carlos Franco-Muñoz; Carolina Muñoz-Arango; Celeny Ortiz; Daniel O. Maldonado-Perez; Diego A. Álvarez-Díaz; Hector Alejandro Ruiz-Moreno; Idabely Betancur Ortiz; Jorge E. Osorio; Juan P. Hernandez-Ortiz; Karl A Ciuoderis; Katherine Laiton-Donato; Laura Silvana Perez; Lina M. Hurtado; Marcela Mercado-Reyes; Maria Angélica Maya; Maria Stella López; Rita Almanza Payares; Sandra Ines Cano; Simón Villegas Velásquez
EPI_ISL_1494955, EPI_ISL_1494956	AIDALAB S.A.S	Instituto Nacional de Salud- Dirección de Investigación en Salud Pública	Carlos Franco-Muñoz; Carmen Osorio; Diana Malo; Diego A. Álvarez-Díaz; Diego Andrés Prada; Gerardo Santamaría; Hector Alejandro Ruiz-Moreno; Jhonnatan Reales-González; Juan Camilo Martinez; Julian Naizaque; Katherine Laiton-Donato; Lisseth Pardo; Magdalena Wiesner; Marcela Mercado-Reyes; Maria T. Herrera-Sepúlveda; Marta Lopez Blanco; Martha Lucia Ospina Martinez; Paola Rojas; Sergio Gomez; Sheryll Corchuelo; Ángela Alarcon Cruz
EPI_ISL_1220051	ANALICEMOS LABORATORIO CLINICO ESPECIALIZADO	Instituto Nacional de Salud- Dirección de Investigación en Salud Pública	Carlos Franco-Muñoz; Diego A. Álvarez-Díaz; Diego Andrés Prada; Gerardo Santamaría; Hector Alejandro Ruiz-Moreno; Jhonnatan Reales-González; Julian Naizaque; Katherine Laiton-Donato; Magdalena Wiesner; Marcela Mercado-Reyes.; Maria T. Herrera-Sepúlveda; Martha Lucia Ospina Martínez; Sheryll Corchuelo
EPI_ISL_1220052	ANALICEMOS LABORATORIO ESPECIALIZADO	Instituto Nacional de Salud- Dirección de Investigación en Salud Pública	Carlos Franco-Muñoz; Diego A. Álvarez-Díaz; Diego Andrés Prada; Gerardo Santamaría; Hector Alejandro Ruiz-Moreno; Jhonnatan Reales-González; Julian Naizaque; Katherine Laiton-Donato; Magdalena Wiesner; Marcela Mercado-Reyes.; Maria T. Herrera-Sepúlveda; Martha Lucia Ospina Martínez; Sheryll Corchuelo
EPI_ISL_1632509, EPI_ISL_1820928, EPI_ISL_1820933, EPI_ISL_2674309	ANALIZAR LABORATORIO CLINICO AUTOMATIZADO - SYNLAB	Instituto Nacional de Salud- Dirección de Investigación en Salud Pública	Carlos Franco-Muñoz; Carmen Osorio; Christian Romero; Diana Malo; Diego A. Álvarez-Díaz; Diego Andrés Prada; Gerardo Santamaría; Hector Alejandro Ruiz-Moreno; Jhonnatan Reales-González; Jorge Rivera; Juan Camilo Martinez; Julian Naizaque; Katherine Laiton-Donato; Lisseth Pardo; Magdalena Wiesner; Marcela Mercado-Reyes; Maria T. Herrera-Sepúlveda; Marta Lopez Blanco; Martha Lucia Ospina Martinez; Paola Rojas; Patricia del Portillo; Sergio Gomez; Sheryll Corchuelo; Ángela Alarcon Cruz
EPI_ISL_4300692	AYUDAS DIAGNOSTICAS SURA	Instituto Nacional de Salud- Dirección de Investigación en Salud Pública	Carlos Franco-Muñoz; Carmen Osorio; Diana Malo; Diego A. Álvarez-Díaz; Diego Andrés Prada; Gerardo Santamaría; Hector Alejandro Ruiz-Moreno; Jhonnatan Reales-González; Jorge Rivera; Juan Camilo Martinez; Julian Naizaque; Katherine Laiton-Donato; Lisseth Pardo; Magdalena Wiesner; Marcela Mercado-Reyes; Maria T. Herrera-Sepúlveda; Marta Lopez Blanco; Martha Lucia Ospina Martinez; Paola Rojas; Sergio Gomez; Sheryll Corchuelo; Ángela Alarcon Cruz
EPI_ISL_1675315, EPI_ISL_1675316, EPI_ISL_1675317, EPI_ISL_1824704, EPI_ISL_2651227, EPI_ISL_2651228	AYUDAS DIAGNOSTICAS SURA	Universidad Nacional de Colombia - Laboratorio Genómico One Health	Andres F. Cardona-Rios; Carlos Franco-Muñoz; Carolina Muñoz-Arango; Celeny Ortiz; Daniel O. Maldonado-Perez; Diego A. Álvarez-Díaz; Hector Alejandro Ruiz-Moreno; Idabely Betancur Ortiz; Jorge E. Osorio; Juan P. Hernandez-Ortiz; Karl A Ciuoderis; Katherine Laiton-Donato; Laura Silvana Perez; Lina M. Hurtado; Marcela Mercado-Reyes; Maria Angélica Maya; Maria Stella López; Rita Almanza Payares; Sandra Ines Cano; Simón Villegas Velásquez
EPI_ISL_2438003, EPI_ISL_2438004, EPI_ISL_2438068, EPI_ISL_2438071, EPI_ISL_2438073, EPI_ISL_2438077, EPI_ISL_2438080, EPI_ISL_2438083			
see above	Adilab	Universidad Nacional de Colombia - Laboratorio Genómico One Health	Andres F. Cardona-Rios; Carlos Franco-Muñoz; Carolina Muñoz-Arango; Celeny Ortiz; Daniel O. Maldonado-Perez; Diego A. Álvarez-Díaz; Hector Alejandro Ruiz-Moreno; Idabely Betancur Ortiz; Jorge E. Osorio; Juan P. Hernandez-Ortiz; Karl A Ciuoderis; Katherine Laiton-Donato; Laura Silvana Perez; Lina M. Hurtado; Marcela Mercado-Reyes; Maria Angélica Maya; Maria Stella López; Rita Almanza Payares; Sandra Ines Cano; Simón Villegas Velásquez
EPI_ISL_3266994, EPI_ISL_3266995, EPI_ISL_3266998, EPI_ISL_3267001, EPI_ISL_3267002, EPI_ISL_3267003, EPI_ISL_3267004, EPI_ISL_3267005, EPI_ISL_3267006, EPI_ISL_3267007, EPI_ISL_3267008, EPI_ISL_3267009, EPI_ISL_3267010, EPI_ISL_3267011, EPI_ISL_3267012, EPI_ISL_3267013, EPI_ISL_3267014, EPI_ISL_3267015, EPI_ISL_3267016, EPI_ISL_3267017, EPI_ISL_3267018, EPI_ISL_3267019, EPI_ISL_3267020, EPI_ISL_3267021, EPI_ISL_3267022, EPI_ISL_3267023, EPI_ISL_3267024, EPI_ISL_3267025, EPI_ISL_3267026, EPI_ISL_3267027, EPI_ISL_3267028, EPI_ISL_3267029, EPI_ISL_3267030, EPI_ISL_3267031, EPI_ISL_3267032, EPI_ISL_3267033, EPI_ISL_3267034, EPI_ISL_3267035, EPI_ISL_3267036, EPI_ISL_3267037, EPI_ISL_3267038, EPI_ISL_3267039, EPI_ISL_3267040, EPI_ISL_3267041, EPI_ISL_3267042, EPI_ISL_3267044, EPI_ISL_3276561, EPI_ISL_3276562, EPI_ISL_3276563, EPI_ISL_3276564, EPI_ISL_3276565, EPI_ISL_3276566, EPI_ISL_3276567, EPI_ISL_3276568, EPI_ISL_3276569, EPI_ISL_3276570, EPI_ISL_3276571, EPI_ISL_3276572, EPI_ISL_3276573, EPI_ISL_3276574, EPI_ISL_3276575, EPI_ISL_3276576, EPI_ISL_3276577, EPI_ISL_3276578, EPI_ISL_3276579, EPI_ISL_3276580, EPI_ISL_3276581, EPI_ISL_3276582, EPI_ISL_3276583, EPI_ISL_3276584, EPI_ISL_3276585, EPI_ISL_3276586, EPI_ISL_3276587, EPI_ISL_3276588, EPI_ISL_3276589, EPI_ISL_3276590, EPI_ISL_3276591, EPI_ISL_3276592, EPI_ISL_3276593, EPI_ISL_3276594, EPI_ISL_3276595, EPI_ISL_3276596, EPI_ISL_3276597, EPI_ISL_3276598, EPI_ISL_3276599, EPI_ISL_3276600, EPI_ISL_3276601, EPI_ISL_3276602, EPI_ISL_3276603, EPI_ISL_3276604, EPI_ISL_3276605, EPI_ISL_3276606, EPI_ISL_3276607, EPI_ISL_3276608, EPI_ISL_3276609, EPI_ISL_3276610, EPI_ISL_3276611, EPI_ISL_3276612, EPI_ISL_3276613, EPI_ISL_3276614, EPI_ISL_3276615, EPI_ISL_3276616, EPI_ISL_3276617, EPI_ISL_3276618, EPI_ISL_3276619, EPI_ISL_3276620, EPI_ISL_3276621, EPI_ISL_3276622, EPI_ISL_3276623, EPI_ISL_3276624, EPI_ISL_3276625, EPI_ISL_3276626, EPI_ISL_3276627, EPI_ISL_3671001, EPI_ISL_3671004, EPI_ISL_3671005, EPI_ISL_3671007, EPI_ISL_3671017, EPI_ISL_3671021, EPI_ISL_3671023, EPI_ISL_3671026, EPI_ISL_3671027, EPI_ISL_3671029, EPI_ISL_3671030, EPI_ISL_3671031, EPI_ISL_3671032, EPI_ISL_3671033, EPI_ISL_3671034, EPI_ISL_3671035, EPI_ISL_3671037, EPI_ISL_3671043, EPI_ISL_3671050, EPI_ISL_3671052, EPI_ISL_3671054, EPI_ISL_3671056, EPI_ISL_3671060, EPI_ISL_3671062, EPI_ISL_3721582, EPI_ISL_3721583, EPI_ISL_3721597, EPI_ISL_3721603, EPI_ISL_3721604, EPI_ISL_3721612, EPI_ISL_3721613, EPI_ISL_3721621			
see above	Ayudas Diagnosticas Sura	Universidad Nacional de Colombia - Laboratorio Genómico One Health	Andres F. Cardona-Rios; Carlos Franco-Muñoz; Carolina Muñoz-Arango; Celeny Ortiz; Daniel O. Maldonado-Perez; Diego A. Álvarez-Díaz; Hector Alejandro Ruiz-Moreno; Idabely Betancur Ortiz; Jorge E. Osorio; Juan P. Hernandez-Ortiz; Karl A Ciuoderis; Katherine Laiton-Donato; Laura Silvana Perez; Lina M. Hurtado; Marcela Mercado-Reyes; Maria Angélica Maya; Maria Stella López; Rita Almanza Payares; Sandra Ines Cano; Simón Villegas Velásquez
EPI_ISL_2438005, EPI_ISL_2438006, EPI_ISL_2438007, EPI_ISL_2438008, EPI_ISL_2438009, EPI_ISL_2438010, EPI_ISL_2438011, EPI_ISL_2438012, EPI_ISL_2438013, EPI_ISL_2438014, EPI_ISL_2438062, EPI_ISL_2438065			
see above	Ayudas diagnosticas SURA	Universidad Nacional de Colombia - Laboratorio Genómico One Health	Andres F. Cardona-Rios; Carlos Franco-Muñoz; Carolina Muñoz-Arango; Celeny Ortiz; Daniel O. Maldonado-Perez; Diego A. Álvarez-Díaz; Hector Alejandro Ruiz-Moreno; Idabely Betancur Ortiz; Jorge E. Osorio; Juan P. Hernandez-Ortiz; Karl A Ciuoderis; Katherine Laiton-Donato; Laura Silvana Perez; Lina M. Hurtado; Marcela Mercado-Reyes; Maria Angélica Maya; Maria Stella López; Rita Almanza Payares; Sandra Ines Cano; Simón Villegas Velásquez
EPI_ISL_845618, EPI_ISL_845619	Ayudas diagnósticas SURA	Instituto Nacional de Salud - Dirección de Investigación en Salud Pública	Carlos Franco-Muñoz; Diego A. Álvarez-Díaz; Diego Andrés Prada; Gerardo Santamaría; Jonathan Reales; Julian Naizaque; Katherine Laiton-Donato; Magdalena Wiesner; Marcela Mercado-Reyes; Maria T. Herrera-Sepúlveda; Martha Lucia Ospina Martinez; Mauricio Pacheco-Montealegre; Paola Muñoz-Laiton; Sheryll Corchuelo
EPI_ISL_1017704	BIO-TEST	Instituto Nacional de Salud- Dirección de Investigación en Salud Pública	Carlos Franco-Muñoz; Diego A. Álvarez-Díaz; Diego Andrés Prada; Gerardo Santamaría; Hector Alejandro Ruiz-Moreno; Jhonnatan Reales-González; Julian Naizaque; Katherine Laiton-Donato; Magdalena Wiesner; Marcela Mercado-Reyes; Maria T. Herrera-Sepúlveda; Martha Lucia Ospina Martinez; Sheryll Corchuelo
EPI_ISL_2321578, EPI_ISL_2321579, EPI_ISL_2321580, EPI_ISL_2321581, EPI_ISL_2321582, EPI_ISL_2321583, EPI_ISL_2321584, EPI_ISL_2321585, EPI_ISL_2321586			
see above	BIOREFERENCIA	Universidad Nacional de Colombia - Laboratorio Genómico One Health	Andres F. Cardona-Rios; Carlos Franco-Muñoz; Carolina Muñoz-Arango; Celeny Ortiz; Daniel O. Maldonado-Perez; Diego A. Álvarez-Díaz; Hector Alejandro Ruiz-Moreno; Idabely Betancur Ortiz; Jorge E. Osorio; Juan P. Hernandez-Ortiz; Karl A Ciuoderis; Katherine Laiton-Donato; Laura Silvana Perez; Lina M. Hurtado; Marcela Mercado-Reyes; Maria Angélica Maya; Maria Stella López; Rita Almanza Payares; Sandra Ines Cano; Simón Villegas Velásquez
EPI_ISL_2657866, EPI_ISL_2828039, EPI_ISL_2828040	CARVAJAL LABORATORIO IPS	Instituto Nacional de Salud- Dirección de Investigación en Salud Pública	Carlos Franco-Muñoz; Carmen Osorio; Diana Malo; Diego A. Álvarez-Díaz; Diego Andrés Prada; Gerardo Santamaría; Hector Alejandro Ruiz-Moreno; Jhonnatan Reales-González; Jorge Rivera; Juan Camilo Martinez; Julian Naizaque; Katherine Laiton-Donato; Lisseth Pardo; Magdalena Wiesner; Marcela Mercado-Reyes; Maria T. Herrera-Sepúlveda; Marta Lopez Blanco; Martha Lucia Ospina Martinez; Paola Rojas; Sergio Gomez; Sheryll Corchuelo; Ángela Alarcon Cruz
EPI_ISL_2674320, EPI_ISL_2674321, EPI_ISL_2674322, EPI_ISL_3385768, EPI_ISL_3385770	CARVAJAL LABORATORIOS	Instituto Nacional de Salud- Dirección de Investigación en Salud Pública	Carlos Franco-Muñoz; Carmen Osorio; Diana Malo; Diego A. Álvarez-Díaz; Diego Andrés Prada; Gerardo Santamaría; Hector Alejandro Ruiz-Moreno; Jhonnatan Reales-González; Jorge Rivera; Juan Camilo Martinez; Julian Naizaque; Katherine Laiton-Donato; Lisseth Pardo; Magdalena Wiesner; Marcela Mercado-Reyes; Maria T. Herrera-Sepúlveda; Marta Lopez Blanco; Martha Lucia Ospina Martinez; Paola Rojas; Sergio Gomez; Sheryll Corchuelo; Ángela Alarcon Cruz
EPI_ISL_2657865	CARVAJALA LABORATORIO IPS	Instituto Nacional de Salud- Dirección de Investigación en Salud Pública	Carlos Franco-Muñoz; Carmen Osorio; Diana Malo; Diego A. Álvarez-Díaz; Diego Andrés Prada; Gerardo Santamaría; Hector Alejandro Ruiz-Moreno; Jhonnatan Reales-González; Jorge Rivera; Juan Camilo Martinez; Julian Naizaque; Katherine Laiton-Donato; Lisseth Pardo; Magdalena Wiesner; Marcela Mercado-Reyes; Maria T. Herrera-Sepúlveda; Marta Lopez Blanco; Martha Lucia Ospina Martinez; Paola Rojas; Sergio Gomez; Sheryll Corchuelo; Ángela Alarcon Cruz
EPI_ISL_2362583, EPI_ISL_2362584	CDI	Instituto Nacional de Salud- Dirección de Investigación en Salud Pública	Carlos Franco-Muñoz; Carmen Osorio; Diana Malo; Diego A. Álvarez-Díaz; Diego Andrés Prada; Gerardo Santamaría; Hector Alejandro Ruiz-Moreno; Jhonnatan Reales-González; Jorge Rivera; Juan Camilo Martinez; Julian Naizaque; Katherine Laiton-Donato; Lisseth Pardo; Magdalena Wiesner; Marcela Mercado-Reyes; Maria T. Herrera-Sepúlveda; Marta Lopez Blanco; Martha Lucia Ospina Martinez; Paola Rojas; Sergio Gomez; Sheryll Corchuelo; Ángela Alarcon Cruz
EPI_ISL_3372558, EPI_ISL_3385816, EPI_ISL_3385817, EPI_ISL_3385844, EPI_ISL_3385850	CENTRO DE GENETICA Y BIOLOGIA MOLECULAR UNIVERSIDAD DEL MAGDALENA	Instituto Nacional de Salud- Dirección de Investigación en Salud Pública	Carlos Franco-Muñoz; Carmen Osorio; Diana Malo; Diego A. Álvarez-Díaz; Diego Andrés Prada; Gerardo Santamaría; Hector Alejandro Ruiz-Moreno; Jhonnatan Reales-González; Jorge Rivera; Juan Camilo Martinez; Julian Naizaque; Katherine Laiton-Donato; Lisseth Pardo; Magdalena Wiesner; Marcela Mercado-Reyes; Maria T. Herrera-Sepúlveda; Marta Lopez Blanco; Martha Lucia Ospina Martinez; Paola Rojas; Sergio Gomez; Sheryll Corchuelo; Ángela Alarcon Cruz
EPI_ISL_1235688	CENTRO MEDICO COLSANITAS LA CALLEJA	Laboratorio de salud pública (Bogotá) and Gencore (Universidad de los Andes)	Alejandro Gómez; David González; Johana Hernandez Gabriela Delgado; Johanna Vargas; Luisa Sacristán; Marcela Guevara-Suarez; Silvia Restrepo
EPI_ISL_2621205, EPI_ISL_2621206, EPI_ISL_2621207, EPI_ISL_2621208	CENTROLAB	Universidad Nacional de Colombia - Laboratorio Genómico One Health	Andres F. Cardona-Rios; Carlos Franco-Muñoz; Carolina Muñoz-Arango; Celeny Ortiz; Daniel O. Maldonado-Perez; Diego A. Álvarez-Díaz; Hector Alejandro Ruiz-Moreno; Idabely Betancur Ortiz; Jorge E. Osorio; Juan P. Hernandez-Ortiz; Karl A Ciuoderis; Katherine Laiton-Donato; Laura Silvana Perez; Lina M. Hurtado; Marcela Mercado-Reyes; Maria Angélica Maya; Maria Stella López; Rita Almanza Payares; Sandra Ines Cano; Simón Villegas Velásquez
EPI_ISL_1494954	CHRISTUS SINERGIA - Clínica Farallones	Instituto Nacional de Salud- Dirección de Investigación en Salud Pública	Carlos Franco-Muñoz; Carmen Osorio; Diana Malo; Diego A. Álvarez-Díaz; Diego Andrés Prada; Gerardo Santamaría; Hector Alejandro Ruiz-Moreno; Jhonnatan Reales-González; Juan Camilo Martinez; Julian Naizaque; Katherine Laiton-Donato; Lisseth Pardo; Magdalena Wiesner; Marcela Mercado-Reyes; Maria T. Herrera-Sepúlveda; Marta Lopez Blanco; Martha Lucia Ospina Martinez; Paola Rojas; Sergio Gomez; Sheryll Corchuelo; Ángela Alarcon Cruz
EPI_ISL_2861244, EPI_ISL_2861245	CLI NICA MISERICORDIA INTERNACIONAL	Instituto Nacional de Salud- Dirección de Investigación en Salud Pública	Carlos Franco-Muñoz; Carmen Osorio; Diana Malo; Diego A. Álvarez-Díaz; Diego Andrés Prada; Gerardo Santamaría; Hector Alejandro Ruiz-Moreno; Jhonnatan Reales-González; Jorge Rivera; Juan Camilo Martinez; Julian Naizaque; Katherine Laiton-Donato; Lisseth Pardo; Magdalena Wiesner; Marcela Mercado-Reyes; Maria T. Herrera-Sepúlveda; Marta Lopez Blanco; Martha Lucia Ospina Martinez; Paola Rojas; Sergio Gomez; Sheryll Corchuelo; Ángela Alarcon Cruz
EPI_ISL_3462747	CLINICA ALTA COMPLEJIDAD DEL CARIBE	Instituto Nacional de Salud	Carlos Franco-Muñoz; Carmen Osorio; Diana Malo; Diego A. Álvarez-Díaz; Diego Andrés Prada; Gerardo Santamaría; Hector Alejandro Ruiz-Moreno; Jhonnatan Reales-González; Jorge Rivera; Juan Camilo Martinez; Julian Naizaque; Katherine Laiton-Donato; Lisseth Pardo; Magdalena Wiesner; Marcela Mercado-Reyes; Maria T. Herrera-Sepúlveda; Marta Lopez Blanco; Martha Lucia Ospina Martinez; Paola Rojas; Sergio Gomez; Sheryll Corchuelo; Ángela Alarcon Cruz
EPI_ISL_4028593, EPI_ISL_4028595, EPI_ISL_4028596, EPI_ISL_4028600, EPI_ISL_4028604, EPI_ISL_4028606, EPI_ISL_4028611, EPI_ISL_4028613			
see above	CLINICA BONNADONA PREVENIR	Molecular Genetics and Antimicrobial Resistance - UGRA, Universidad El Bosque	Betsy Castro; Catalina Espitia; Jinnethe Reyes; Lorena Diaz; Marcela Mercado; Mauricio Pacheco; Rafael Rios; Sandra Rincon; Valentina Martinez
EPI_ISL_1303374, EPI_ISL_1632513	CLINICA COLSANITAS CENTRAL DE REFERENCIA	Instituto Nacional de Salud- Dirección de Investigación en Salud Pública	Carlos Franco-Muñoz; Carmen Osorio; Diana Malo; Diego A. Álvarez-Díaz; Diego Andrés Prada; Gerardo Santamaría; Hector Alejandro Ruiz-Moreno; Jhonnatan Reales-González; Jorge Rivera; Juan Camilo Martinez; Julian Naizaque; Katherine Laiton-Donato; Lisseth Pardo; Magdalena Wiesner; Marcela Mercado-Reyes; Maria T. Herrera-Sepúlveda; Marta Lopez Blanco; Martha Lucia Ospina Martinez; Paola Rojas; Sergio Gomez; Sheryll Corchuelo; Ángela Alarcon Cruz
EPI_ISL_2362563	CLINICA DE LA COSTA	Instituto Nacional de Salud- Dirección de Investigación en Salud Pública	Carlos Franco-Muñoz; Carmen Osorio; Diana Malo; Diego A. Álvarez-Díaz; Diego Andrés Prada; Gerardo Santamaría; Hector Alejandro Ruiz-Moreno; Jhonnatan Reales-González; Jorge Rivera; Juan Camilo

			Martinez; Julian Naizaque; Katherine Laiton-Donato; Lisseth Pardo; Magdalena Wiesner; Marcela Mercado-Reyes; Maria T. Herrera-Sepúlveda; Marta Lopez Blanco; Martha Lucia Ospina Martinez; Paola Rojas; Sergio Gomez; Sheryll Corchuelo; Ángela Alarcon Cruz
EPI_ISL_906540, EPI_ISL_906544	CLINICA DE OCCIDENTE	Instituto Nacional de Salud- Dirección de Investigación en Salud Pública, Universidad de los Andes- Applied genomics research group, Vicerrectoria de Investigación y Creación, Universidad de los Andes- Systems and Computing Engineering Department	Carlos Franco-Muñoz; Diego A. Álvarez-Díaz; Diego Andrés Prada; Gerardo Santamaría Jorge Duitama; Héctor Alejandro Ruiz-Moreno; Jhonnatan Reales-González; Jorge Ivan Diaz; Julian Naizaque; Katherine Laiton-Donato; Laura Natalia Gonzalez; Magdalena Wiesner; Marcela Mercado-Reyes; Maria T. Herrera-Sepúlveda; Martha Lucia Ospina Martinez; Mauricio Pacheco-Montealegre; Sheryll Corchuelo; Silvia Restrepo-Restrepo
EPI_ISL_1632503, EPI_ISL_1820958	CLINICA FARALLONES	Instituto Nacional de Salud- Dirección de Investigación en Salud Pública	Carlos Franco-Muñoz; Carmen Osorio; Christian Romero; Diana Malo; Diego A. Álvarez-Díaz; Diego Andrés Prada; Gerardo Santamaría; Hector Alejandro Ruiz-Moreno; Jhonnatan Reales-González; Jorge Rivera; Juan Camilo Martinez; Julian Naizaque; Katherine Laiton-Donato; Lisseth Pardo; Magdalena Wiesner; Marcela Mercado-Reyes; Maria T. Herrera-Sepúlveda; Marta Lopez Blanco; Martha Lucia Ospina Martinez; Paola Rojas; Patricia del Portillo; Sergio Gomez; Sheryll Corchuelo; Ángela Alarcon Cruz
EPI_ISL_3459396, EPI_ISL_3459397	CLINICA IBEROAMERICANA	Instituto Nacional de Salud	Carlos Franco-Muñoz; Carmen Osorio; Diana Malo; Diego A. Álvarez-Díaz; Diego Andrés Prada; Gerardo Santamaría; Hector Alejandro Ruiz-Moreno; Jhonnatan Reales-González; Jorge Rivera; Juan Camilo Martinez; Julian Naizaque; Katherine Laiton-Donato; Lisseth Pardo; Magdalena Wiesner; Marcela Mercado-Reyes; Maria T. Herrera-Sepúlveda; Marta Lopez Blanco; Martha Lucia Ospina Martinez; Paola Rojas; Sergio Gomez; Sheryll Corchuelo; Ángela Alarcon Cruz
EPI_ISL_906536	CLINICA OCCIDENTE	Instituto Nacional de Salud- Dirección de Investigación en Salud Pública, Universidad de los Andes- Applied genomics research group, Vicerrectoria de Investigación y Creación, Universidad de los Andes- Systems and Computing Engineering Department	Carlos Franco-Muñoz; Diego A. Álvarez-Díaz; Diego Andrés Prada; Gerardo Santamaría Jorge Duitama; Héctor Alejandro Ruiz-Moreno; Jhonnatan Reales-González; Jorge Ivan Diaz; Julian Naizaque; Katherine Laiton-Donato; Laura Natalia Gonzalez; Magdalena Wiesner; Marcela Mercado-Reyes; Maria T. Herrera-Sepúlveda; Martha Lucia Ospina Martinez; Mauricio Pacheco-Montealegre; Sheryll Corchuelo; Silvia Restrepo-Restrepo
EPI_ISL_3459376, EPI_ISL_3459406, EPI_ISL_3459408	CLINICA PRIMAVERA DE VILLAVICENCIO	Instituto Nacional de Salud	Carlos Franco-Muñoz; Carmen Osorio; Diana Malo; Diego A. Álvarez-Díaz; Diego Andrés Prada; Gerardo Santamaría; Hector Alejandro Ruiz-Moreno; Jhonnatan Reales-González; Jorge Rivera; Juan Camilo Martinez; Julian Naizaque; Katherine Laiton-Donato; Lisseth Pardo; Magdalena Wiesner; Marcela Mercado-Reyes; Maria T. Herrera-Sepúlveda; Marta Lopez Blanco; Martha Lucia Ospina Martinez; Paola Rojas; Sergio Gomez; Sheryll Corchuelo; Ángela Alarcon Cruz
EPI_ISL_3355514, EPI_ISL_3355515, EPI_ISL_3355516	CLINICA PUTUMAYO	Corporacion CorpoGen-Universidad de los Andes-Universidad Central	Christian Romero; Jorge Duitama; Juan Manuel Anzola; Laura González; Maryam Chaib De Mares; María Mercedes Zambrano; Nelly Díaz; Patricia Del Portillo; Silvia Restrepo
EPI_ISL_1629738, EPI_ISL_1629739, EPI_ISL_1629740, EPI_ISL_1629741, EPI_ISL_1629742, EPI_ISL_1675308, EPI_ISL_1675309			
see above	CLÍNICA U BOLIVARIANA	Universidad Nacional de Colombia - Laboratorio Genómico One Health	Andres F. Cardona-Rios; Carlos Franco-Muñoz; Daniel O. Maldonado-Perez; Diego A. Álvarez-Díaz; Eliana Patricia Calvo Tapiero; Hector Alejandro Ruiz-Moreno; Idabely Betancur Ortiz; Jorge E. Osorio; Juan P. Hernandez-Ortiz; Karl A Ciuoderis; Katherine Laiton-Donato; Laura Silvana Perez; Lina M. Hurtado; Marcela Mercado-Reyes; Maria Angélica Maya; Maria Stella López; Rita Almanza Payares; Sandra Ines Cano; Simón Villegas Velásquez
EPI_ISL_2155042, EPI_ISL_3385771, EPI_ISL_3385790, EPI_ISL_3385796, EPI_ISL_3385855	COLCAN	Instituto Nacional de Salud- Dirección de Investigación en Salud Pública	Carlos Franco-Muñoz; Carmen Osorio; Diana Malo; Diego A. Álvarez-Díaz; Diego Andrés Prada; Gerardo Santamaría; Hector Alejandro Ruiz-Moreno; Jhonnatan Reales-González; Jorge Rivera; Juan Camilo Martinez; Julian Naizaque; Katherine Laiton-Donato; Lisseth Pardo; Magdalena Wiesner; Marcela Mercado-Reyes; Maria T. Herrera-Sepúlveda; Marta Lopez Blanco; Martha Lucia Ospina Martinez; Paola Rojas; Sergio Gomez; Sheryll Corchuelo; Ángela Alarcon Cruz
EPI_ISL_2674313, EPI_ISL_2674314, EPI_ISL_2674315, EPI_ISL_2674316	COMFANDI - SAN NICOLAS	Instituto Nacional de Salud- Dirección de Investigación en Salud Pública	Carlos Franco-Muñoz; Carmen Osorio; Diana Malo; Diego A. Álvarez-Díaz; Diego Andrés Prada; Gerardo Santamaría; Hector Alejandro Ruiz-Moreno; Jhonnatan Reales-González; Jorge Rivera; Juan Camilo Martinez; Julian Naizaque; Katherine Laiton-Donato; Lisseth Pardo; Magdalena Wiesner; Marcela Mercado-Reyes; Maria T. Herrera-Sepúlveda; Marta Lopez Blanco; Martha Lucia Ospina Martinez; Paola Rojas; Sergio Gomez; Sheryll Corchuelo; Ángela Alarcon Cruz
EPI_ISL_1632500, EPI_ISL_1632501, EPI_ISL_1632502	CONFANDI SAN NICOLAS	Instituto Nacional de Salud- Dirección de Investigación en Salud Pública	Carlos Franco-Muñoz; Carmen Osorio; Diana Malo; Diego A. Álvarez-Díaz; Diego Andrés Prada; Gerardo Santamaría; Hector Alejandro Ruiz-Moreno; Jhonnatan Reales-González; Jorge Rivera; Juan Camilo Martinez; Julian Naizaque; Katherine Laiton-Donato; Lisseth Pardo; Magdalena Wiesner; Marcela Mercado-Reyes; Maria T. Herrera-Sepúlveda; Marta Lopez Blanco; Martha Lucia Ospina Martinez; Paola Rojas; Sergio Gomez; Sheryll Corchuelo; Ángela Alarcon Cruz
EPI_ISL_2674337	CORPOMEDICA	Instituto Nacional de Salud- Dirección de Investigación en Salud Pública	Carlos Franco-Muñoz; Carmen Osorio; Diana Malo; Diego A. Álvarez-Díaz; Diego Andrés Prada; Gerardo Santamaría; Hector Alejandro Ruiz-Moreno; Jhonnatan Reales-González; Jorge Rivera; Juan Camilo Martinez; Julian Naizaque; Katherine Laiton-Donato; Lisseth Pardo; Magdalena Wiesner; Marcela Mercado-Reyes; Maria T. Herrera-Sepúlveda; Marta Lopez Blanco; Martha Lucia Ospina Martinez; Paola Rojas; Sergio Gomez; Sheryll Corchuelo; Ángela Alarcon Cruz
EPI_ISL_2657867, EPI_ISL_2657868	COVID 19- EPS FAMISANAR	Instituto Nacional de Salud- Dirección de Investigación en Salud Pública	Carlos Franco-Muñoz; Carmen Osorio; Diana Malo; Diego A. Álvarez-Díaz; Diego Andrés Prada; Gerardo Santamaría; Hector Alejandro Ruiz-Moreno; Jhonnatan Reales-González; Jorge Rivera; Juan Camilo Martinez; Julian Naizaque; Katherine Laiton-Donato; Lisseth Pardo; Magdalena Wiesner; Marcela Mercado-Reyes; Maria T. Herrera-Sepúlveda; Marta Lopez Blanco; Martha Lucia Ospina Martinez; Paola Rojas; Sergio Gomez; Sheryll Corchuelo; Ángela Alarcon Cruz
EPI_ISL_1220046	Carvajal Laboratorio IPS SAS	Instituto Nacional de Salud- Dirección de Investigación en Salud Pública	Carlos Franco-Muñoz; Diego A. Álvarez-Díaz; Diego Andrés Prada; Gerardo Santamaría; Hector Alejandro Ruiz-Moreno; Jhonnatan Reales-González; Julian Naizaque; Katherine Laiton-Donato; Magdalena Wiesner; Marcela Mercado-Reyes.; Maria T. Herrera-Sepúlveda; Martha Lucia Ospina Martínez; Sheryll Corchuelo
EPI_ISL_794663	Carvajal Laboratorios	Instituto Nacional de Salud - Dirección de Investigación en Salud Pública	Carlos Franco-Muñoz; Diego A. Álvarez-Díaz; Diego Andrés Prada; Gerardo Santamaría; Jonathan Reales; Julian Naizaque; Katherine Laiton-Donato; Magdalena Wiesner; Marcela Mercado-Reyes; Maria T. Herrera; Martha Lucia Ospina Martinez; Mauricio Pacheco-Montealegre; Paola Muñoz-Laiton; Sheryl Corchuelo
EPI_ISL_2674351, EPI_ISL_2674352	Centro de Atención y Daignóstico de Enfermedades Infecciosas-CDI	Instituto Nacional de Salud- Dirección de Investigación en Salud Pública	Carlos Franco-Muñoz; Carmen Osorio; Diana Malo; Diego A. Álvarez-Díaz; Diego Andrés Prada; Gerardo Santamaría; Hector Alejandro Ruiz-Moreno; Jhonnatan Reales-González; Jorge Rivera; Juan Camilo Martinez; Julian Naizaque; Katherine Laiton-Donato; Lisseth Pardo; Magdalena Wiesner; Marcela Mercado-Reyes; Maria T. Herrera-Sepúlveda; Marta Lopez Blanco; Martha Lucia Ospina Martinez; Paola Rojas; Sergio Gomez; Sheryll Corchuelo; Ángela Alarcon Cruz
EPI_ISL_1494948	Centro de Atención y Diagnóstico de Enfermedades Infecciosas	Instituto Nacional de Salud- Dirección de Investigación en Salud Pública	Carlos Franco-Muñoz; Carmen Osorio; Diana Malo; Diego A. Álvarez-Díaz; Diego Andrés Prada; Gerardo Santamaría; Hector Alejandro Ruiz-Moreno; Jhonnatan Reales-González; Juan Camilo Martinez; Julian Naizaque; Katherine Laiton-Donato; Lisseth Pardo; Magdalena Wiesner; Marcela Mercado-Reyes; Maria T. Herrera-Sepúlveda; Marta Lopez Blanco; Martha Lucia Ospina Martinez; Paola Rojas; Sergio Gomez; Sheryll Corchuelo; Ángela Alarcon Cruz
EPI_ISL_2339875, EPI_ISL_2339876, EPI_ISL_2339877, EPI_ISL_2339878, EPI_ISL_2339879, EPI_ISL_2339880, EPI_ISL_2339881, EPI_ISL_2339882, EPI_ISL_2339883, EPI_ISL_2339884, EPI_ISL_2339885, EPI_ISL_2339886, EPI_ISL_2339887, EPI_ISL_2339888, EPI_ISL_2339889, EPI_ISL_2339890, EPI_ISL_2339891, EPI_ISL_2339892, EPI_ISL_2339893, EPI_ISL_2339894, EPI_ISL_2339895, EPI_ISL_2339896, EPI_ISL_2339897, EPI_ISL_2339898, EPI_ISL_2339899, EPI_ISL_2339900, EPI_ISL_2339901, EPI_ISL_2339902, EPI_ISL_2339903, EPI_ISL_2339904, EPI_ISL_2339905, EPI_ISL_2339906, EPI_ISL_2339907, EPI_ISL_2339908, EPI_ISL_2339909			
see above	Centro de Estudio de Enfermedades Autoinmunes (CREA), Universidad del Rosario, Bogota, Colombia	Centro de Investigaciones en Microbiología y Biotecnología-UR (CIMBIUR), Facultad de Ciencias Naturales, Universidad del Rosario, Bogotá, Colombia	Carolina Ramírez-Santana; Gustavo Salguero; Juan David Ramírez; Juan Esteban Gallo; Juan-Manuel Anaya; Luz H. Patiño; Marina Muñoz; Nathalia Ballesteros; Sergio Castañeda
EPI_ISL_941104, EPI_ISL_941105, EPI_ISL_941106, EPI_ISL_941107, EPI_ISL_941108, EPI_ISL_941109, EPI_ISL_941110, EPI_ISL_941111, EPI_ISL_941112, EPI_ISL_941113, EPI_ISL_941114, EPI_ISL_941115, EPI_ISL_941116, EPI_ISL_941117, EPI_ISL_941118, EPI_ISL_941119, EPI_ISL_941120, EPI_ISL_941121, EPI_ISL_941122, EPI_ISL_941123, EPI_ISL_941124, EPI_ISL_941125, EPI_ISL_941126, EPI_ISL_941127, EPI_ISL_941128, EPI_ISL_941129, EPI_ISL_941130, EPI_ISL_941131, EPI_ISL_941132, EPI_ISL_941133, EPI_ISL_941134, EPI_ISL_941135, EPI_ISL_941136, EPI_ISL_941137, EPI_ISL_941138, EPI_ISL_941139, EPI_ISL_941140, EPI_ISL_941141, EPI_ISL_941142, EPI_ISL_941143, EPI_ISL_941144, EPI_ISL_941145, EPI_ISL_941146, EPI_ISL_941147, EPI_ISL_941148, EPI_ISL_941149, EPI_ISL_941150, EPI_ISL_941151, EPI_ISL_941152, EPI_ISL_941153, EPI_ISL_941154, EPI_ISL_941155, EPI_ISL_941156, EPI_ISL_941157, EPI_ISL_941158, EPI_ISL_941159, EPI_ISL_941160, EPI_ISL_941161, EPI_ISL_941162			
see above	Centro de Investigaciones en Microbiología y Biotecnología-UR (CIMBIUR), Facultad de Ciencias Naturales, Universidad del Rosario, Bogotá, Colombia	Centro de Investigaciones en Microbiología y Biotecnología-UR (CIMBIUR), Facultad de Ciencias Naturales, Universidad del Rosario, Bogotá, Colombia Icahn School of Medicine at Mount Sinai, New York, USA	Adriana van de Guchte; Alberto Paniz-Mondolfi; Alejandro Feged-Rivadeneira; Ana S. Gonzalez-Reiche; Andrés Angel; Carolina Flórez; Carolina Hernández; Emilia Mia Sordillo; Felipe González-Casabianca; Hala Alejel Alshammary; Harm van Bakel; Iván Carroll; Jaime Cascante; Jayeeta Dutta; Juan David Ramírez; Luz Helena Patiño; Marina Muñoz; Matthew M. Hernandez; Mauricio Santos-Vega; Mónica Palma-Cuero; Nathalia Ballesteros; Sergio Gomez; Viviana Simon; Zenab Khan
EPI_ISL_941941, EPI_ISL_941944, EPI_ISL_941945, EPI_ISL_941946, EPI_ISL_941947, EPI_ISL_941948, EPI_ISL_941950, EPI_ISL_941952, EPI_ISL_941954, EPI_ISL_941959, EPI_ISL_941961, EPI_ISL_941962, EPI_ISL_941964, EPI_ISL_941965, EPI_ISL_941966, EPI_ISL_941967, EPI_ISL_941968, EPI_ISL_941969, EPI_ISL_941970, EPI_ISL_941971, EPI_ISL_941972, EPI_ISL_941973, EPI_ISL_941974, EPI_ISL_941975, EPI_ISL_941976, EPI_ISL_941977, EPI_ISL_941978, EPI_ISL_941979, EPI_ISL_941980, EPI_ISL_941981, EPI_ISL_941982, EPI_ISL_941983, EPI_ISL_941995, EPI_ISL_941996, EPI_ISL_941997, EPI_ISL_941998, EPI_ISL_941999, EPI_ISL_942000, EPI_ISL_942001, EPI_ISL_942002, EPI_ISL_942004, EPI_ISL_942005, EPI_ISL_944634, EPI_ISL_944635, EPI_ISL_944636, EPI_ISL_944637, EPI_ISL_944638			
see above	Centro de Investigaciones en Microbiología y Biotecnología-UR (CIMBIUR), Facultad de Ciencias Naturales, Universidad del Rosario, Bogotá, Colombia	Centro de Investigaciones en Microbiología y Biotecnología-UR (CIMBIUR), Facultad de Ciencias Naturales, Universidad del Rosario, Bogotá, Colombia Instituto Nacional de Salud, Bogotá, Colombia Icahn School of Medicine at Mount Sinai, New York, USA	Adriana van de Guchte; Alberto Paniz-Mondolfi; Ana S. Gonzalez-Reiche; Carolina Flórez; Carolina Hernández; Emilia Mia Sordillo; Hala Alejel Alshammary; Harm van Bakel; Jayeeta Dutta; Juan David Ramírez; Luz Helena Patiño; Marina Muñoz; Matthew M. Hernandez; Nathalia Ballesteros; Sergio Gomez; Viviana Simon; Zenab Khan
EPI_ISL_3721575, EPI_ISL_3721576, EPI_ISL_3721581, EPI_ISL_3721584, EPI_ISL_3721588, EPI_ISL_3721589, EPI_ISL_3721591, EPI_ISL_3721598, EPI_ISL_3721617			
see above	Clinica SOMER Rionegro	Universidad Nacional de Colombia - Laboratorio Genómico One Health	Andres F. Cardona-Rios; Carlos Franco-Muñoz; Carolina Muñoz-Arango; Celeny Ortiz; Daniel O. Maldonado-Perez; Diego A. Álvarez-Díaz; Hector Alejandro Ruiz-Moreno; Idabely Betancur Ortiz; Jorge E. Osorio; Juan P. Hernandez-Ortiz; Karl A Ciuoderis; Katherine Laiton-Donato; Laura Silvana Perez; Lina M. Hurtado; Marcela Mercado-Reyes; Maria Angélica Maya; Maria Stella López; Rita Almanza Payares; Sandra Ines Cano; Simón Villegas Velásquez
EPI_ISL_1494958	Clinica Universitaria Medicina Integral - Laboratorio Molecular	Instituto Nacional de Salud- Dirección de Investigación en Salud Pública	Carlos Franco-Muñoz; Carmen Osorio; Diana Malo; Diego A. Álvarez-Díaz; Diego Andrés Prada; Gerardo Santamaría; Hector Alejandro Ruiz-Moreno; Jhonnatan Reales-González; Juan Camilo Martinez; Julian Naizaque; Katherine Laiton-Donato; Lisseth Pardo; Magdalena Wiesner; Marcela Mercado-Reyes; Maria T. Herrera-Sepúlveda; Marta Lopez Blanco; Martha Lucia Ospina Martinez; Paola Rojas; Sergio Gomez; Sheryll Corchuelo; Ángela Alarcon Cruz
EPI_ISL_1303368	Clinica de la Costa	Instituto Nacional de Salud- Dirección de Investigación en Salud Pública	Carlos Franco-Muñoz; Carmen Osorio; Diana Malo; Diego A. Álvarez-Díaz; Diego Andrés Prada; Gerardo Santamaría; Hector Alejandro Ruiz-Moreno; Jhonnatan Reales-González; Juan Camilo Martinez; Julian Naizaque; Katherine Laiton-Donato; Lisseth Pardo; Magdalena Wiesner; Marcela Mercado-Reyes; Maria T. Herrera-Sepúlveda; Marta Lopez Blanco; Martha Lucia Ospina Martinez; Sergio Gomez; Sheryll Corchuelo; Ángela Alarcon Cruz
EPI_ISL_1017711	Clinica del norte	Instituto Nacional de Salud- Dirección de Investigación en Salud Pública	Carlos Franco-Muñoz; Diego A. Álvarez-Díaz; Diego Andrés Prada; Gerardo Santamaría; Hector Alejandro Ruiz-Moreno; Jhonnatan Reales-González; Julian Naizaque; Katherine Laiton-Donato; Magdalena Wiesner; Marcela Mercado-Reyes; Maria T. Herrera-Sepúlveda; Martha Lucia Ospina Martinez; Sheryll Corchuelo
EPI_ISL_1805633, EPI_ISL_1820962, EPI_ISL_1821066, EPI_ISL_1821067, EPI_ISL_1821068	Clinica los Nogales	Instituto Nacional de Salud- Dirección de Investigación en Salud Pública	Carlos Franco-Muñoz; Carmen Osorio; Diana Malo; Diego A. Álvarez-Díaz; Diego Andrés Prada; Gerardo Santamaría; Hector Alejandro Ruiz-Moreno; Jhonnatan Reales-González; Jorge Rivera; Juan Camilo Martinez; Julian Naizaque; Katherine Laiton-Donato; Lisseth Pardo; Magdalena Wiesner; Marcela Mercado-Reyes; Maria T. Herrera-Sepúlveda; Marta Lopez Blanco; Martha Lucia Ospina Martinez; Paola Rojas; Sergio Gomez; Sheryll Corchuelo; Ángela Alarcon Cruz
EPI_ISL_2142003, EPI_ISL_2148602, EPI_ISL_2150975, EPI_ISL_2151000, EPI_ISL_2151070, EPI_ISL_2151071, EPI_ISL_2151072, EPI_ISL_2151105, EPI_ISL_2151106, EPI_ISL_2151107, EPI_ISL_2151121, EPI_ISL_2151155			
see above	Clínica Meta	Instituto de Virología-Universidad El Bosque	Adriana Franco; Andres F. Cardona-Rios; Carolina Coronel-Ruiz; Eliana P. Calvo; Felix G. Delgado; Jaime E. Castellanos; Jhann Andrés Arturo; Jorge E. Osorio; Juan P. Hernandez-Ortiz; Karl A Ciuoderis; L. Johana Madroñero; Lady Lopez; Laura Silvana Pérez; Lilia Bernal-Cepeda; Maria Fernanda Montañez-Suarez; Maria Stella López; María Angélica Calderón-Peláez; Sigrid J. Camacho-Ortega; Sonia P. Bohórquez; Myriam Lucia Velandia-Romero
EPI_ISL_1494957	Clínica Primavera	Instituto Nacional de Salud- Dirección de Investigación en Salud Pública	Carlos Franco-Muñoz; Carmen Osorio; Diana Malo; Diego A. Álvarez-Díaz; Diego Andrés Prada; Gerardo Santamaría; Hector Alejandro Ruiz-Moreno; Jhonnatan Reales-González; Juan Camilo Martinez; Julian Naizaque; Katherine Laiton-Donato; Lisseth Pardo; Magdalena Wiesner; Marcela Mercado-Reyes; Maria T. Herrera-Sepúlveda; Marta Lopez Blanco; Martha Lucia Ospina Martinez; Paola Rojas; Sergio Gomez; Sheryll Corchuelo; Ángela Alarcon Cruz
EPI_ISL_1494952, EPI_ISL_1494953	Comfandi	Instituto Nacional de Salud- Dirección de Investigación en Salud Pública	Carlos Franco-Muñoz; Carmen Osorio; Diana Malo; Diego A. Álvarez-Díaz; Diego Andrés Prada; Gerardo Santamaría; Hector Alejandro Ruiz-Moreno; Jhonnatan Reales-González; Juan Camilo Martinez; Julian Naizaque; Katherine Laiton-Donato; Lisseth Pardo; Magdalena Wiesner; Marcela Mercado-Reyes; Maria T. Herrera-Sepúlveda; Marta Lopez Blanco; Martha Lucia Ospina Martinez; Paola Rojas; Sergio Gomez; Sheryll Corchuelo; Ángela Alarcon Cruz
EPI_ISL_3368470, EPI_ISL_3368474, EPI_ISL_3368475, EPI_ISL_3368476, EPI_ISL_4417562, EPI_ISL_4417582, EPI_ISL_4417583, EPI_ISL_4417584, EPI_ISL_4417613, EPI_ISL_4417618, EPI_ISL_4417636, EPI_ISL_4417646, EPI_ISL_4417650, EPI_ISL_4417651, EPI_ISL_4417654, EPI_ISL_4417655, EPI_ISL_4417656, EPI_ISL_4417658			
see above	Compensar Calle 63	Centro de Investigaciones en Microbiología y Biotecnología-UR (CIMBIUR), Facultad de Ciencias Naturales, Universidad del Rosario, Bogotá, Colombia	Angie Ramírez; Juan David Ramírez; Luz H. Patiño; Marcela Mercado-Reyes; Marina Muñoz; Nathalia Ballesteros; Nicolas Niño; Sergio Castañeda
EPI_ISL_2009069, EPI_ISL_2155043, EPI_ISL_2155044, EPI_ISL_2158345	DIRECCION DE SANIDAD EJERCITO NACIONAL	Instituto Nacional de Salud- Dirección de Investigación en Salud Pública	Carlos Franco-Muñoz; Carmen Osorio; Diana Malo; Diego A. Álvarez-Díaz; Diego Andrés Prada; Gerardo Santamaría; Hector Alejandro Ruiz-Moreno; Jhonnatan Reales-González; Jorge Rivera; Juan Camilo Martinez; Julian Naizaque; Katherine Laiton-Donato; Lisseth Pardo; Magdalena Wiesner; Marcela Mercado-Reyes; Maria T. Herrera-Sepúlveda; Marta Lopez Blanco; Martha Lucia Ospina Martinez; Paola Rojas; Sergio Gomez; Sheryll Corchuelo; Ángela Alarcon Cruz

EPI_ISL_794658	DIRECCION DE SANIDAD POLICIA NACIONAL	Instituto Nacional de Salud - Dirección de Investigación en Salud Pública	Carlos Franco-Muñoz; Diego A. Álvarez-Díaz; Diego Andrés Prada; Gerardo Santamaría; Jonathan Reales; Julian Naizaque; Katherine Laiton-Donato; Magdalena Wiesner; Marcela Mercado-Reyes; Maria T. Herrera; Martha Lucia Ospina Martinez; Mauricio Pacheco-Montealegre; Paola Muñoz-Laiton; Sheryl Corchuelo
EPI_ISL_906550, EPI_ISL_906551	DIRECCION DEPARTAMENTAL DE SALUD DE AMAZONAS	Instituto Nacional de Salud- Dirección de Investigación en Salud Pública, Universidad de los Andes- Applied genomics research group, Vicerrectoria de Investigación y Creación, Universidad de los Andes- Systems and Computing Engineering Department	Carlos Franco-Muñoz; Diego A. Álvarez-Díaz; Diego Andrés Prada; Gerardo Santamaría Jorge Duitama; Héctor Alejandro Ruiz-Moreno; Jhonnatan Reales-González; Jorge Ivan Diaz; Julian Naizaque; Katherine Laiton-Donato; Laura Natalia Gonzalez; Magdalena Wiesner; Marcela Mercado-Reyes; Maria T. Herrera-Sepúlveda; Martha Lucia Ospina Martinez; Mauricio Pacheco-Montealegre; Sheryll Corchuelo; Silvia Restrepo-Restrepo
EPI_ISL_906556	DIRECCION DEPARTAMENTAL DE SALUD DE GUAJIRA	Instituto Nacional de Salud- Dirección de Investigación en Salud Pública, Universidad de los Andes- Applied genomics research group, Vicerrectoria de Investigación y Creación, Universidad de los Andes- Systems and Computing Engineering Department	Carlos Franco-Muñoz; Diego A. Álvarez-Díaz; Diego Andrés Prada; Gerardo Santamaría Jorge Duitama; Héctor Alejandro Ruiz-Moreno; Jhonnatan Reales-González; Jorge Ivan Diaz; Julian Naizaque; Katherine Laiton-Donato; Laura Natalia Gonzalez; Magdalena Wiesner; Marcela Mercado-Reyes; Maria T. Herrera-Sepúlveda; Martha Lucia Ospina Martinez; Mauricio Pacheco-Montealegre; Sheryll Corchuelo; Silvia Restrepo-Restrepo
EPI_ISL_2834726, EPI_ISL_2834728	DISAN POLICIA NACIONAL	Instituto Nacional de Salud- Dirección de Investigación en Salud Pública	Carlos Franco-Muñoz; Carmen Osorio; Diana Malo; Diego A. Álvarez-Díaz; Diego Andrés Prada; Gerardo Santamaría; Hector Alejandro Ruiz-Moreno; Jhonnatan Reales-González; Jorge Rivera; Juan Camilo Martinez; Julian Naizaque; Katherine Laiton-Donato; Lisseth Pardo; Magdalena Wiesner; Marcela Mercado-Reyes; Maria T. Herrera-Sepúlveda; Marta Lopez Blanco; Martha Lucia Ospina Martinez; Paola Rojas; Sergio Gomez; Sheryll Corchuelo; Ángela Alarcon Cruz
EPI_ISL_4028598, EPI_ISL_4028608, EPI_ISL_4028609	DISAN POLICIA NACIONAL	Molecular Genetics and Antimicrobial Resistance - UGRA, Universidad El Bosque	Betsy Castro; Catalina Espitia; Jinnethe Reyes; Lorena Diaz; Marcela Mercado; Mauricio Pacheco; Rafael Rios; Sandra Rincon; Valentina Martinez
EPI_ISL_794661	DRECCION DE SANIDAD POLICIA NACIONAL	Instituto Nacional de Salud - Dirección de Investigación en Salud Pública	Carlos Franco-Muñoz; Diego A. Álvarez-Díaz; Diego Andrés Prada; Gerardo Santamaría; Jonathan Reales; Julian Naizaque; Katherine Laiton-Donato; Magdalena Wiesner; Marcela Mercado-Reyes; Maria T. Herrera; Martha Lucia Ospina Martinez; Mauricio Pacheco-Montealegre; Paola Muñoz-Laiton; Sheryl Corchuelo
EPI_ISL_4006772	Diagnóstico CIB	Corporación para Investigaciones Biológicas-CIB	Jeanneth Mosquera Rendon; Katterine Molina Hoyos; Marcela Mercado Reyes; Uriel A. Hurtado Paez
EPI_ISL_906529	Direccion de partamental de salud del Amazonas	Instituto Nacional de Salud- Dirección de Investigación en Salud Pública, Universidad de los Andes- Applied genomics research group, Vicerrectoria de Investigación y Creación, Universidad de los Andes- Systems and Computing Engineering Department	Carlos Franco-Muñoz; Diego A. Álvarez-Díaz; Diego Andrés Prada; Gerardo Santamaría Jorge Duitama; Héctor Alejandro Ruiz-Moreno; Jhonnatan Reales-González; Jorge Ivan Diaz; Julian Naizaque; Katherine Laiton-Donato; Laura Natalia Gonzalez; Magdalena Wiesner; Marcela Mercado-Reyes; Maria T. Herrera-Sepúlveda; Martha Lucia Ospina Martinez; Mauricio Pacheco-Montealegre; Sheryll Corchuelo; Silvia Restrepo-Restrepo
EPI_ISL_902736	Dirección Departamental de Salud de Pública Leticia	Instituto Nacional de Salud- Dirección de Investigación en Salud Pública, Universidad de los Andes- Applied genomics research group, Vicerrectoria de Investigación y Creación, Universidad de los Andes- Systems and Computing Engineering Department	Carlos Franco-Muñoz; Diego A. Álvarez-Díaz; Diego Andrés Prada; Jorge Duitama; Jorge Ivan Diaz; Katherine Laiton-Donato; Laura Natalia Gonzalez; Magdalena Wiesner; Marcela Mercado-Reyes; Maria T. Herrera-Sepúlveda; Martha Lucia Ospina Martinez; Mauricio Pacheco-Montealegre; Silvia Restrepo-Restrepo
EPI_ISL_845621, EPI_ISL_845622, EPI_ISL_845623, EPI_ISL_845624, EPI_ISL_845625	Dirección de Sanidad Ejército	Instituto Nacional de Salud - Dirección de Investigación en Salud Pública	Carlos Franco-Muñoz; Diego A. Álvarez-Díaz; Diego Andrés Prada; Gerardo Santamaría; Jonathan Reales; Julian Naizaque; Katherine Laiton-Donato; Magdalena Wiesner; Marcela Mercado-Reyes; Maria T. Herrera-Sepúlveda; Martha Lucia Ospina Martinez; Mauricio Pacheco-Montealegre; Paola Muñoz-Laiton; Sheryll Corchuelo
EPI_ISL_1137618, EPI_ISL_1303371, EPI_ISL_1303372, EPI_ISL_1303373	Dirección de Sanidad Policia Nacional	Instituto Nacional de Salud- Dirección de Investigación en Salud Pública	Carlos Franco-Muñoz; Carmen Osorio; Diana Malo; Diego A. Álvarez-Díaz; Diego Andrés Prada; Gerardo Santamaría; Hector Alejandro Ruiz-Moreno; Jhonnatan Reales-González; Juan Camilo Martinez; Julian Naizaque; Katherine Laiton-Donato; Lisseth Pardo; Magdalena Wiesner; Marcela Mercado-Reyes; Marcela Mercado-Reyes.; Maria T. Herrera-Sepúlveda; Marta Lopez Blanco; Martha Lucia Ospina Martinez; Martha Lucia Ospina Martínez; Sergio Gomez; Sheryll Corchuelo; Ángela Alarcon Cruz
EPI_ISL_3391977, EPI_ISL_3391979, EPI_ISL_3391983	Disan Policia Nacional	Instituto Nacional de Salud- Dirección de Investigación en Salud Pública	Carlos Franco-Muñoz; Carmen Osorio; Diana Malo; Diego A. Álvarez-Díaz; Diego Andrés Prada; Gerardo Santamaría; Hector Alejandro Ruiz-Moreno; Jhonnatan Reales-González; Jorge Rivera; Juan Camilo Martinez; Julian Naizaque; Katherine Laiton-Donato; Lisseth Pardo; Magdalena Wiesner; Marcela Mercado-Reyes; Maria T. Herrera-Sepúlveda; Marta Lopez Blanco; Martha Lucia Ospina Martinez; Paola Rojas; Sergio Gomez; Sheryll Corchuelo; Ángela Alarcon Cruz
EPI_ISL_1220066, EPI_ISL_1582978, EPI_ISL_1582979, EPI_ISL_1582980, EPI_ISL_2828045, EPI_ISL_2828046, EPI_ISL_2828047, EPI_ISL_2828048, EPI_ISL_2828049, EPI_ISL_2828050, EPI_ISL_2828051, EPI_ISL_2828052, EPI_ISL_2828053			
see above	E.S.E. HOSPITAL SAN JOSE DE MAICAO	Instituto Nacional de Salud- Dirección de Investigación en Salud Pública	Carlos Franco-Muñoz; Carmen Osorio; Diana Malo; Diego A. Álvarez-Díaz; Diego Andrés Prada; Gerardo Santamaría; Hector Alejandro Ruiz-Moreno; Jhonnatan Reales-González; Jorge Rivera; Juan Camilo Martinez; Julian Naizaque; Katherine Laiton-Donato; Lisseth Pardo; Magdalena Wiesner; Marcela Mercado-Reyes; Marcela Mercado-Reyes.; Maria T. Herrera-Sepúlveda; Marta Lopez Blanco; Martha Lucia Ospina Martinez; Martha Lucia Ospina Martínez; Paola Rojas; Sergio Gomez; Sheryll Corchuelo; Ángela Alarcon Cruz
EPI_ISL_906545	E.S.E. HOSPITAL SAN JOSE DE MAICAO	Instituto Nacional de Salud- Dirección de Investigación en Salud Pública, Universidad de los Andes- Applied genomics research group, Vicerrectoria de Investigación y Creación, Universidad de los Andes- Systems and Computing Engineering Department	Carlos Franco-Muñoz; Diego A. Álvarez-Díaz; Diego Andrés Prada; Gerardo Santamaría Jorge Duitama; Héctor Alejandro Ruiz-Moreno; Jhonnatan Reales-González; Jorge Ivan Diaz; Julian Naizaque; Katherine Laiton-Donato; Laura Natalia Gonzalez; Magdalena Wiesner; Marcela Mercado-Reyes; Maria T. Herrera-Sepúlveda; Martha Lucia Ospina Martinez; Mauricio Pacheco-Montealegre; Sheryll Corchuelo; Silvia Restrepo-Restrepo
EPI_ISL_1220053, EPI_ISL_2674349	EMPRESA SOCIAL DEL ESTADO HOSPITAL UNIVERSITARIO DE SANTANDER	Instituto Nacional de Salud- Dirección de Investigación en Salud Pública	Carlos Franco-Muñoz; Carmen Osorio; Diana Malo; Diego A. Álvarez-Díaz; Diego Andrés Prada; Gerardo Santamaría; Hector Alejandro Ruiz-Moreno; Jhonnatan Reales-González; Jorge Rivera; Juan Camilo Martinez; Julian Naizaque; Katherine Laiton-Donato; Lisseth Pardo; Magdalena Wiesner; Marcela Mercado-Reyes; Marcela Mercado-Reyes.; Maria T. Herrera-Sepúlveda; Marta Lopez Blanco; Martha Lucia Ospina Martinez; Martha Lucia Ospina Martínez; Paola Rojas; Sergio Gomez; Sheryll Corchuelo; Ángela Alarcon Cruz
EPI_ISL_956304, EPI_ISL_956305	ESE INSTITUTO NACIONAL DE CANCEROLOGIA	Instituto Nacional de Salud- Dirección de Investigación en Salud Pública	Carlos Franco-Muñoz; Diego A. Álvarez-Díaz; Diego Andrés Prada; Gerardo Santamaría; Hector Alejandro Ruiz-Moreno; Jhonnatan Reales-González; Julian Naizaque; Katherine Laiton-Donato; Magdalena Wiesner; Marcela Mercado-Reyes; Maria T. Herrera-Sepúlveda; Martha Lucia Ospina Martinez; Mauricio Pacheco-Montealegre; Sheryll Corchuelo
EPI_ISL_1494949	ESE. SAN ISIDRO DEL MUNICIPIO DE TONA	Instituto Nacional de Salud- Dirección de Investigación en Salud Pública	Carlos Franco-Muñoz; Carmen Osorio; Diana Malo; Diego A. Álvarez-Díaz; Diego Andrés Prada; Gerardo Santamaría; Hector Alejandro Ruiz-Moreno; Jhonnatan Reales-González; Juan Camilo Martinez; Julian Naizaque; Katherine Laiton-Donato; Lisseth Pardo; Magdalena Wiesner; Marcela Mercado-Reyes; Maria T. Herrera-Sepúlveda; Marta Lopez Blanco; Martha Lucia Ospina Martinez; Paola Rojas; Sergio Gomez; Sheryll Corchuelo; Ángela Alarcon Cruz
EPI_ISL_2362561	FUNDACION CARDIO INFANTIL	Instituto Nacional de Salud- Dirección de Investigación en Salud Pública	Carlos Franco-Muñoz; Carmen Osorio; Diana Malo; Diego A. Álvarez-Díaz; Diego Andrés Prada; Gerardo Santamaría; Hector Alejandro Ruiz-Moreno; Jhonnatan Reales-González; Jorge Rivera; Juan Camilo Martinez; Julian Naizaque; Katherine Laiton-Donato; Lisseth Pardo; Magdalena Wiesner; Marcela Mercado-Reyes; Maria T. Herrera-Sepúlveda; Marta Lopez Blanco; Martha Lucia Ospina Martinez; Paola Rojas; Sergio Gomez; Sheryll Corchuelo; Ángela Alarcon Cruz
EPI_ISL_3459385	FUNDACION CARDIOINFANTIL	Instituto Nacional de Salud	Carlos Franco-Muñoz; Carmen Osorio; Diana Malo; Diego A. Álvarez-Díaz; Diego Andrés Prada; Gerardo Santamaría; Hector Alejandro Ruiz-Moreno; Jhonnatan Reales-González; Jorge Rivera; Juan Camilo Martinez; Julian Naizaque; Katherine Laiton-Donato; Lisseth Pardo; Magdalena Wiesner; Marcela Mercado-Reyes; Maria T. Herrera-Sepúlveda; Marta Lopez Blanco; Martha Lucia Ospina Martinez; Martha Patricia Sánchez Zorro; Paola Rojas; Rodrigo Cabrera Perez; Sergio Gomez; Sheryll Corchuelo; Ángela Alarcon Cruz
EPI_ISL_845626	FUNDACION CARDIOINFANTIL	Instituto Nacional de Salud - Dirección de Investigación en Salud Pública	Carlos Franco-Muñoz; Diego A. Álvarez-Díaz; Diego Andrés Prada; Gerardo Santamaría; Jonathan Reales; Julian Naizaque; Katherine Laiton-Donato; Magdalena Wiesner; Marcela Mercado-Reyes; Maria T. Herrera-Sepúlveda; Martha Lucia Ospina Martinez; Mauricio Pacheco-Montealegre; Paola Muñoz-Laiton; Sheryll Corchuelo
EPI_ISL_3066763, EPI_ISL_3398803, EPI_ISL_3398804, EPI_ISL_3398805, EPI_ISL_3398806, EPI_ISL_3398807, EPI_ISL_3398808, EPI_ISL_3398809			
see above	FUNDACION CARDIOVASCULAR DE COLOMBIA	Instituto Nacional de Salud	Carlos Franco-Muñoz; Carmen Osorio; Diana Malo; Diego A. Álvarez-Díaz; Diego Andrés Prada; Gerardo Santamaría; Hector Alejandro Ruiz-Moreno; Jhonnatan Reales-González; Jorge Rivera; Juan Camilo Martinez; Julian Naizaque; Katherine Laiton-Donato; Lisseth Pardo; Magdalena Wiesner; Marcela Mercado-Reyes; Maria T.Herrera-Sepúlveda; Marta Lopez Blanco; Martha Lucia Ospina Martinez; Paola Rojas; Sergio Gomez; Sheryll Corchuelo; Ángela Alarcon Cruz
EPI_ISL_3385831	FUNDACION CARDIOVASCULAR DE COLOMBIA	Instituto Nacional de Salud- Dirección de Investigación en Salud Pública	Carlos Franco-Muñoz; Carmen Osorio; Diana Malo; Diego A. Álvarez-Díaz; Diego Andrés Prada; Gerardo Santamaría; Hector Alejandro Ruiz-Moreno; Jhonnatan Reales-González; Jorge Rivera; Juan Camilo Martinez; Julian Naizaque; Katherine Laiton-Donato; Lisseth Pardo; Magdalena Wiesner; Marcela Mercado-Reyes; Maria T. Herrera-Sepúlveda; Marta Lopez Blanco; Martha Lucia Ospina Martinez; Paola Rojas; Sergio Gomez; Sheryll Corchuelo; Ángela Alarcon Cruz
EPI_ISL_2158349	FUNDACION HOSPITAL SAN PEDRO	Instituto Nacional de Salud- Dirección de Investigación en Salud Pública	Carlos Franco-Muñoz; Carmen Osorio; Diana Malo; Diego A. Álvarez-Díaz; Diego Andrés Prada; Gerardo Santamaría; Hector Alejandro Ruiz-Moreno; Jhonnatan Reales-González; Jorge Rivera; Juan Camilo Martinez; Julian Naizaque; Katherine Laiton-Donato; Lisseth Pardo; Magdalena Wiesner; Marcela Mercado-Reyes; Maria T. Herrera-Sepúlveda; Marta Lopez Blanco; Martha Lucia Ospina Martinez; Paola Rojas; Sergio Gomez; Sheryll Corchuelo; Ángela Alarcon Cruz
EPI_ISL_906543	FUNDACION HOSPITAL SAN PEDRO	Instituto Nacional de Salud- Dirección de Investigación en Salud Pública, Universidad de los Andes- Applied genomics research group, Vicerrectoria de Investigación y Creación, Universidad de los Andes- Systems and Computing Engineering Department	Carlos Franco-Muñoz; Diego A. Álvarez-Díaz; Diego Andrés Prada; Gerardo Santamaría Jorge Duitama; Héctor Alejandro Ruiz-Moreno; Jhonnatan Reales-González; Jorge Ivan Diaz; Julian Naizaque; Katherine Laiton-Donato; Laura Natalia Gonzalez; Magdalena Wiesner; Marcela Mercado-Reyes; Maria T. Herrera-Sepúlveda; Martha Lucia Ospina Martinez; Mauricio Pacheco-Montealegre; Sheryll Corchuelo; Silvia Restrepo-Restrepo
EPI_ISL_3385801	FUNDACION HOSPITAL SAN PEDRO DIOCESIS DE PASTO	Instituto Nacional de Salud- Dirección de Investigación en Salud Pública	Carlos Franco-Muñoz; Carmen Osorio; Diana Malo; Diego A. Álvarez-Díaz; Diego Andrés Prada; Gerardo Santamaría; Hector Alejandro Ruiz-Moreno; Jhonnatan Reales-González; Jorge Rivera; Juan Camilo Martinez; Julian Naizaque; Katherine Laiton-Donato; Lisseth Pardo; Magdalena Wiesner; Marcela Mercado-Reyes; Maria T. Herrera-Sepúlveda; Marta Lopez Blanco; Martha Lucia Ospina Martinez; Paola Rojas; Sergio Gomez; Sheryll Corchuelo; Ángela Alarcon Cruz
EPI_ISL_4300700	FUNDACION HOSPITALARIA SAN VICENTE DE PAUL	Instituto Nacional de Salud- Dirección de Investigación en Salud Pública	Carlos Franco-Muñoz; Carmen Osorio; Diana Malo; Diego A. Álvarez-Díaz; Diego Andrés Prada; Gerardo Santamaría; Hector Alejandro Ruiz-Moreno; Jhonnatan Reales-González; Jorge Rivera; Juan Camilo Martinez; Julian Naizaque; Katherine Laiton-Donato; Lisseth Pardo; Magdalena Wiesner; Marcela Mercado-Reyes; Maria T. Herrera-Sepúlveda; Marta Lopez Blanco; Martha Lucia Ospina Martinez; Paola Rojas; Sergio Gomez; Sheryll Corchuelo; Ángela Alarcon Cruz
EPI_ISL_3066765, EPI_ISL_3066766, EPI_ISL_3066767, EPI_ISL_3066768, EPI_ISL_3066769, EPI_ISL_3398811, EPI_ISL_3398812, EPI_ISL_3398813, EPI_ISL_3398814, EPI_ISL_3398815, EPI_ISL_3398816, EPI_ISL_3398817, EPI_ISL_3398818, EPI_ISL_3398819, EPI_ISL_3398820, EPI_ISL_3459379, EPI_ISL_3459380, EPI_ISL_3459394, EPI_ISL_3459412			
see above	FUNDACION VALLE DE LILI	Instituto Nacional de Salud	Carlos Franco-Muñoz; Carmen Osorio; Diana Malo; Diego A. Álvarez-Díaz; Diego Andrés Prada; Gerardo Santamaría; Hector Alejandro Ruiz-Moreno; Jhonnatan Reales-González; Jorge Rivera; Juan Camilo Martinez; Julian Naizaque; Katherine Laiton-Donato; Lisseth Pardo; Magdalena Wiesner; Marcela Mercado-Reyes; Maria T. Herrera-Sepúlveda; Maria T.Herrera-Sepúlveda; Marta Lopez Blanco; Martha Lucia Ospina Martinez; Paola Rojas; Sergio Gomez; Sheryll Corchuelo; Ángela Alarcon Cruz
EPI_ISL_1632497, EPI_ISL_1632498	FUNDACION VALLE DE LILI	Instituto Nacional de Salud- Dirección de Investigación en Salud Pública	Carlos Franco-Muñoz; Carmen Osorio; Diana Malo; Diego A. Álvarez-Díaz; Diego Andrés Prada; Gerardo Santamaría; Hector Alejandro Ruiz-Moreno; Jhonnatan Reales-González; Jorge Rivera; Juan Camilo Martinez; Julian Naizaque; Katherine Laiton-Donato; Lisseth Pardo; Magdalena Wiesner; Marcela Mercado-Reyes; Maria T. Herrera-Sepúlveda; Marta Lopez Blanco; Martha Lucia Ospina Martinez; Paola Rojas; Sergio Gomez; Sheryll Corchuelo; Ángela Alarcon Cruz
EPI_ISL_1220058, EPI_ISL_1220059, EPI_ISL_1220060	FUNDACION VALLE DEL LILI	Instituto Nacional de Salud- Dirección de Investigación en Salud Pública	Carlos Franco-Muñoz; Diego A. Álvarez-Díaz; Diego Andrés Prada; Gerardo Santamaría; Hector Alejandro Ruiz-Moreno; Jhonnatan Reales-González; Julian Naizaque; Katherine Laiton-Donato; Magdalena Wiesner; Marcela Mercado-Reyes.; Maria T. Herrera-Sepúlveda; Martha Lucia Ospina Martínez; Sheryll Corchuelo
EPI_ISL_3355483, EPI_ISL_3355484, EPI_ISL_3355485, EPI_ISL_3369934	FUNDACIÓN HOSPITAL SAN PEDRO	Corporacion CorpoGen-Universidad de los Andes-Universidad Central	Christian Romero; Jorge Duitama; Juan Manuel Anzola; Laura González; Maryam Chaib De Mares; María Mercedes Zambrano; Nelly Díaz; Patricia Del Portillo; Silvia Restrepo
EPI_ISL_3065505	FUNDACIÓN VALLE DEL LILI	Laboratorio de Biotecnología, Universidad Icesi	Andrés M. Castillo-Giraldo; Beatriz Parra; Diana López-Álvarez; Ludwig Luis Albornoz-Tovar; Marcela Mercado-Reyes; María F. Villegas-Torres; María I. Gutiérrez López; Paola A. Caicedo
EPI_ISL_1494951	Fundacion Cardioinafantil	Instituto Nacional de Salud- Dirección de Investigación en Salud Pública	Carlos Franco-Muñoz; Carmen Osorio; Diana Malo; Diego A. Álvarez-Díaz; Diego Andrés Prada; Gerardo Santamaría; Hector Alejandro Ruiz-Moreno; Jhonnatan Reales-González; Juan Camilo Martinez; Julian Naizaque; Katherine Laiton-Donato; Lisseth Pardo; Magdalena Wiesner; Marcela Mercado-Reyes; Maria T. Herrera-Sepúlveda; Marta Lopez Blanco; Martha Lucia Ospina Martinez; Paola Rojas; Sergio Gomez; Sheryll Corchuelo; Ángela Alarcon Cruz
EPI_ISL_794649, EPI_ISL_794650, EPI_ISL_794651, EPI_ISL_794662	Fundación Cardio Infantil	Instituto Nacional de Salud - Dirección de Investigación en Salud Pública	Carlos Franco-Muñoz; Diego A. Álvarez-Díaz; Diego Andrés Prada; Gerardo Santamaría; Jonathan Reales; Julian Naizaque; Katherine Laiton-Donato; Magdalena Wiesner; Marcela Mercado-Reyes; Maria T. Herrera; Martha Lucia Ospina Martinez; Mauricio Pacheco-Montealegre; Paola Muñoz-Laiton; Sheryl Corchuelo
EPI_ISL_1137616, EPI_ISL_1137617, EPI_ISL_1201897	Fundación Cardio Infantil	Instituto Nacional de Salud- Dirección de Investigación en Salud Pública	Carlos Franco-Muñoz; Diego A. Álvarez-Díaz; Diego Andrés Prada; Gerardo Santamaría; Hector Alejandro Ruiz-Moreno; Jhonnatan Reales-González; Julian Naizaque; Katherine Laiton-Donato; Magdalena Wiesner; Marcela Mercado-Reyes.; Maria T. Herrera-Sepúlveda; Martha Lucia Ospina Martínez; Sheryll Corchuelo
EPI_ISL_794654, EPI_ISL_794655, EPI_ISL_794664, EPI_ISL_794665	Fundación Valle del Lili	Instituto Nacional de Salud - Dirección de Investigación en Salud Pública	Carlos Franco-Muñoz; Diego A. Álvarez-Díaz; Diego Andrés Prada; Gerardo Santamaría; Jonathan Reales; Julian Naizaque; Katherine Laiton-Donato; Magdalena Wiesner; Marcela Mercado-Reyes; Maria T. Herrera; Martha Lucia Ospina Martinez; Mauricio Pacheco-Montealegre; Paola Muñoz-Laiton; Sheryl Corchuelo

EPI_ISL_1626610, EPI_ISL_1626611, EPI_ISL_1626612, EPI_ISL_1626613, EPI_ISL_1626615, EPI_ISL_1626616, EPI_ISL_2157195, EPI_ISL_2157196, EPI_ISL_2157197, EPI_ISL_2157198, EPI_ISL_2157199, EPI_ISL_2157200, EPI_ISL_2157201, EPI_ISL_2157211, EPI_ISL_2157212, EPI_ISL_3491798, EPI_ISL_3491799, EPI_ISL_3491800, EPI_ISL_3491801, EPI_ISL_3491802, EPI_ISL_3491803, EPI_ISL_3491804, EPI_ISL_3491805, EPI_ISL_3491806, EPI_ISL_3491807, EPI_ISL_3491808, EPI_ISL_3491809, EPI_ISL_3491810, EPI_ISL_3491811, EPI_ISL_3491812, EPI_ISL_3491813, EPI_ISL_3491814, EPI_ISL_3491815, EPI_ISL_3491816, EPI_ISL_3491817, EPI_ISL_3491818, EPI_ISL_3491819, EPI_ISL_3491820, EPI_ISL_3491821, EPI_ISL_3491822, EPI_ISL_3491823, EPI_ISL_3491824, EPI_ISL_3491825, EPI_ISL_3491826, EPI_ISL_3491827, EPI_ISL_3491828, EPI_ISL_3491829, EPI_ISL_3491830, EPI_ISL_3491831, EPI_ISL_3491832, EPI_ISL_3491833, EPI_ISL_3491834, EPI_ISL_3491835, EPI_ISL_3673676, EPI_ISL_3673677, EPI_ISL_3673678, EPI_ISL_4196870, EPI_ISL_4196871, EPI_ISL_4196872, EPI_ISL_4196873, EPI_ISL_4196874, EPI_ISL_4196875, EPI_ISL_4196876, EPI_ISL_4196877, EPI_ISL_4196878, EPI_ISL_4196879, EPI_ISL_4198482, EPI_ISL_4198483, EPI_ISL_4198484, EPI_ISL_4198485, EPI_ISL_4198486, EPI_ISL_4198487, EPI_ISL_4198488, EPI_ISL_4198489, EPI_ISL_4198490, EPI_ISL_4198491, EPI_ISL_4198492, EPI_ISL_4198493, EPI_ISL_4198494, EPI_ISL_4198495, EPI_ISL_4198496, EPI_ISL_4198497, EPI_ISL_4198498, EPI_ISL_4198499, EPI_ISL_4198500			
see above	Gencore - Universidad de los Andes	Gencore - Universidad de los Andes	Ana Maria Palacio; Catherine Jaller; Cristian Barrera; David Gonzalez; David González; Erica Salguero; Gabriela Ariza; Luisa Sacristan; Marcela Guevara; Marcela Mercado; Silvia Restrepo
EPI_ISL_3156446, EPI_ISL_3156447, EPI_ISL_3156448, EPI_ISL_3156449, EPI_ISL_3156450, EPI_ISL_3156451, EPI_ISL_3156452, EPI_ISL_3156453			
see above	Gencore-Universidad de los Andes	Laboratorio de biotecnología, Universidad Icesi	Jorge Duitama; Laura N. Gonzalez; Marcela Guevara; Maria F. Villegas; Maria I. Gutierrez; Paola Caicedo; Silvia Restrepo
EPI_ISL_4005445	Grupo Inmunovirología, Universidad de Antioquia	Grupo Inmunovirología, Universidad de Antioquia	Wbeimar Aguilar-Jimenez Geysson J. Fernandez Maria T. Rugeles Francisco J. Diaz
EPI_ISL_447734, EPI_ISL_447735, EPI_ISL_447736, EPI_ISL_447737, EPI_ISL_447738, EPI_ISL_447739, EPI_ISL_447740, EPI_ISL_447741, EPI_ISL_447742, EPI_ISL_447743, EPI_ISL_447744, EPI_ISL_447745, EPI_ISL_447746, EPI_ISL_447747, EPI_ISL_447748, EPI_ISL_447749, EPI_ISL_447750, EPI_ISL_447751, EPI_ISL_447752, EPI_ISL_447753, EPI_ISL_447754			
see above	Grupo de Investigaciones Microbiológicas-UR (GIMUR), Departamento de Biología, Facultad de Ciencias Naturales, Universidad del Rosario, Bogotá, Colombia	Grupo de Investigaciones Microbiológicas-UR (GIMUR), Departamento de Biología, Facultad de Ciencias Naturales, Universidad del Rosario, Bogotá, Colombia Instituto Nacional de Salud, Bogotá, Colombia Icahn School of Medicine at Mount Sinai, New York, USA	Adriana Castillo; Alberto Paniz-Mondolfi; Ana S. Gonzalez-Reiche; Angelica Rico; Aníbal A. Teherán; Carolina Florez; Carolina Hernandez; David Martínez; Emilia Mia Sordillo; Esther C. Barros; Harm van Bakel; Jesús E. Jaimes; Juan David Ramírez; Laura Vega; Lisseth Pardo; Marina Muñoz; Martha L. Ospina; Matthew M. Hernandez; Nathalia Ballesteros; Sergio Castañeda; Sergio Gomez; Viviana Simon
EPI_ISL_1040921, EPI_ISL_1040922, EPI_ISL_1040923, EPI_ISL_1040924, EPI_ISL_1040925, EPI_ISL_1040926, EPI_ISL_1824179, EPI_ISL_1824180, EPI_ISL_1824181, EPI_ISL_1824182, EPI_ISL_1824183, EPI_ISL_1824184, EPI_ISL_1824185, EPI_ISL_1824186, EPI_ISL_1824187, EPI_ISL_1824188, EPI_ISL_2339776, EPI_ISL_2339777, EPI_ISL_2339778, EPI_ISL_2339779, EPI_ISL_2339780, EPI_ISL_2339781, EPI_ISL_2339782, EPI_ISL_2339783, EPI_ISL_2339784, EPI_ISL_2339785, EPI_ISL_2339786, EPI_ISL_2339787, EPI_ISL_2339788, EPI_ISL_2339789, EPI_ISL_2339790, EPI_ISL_2339791, EPI_ISL_2339792, EPI_ISL_2339793, EPI_ISL_2339794, EPI_ISL_2339795, EPI_ISL_2339796, EPI_ISL_2339797, EPI_ISL_2339798, EPI_ISL_2339799, EPI_ISL_2339800, EPI_ISL_2339801, EPI_ISL_2339802, EPI_ISL_2339803, EPI_ISL_2339804, EPI_ISL_2339805, EPI_ISL_2339806, EPI_ISL_2339807, EPI_ISL_2339808, EPI_ISL_2339809, EPI_ISL_2339810, EPI_ISL_2339811, EPI_ISL_2339812, EPI_ISL_2339813, EPI_ISL_2339814, EPI_ISL_2339815, EPI_ISL_2339816, EPI_ISL_2339817, EPI_ISL_2339818, EPI_ISL_2339819, EPI_ISL_2339820, EPI_ISL_2339821, EPI_ISL_2339822, EPI_ISL_2339823, EPI_ISL_2339824, EPI_ISL_2339825, EPI_ISL_2339826, EPI_ISL_2339827, EPI_ISL_2339828, EPI_ISL_2339829, EPI_ISL_2339830, EPI_ISL_2339831, EPI_ISL_2339832, EPI_ISL_2339833, EPI_ISL_2339834, EPI_ISL_2339835, EPI_ISL_2339836, EPI_ISL_2339837, EPI_ISL_2339838, EPI_ISL_2339839, EPI_ISL_2339840, EPI_ISL_2339841, EPI_ISL_2339842, EPI_ISL_2339843, EPI_ISL_2339844, EPI_ISL_2339845, EPI_ISL_2339846, EPI_ISL_2339847, EPI_ISL_2339848, EPI_ISL_2339849, EPI_ISL_2339850, EPI_ISL_2339851, EPI_ISL_2339852, EPI_ISL_2339853, EPI_ISL_2339854, EPI_ISL_2339855, EPI_ISL_2567621, EPI_ISL_2576918, EPI_ISL_2576938, EPI_ISL_2584487, EPI_ISL_2584488, EPI_ISL_2584490, EPI_ISL_2584493, EPI_ISL_2584495, EPI_ISL_2584501, EPI_ISL_2584502, EPI_ISL_2584503, EPI_ISL_2584507, EPI_ISL_2584517, EPI_ISL_2584519, EPI_ISL_2584520, EPI_ISL_2584525, EPI_ISL_2584529, EPI_ISL_2584533, EPI_ISL_2584537, EPI_ISL_2584540, EPI_ISL_2584557, EPI_ISL_2584559, EPI_ISL_2584561, EPI_ISL_2584563, EPI_ISL_2584564, EPI_ISL_2584567, EPI_ISL_2584574, EPI_ISL_2603131, EPI_ISL_2603132, EPI_ISL_2603133, EPI_ISL_2603134, EPI_ISL_2603135, EPI_ISL_2603136, EPI_ISL_2603137, EPI_ISL_2603138, EPI_ISL_2603139, EPI_ISL_2603140, EPI_ISL_2603141, EPI_ISL_2603142, EPI_ISL_2603143, EPI_ISL_2603144, EPI_ISL_2603145, EPI_ISL_2603146, EPI_ISL_2603147, EPI_ISL_2603148, EPI_ISL_2603149, EPI_ISL_2603150, EPI_ISL_2603151, EPI_ISL_2603152, EPI_ISL_2603153, EPI_ISL_2603154, EPI_ISL_2603155, EPI_ISL_2603156, EPI_ISL_2603157, EPI_ISL_2603158, EPI_ISL_2603159, EPI_ISL_2603160, EPI_ISL_2603161, EPI_ISL_2603162, EPI_ISL_2603163, EPI_ISL_2603164, EPI_ISL_2603165, EPI_ISL_2603166, EPI_ISL_2603167, EPI_ISL_2603168, EPI_ISL_2603169, EPI_ISL_2603170, EPI_ISL_2603171, EPI_ISL_2603172, EPI_ISL_2603173, EPI_ISL_2603174, EPI_ISL_2603175, EPI_ISL_2603176, EPI_ISL_2603177, EPI_ISL_2603178, EPI_ISL_2603179, EPI_ISL_2603180, EPI_ISL_2603181, EPI_ISL_2603182, EPI_ISL_2603183			
see above	Grupo de Investigación en Enfermedades Tropicales del Ejército (GINETEJ), Laboratorio de Referencia e Investigación, Dirección de Sanidad Ejército, Bogotá, Colombia	Centro de Investigaciones en Microbiología y Biotecnología-UR (CIMBIUR), Facultad de Ciencias Naturales, Universidad del Rosario, Bogotá, Colombia	Alberto Paniz-Mondolfi; Camilo A. Correa-Cárdenas; Camilo Correa-Cardenas; Carlos A. Rincón; Carolina Oliveros; Claudia Méndez; Claudia P. Mendez; Elizabeth K. Marquez; Elizabeth K. Márquez; Frank de los Santos Ortiz; Juan David Ramírez; Julie J. Perez; Julie Pérez; Lorena Albarracín; Luz H. Patiño; Magda C. Oliveros; Maria C. Duque; Maria Clara Duque; Marina Muñoz; María Teresa Alvarado; Nathalia Ballesteros; Sergio Castañeda; Sergio Gutíerrez-Riveros; Yanira Romero; Zulma Cucunubá
EPI_ISL_1629753, EPI_ISL_1629754, EPI_ISL_1629755, EPI_ISL_1629756, EPI_ISL_1629757, EPI_ISL_1629758, EPI_ISL_1629759			
see above	H.U SAN VICENTE FUNDACION	Universidad Nacional de Colombia - Laboratorio Genómico One Health	Andres F. Cardona-Ríos; Carlos Franco-Muñoz; Daniel O. Maldonado-Perez; Diego A. Álvarez-Díaz; Eliana Patricia Calvo Tapiero; Hector Alejandro Ruiz-Moreno; Idabely Betancur Ortiz; Jorge E. Osorio; Juan P. Hernandez-Ortiz; Karl A Ciuoderis; Laura Silvana Perez; Marcela Mercado-Reyes; Maria Stella López; Sandra Ines Cano; Simón Villegas Velásquez
EPI_ISL_1805648, EPI_ISL_1805649, EPI_ISL_1821073, EPI_ISL_1821074, EPI_ISL_1821075	HIC	Instituto Nacional de Salud- Dirección de Investigación en Salud Pública	Carlos Franco-Muñoz; Carmen Osorio; Diana Malo; Diego A. Álvarez-Díaz; Diego Andrés Prada; Gerardo Santamaría; Hector Alejandro Ruiz-Moreno; Jhonnatan Reales-González; Jorge Rivera; Juan Camilo Martinez; Julian Naizaque; Katherine Laiton-Donato; Lisseth Pardo; Magdalena Wiesner; Marcela Mercado-Reyes; Maria T. Herrera-Sepúlveda; Marta Lopez Blanco; Martha Lucia Ospina Martinez; Paola Rojas; Sergio Gomez; Sheryll Corchuelo; Ángela Alarcon Cruz
EPI_ISL_1805647, EPI_ISL_1820966, EPI_ISL_1821071, EPI_ISL_1821072	HIGUERA ESCALANTE	Instituto Nacional de Salud- Dirección de Investigación en Salud Pública	Carlos Franco-Muñoz; Carmen Osorio; Diana Malo; Diego A. Álvarez-Díaz; Diego Andrés Prada; Gerardo Santamaría; Hector Alejandro Ruiz-Moreno; Jhonnatan Reales-González; Jorge Rivera; Juan Camilo Martinez; Julian Naizaque; Katherine Laiton-Donato; Lisseth Pardo; Magdalena Wiesner; Marcela Mercado-Reyes; Maria T. Herrera-Sepúlveda; Marta Lopez Blanco; Martha Lucia Ospina Martinez; Paola Rojas; Sergio Gomez; Sheryll Corchuelo; Ángela Alarcon Cruz
EPI_ISL_2155046	HOSPITAL CIVIL DE IPIALES	Instituto Nacional de Salud- Dirección de Investigación en Salud Pública	Carlos Franco-Muñoz; Carmen Osorio; Diana Malo; Diego A. Álvarez-Díaz; Diego Andrés Prada; Gerardo Santamaría; Hector Alejandro Ruiz-Moreno; Jhonnatan Reales-González; Jorge Rivera; Juan Camilo Martinez; Julian Naizaque; Katherine Laiton-Donato; Lisseth Pardo; Magdalena Wiesner; Marcela Mercado-Reyes; Maria T. Herrera-Sepúlveda; Marta Lopez Blanco; Martha Lucia Ospina Martinez; Paola Rojas; Sergio Gomez; Sheryll Corchuelo; Ángela Alarcon Cruz
EPI_ISL_2674339	HOSPITAL COMUNAL MALVINAS	Instituto Nacional de Salud- Dirección de Investigación en Salud Pública	Carlos Franco-Muñoz; Carmen Osorio; Diana Malo; Diego A. Álvarez-Díaz; Diego Andrés Prada; Gerardo Santamaría; Hector Alejandro Ruiz-Moreno; Jhonnatan Reales-González; Jorge Rivera; Juan Camilo Martinez; Julian Naizaque; Katherine Laiton-Donato; Lisseth Pardo; Magdalena Wiesner; Marcela Mercado-Reyes; Maria T. Herrera-Sepúlveda; Marta Lopez Blanco; Martha Lucia Ospina Martinez; Paola Rojas; Sergio Gomez; Sheryll Corchuelo; Ángela Alarcon Cruz
EPI_ISL_3459378, EPI_ISL_3459388, EPI_ISL_3459393, EPI_ISL_3459409	HOSPITAL DEPARTAMENTAL DE VILLAVICENCIO	Instituto Nacional de Salud	Carlos Franco-Muñoz; Carmen Osorio; Diana Malo; Diego A. Álvarez-Díaz; Diego Andrés Prada; Gerardo Santamaría; Hector Alejandro Ruiz-Moreno; Jhonnatan Reales-González; Jorge Rivera; Juan Camilo Martinez; Julian Naizaque; Katherine Laiton-Donato; Lisseth Pardo; Magdalena Wiesner; Marcela Mercado-Reyes; Maria T. Herrera-Sepúlveda; Marta Lopez Blanco; Martha Lucia Ospina Martinez; Paola Rojas; Sergio Gomez; Sheryll Corchuelo; Ángela Alarcon Cruz
EPI_ISL_1582982, EPI_ISL_1582983	HOSPITAL DEPARTAMENTAL DE VILLAVICENCIO	Instituto Nacional de Salud- Dirección de Investigación en Salud Pública	Carlos Franco-Muñoz; Carmen Osorio; Diana Malo; Diego A. Álvarez-Díaz; Diego Andrés Prada; Gerardo Santamaría; Hector Alejandro Ruiz-Moreno; Jhonnatan Reales-González; Juan Camilo Martinez; Julian Naizaque; Katherine Laiton-Donato; Lisseth Pardo; Magdalena Wiesner; Marcela Mercado-Reyes; Maria T. Herrera-Sepúlveda; Marta Lopez Blanco; Martha Lucia Ospina Martinez; Paola Rojas; Sergio Gomez; Sheryll Corchuelo; Ángela Alarcon Cruz
EPI_ISL_906539	HOSPITAL DEPARTAMENTAL DE VILLAVICENCIO	Instituto Nacional de Salud- Dirección de Investigación en Salud Pública, Universidad de los Andes- Applied genomics research group, Vicerrectoria de Investigación y Creación, Universidad de los Andes- Systems and Computing Engineering Department	Carlos Franco-Muñoz; Diego A. Álvarez-Díaz; Diego Andrés Prada; Gerardo Santamaría Jorge Duitama; Héctor Alejandro Ruiz-Moreno; Jhonnatan Reales-González; Jorge Ivan Diaz; Julian Naizaque; Katherine Laiton-Donato; Laura Natalia Gonzalez; Magdalena Wiesner; Marcela Mercado-Reyes; Maria T. Herrera-Sepúlveda; Martha Lucia Ospina Martinez; Mauricio Pacheco-Montealegre; Sheryll Corchuelo; Silvia Restrepo-Restrepo
EPI_ISL_906535, EPI_ISL_906537, EPI_ISL_906547	HOSPITAL DEPARTAMENTAL DE VILLAVICENCIO E.S.E.	Instituto Nacional de Salud- Dirección de Investigación en Salud Pública, Universidad de los Andes- Applied genomics research group, Vicerrectoria de Investigación y Creación, Universidad de los Andes- Systems and Computing Engineering Department	Carlos Franco-Muñoz; Diego A. Álvarez-Díaz; Diego Andrés Prada; Gerardo Santamaría Jorge Duitama; Héctor Alejandro Ruiz-Moreno; Jhonnatan Reales-González; Jorge Ivan Diaz; Julian Naizaque; Katherine Laiton-Donato; Laura Natalia Gonzalez; Magdalena Wiesner; Marcela Mercado-Reyes; Maria T. Herrera-Sepúlveda; Martha Lucia Ospina Martinez; Mauricio Pacheco-Montealegre; Sheryll Corchuelo; Silvia Restrepo-Restrepo
EPI_ISL_1821070, EPI_ISL_2500967, EPI_ISL_2500968, EPI_ISL_2500969, EPI_ISL_2674336, EPI_ISL_2674338, EPI_ISL_2674340			
see above	HOSPITAL DEPARTAMENTAL MARIA INMACULADA	Instituto Nacional de Salud- Dirección de Investigación en Salud Pública	Carlos Franco-Muñoz; Carmen Osorio; Diana Malo; Diego A. Álvarez-Díaz; Diego Andrés Prada; Gerardo Santamaría; Hector Alejandro Ruiz-Moreno; Jhonnatan Reales-González; Jorge Rivera; Juan Camilo Martinez; Julian Naizaque; Katherine Laiton-Donato; Lisseth Pardo; Magdalena Wiesner; Marcela Mercado-Reyes; Maria T. Herrera-Sepúlveda; Marta Lopez Blanco; Martha Lucia Ospina Martinez; Paola Rojas; Sergio Gomez; Sheryll Corchuelo; Ángela Alarcon Cruz
EPI_ISL_1494944	HOSPITAL DEPARTAMENTAL SAN VICENTE DE PAUL	Instituto Nacional de Salud- Dirección de Investigación en Salud Pública	Carlos Franco-Muñoz; Carmen Osorio; Diana Malo; Diego A. Álvarez-Díaz; Diego Andrés Prada; Gerardo Santamaría; Hector Alejandro Ruiz-Moreno; Jhonnatan Reales-González; Juan Camilo Martinez; Julian Naizaque; Katherine Laiton-Donato; Lisseth Pardo; Magdalena Wiesner; Marcela Mercado-Reyes; Maria T. Herrera-Sepúlveda; Marta Lopez Blanco; Martha Lucia Ospina Martinez; Paola Rojas; Sergio Gomez; Sheryll Corchuelo; Ángela Alarcon Cruz
EPI_ISL_2362564, EPI_ISL_2362565, EPI_ISL_2362566, EPI_ISL_2362567	HOSPITAL MARIA INMACULADA	Instituto Nacional de Salud- Dirección de Investigación en Salud Pública	Carlos Franco-Muñoz; Carmen Osorio; Diana Malo; Diego A. Álvarez-Díaz; Diego Andrés Prada; Gerardo Santamaría; Hector Alejandro Ruiz-Moreno; Jhonnatan Reales-González; Jorge Rivera; Juan Camilo Martinez; Julian Naizaque; Katherine Laiton-Donato; Lisseth Pardo; Magdalena Wiesner; Marcela Mercado-Reyes; Maria T. Herrera-Sepúlveda; Marta Lopez Blanco; Martha Lucia Ospina Martinez; Paola Rojas; Sergio Gomez; Sheryll Corchuelo; Ángela Alarcon Cruz
EPI_ISL_2674344	HOSPITAL PEDRO LEON ALVAREZ DIAZ	Instituto Nacional de Salud- Dirección de Investigación en Salud Pública	Carlos Franco-Muñoz; Carmen Osorio; Diana Malo; Diego A. Álvarez-Díaz; Diego Andrés Prada; Gerardo Santamaría; Hector Alejandro Ruiz-Moreno; Jhonnatan Reales-González; Jorge Rivera; Juan Camilo Martinez; Julian Naizaque; Katherine Laiton-Donato; Lisseth Pardo; Magdalena Wiesner; Marcela Mercado-Reyes; Maria T. Herrera-Sepúlveda; Marta Lopez Blanco; Martha Lucia Ospina Martinez; Paola Rojas; Sergio Gomez; Sheryll Corchuelo; Ángela Alarcon Cruz
EPI_ISL_2674341	HOSPITAL PROFESOR JORGE CAVELIER	Instituto Nacional de Salud- Dirección de Investigación en Salud Pública	Carlos Franco-Muñoz; Carmen Osorio; Diana Malo; Diego A. Álvarez-Díaz; Diego Andrés Prada; Gerardo Santamaría; Hector Alejandro Ruiz-Moreno; Jhonnatan Reales-González; Jorge Rivera; Juan Camilo Martinez; Julian Naizaque; Katherine Laiton-Donato; Lisseth Pardo; Magdalena Wiesner; Marcela Mercado-Reyes; Maria T. Herrera-Sepúlveda; Marta Lopez Blanco; Martha Lucia Ospina Martinez; Paola Rojas; Sergio Gomez; Sheryll Corchuelo; Ángela Alarcon Cruz
EPI_ISL_2674346, EPI_ISL_2674348	HOSPITAL REGIONAL DE LA ORINOQUIA	Instituto Nacional de Salud- Dirección de Investigación en Salud Pública	Carlos Franco-Muñoz; Carmen Osorio; Diana Malo; Diego A. Álvarez-Díaz; Diego Andrés Prada; Gerardo Santamaría; Hector Alejandro Ruiz-Moreno; Jhonnatan Reales-González; Jorge Rivera; Juan Camilo Martinez; Julian Naizaque; Katherine Laiton-Donato; Lisseth Pardo; Magdalena Wiesner; Marcela Mercado-Reyes; Maria T. Herrera-Sepúlveda; Marta Lopez Blanco; Martha Lucia Ospina Martinez; Paola Rojas; Sergio Gomez; Sheryll Corchuelo; Ángela Alarcon Cruz
EPI_ISL_2009065, EPI_ISL_2158346	HOSPITAL REGIONAL DE ORINOQUIA	Instituto Nacional de Salud- Dirección de Investigación en Salud Pública	Carlos Franco-Muñoz; Carmen Osorio; Diana Malo; Diego A. Álvarez-Díaz; Diego Andrés Prada; Gerardo Santamaría; Hector Alejandro Ruiz-Moreno; Jhonnatan Reales-González; Jorge Rivera; Juan Camilo Martinez; Julian Naizaque; Katherine Laiton-Donato; Lisseth Pardo; Magdalena Wiesner; Marcela Mercado-Reyes; Maria T. Herrera-Sepúlveda; Marta Lopez Blanco; Martha Lucia Ospina Martinez; Paola Rojas; Sergio Gomez; Sheryll Corchuelo; Ángela Alarcon Cruz
EPI_ISL_2834729, EPI_ISL_2834732	HOSPITAL REGIONAL DE ZIPAQUIRA	Instituto Nacional de Salud- Dirección de Investigación en Salud Pública	Carlos Franco-Muñoz; Carmen Osorio; Diana Malo; Diego A. Álvarez-Díaz; Diego Andrés Prada; Gerardo Santamaría; Hector Alejandro Ruiz-Moreno; Jhonnatan Reales-González; Jorge Rivera; Juan Camilo Martinez; Julian Naizaque; Katherine Laiton-Donato; Lisseth Pardo; Magdalena Wiesner; Marcela Mercado-Reyes; Maria T. Herrera-Sepúlveda; Marta Lopez Blanco; Martha Lucia Ospina Martinez; Paola Rojas; Sergio Gomez; Sheryll Corchuelo; Ángela Alarcon Cruz
EPI_ISL_2674347	HOSPITAL REGIONAL ORINOQUIA	Instituto Nacional de Salud- Dirección de Investigación en Salud Pública	Carlos Franco-Muñoz; Carmen Osorio; Diana Malo; Diego A. Álvarez-Díaz; Diego Andrés Prada; Gerardo Santamaría; Hector Alejandro Ruiz-Moreno; Jhonnatan Reales-González; Jorge Rivera; Juan Camilo Martinez; Julian Naizaque; Katherine Laiton-Donato; Lisseth Pardo; Magdalena Wiesner; Marcela Mercado-Reyes; Maria T. Herrera-Sepúlveda; Marta Lopez Blanco; Martha Lucia Ospina Martinez; Paola Rojas; Sergio Gomez; Sheryll Corchuelo; Ángela Alarcon Cruz
EPI_ISL_2674280	HOSPITAL SAN ANTONIO	Instituto Nacional de Salud- Dirección de Investigación en Salud Pública	Carlos Franco-Muñoz; Carmen Osorio; Diana Malo; Diego A. Álvarez-Díaz; Diego Andrés Prada; Gerardo Santamaría; Hector Alejandro Ruiz-Moreno; Jhonnatan Reales-González; Jorge Rivera; Juan Camilo Martinez; Julian Naizaque; Katherine Laiton-Donato; Lisseth Pardo; Magdalena Wiesner; Marcela Mercado-Reyes; Maria T. Herrera-Sepúlveda; Marta Lopez Blanco; Martha Lucia Ospina Martinez; Paola Rojas; Sergio Gomez; Sheryll Corchuelo; Ángela Alarcon Cruz
EPI_ISL_3385772, EPI_ISL_3385782, EPI_ISL_3385814, EPI_ISL_3385846, EPI_ISL_3385847	HOSPITAL SAN JOSE DE MAICAO	Instituto Nacional de Salud- Dirección de Investigación en Salud Pública	Carlos Franco-Muñoz; Carmen Osorio; Diana Malo; Diego A. Álvarez-Díaz; Diego Andrés Prada; Gerardo Santamaría; Hector Alejandro Ruiz-Moreno; Jhonnatan Reales-González; Jorge Rivera; Juan Camilo Martinez; Julian Naizaque; Katherine Laiton-Donato; Lisseth Pardo; Magdalena Wiesner; Marcela Mercado-Reyes; Maria T. Herrera-Sepúlveda; Marta Lopez Blanco; Martha Lucia Ospina Martinez; Paola Rojas; Sergio Gomez; Sheryll Corchuelo; Ángela Alarcon Cruz
EPI_ISL_906541	HOSPITAL SAN JOSE DE MAICAO	Instituto Nacional de Salud- Dirección de Investigación en Salud Pública, Universidad de los Andes- Applied genomics research group, Vicerrectoria de Investigación y Creación, Universidad de los Andes- Systems and Computing Engineering Department	Carlos Franco-Muñoz; Diego A. Álvarez-Díaz; Diego Andrés Prada; Gerardo Santamaría Jorge Duitama; Héctor Alejandro Ruiz-Moreno; Jhonnatan Reales-González; Jorge Ivan Diaz; Julian Naizaque; Katherine Laiton-Donato; Laura Natalia Gonzalez; Magdalena Wiesner; Marcela Mercado-Reyes; Maria T. Herrera-Sepúlveda; Martha Lucia Ospina Martinez; Mauricio Pacheco-Montealegre; Sheryll Corchuelo; Silvia Restrepo-Restrepo
EPI_ISL_1632499, EPI_ISL_3385815	HOSPITAL UNIVERSITARIO DEL VALLE	Instituto Nacional de Salud- Dirección de Investigación en Salud Pública	Carlos Franco-Muñoz; Carmen Osorio; Diana Malo; Diego A. Álvarez-Díaz; Diego Andrés Prada; Gerardo Santamaría; Hector Alejandro Ruiz-Moreno; Jhonnatan Reales-González; Jorge Rivera; Juan Camilo Martinez; Julian Naizaque; Katherine Laiton-Donato; Lisseth Pardo; Magdalena Wiesner; Marcela Mercado-Reyes; Maria T. Herrera-Sepúlveda; Marta Lopez Blanco; Martha Lucia Ospina Martinez; Paola Rojas; Sergio Gomez; Sheryll Corchuelo; Ángela Alarcon Cruz
EPI_ISL_1805634, EPI_ISL_1820963, EPI_ISL_1820964, EPI_ISL_1820965, EPI_ISL_3385829	HOSPITAL UNIVERSITARIO HERMANO MONCALEANO	Instituto Nacional de Salud- Dirección de Investigación en Salud Pública	Carlos Franco-Muñoz; Carmen Osorio; Diana Malo; Diego A. Álvarez-Díaz; Diego Andrés Prada; Gerardo Santamaría; Hector Alejandro Ruiz-Moreno; Jhonnatan Reales-González; Jorge Rivera; Juan Camilo Martinez; Julian Naizaque; Katherine Laiton-Donato; Lisseth Pardo; Magdalena Wiesner; Marcela Mercado-Reyes; Maria T. Herrera-Sepúlveda; Marta Lopez Blanco; Martha Lucia Ospina Martinez; Paola Rojas; Sergio Gomez; Sheryll Corchuelo; Ángela Alarcon Cruz
EPI_ISL_3456029	HOSPITAL UNIVERSITARIO HERNANDO MONCALEANO	Instituto Nacional de Salud	Carlos Franco-Muñoz; Carmen Osorio; Diana Malo; Diego A. Álvarez-Díaz; Diego Andrés Prada; Gerardo Santamaría; Hector Alejandro Ruiz-Moreno; Jhonnatan Reales-González; Jorge Rivera; Juan Camilo

	PERDOMO		Martinez; Julian Naizaque; Katherine Laiton-Donato; Lisseth Pardo; Magdalena Wiesner; Marcela Mercado-Reyes; Maria T. Herrera-Sepúlveda; Marta Lopez Blanco; Martha Lucia Ospina Martinez; Paola Rojas; Sergio Gomez; Sheryll Corchuelo; Ángela Alarcon Cruz
EPI_ISL_3459377, EPI_ISL_3459390, EPI_ISL_3459404, EPI_ISL_3462744	HOSPITAL UNIVERSITARIO HERNANDO MONCALEANO PERDOMO (HUHMP	Instituto Nacional de Salud	Carlos Franco-Muñoz; Carmen Osorio; Diana Malo; Diego A. Álvarez-Díaz; Diego Andrés Prada; Gerardo Santamaría; Hector Alejandro Ruiz-Moreno; Jhonnatan Reales-González; Jorge Rivera; Juan Camilo Martinez; Julian Naizaque; Katherine Laiton-Donato; Lisseth Pardo; Magdalena Wiesner; Marcela Mercado-Reyes; Maria T. Herrera-Sepúlveda; Marta Lopez Blanco; Martha Lucia Ospina Martinez; Paola Rojas; Sergio Gomez; Sheryll Corchuelo; Ángela Alarcon Cruz
EPI_ISL_4300693, EPI_ISL_4300695	HOSPITAL UNIVERSITARIO HERNANDO MONCALEANO PERDOMO (HUHMP)	Instituto Nacional de Salud- Dirección de Investigación en Salud Pública	Carlos Franco-Muñoz; Carmen Osorio; Diana Malo; Diego A. Álvarez-Díaz; Diego Andrés Prada; Gerardo Santamaría; Hector Alejandro Ruiz-Moreno; Jhonnatan Reales-González; Jorge Rivera; Juan Camilo Martinez; Julian Naizaque; Katherine Laiton-Donato; Lisseth Pardo; Magdalena Wiesner; Marcela Mercado-Reyes; Maria T. Herrera-Sepúlveda; Marta Lopez Blanco; Martha Lucia Ospina Martinez; Paola Rojas; Sergio Gomez; Sheryll Corchuelo; Ángela Alarcon Cruz
EPI_ISL_794659, EPI_ISL_794660	HOSPITAL UNIVERSITARIO SAN IGNACIO	Instituto Nacional de Salud - Dirección de Investigación en Salud Pública	Carlos Franco-Muñoz; Diego A. Álvarez-Díaz; Diego Andrés Prada; Gerardo Santamaría; Jonathan Reales; Julian Naizaque; Katherine Laiton-Donato; Magdalena Wiesner; Marcela Mercado-Reyes; Maria T. Herrera; Martha Lucia Ospina Martinez; Mauricio Pacheco-Montealegre; Paola Muñoz-Laiton; Sheryl Corchuelo
EPI_ISL_1960043, EPI_ISL_1960044, EPI_ISL_1960062, EPI_ISL_1960073, EPI_ISL_2437999, EPI_ISL_2438000, EPI_ISL_2438001, EPI_ISL_2438002, EPI_ISL_2621174, EPI_ISL_2621175, EPI_ISL_2621176, EPI_ISL_2621859, EPI_ISL_2651225, EPI_ISL_2651226, EPI_ISL_3276546, EPI_ISL_3276551, EPI_ISL_3276552, EPI_ISL_3276553, EPI_ISL_3276554			
see above	HPTU	Universidad Nacional de Colombia - Laboratorio Genómico One Health	Andres F. Cardona-Rios; Carlos Franco-Muñoz; Carolina Muñoz-Arango; Celeny Ortiz; Daniel O. Maldonado-Perez; Diego A. Álvarez-Díaz; Hector Alejandro Ruiz-Moreno; Idabely Betancur Ortiz; Jorge E. Osorio; Juan P. Hernandez-Ortiz; Karl A Ciuoderis; Katherine Laiton-Donato; Laura Silvana Perez; Lina M. Hurtado; Marcela Mercado-Reyes; Maria Angélica Maya; Maria Stella López; Rita Almanza Payares; Sandra Ines Cano; Simón Villegas Velásquez
EPI_ISL_3320721, EPI_ISL_3320726, EPI_ISL_3320727, EPI_ISL_3320732, EPI_ISL_3320753	Hemato Oncologos	Instituto Nacional de Salud	Carlos Franco-Muñoz; Carmen Osorio; Diana Malo; Diego A. Álvarez-Díaz; Diego Andrés Prada; Gerardo Santamaría; Hector Alejandro Ruiz-Moreno; Jhonnatan Reales-González; Jorge Rivera; Juan Camilo Martinez; Julian Naizaque; Katherine Laiton-Donato; Lisseth Pardo; Magdalena Wiesner; Marcela Mercado-Reyes; Maria T. Herrera-Sepúlveda; Marta Lopez Blanco; Martha Lucia Ospina Martinez; Paola Rojas; Sergio Gomez; Sheryll Corchuelo; Ángela Alarcon Cruz
EPI_ISL_1017712	Hemato Oncologos S.A	Instituto Nacional de Salud- Dirección de Investigación en Salud Pública	Carlos Franco-Muñoz; Diego A. Álvarez-Díaz; Diego Andrés Prada; Gerardo Santamaría; Hector Alejandro Ruiz-Moreno; Jhonnatan Reales-González; Julian Naizaque; Katherine Laiton-Donato; Magdalena Wiesner; Marcela Mercado-Reyes; Maria T. Herrera-Sepúlveda; Martha Lucia Ospina Martinez; Sheryll Corchuelo
EPI_ISL_3391982	Hospital Regional de Zipaquira	Instituto Nacional de Salud- Dirección de Investigación en Salud Pública	Carlos Franco-Muñoz; Carmen Osorio; Diana Malo; Diego A. Álvarez-Díaz; Diego Andrés Prada; Gerardo Santamaría; Hector Alejandro Ruiz-Moreno; Jhonnatan Reales-González; Jorge Rivera; Juan Camilo Martinez; Julian Naizaque; Katherine Laiton-Donato; Lisseth Pardo; Magdalena Wiesner; Marcela Mercado-Reyes; Maria T. Herrera-Sepúlveda; Marta Lopez Blanco; Martha Lucia Ospina Martinez; Paola Rojas; Sergio Gomez; Sheryll Corchuelo; Ángela Alarcon Cruz
EPI_ISL_1091919, EPI_ISL_1091920, EPI_ISL_1091921	Hospital Departamental María Inmaculada	Instituto Nacional de Salud- Dirección de Investigación en Salud Pública	Carlos Franco-Muñoz; Diego A. Álvarez-Díaz; Diego Andrés Prada; Gerardo Santamaría; Hector Alejandro Ruiz-Moreno; Jhonnatan Reales-González; Julian Naizaque; Katherine Laiton-Donato; Magdalena Wiesner; Marcela Mercado-Reyes; Maria T. Herrera-Sepúlveda; Martha Lucia Ospina Martinez; Sheryll Corchuelo
EPI_ISL_3671000, EPI_ISL_3671012, EPI_ISL_3671013, EPI_ISL_3671057	Hospital Fundacion San Vicente de Paul	Universidad Nacional de Colombia - Laboratorio Genómico One Health	Andres F. Cardona-Rios; Carlos Franco-Muñoz; Carolina Muñoz-Arango; Celeny Ortiz; Daniel O. Maldonado-Perez; Diego A. Álvarez-Díaz; Hector Alejandro Ruiz-Moreno; Idabely Betancur Ortiz; Jorge E. Osorio; Juan P. Hernandez-Ortiz; Karl A Ciuoderis; Katherine Laiton-Donato; Laura Silvana Perez; Lina M. Hurtado; Marcela Mercado-Reyes; Maria Angélica Maya; Maria Stella López; Rita Almanza Payares; Sandra Ines Cano; Simón Villegas Velásquez
EPI_ISL_3332697	Hospital Pablo Tobon	Corporación para Investigaciones Biológicas-CIB	Jeanneth Mosquera Rendon; Jenny Santiago Cuesta; Marcela Mercado Reyes; Uriel A. Hurtado Paez
EPI_ISL_3671058, EPI_ISL_3671065, EPI_ISL_3721574, EPI_ISL_3721593, EPI_ISL_3721595, EPI_ISL_3721602, EPI_ISL_3721618			
see above	Hospital Pablo Tobon	Universidad Nacional de Colombia - Laboratorio Genómico One Health	Andres F. Cardona-Rios; Carlos Franco-Muñoz; Carolina Muñoz-Arango; Celeny Ortiz; Daniel O. Maldonado-Perez; Diego A. Álvarez-Díaz; Hector Alejandro Ruiz-Moreno; Idabely Betancur Ortiz; Jorge E. Osorio; Juan P. Hernandez-Ortiz; Karl A Ciuoderis; Katherine Laiton-Donato; Laura Silvana Perez; Lina M. Hurtado; Marcela Mercado-Reyes; Maria Angélica Maya; Maria Stella López; Rita Almanza Payares; Sandra Ines Cano; Simón Villegas Velásquez
EPI_ISL_1015358, EPI_ISL_1015359, EPI_ISL_1015360, EPI_ISL_1629716, EPI_ISL_1675294, EPI_ISL_1675295, EPI_ISL_1675296, EPI_ISL_1675297, EPI_ISL_1675298, EPI_ISL_1675326, EPI_ISL_1675327, EPI_ISL_1675328, EPI_ISL_1675329, EPI_ISL_1675330			
see above	Hospital Pablo Tobón Uribe	Universidad Nacional de Colombia - Laboratorio Genómico One Health	Andres F. Cardona-Rios; Carlos Franco-Muñoz; Daniel O. Maldonado-Perez; Diego A. Álvarez-Díaz; Eliana Patricia Calvo Tapiero; Hector Alejandro Ruiz-Moreno; Idabely Betancur Ortiz; Jorge E. Osorio; Juan P. Hernandez-Ortiz; Karl A Ciuoderis; Katherine Laiton-Donato; Laura Silvana Perez; Lina M. Hurtado; Marcela Mercado-Reyes; Maria Angélica Maya; Maria Stella López; Rita Almanza Payares; Sandra Ines Cano; Simón Villegas Velásquez
EPI_ISL_3320718, EPI_ISL_3320723	Hospital Regional de la Orinoquia -HORO	Instituto Nacional de Salud	Carlos Franco-Muñoz; Carmen Osorio; Diana Malo; Diego A. Álvarez-Díaz; Diego Andrés Prada; Gerardo Santamaría; Hector Alejandro Ruiz-Moreno; Jhonnatan Reales-González; Jorge Rivera; Juan Camilo Martinez; Julian Naizaque; Katherine Laiton-Donato; Lisseth Pardo; Magdalena Wiesner; Marcela Mercado-Reyes; Maria T. Herrera-Sepúlveda; Marta Lopez Blanco; Martha Lucia Ospina Martinez; Paola Rojas; Sergio Gomez; Sheryll Corchuelo; Ángela Alarcon Cruz
EPI_ISL_2674328	Hospital Regional de Zipaquira	Instituto Nacional de Salud- Dirección de Investigación en Salud Pública	Carlos Franco-Muñoz; Carmen Osorio; Diana Malo; Diego A. Álvarez-Díaz; Diego Andrés Prada; Gerardo Santamaría; Hector Alejandro Ruiz-Moreno; Jhonnatan Reales-González; Jorge Rivera; Juan Camilo Martinez; Julian Naizaque; Katherine Laiton-Donato; Lisseth Pardo; Magdalena Wiesner; Marcela Mercado-Reyes; Maria T. Herrera-Sepúlveda; Marta Lopez Blanco; Martha Lucia Ospina Martinez; Paola Rojas; Sergio Gomez; Sheryll Corchuelo; Ángela Alarcon Cruz
EPI_ISL_906532	Hospital San Jose de Maicao	Instituto Nacional de Salud- Dirección de Investigación en Salud Pública, Universidad de los Andes- Applied genomics research group, Vicerrectoria de Investigación y Creación, Universidad de los Andes- Systems and Computing Engineering Department	Carlos Franco-Muñoz; Diego A. Álvarez-Díaz; Diego Andrés Prada; Gerardo Santamaría Jorge Duitama; Héctor Alejandro Ruiz-Moreno; Jhonnatan Reales-González; Jorge Ivan Diaz; Julian Naizaque; Katherine Laiton-Donato; Laura Natalia Gonzalez; Magdalena Wiesner; Marcela Mercado-Reyes; Maria T. Herrera-Sepúlveda; Martha Lucia Ospina Martinez; Mauricio Pacheco-Montealegre; Sheryll Corchuelo; Silvia Restrepo-Restrepo
EPI_ISL_1017706, EPI_ISL_1017707, EPI_ISL_1017708, EPI_ISL_1017709, EPI_ISL_1017710	Hospital Universitario Hernando Moncaleano Perdomo	Instituto Nacional de Salud- Dirección de Investigación en Salud Pública	Carlos Franco-Muñoz; Diego A. Álvarez-Díaz; Diego Andrés Prada; Gerardo Santamaría; Hector Alejandro Ruiz-Moreno; Jhonnatan Reales-González; Julian Naizaque; Katherine Laiton-Donato; Magdalena Wiesner; Marcela Mercado-Reyes; Maria T. Herrera-Sepúlveda; Martha Lucia Ospina Martinez; Sheryll Corchuelo
EPI_ISL_1220047	Hospital Universitario de San Rafael de Tunja	Instituto Nacional de Salud- Dirección de Investigación en Salud Pública	Carlos Franco-Muñoz; Diego A. Álvarez-Díaz; Diego Andrés Prada; Gerardo Santamaría; Hector Alejandro Ruiz-Moreno; Jhonnatan Reales-González; Julian Naizaque; Katherine Laiton-Donato; Magdalena Wiesner; Marcela Mercado-Reyes.; Maria T. Herrera-Sepúlveda; Martha Lucia Ospina Martínez; Sheryll Corchuelo
EPI_ISL_4419183	Hospital Universitario del Valle- Evaristo Garcia	Universidad del Valle	Programa Nacional de Caracterización Genómica de SARS-CoV-2
EPI_ISL_1091779, EPI_ISL_1091780, EPI_ISL_1091781, EPI_ISL_1629717, EPI_ISL_1629718, EPI_ISL_1629719, EPI_ISL_1629720, EPI_ISL_1675293, EPI_ISL_1675313, EPI_ISL_1675314, EPI_ISL_1675331, EPI_ISL_1824700, EPI_ISL_1824702, EPI_ISL_1824703, EPI_ISL_1824709, EPI_ISL_1824710, EPI_ISL_2321573, EPI_ISL_2321574, EPI_ISL_2321575			
see above	ICMT	Universidad Nacional de Colombia - Laboratorio Genómico One Health	Andres F. Cardona-Rios; Carlos Franco-Muñoz; Carolina Muñoz-Arango; Celeny Ortiz; Daniel O. Maldonado-Perez; Diego A. Álvarez-Díaz; Eliana Patricia Calvo Tapiero; Hector Alejandro Ruiz-Moreno; Idabely Betancur Ortiz; Jorge E. Osorio; Juan P. Hernandez-Ortiz; Karl A Ciuoderis; Katherine Laiton-Donato; Laura Silvana Perez; Lina M. Hurtado; Marcela Mercado-Reyes; Maria Angélica Maya; Maria Stella López; Rita Almanza Payares; Sandra Ines Cano; Simón Villegas Velásquez
EPI_ISL_1960050, EPI_ISL_1960054, EPI_ISL_1960074, EPI_ISL_1960079, EPI_ISL_2136274, EPI_ISL_2321589, EPI_ISL_2321590, EPI_ISL_2321591, EPI_ISL_2621161, EPI_ISL_2621162, EPI_ISL_2621163, EPI_ISL_2621164, EPI_ISL_2621165, EPI_ISL_2621166, EPI_ISL_2621167, EPI_ISL_2621168, EPI_ISL_2621169, EPI_ISL_2621170, EPI_ISL_2621171, EPI_ISL_2621172, EPI_ISL_2621173, EPI_ISL_2621884, EPI_ISL_2651215, EPI_ISL_2651216, EPI_ISL_2651217, EPI_ISL_2651218, EPI_ISL_2651219, EPI_ISL_2651220, EPI_ISL_2651221, EPI_ISL_2651222, EPI_ISL_2651223			
see above	ICMT-APARTADO	Universidad Nacional de Colombia - Laboratorio Genómico One Health	Andres F. Cardona-Rios; Carlos Franco-Muñoz; Carolina Muñoz-Arango; Celeny Ortiz; Daniel O. Maldonado-Perez; Diego A. Álvarez-Díaz; Hector Alejandro Ruiz-Moreno; Idabely Betancur Ortiz; Jorge E. Osorio; Juan P. Hernandez-Ortiz; Karl A Ciuoderis; Katherine Laiton-Donato; Laura Silvana Perez; Lina M. Hurtado; Marcela Mercado-Reyes; Maria Angélica Maya; Maria Stella López; Rita Almanza Payares; Sandra Ines Cano; Simón Villegas Velásquez
EPI_ISL_3276628, EPI_ISL_3276629, EPI_ISL_3276630, EPI_ISL_3276631, EPI_ISL_3276632, EPI_ISL_3276633, EPI_ISL_3276634, EPI_ISL_3276635, EPI_ISL_3276636, EPI_ISL_3276637, EPI_ISL_3276638, EPI_ISL_3276639, EPI_ISL_3276640, EPI_ISL_3276641, EPI_ISL_3276642, EPI_ISL_3721578, EPI_ISL_3721579, EPI_ISL_3721580, EPI_ISL_3721594, EPI_ISL_3721606, EPI_ISL_3721609, EPI_ISL_3721616, EPI_ISL_3721624			
see above	ICMT-Apartado	Universidad Nacional de Colombia - Laboratorio Genómico One Health	Andres F. Cardona-Rios; Carlos Franco-Muñoz; Carolina Muñoz-Arango; Celeny Ortiz; Daniel O. Maldonado-Perez; Diego A. Álvarez-Díaz; Hector Alejandro Ruiz-Moreno; Idabely Betancur Ortiz; Jorge E. Osorio; Juan P. Hernandez-Ortiz; Karl A Ciuoderis; Katherine Laiton-Donato; Laura Silvana Perez; Lina M. Hurtado; Marcela Mercado-Reyes; Maria Angélica Maya; Maria Stella López; Rita Almanza Payares; Sandra Ines Cano; Simón Villegas Velásquez
EPI_ISL_2621157, EPI_ISL_2621158, EPI_ISL_2621159, EPI_ISL_2621160, EPI_ISL_2651224	ICMT-SABANETA	Universidad Nacional de Colombia - Laboratorio Genómico One Health	Andres F. Cardona-Rios; Carlos Franco-Muñoz; Carolina Muñoz-Arango; Celeny Ortiz; Daniel O. Maldonado-Perez; Diego A. Álvarez-Díaz; Hector Alejandro Ruiz-Moreno; Idabely Betancur Ortiz; Jorge E. Osorio; Juan P. Hernandez-Ortiz; Karl A Ciuoderis; Katherine Laiton-Donato; Laura Silvana Perez; Lina M. Hurtado; Marcela Mercado-Reyes; Maria Angélica Maya; Maria Stella López; Rita Almanza Payares; Sandra Ines Cano; Simón Villegas Velásquez
EPI_ISL_4198462, EPI_ISL_4198463, EPI_ISL_4198464, EPI_ISL_4198465, EPI_ISL_4198466, EPI_ISL_4198467, EPI_ISL_4198468, EPI_ISL_4198469, EPI_ISL_4198470, EPI_ISL_4198471, EPI_ISL_4198472, EPI_ISL_4198473, EPI_ISL_4198474, EPI_ISL_4198475, EPI_ISL_4198476, EPI_ISL_4198477, EPI_ISL_4198478, EPI_ISL_4198479, EPI_ISL_4198480, EPI_ISL_4198481			
see above	IDIME	Gencore - Universidad de los Andes	Cristian Barrera; David González; Gabriela Ariza; Luisa Sacristan; Marcela Guevara; Marcela Mercado; Silvia Restrepo
EPI_ISL_845630, EPI_ISL_845631, EPI_ISL_845632, EPI_ISL_845633	IDIME S.A	Instituto Nacional de Salud - Dirección de Investigación en Salud Pública	Carlos Franco-Muñoz; Diego A. Álvarez-Díaz; Diego Andrés Prada; Gerardo Santamaría; Jonathan Reales; Julian Naizaque; Katherine Laiton-Donato; Magdalena Wiesner; Marcela Mercado-Reyes; Maria T. Herrera-Sepúlveda; Martha Lucia Ospina Martinez; Mauricio Pacheco-Montealegre; Paola Muñoz-Laiton; Sheryll Corchuelo
EPI_ISL_3459395, EPI_ISL_3459416, EPI_ISL_3459417	IDIME-CLINICA NUEVA DE CALI	Instituto Nacional de Salud	Carlos Franco-Muñoz; Carmen Osorio; Diana Malo; Diego A. Álvarez-Díaz; Diego Andrés Prada; Gerardo Santamaría; Hector Alejandro Ruiz-Moreno; Jhonnatan Reales-González; Jorge Rivera; Juan Camilo Martinez; Julian Naizaque; Katherine Laiton-Donato; Lisseth Pardo; Magdalena Wiesner; Marcela Mercado-Reyes; Maria T. Herrera-Sepúlveda; Marta Lopez Blanco; Martha Lucia Ospina Martinez; Paola Rojas; Sergio Gomez; Sheryll Corchuelo; Ángela Alarcon Cruz
EPI_ISL_1820961, EPI_ISL_2155041	IMAT	Instituto Nacional de Salud- Dirección de Investigación en Salud Pública	Carlos Franco-Muñoz; Carmen Osorio; Diana Malo; Diego A. Álvarez-Díaz; Diego Andrés Prada; Gerardo Santamaría; Hector Alejandro Ruiz-Moreno; Jhonnatan Reales-González; Jorge Rivera; Juan Camilo Martinez; Julian Naizaque; Katherine Laiton-Donato; Lisseth Pardo; Magdalena Wiesner; Marcela Mercado-Reyes; Maria T. Herrera-Sepúlveda; Marta Lopez Blanco; Martha Lucia Ospina Martinez; Paola Rojas; Sergio Gomez; Sheryll Corchuelo; Ángela Alarcon Cruz
EPI_ISL_2827770, EPI_ISL_2827772, EPI_ISL_2827774, EPI_ISL_2827780, EPI_ISL_2827784, EPI_ISL_3391978, EPI_ISL_3391981, EPI_ISL_3391984, EPI_ISL_3391985, EPI_ISL_3391986			
see above	IMAT S.A.S.	Instituto Nacional de Salud- Dirección de Investigación en Salud Pública	Carlos Franco-Muñoz; Carmen Osorio; Diana Malo; Diego A. Álvarez-Díaz; Diego Andrés Prada; Gerardo Santamaría; Hector Alejandro Ruiz-Moreno; Jhonnatan Reales-González; Jorge Rivera; Juan Camilo Martinez; Julian Naizaque; Katherine Laiton-Donato; Lisseth Pardo; Magdalena Wiesner; Marcela Mercado-Reyes; Maria T. Herrera-Sepúlveda; Marta Lopez Blanco; Martha Lucia Ospina Martinez; Paola Rojas; Sergio Gomez; Sheryll Corchuelo; Ángela Alarcon Cruz
EPI_ISL_3459414	INS	Instituto Nacional de Salud	Carlos Franco-Muñoz; Carmen Osorio; Diana Malo; Diego A. Álvarez-Díaz; Diego Andrés Prada; Gerardo Santamaría; Hector Alejandro Ruiz-Moreno; Jhonnatan Reales-González; Jorge Rivera; Juan Camilo Martinez; Julian Naizaque; Katherine Laiton-Donato; Lisseth Pardo; Magdalena Wiesner; Marcela Mercado-Reyes; Maria T. Herrera-Sepúlveda; Marta Lopez Blanco; Martha Lucia Ospina Martinez; Paola Rojas; Sergio Gomez; Sheryll Corchuelo; Ángela Alarcon Cruz
EPI_ISL_2674342, EPI_ISL_2674343, EPI_ISL_2674345	IPS YENNY ZORAYA SALAZAR	Instituto Nacional de Salud- Dirección de Investigación en Salud Pública	Carlos Franco-Muñoz; Carmen Osorio; Diana Malo; Diego A. Álvarez-Díaz; Diego Andrés Prada; Gerardo Santamaría; Hector Alejandro Ruiz-Moreno; Jhonnatan Reales-González; Jorge Rivera; Juan Camilo Martinez; Julian Naizaque; Katherine Laiton-Donato; Lisseth Pardo; Magdalena Wiesner; Marcela Mercado-Reyes; Maria T. Herrera-Sepúlveda; Marta Lopez Blanco; Martha Lucia Ospina Martinez; Paola Rojas; Sergio Gomez; Sheryll Corchuelo; Ángela Alarcon Cruz
EPI_ISL_3721577, EPI_ISL_3721585, EPI_ISL_3721587, EPI_ISL_3721600, EPI_ISL_3721608, EPI_ISL_3721610, EPI_ISL_3721614, EPI_ISL_3721619, EPI_ISL_3721620			
see above	Instituto Colombiano Medicina Tropical ICMT - Sede Sabaneta	Universidad Nacional de Colombia - Laboratorio Genómico One Health	Andres F. Cardona-Rios; Carlos Franco-Muñoz; Carolina Muñoz-Arango; Celeny Ortiz; Daniel O. Maldonado-Perez; Diego A. Álvarez-Díaz; Hector Alejandro Ruiz-Moreno; Idabely Betancur Ortiz; Jorge E. Osorio; Juan P. Hernandez-Ortiz; Karl A Ciuoderis; Katherine Laiton-Donato; Laura Silvana Perez; Lina M. Hurtado; Marcela Mercado-Reyes; Maria Angélica Maya; Maria Stella López; Rita Almanza Payares; Sandra Ines Cano; Simón Villegas Velásquez

EPI_ISL_3276643	Instituto Colombiano de Medicina Tropical - Apartadó	Universidad Nacional de Colombia - Laboratorio Genómico One Health	Andres F. Cardona-Rios; Carlos Franco-Muñoz; Carolina Muñoz-Arango; Celeny Ortiz; Daniel O. Maldonado-Perez; Diego A. Álvarez-Díaz; Hector Alejandro Ruiz-Moreno; Idabely Betancur Ortiz; Jorge E. Osorio; Juan P. Hernandez-Ortiz; Karl A Ciuoderis; Katherine Laiton-Donato; Laura Silvana Perez; Lina M. Hurtado; Marcela Mercado-Reyes; Maria Angélica Maya; Maria Stella López; Rita Almanza Payares; Sandra Ines Cano; Simón Villegas Velásquez
EPI_ISL_1015735	Instituto Nacional de Cancerologia	Instituto Nacional de Salud	Carlos Franco-Muñoz; Diego A. Álvarez-Díaz; Diego Andrés Prada; Gerardo Santamaría; Hector Alejandro Ruiz-Moreno; Jhonnatan Reales-González; Julian Naizaque; Katherine Laiton-Donato; Magdalena Wiesner; Marcela Mercado-Reyes; Maria T. Herrera-Sepúlveda; Martha Lucia Ospina Martinez; Sheryll Corchuelo
EPI_ISL_845634	Instituto Nacional de Cancerologia	Instituto Nacional de Salud - Dirección de Investigación en Salud Pública	Carlos Franco-Muñoz; Diego A. Álvarez-Díaz; Diego Andrés Prada; Gerardo Santamaría; Jonathan Reales; Julian Naizaque; Katherine Laiton-Donato; Magdalena Wiesner; Marcela Mercado-Reyes; Maria T. Herrera-Sepúlveda; Martha Lucia Ospina Martinez; Mauricio Pacheco-Montealegre; Paola Muñoz-Laiton; Sheryll Corchuelo
EPI_ISL_1557223	Instituto Nacional de Salud	Corporacion Corpogen Universidad de los Andes Universidad Central	Anzola; Chaib De Mares; Christian; Del Portillo; Duitama; Díaz; González; Jorge; Juan Manuel; Laura; Maryam; María Mercedes; Nelly; Patricia; Restrepo; Romero; Silvia; Zambrano
EPI_ISL_3319858, EPI_ISL_3320717, EPI_ISL_3320722	Instituto Nacional de Salud	Instituto Nacional de Salud	Carlos Franco-Muñoz; Carmen Osorio; Diana Malo; Diego A. Álvarez-Díaz; Diego Andrés Prada; Gerardo Santamaría; Hector Alejandro Ruiz-Moreno; Jhonnatan Reales-González; Jorge Rivera; Juan Camilo Martinez; Julian Naizaque; Katherine Laiton-Donato; Lisseth Pardo; Magdalena Wiesner; Marcela Mercado-Reyes; Maria T. Herrera-Sepúlveda; Marta Lopez Blanco; Martha Lucia Ospina Martinez; Paola Rojas; Sergio Gomez; Sheryll Corchuelo; Ángela Alarcon Cruz
EPI_ISL_418262	Instituto Nacional de Salud	Instituto Nacional de Salud Universidad Cooperativa de Colombia Instituto Alexander von Humboldt Imperial College-London London School of Hygiene & Tropical Medicine	Astrid C. Flórez; Carlos Franco-Muñoz; Christian Julian Villabona-Arenas; Diana Marcela Walteros-Acero; Diego A. Álvarez-Díaz; Erika Ospitia; Gloria Puerto; Jose A. Usme-Ciro; Juliana Barbosa; Katherine Laiton-Donato; Liz Villabona-Arenas; Luz Dary Rodriguez; Mailyn A.Gonzalez; Marcela Mercado-Reyes; Martha Lucia Ospina Martinez; Nicolas D. Franco-Sierra; Nuno Rodrigues Faria; Sergio Gomez Rangel; Sussy Echeverria; Zulma M. Cucunubá
EPI_ISL_2155036, EPI_ISL_2362572, EPI_ISL_2500925, EPI_ISL_2500926, EPI_ISL_2500927, EPI_ISL_2500928, EPI_ISL_2500929, EPI_ISL_2500930, EPI_ISL_2500931, EPI_ISL_2500932, EPI_ISL_2500933, EPI_ISL_2500934, EPI_ISL_2500935, EPI_ISL_2500936, EPI_ISL_2500937, EPI_ISL_2500938, EPI_ISL_2500939, EPI_ISL_2500940, EPI_ISL_2500941, EPI_ISL_2500942, EPI_ISL_2500943, EPI_ISL_2500944, EPI_ISL_2500945, EPI_ISL_2500946, EPI_ISL_2500947, EPI_ISL_2500948, EPI_ISL_2500949, EPI_ISL_2500950, EPI_ISL_2500951, EPI_ISL_2500952, EPI_ISL_2500953, EPI_ISL_2500954, EPI_ISL_2500955, EPI_ISL_2500956, EPI_ISL_2500957, EPI_ISL_2500958, EPI_ISL_2500959, EPI_ISL_2500960, EPI_ISL_2500961, EPI_ISL_2500962, EPI_ISL_2500963, EPI_ISL_2500964, EPI_ISL_2500965, EPI_ISL_2500966, EPI_ISL_2500970, EPI_ISL_2500971, EPI_ISL_2609609, EPI_ISL_2827760, EPI_ISL_2827762, EPI_ISL_2827775, EPI_ISL_2827781, EPI_ISL_2827785			
see above	Instituto Nacional de Salud	Instituto Nacional de Salud- Dirección de Investigación en Salud Pública	Carlos Franco-Muñoz; Carmen Osorio; Diana Malo; Diego A. Álvarez-Díaz; Diego Andrés Prada; Gerardo Santamaría; Hector Alejandro Ruiz-Moreno; Jhonnatan Reales-González; Jorge Rivera; Juan Camilo Martinez; Julian Naizaque; Katherine Laiton-Donato; Lisseth Pardo; Magdalena Wiesner; Marcela Mercado-Reyes; Maria T. Herrera-Sepúlveda; Marta Lopez Blanco; Martha Lucia Ospina Martinez; Paola Rojas; Sergio Gomez; Sheryll Corchuelo; Ángela Alarcon Cruz
EPI_ISL_791084, EPI_ISL_791085, EPI_ISL_791086, EPI_ISL_791087, EPI_ISL_791088, EPI_ISL_791089, EPI_ISL_791090, EPI_ISL_791091, EPI_ISL_791092, EPI_ISL_791093, EPI_ISL_791094, EPI_ISL_791095, EPI_ISL_791096			
see above	Instituto Nacional de Salud - Unidad de Secuenciación y Análisis Genómico	Instituto Nacional de Salud - Dirección de Investigación en Salud Pública	Carlos Franco-Muñoz; Diego A. Álvarez-Díaz; Diego Andrés Prada; Gerardo Santamaría; Jeadran Malagon-Rojas; Jesith Toloza; Jonathan Reales; Julia Almentero; Julian Naizaque; Katherine Laiton-Donato; Magdalena Wiesner; Marcela Mercado-Reyes; Maria T. Herrera; Martha Lucia Ospina Martinez; Mauricio Pacheco-Montealegre; Paola Muñoz-Laiton; Ronald Lopez; Sheryl Corchuelo
EPI_ISL_456116, EPI_ISL_456117, EPI_ISL_456118, EPI_ISL_456119, EPI_ISL_456120, EPI_ISL_456121, EPI_ISL_456122, EPI_ISL_456123, EPI_ISL_456124, EPI_ISL_456125, EPI_ISL_456126, EPI_ISL_456127, EPI_ISL_456128, EPI_ISL_456129, EPI_ISL_456130, EPI_ISL_456131, EPI_ISL_456132, EPI_ISL_456133, EPI_ISL_456134, EPI_ISL_456135, EPI_ISL_456136, EPI_ISL_456137, EPI_ISL_456138, EPI_ISL_456139, EPI_ISL_456140, EPI_ISL_456141, EPI_ISL_456142, EPI_ISL_456143, EPI_ISL_456144, EPI_ISL_456145, EPI_ISL_456146, EPI_ISL_456147, EPI_ISL_456148, EPI_ISL_456149, EPI_ISL_456150, EPI_ISL_456151, EPI_ISL_456152, EPI_ISL_456153, EPI_ISL_456154, EPI_ISL_456155, EPI_ISL_456156			
see above	Instituto Nacional de Salud - Unidad de Secuenciación y Análisis Genómico	Instituto Nacional de Salud, Universidad Cooperativa de Colombia, Instituto Alexander von Humboldt, Imperial College-London, London School of Hygiene & Tropical Medicine	Astrid C. Flórez; Carlos Franco-Muñoz; Christian Julian Villabona-Arenas; Diana Marcela Walteros-Acero; Diego A. Álvarez-Díaz; Erika Ospitia; Gloria Puerto; Jose A. Usme-Ciro; Juliana Barbosa; Katherine Laiton-Donato; Liz Villabona-Arenas; Luz Dary Rodriguez; Mailyn A.Gonzalez; Marcela Mercado-Reyes.; Martha Lucia Ospina Martinez; Nicolas D. Franco-Sierra; Sergio Gomez-Rangel; Sussy Echeverria; Zulma M. Cucunubá
EPI_ISL_941942, EPI_ISL_941943, EPI_ISL_941949, EPI_ISL_941951, EPI_ISL_941953, EPI_ISL_941955, EPI_ISL_941956, EPI_ISL_941957, EPI_ISL_941958, EPI_ISL_941960, EPI_ISL_941963, EPI_ISL_941984, EPI_ISL_941985, EPI_ISL_941986, EPI_ISL_941987, EPI_ISL_941988, EPI_ISL_941989, EPI_ISL_941990, EPI_ISL_941991, EPI_ISL_941992, EPI_ISL_941993, EPI_ISL_941994, EPI_ISL_942003, EPI_ISL_942006			
see above	Instituto Nacional de Salud, Bogotá, Colombia	Centro de Investigaciones en Microbiología y Biotecnología-UR (CIMBIUR), Facultad de Ciencias Naturales, Universidad del Rosario, Bogotá, Colombia Instituto Nacional de Salud, Bogotá, Colombia Icahn School of Medicine at Mount Sinai, New York, USA	Adriana van de Guchte; Alberto Paniz-Mondolfi; Ana S. Gonzalez-Reiche; Carolina Flórez; Carolina Hernández; Emilia Mia Sordillo; Hala Alejel Alshammary; Harm van Bakel; Jayeeta Dutta; Juan David Ramírez; Luz Helena Patiño; Marina Muñoz; Matthew M. Hernandez; Nathalia Ballesteros; Sergio Gomez; Viviana Simon; Zenab Khan
EPI_ISL_447755, EPI_ISL_447756, EPI_ISL_447757, EPI_ISL_447758, EPI_ISL_447759, EPI_ISL_447760, EPI_ISL_447761, EPI_ISL_447762, EPI_ISL_447763, EPI_ISL_447764, EPI_ISL_447765, EPI_ISL_447766, EPI_ISL_447767, EPI_ISL_447768, EPI_ISL_447769, EPI_ISL_447771, EPI_ISL_447772, EPI_ISL_447774, EPI_ISL_447775, EPI_ISL_447776, EPI_ISL_447777, EPI_ISL_447778, EPI_ISL_447779, EPI_ISL_447780, EPI_ISL_447781, EPI_ISL_447782, EPI_ISL_447783, EPI_ISL_447784, EPI_ISL_447785, EPI_ISL_447786, EPI_ISL_447787, EPI_ISL_447789, EPI_ISL_447790, EPI_ISL_447791, EPI_ISL_447792, EPI_ISL_447793, EPI_ISL_447794, EPI_ISL_447795, EPI_ISL_447796, EPI_ISL_447797, EPI_ISL_447798, EPI_ISL_447799, EPI_ISL_447800, EPI_ISL_447801, EPI_ISL_447802, EPI_ISL_447803, EPI_ISL_447804, EPI_ISL_447805, EPI_ISL_447806, EPI_ISL_447807, EPI_ISL_447808, EPI_ISL_447809, EPI_ISL_447810, EPI_ISL_447811, EPI_ISL_447812, EPI_ISL_447813, EPI_ISL_447814, EPI_ISL_447815, EPI_ISL_447816, EPI_ISL_447817			
see above	Instituto Nacional de Salud, Bogotá, Colombia	Grupo de Investigaciones Microbiológicas-UR (GIMUR), Departamento de Biología, Facultad de Ciencias Naturales, Universidad del Rosario, Bogotá, Colombia Instituto Nacional de Salud, Bogotá, Colombia Icahn School of Medicine at Mount Sinai, New York, USA	Adriana Castillo; Alberto Paniz-Mondolfi; Ana S. Gonzalez-Reiche; Angelica Rico; Aníbal A. Teherán; Carolina Florez; Carolina Hernandez; David Martínez; Emilia Mia Sordillo; Esther C. Barros; Harm van Bakel; Jesús E. Jaimes; Juan David Ramírez; Laura Vega; Lisseth Pardo; Marina Muñoz; Martha L. Ospina; Matthew M. Hernandez; Nathalia Ballesteros; Sergio Castañeda; Sergio Gomez; Viviana Simon
EPI_ISL_497736, EPI_ISL_497738, EPI_ISL_497744, EPI_ISL_497745, EPI_ISL_498152, EPI_ISL_498153, EPI_ISL_498154, EPI_ISL_498155, EPI_ISL_498156, EPI_ISL_498157, EPI_ISL_498158, EPI_ISL_498159, EPI_ISL_498160, EPI_ISL_498161, EPI_ISL_498162, EPI_ISL_498163, EPI_ISL_498164, EPI_ISL_498165, EPI_ISL_498166, EPI_ISL_498167, EPI_ISL_498168, EPI_ISL_498169, EPI_ISL_498170, EPI_ISL_526932, EPI_ISL_526933, EPI_ISL_526934, EPI_ISL_526949, EPI_ISL_526950, EPI_ISL_526951, EPI_ISL_526953, EPI_ISL_526954, EPI_ISL_526955, EPI_ISL_526956, EPI_ISL_526957, EPI_ISL_526958, EPI_ISL_526959, EPI_ISL_526960, EPI_ISL_526961, EPI_ISL_526962, EPI_ISL_526963, EPI_ISL_526964, EPI_ISL_526965, EPI_ISL_526967, EPI_ISL_526968, EPI_ISL_526969, EPI_ISL_526970, EPI_ISL_526971, EPI_ISL_526973, EPI_ISL_526974, EPI_ISL_653745, EPI_ISL_653746, EPI_ISL_653747, EPI_ISL_653748, EPI_ISL_653749, EPI_ISL_653750, EPI_ISL_653751, EPI_ISL_653752, EPI_ISL_653753, EPI_ISL_653754, EPI_ISL_653755, EPI_ISL_653756, EPI_ISL_653757, EPI_ISL_653758, EPI_ISL_653759, EPI_ISL_653760, EPI_ISL_653761, EPI_ISL_653762, EPI_ISL_653824, EPI_ISL_653825, EPI_ISL_654793, EPI_ISL_739663, EPI_ISL_739664, EPI_ISL_739665, EPI_ISL_739666, EPI_ISL_739667, EPI_ISL_739668, EPI_ISL_739669, EPI_ISL_739670, EPI_ISL_739671, EPI_ISL_739672, EPI_ISL_739673, EPI_ISL_739674, EPI_ISL_739675, EPI_ISL_739676, EPI_ISL_739677, EPI_ISL_739678, EPI_ISL_739679, EPI_ISL_739680, EPI_ISL_739681, EPI_ISL_739682, EPI_ISL_739683			
see above	Instituto Nacional de Salud, Bogotá, Colombia	Instituto Nacional de Salud, Bogotá, Colombia	Astrid C. Flórez; Carlos Andrés Durán; Carlos Franco-Muñoz; Carolina Ferro; Christian Julian Villabona-Arenas; Diana Marcela Walteros-Acero; Diego A. Álvarez-Díaz; Diego Andrés Prada; Edmilson F. de Oliveira-Filho; Felix Betzler; Franklin Prieto; Jeadran Malagón-Rojas; Jonathan Reales; Jose A. Usme-Ciro; Katherine Laiton-Donato; Liz Villabona-Arenas; Magdalena Weisner; Marcela Mercado-Reyes; Martha Lucia Ospina Martinez; Mauricio Pacheco-Montealegre; Nicolas D. Franco-Sierra; Sheryl Corchuelo; Sussy Echeverria; Wendy K. Jo; Zulma M. Cucunubá
EPI_ISL_1424059, EPI_ISL_1424060, EPI_ISL_1424061, EPI_ISL_1424062, EPI_ISL_1424063, EPI_ISL_1494947	Instituto Nacional de Salud- Dirección de Investigación en Salud Pública	Instituto Nacional de Salud- Dirección de Investigación en Salud Pública	Carlos Franco-Muñoz; Carmen Osorio; Diana Malo; Diego A. Álvarez-Díaz; Diego Andrés Prada; Gerardo Santamaría; Hector Alejandro Ruiz-Moreno; Jhonnatan Reales-González; Juan Camilo Martinez; Julian Naizaque; Katherine Laiton-Donato; Lisseth Pardo; Magdalena Wiesner; Marcela Mercado-Reyes; Marcela Mercado-Reyes.; Maria T. Herrera-Sepúlveda; Marta Lopez Blanco; Martha Lucia Ospina Martinez; Martha Lucia Ospina Martínez; Mauricio Pacheco; Paola Rojas; Sergio Gomez; Sheryll Corchuelo; Ángela Alarcon Cruz
EPI_ISL_956282, EPI_ISL_956283, EPI_ISL_956284, EPI_ISL_956285, EPI_ISL_956286, EPI_ISL_956287, EPI_ISL_956288, EPI_ISL_956289, EPI_ISL_956290, EPI_ISL_956291, EPI_ISL_956292, EPI_ISL_956293, EPI_ISL_956294, EPI_ISL_956295, EPI_ISL_956296, EPI_ISL_956297, EPI_ISL_1017705, EPI_ISL_1303366, EPI_ISL_1303367, EPI_ISL_1303376, EPI_ISL_1303377			
see above	Instituto Nacional de Salud- Dirección de Redes de Laboratorios de Salud Pública	Instituto Nacional de Salud- Dirección de Investigación en Salud Pública	Carlos Franco-Muñoz; Carmen Osorio; Diana Malo; Diego A. Álvarez-Díaz; Diego Andrés Prada; Gerardo Santamaría; Hector Alejandro Ruiz-Moreno; Jhonnatan Reales-González; Juan Camilo Martinez; Julian Naizaque; Katherine Laiton-Donato; Lisseth Pardo; Magdalena Wiesner; Marcela Mercado-Reyes; Maria T. Herrera-Sepúlveda; Marta Lopez Blanco; Martha Lucia Ospina Martinez; Mauricio Pacheco-Montealegre; Sergio Gomez; Sheryll Corchuelo; Ángela Alarcon Cruz
EPI_ISL_2657877, EPI_ISL_2657878	Instituto de Diagnóstico Médico	Instituto Nacional de Salud- Dirección de Investigación en Salud Pública	Carlos Franco-Muñoz; Carmen Osorio; Diana Malo; Diego A. Álvarez-Díaz; Diego Andrés Prada; Gerardo Santamaría; Hector Alejandro Ruiz-Moreno; Jhonnatan Reales-González; Jorge Rivera; Juan Camilo Martinez; Julian Naizaque; Katherine Laiton-Donato; Lisseth Pardo; Magdalena Wiesner; Marcela Mercado-Reyes; Maria T. Herrera-Sepúlveda; Marta Lopez Blanco; Martha Lucia Ospina Martinez; Paola Rojas; Sergio Gomez; Sheryll Corchuelo; Ángela Alarcon Cruz
EPI_ISL_4006773, EPI_ISL_4006774, EPI_ISL_4006775, EPI_ISL_4006776, EPI_ISL_4006777, EPI_ISL_4006778, EPI_ISL_4207333, EPI_ISL_4207334			
see above	Instituto de Investigaciones Biológicas del Trópico (IIBT)	Corporación para Investigaciones Biológicas-CIB	Jeanneth Mosquera Rendon; Katterine Molina Hoyos; Marcela Mercado Reyes; Uriel A. Hurtado Paez
EPI_ISL_1220061, EPI_ISL_1220062, EPI_ISL_1220063, EPI_ISL_1220064, EPI_ISL_1220065, EPI_ISL_1494940	Investigaciones Biomédicas - Universidad de Sucre	Instituto Nacional de Salud- Dirección de Investigación en Salud Pública	Carlos Franco-Muñoz; Carmen Osorio; Diana Malo; Diego A. Álvarez-Díaz; Diego Andrés Prada; Gerardo Santamaría; Hector Alejandro Ruiz-Moreno; Jhonnatan Reales-González; Juan Camilo Martinez; Julian Naizaque; Katherine Laiton-Donato; Lisseth Pardo; Magdalena Wiesner; Marcela Mercado-Reyes; Marcela Mercado-Reyes.; Maria T. Herrera-Sepúlveda; Marta Lopez Blanco; Martha Lucia Ospina Martinez; Martha Lucia Ospina Martínez; Paola Rojas; Sergio Gomez; Sheryll Corchuelo; Ángela Alarcon Cruz
EPI_ISL_3462742	LAB. ANGELES BIOLOGIA MOLECULAR	Instituto Nacional de Salud	Carlos Franco-Muñoz; Carmen Osorio; Diana Malo; Diego A. Álvarez-Díaz; Diego Andrés Prada; Gerardo Santamaría; Hector Alejandro Ruiz-Moreno; Jhonnatan Reales-González; Jorge Rivera; Juan Camilo Martinez; Julian Naizaque; Katherine Laiton-Donato; Lisseth Pardo; Magdalena Wiesner; Marcela Mercado-Reyes; Maria T. Herrera-Sepúlveda; Marta Lopez Blanco; Martha Lucia Ospina Martinez; Paola Rojas; Sergio Gomez; Sheryll Corchuelo; Ángela Alarcon Cruz
EPI_ISL_3355517, EPI_ISL_3369923, EPI_ISL_3369926, EPI_ISL_3369932, EPI_ISL_3369938, EPI_ISL_3369939, EPI_ISL_3369945, EPI_ISL_3369946, EPI_ISL_3369950			
see above	LABIORATORIO HIGUERA ESCALANTE	Corporacion CorpoGen-Universidad de los Andes-Universidad Central	Christian Romero; Jorge Duitama; Juan Manuel Anzola; Laura González; Maryam Chaib De Mares; María Mercedes Zambrano; Nelly Díaz; Patricia Del Portillo; Silvia Restrepo
EPI_ISL_1629760, EPI_ISL_1629761	LABMÉDICO	Universidad Nacional de Colombia - Laboratorio Genómico One Health	Andres F. Cardona-Rios; Carlos Franco-Muñoz; Daniel O. Maldonado-Perez; Diego A. Álvarez-Díaz; Eliana Patricia Calvo Tapiero; Hector Alejandro Ruiz-Moreno; Idabely Betancur Ortiz; Jorge E. Osorio; Juan P. Hernandez-Ortiz; Karl A Ciuoderis; Laura Silvana Perez; Marcela Mercado-Reyes; Maria Stella López; Sandra Ines Cano; Simón Villegas Velásquez
EPI_ISL_956303	LABORATORIO ANALIZAR SYNLAB	Instituto Nacional de Salud- Dirección de Investigación en Salud Pública	Carlos Franco-Muñoz; Diego A. Álvarez-Díaz; Diego Andrés Prada; Gerardo Santamaría; Hector Alejandro Ruiz-Moreno; Jhonnatan Reales-González; Julian Naizaque; Katherine Laiton-Donato; Magdalena Wiesner; Marcela Mercado-Reyes; Maria T. Herrera-Sepúlveda; Martha Lucia Ospina Martinez; Mauricio Pacheco-Montealegre; Sheryll Corchuelo
EPI_ISL_4300691	LABORATORIO ANGELES BIOLOGIA MOLECULAR IPS	Instituto Nacional de Salud- Dirección de Investigación en Salud Pública	Carlos Franco-Muñoz; Carmen Osorio; Diana Malo; Diego A. Álvarez-Díaz; Diego Andrés Prada; Gerardo Santamaría; Hector Alejandro Ruiz-Moreno; Jhonnatan Reales-González; Jorge Rivera; Juan Camilo Martinez; Julian Naizaque; Katherine Laiton-Donato; Lisseth Pardo; Magdalena Wiesner; Marcela Mercado-Reyes; Maria T. Herrera-Sepúlveda; Marta Lopez Blanco; Martha Lucia Ospina Martinez; Paola Rojas; Sergio Gomez; Sheryll Corchuelo; Ángela Alarcon Cruz
EPI_ISL_2657873, EPI_ISL_2657874, EPI_ISL_2828041, EPI_ISL_2828042, EPI_ISL_2828043, EPI_ISL_2828044	LABORATORIO BILOGIA MOLECULAR IMAT SAS	Instituto Nacional de Salud- Dirección de Investigación en Salud Pública	Carlos Franco-Muñoz; Carmen Osorio; Diana Malo; Diego A. Álvarez-Díaz; Diego Andrés Prada; Gerardo Santamaría; Hector Alejandro Ruiz-Moreno; Jhonnatan Reales-González; Jorge Rivera; Juan Camilo Martinez; Julian Naizaque; Katherine Laiton-Donato; Lisseth Pardo; Magdalena Wiesner; Marcela Mercado-Reyes; Maria T. Herrera-Sepúlveda; Marta Lopez Blanco; Martha Lucia Ospina Martinez; Paola Rojas; Sergio Gomez; Sheryll Corchuelo; Ángela Alarcon Cruz
EPI_ISL_2674286, EPI_ISL_2674287, EPI_ISL_2674288	LABORATORIO BIO-TEST	Instituto Nacional de Salud- Dirección de Investigación en Salud Pública	Carlos Franco-Muñoz; Carmen Osorio; Diana Malo; Diego A. Álvarez-Díaz; Diego Andrés Prada; Gerardo Santamaría; Hector Alejandro Ruiz-Moreno; Jhonnatan Reales-González; Jorge Rivera; Juan Camilo Martinez; Julian Naizaque; Katherine Laiton-Donato; Lisseth Pardo; Magdalena Wiesner; Marcela Mercado-Reyes; Maria T. Herrera-Sepúlveda; Marta Lopez Blanco; Martha Lucia Ospina Martinez; Paola Rojas; Sergio Gomez; Sheryll Corchuelo; Ángela Alarcon Cruz
EPI_ISL_1220055, EPI_ISL_1220056, EPI_ISL_1220057	LABORATORIO BIOLOGIA MOLECULAR IMAT SAS	Instituto Nacional de Salud- Dirección de Investigación en Salud Pública	Carlos Franco-Muñoz; Diego A. Álvarez-Díaz; Diego Andrés Prada; Gerardo Santamaría; Hector Alejandro Ruiz-Moreno; Jhonnatan Reales-González; Julian Naizaque; Katherine Laiton-Donato; Magdalena Wiesner; Marcela Mercado-Reyes.; Maria T. Herrera-Sepúlveda; Martha Lucia Ospina Martínez; Sheryll Corchuelo
EPI_ISL_2674297, EPI_ISL_2674298, EPI_ISL_2674299, EPI_ISL_2674300	LABORATORIO CARVAJAL	Instituto Nacional de Salud- Dirección de Investigación en Salud Pública	Carlos Franco-Muñoz; Carmen Osorio; Diana Malo; Diego A. Álvarez-Díaz; Diego Andrés Prada; Gerardo Santamaría; Hector Alejandro Ruiz-Moreno; Jhonnatan Reales-González; Jorge Rivera; Juan Camilo Martinez; Julian Naizaque; Katherine Laiton-Donato; Lisseth Pardo; Magdalena Wiesner; Marcela Mercado-Reyes; Maria T. Herrera-Sepúlveda; Marta Lopez Blanco; Martha Lucia Ospina Martinez; Paola Rojas; Sergio Gomez; Sheryll Corchuelo; Ángela Alarcon Cruz
EPI_ISL_2657870, EPI_ISL_2657871, EPI_ISL_2657872	LABORATORIO CLINICA IMBANACO	Instituto Nacional de Salud- Dirección de Investigación en Salud Pública	Carlos Franco-Muñoz; Carmen Osorio; Diana Malo; Diego A. Álvarez-Díaz; Diego Andrés Prada; Gerardo Santamaría; Hector Alejandro Ruiz-Moreno; Jhonnatan Reales-González; Jorge Rivera; Juan Camilo Martinez; Julian Naizaque; Katherine Laiton-Donato; Lisseth Pardo; Magdalena Wiesner; Marcela Mercado-Reyes; Maria T. Herrera-Sepúlveda; Marta Lopez Blanco; Martha Lucia Ospina Martinez; Paola Rojas; Sergio Gomez; Sheryll Corchuelo; Ángela Alarcon Cruz
EPI_ISL_3462745, EPI_ISL_3462746, EPI_ISL_3462749	LABORATORIO CLINICO BIO-TEST	Instituto Nacional de Salud	Carlos Franco-Muñoz; Carmen Osorio; Diana Malo; Diego A. Álvarez-Díaz; Diego Andrés Prada; Gerardo Santamaría; Hector Alejandro Ruiz-Moreno; Jhonnatan Reales-González; Jorge Rivera; Juan Camilo Martinez; Julian Naizaque; Katherine Laiton-Donato; Lisseth Pardo; Magdalena Wiesner; Marcela Mercado-Reyes; Maria T. Herrera-Sepúlveda; Marta Lopez Blanco; Martha Lucia Ospina Martinez; Paola Rojas; Sergio Gomez; Sheryll Corchuelo; Ángela Alarcon Cruz
EPI_ISL_3385769, EPI_ISL_3385773, EPI_ISL_3385774, EPI_ISL_3385775, EPI_ISL_3385780, EPI_ISL_3385788, EPI_ISL_3385789, EPI_ISL_3385791, EPI_ISL_3385811, EPI_ISL_3385818, EPI_ISL_3385822, EPI_ISL_3385837, EPI_ISL_3385839, EPI_ISL_3385842, EPI_ISL_3385845, EPI_ISL_3385848			
see above	LABORATORIO CLINICO CRISTIAN GRAM IPS SAS	Instituto Nacional de Salud- Dirección de Investigación en Salud Pública	Carlos Franco-Muñoz; Carmen Osorio; Diana Malo; Diego A. Álvarez-Díaz; Diego Andrés Prada; Gerardo Santamaría; Hector Alejandro Ruiz-Moreno; Jhonnatan Reales-González; Jorge Rivera; Juan Camilo Martinez; Julian Naizaque; Katherine Laiton-Donato; Lisseth Pardo; Magdalena Wiesner; Marcela Mercado-Reyes; Maria T. Herrera-Sepúlveda; Marta Lopez Blanco; Martha Lucia Ospina Martinez; Paola Rojas; Sergio Gomez; Sheryll Corchuelo; Ángela Alarcon Cruz

EPI_ISL_4300696	LABORATORIO CLINICO ESPECIALIZADO AIDA ASCENCIO	Instituto Nacional de Salud- Dirección de Investigación en Salud Pública	Carlos Franco-Muñoz; Carmen Osorio; Diana Malo; Diego A. Álvarez-Díaz; Diego Andrés Prada; Gerardo Santamaría; Hector Alejandro Ruiz-Moreno; Jhonnatan Reales-González; Jorge Rivera; Juan Camilo Martinez; Julian Naizaque; Katherine Laiton-Donato; Lisseth Pardo; Magdalena Wiesner; Marcela Mercado-Reyes; Maria T. Herrera-Sepúlveda; Marta Lopez Blanco; Martha Lucia Ospina Martinez; Paola Rojas; Sergio Gomez; Sheryll Corchuelo; Ángela Alarcon Cruz
EPI_ISL_2834727, EPI_ISL_2834730, EPI_ISL_2834731, EPI_ISL_3385805, EPI_ISL_3385825, EPI_ISL_3385832, EPI_ISL_3385851			
see above	LABORATORIO CLINICO HIGUERA ESCALANTE	Instituto Nacional de Salud- Dirección de Investigación en Salud Pública	Carlos Franco-Muñoz; Carmen Osorio; Diana Malo; Diego A. Álvarez-Díaz; Diego Andrés Prada; Gerardo Santamaría; Hector Alejandro Ruiz-Moreno; Jhonnatan Reales-González; Jorge Rivera; Juan Camilo Martinez; Julian Naizaque; Katherine Laiton-Donato; Lisseth Pardo; Magdalena Wiesner; Marcela Mercado-Reyes; Maria T. Herrera-Sepúlveda; Marta Lopez Blanco; Martha Lucia Ospina Martinez; Paola Rojas; Sergio Gomez; Sheryll Corchuelo; Ángela Alarcon Cruz
EPI_ISL_1494945, EPI_ISL_1494946	LABORATORIO CLINICO IMAT SAS	Instituto Nacional de Salud- Dirección de Investigación en Salud Pública	Carlos Franco-Muñoz; Carmen Osorio; Diana Malo; Diego A. Álvarez-Díaz; Diego Andrés Prada; Gerardo Santamaría; Hector Alejandro Ruiz-Moreno; Jhonnatan Reales-González; Juan Camilo Martinez; Julian Naizaque; Katherine Laiton-Donato; Lisseth Pardo; Magdalena Wiesner; Marcela Mercado-Reyes; Maria T. Herrera-Sepúlveda; Marta Lopez Blanco; Martha Lucia Ospina Martinez; Paola Rojas; Sergio Gomez; Sheryll Corchuelo; Ángela Alarcon Cruz
EPI_ISL_2155045, EPI_ISL_2158347, EPI_ISL_2158350, EPI_ISL_2674290, EPI_ISL_2674291, EPI_ISL_2674292, EPI_ISL_2674293, EPI_ISL_2861247, EPI_ISL_2861248			
see above	LABORATORIO CLINICO MASVIDA DE LA COSTA	Instituto Nacional de Salud- Dirección de Investigación en Salud Pública	Carlos Franco-Muñoz; Carmen Osorio; Diana Malo; Diego A. Álvarez-Díaz; Diego Andrés Prada; Gerardo Santamaría; Hector Alejandro Ruiz-Moreno; Jhonnatan Reales-González; Jorge Rivera; Juan Camilo Martinez; Julian Naizaque; Katherine Laiton-Donato; Lisseth Pardo; Magdalena Wiesner; Marcela Mercado-Reyes; Maria T. Herrera-Sepúlveda; Marta Lopez Blanco; Martha Lucia Ospina Martinez; Paola Rojas; Sergio Gomez; Sheryll Corchuelo; Ángela Alarcon Cruz
EPI_ISL_1821062, EPI_ISL_1821063, EPI_ISL_1821064, EPI_ISL_1821065	LABORATORIO CLINICO SYNLAB	Instituto Nacional de Salud- Dirección de Investigación en Salud Pública	Carlos Franco-Muñoz; Carmen Osorio; Diana Malo; Diego A. Álvarez-Díaz; Diego Andrés Prada; Gerardo Santamaría; Hector Alejandro Ruiz-Moreno; Jhonnatan Reales-González; Jorge Rivera; Juan Camilo Martinez; Julian Naizaque; Katherine Laiton-Donato; Lisseth Pardo; Magdalena Wiesner; Marcela Mercado-Reyes; Maria T. Herrera-Sepúlveda; Marta Lopez Blanco; Martha Lucia Ospina Martinez; Paola Rojas; Sergio Gomez; Sheryll Corchuelo; Ángela Alarcon Cruz
EPI_ISL_2155047	LABORATORIO CLINIZAD	Instituto Nacional de Salud- Dirección de Investigación en Salud Pública	Carlos Franco-Muñoz; Carmen Osorio; Diana Malo; Diego A. Álvarez-Díaz; Diego Andrés Prada; Gerardo Santamaría; Hector Alejandro Ruiz-Moreno; Jhonnatan Reales-González; Jorge Rivera; Juan Camilo Martinez; Julian Naizaque; Katherine Laiton-Donato; Lisseth Pardo; Magdalena Wiesner; Marcela Mercado-Reyes; Maria T. Herrera-Sepúlveda; Marta Lopez Blanco; Martha Lucia Ospina Martinez; Paola Rojas; Sergio Gomez; Sheryll Corchuelo; Ángela Alarcon Cruz
EPI_ISL_1220048	LABORATORIO CLÍNICO FUNDACIÓN HOSPITAL SAN PEDRO	Instituto Nacional de Salud- Dirección de Investigación en Salud Pública	Carlos Franco-Muñoz; Diego A. Álvarez-Díaz; Diego Andrés Prada; Gerardo Santamaría; Hector Alejandro Ruiz-Moreno; Jhonnatan Reales-González; Julian Naizaque; Katherine Laiton-Donato; Magdalena Wiesner; Marcela Mercado-Reyes.; Maria T. Herrera-Sepúlveda; Martha Lucia Ospina Martínez; Sheryll Corchuelo
EPI_ISL_2674282, EPI_ISL_2674283	LABORATORIO CONTINENTAL	Instituto Nacional de Salud- Dirección de Investigación en Salud Pública	Carlos Franco-Muñoz; Carmen Osorio; Diana Malo; Diego A. Álvarez-Díaz; Diego Andrés Prada; Gerardo Santamaría; Hector Alejandro Ruiz-Moreno; Jhonnatan Reales-González; Jorge Rivera; Juan Camilo Martinez; Julian Naizaque; Katherine Laiton-Donato; Lisseth Pardo; Magdalena Wiesner; Marcela Mercado-Reyes; Maria T. Herrera-Sepúlveda; Marta Lopez Blanco; Martha Lucia Ospina Martinez; Paola Rojas; Sergio Gomez; Sheryll Corchuelo; Ángela Alarcon Cruz
EPI_ISL_3355467, EPI_ISL_3355468, EPI_ISL_3355469, EPI_ISL_3355470, EPI_ISL_3355471, EPI_ISL_3355477, EPI_ISL_3369922, EPI_ISL_3369925, EPI_ISL_3369928, EPI_ISL_3369951, EPI_ISL_3369952			
see above	LABORATORIO DE BIOLOGÍA MOLECULAR Y BIOTECNOLOGIA	Corporacion CorpoGen-Universidad de los Andes-Universidad Central	Christian Romero; Jorge Duitama; Juan Manuel Anzola; Laura González; Maryam Chaib De Mares; María Mercedes Zambrano; Nelly Díaz; Patricia Del Portillo; Silvia Restrepo
EPI_ISL_2674325, EPI_ISL_2674326, EPI_ISL_2674327	LABORATORIO DE INFECCION E INMUNIDAD	Instituto Nacional de Salud- Dirección de Investigación en Salud Pública	Carlos Franco-Muñoz; Carmen Osorio; Diana Malo; Diego A. Álvarez-Díaz; Diego Andrés Prada; Gerardo Santamaría; Hector Alejandro Ruiz-Moreno; Jhonnatan Reales-González; Jorge Rivera; Juan Camilo Martinez; Julian Naizaque; Katherine Laiton-Donato; Lisseth Pardo; Magdalena Wiesner; Marcela Mercado-Reyes; Maria T. Herrera-Sepúlveda; Marta Lopez Blanco; Martha Lucia Ospina Martinez; Paola Rojas; Sergio Gomez; Sheryll Corchuelo; Ángela Alarcon Cruz
EPI_ISL_1632505, EPI_ISL_1632506, EPI_ISL_1632507, EPI_ISL_1632508, EPI_ISL_1820934	LABORATORIO DE INVESTIGACION HORMONAL	Instituto Nacional de Salud- Dirección de Investigación en Salud Pública	Carlos Franco-Muñoz; Carmen Osorio; Christian Romero; Diana Malo; Diego A. Álvarez-Díaz; Diego Andrés Prada; Gerardo Santamaría; Hector Alejandro Ruiz-Moreno; Jhonnatan Reales-González; Jorge Rivera; Juan Camilo Martinez; Julian Naizaque; Katherine Laiton-Donato; Lisseth Pardo; Magdalena Wiesner; Marcela Mercado-Reyes; Maria T. Herrera-Sepúlveda; Marta Lopez Blanco; Martha Lucia Ospina Martinez; Paola Rojas; Patricia del Portillo; Sergio Gomez; Sheryll Corchuelo; Ángela Alarcon Cruz
EPI_ISL_1629743, EPI_ISL_1629744, EPI_ISL_1629745, EPI_ISL_1629746, EPI_ISL_1629747, EPI_ISL_1675322, EPI_ISL_1824706, EPI_ISL_1824707, EPI_ISL_2621839, EPI_ISL_2621840, EPI_ISL_2621841, EPI_ISL_2621842, EPI_ISL_2621843, EPI_ISL_2621844, EPI_ISL_2621845, EPI_ISL_2621846, EPI_ISL_2621847, EPI_ISL_2621848, EPI_ISL_2621849, EPI_ISL_2621850, EPI_ISL_2621851, EPI_ISL_2621860, EPI_ISL_2621861, EPI_ISL_2621862, EPI_ISL_2621863, EPI_ISL_2621864, EPI_ISL_2621865, EPI_ISL_2621866, EPI_ISL_2621867, EPI_ISL_2621868, EPI_ISL_2621869, EPI_ISL_2621870, EPI_ISL_2621871, EPI_ISL_2621872, EPI_ISL_2621873, EPI_ISL_2621874, EPI_ISL_2621875			
see above	LABORATORIO ECHAVARRIA	Universidad Nacional de Colombia - Laboratorio Genómico One Health	Andres F. Cardona-Rios; Carlos Franco-Muñoz; Carolina Muñoz-Arango; Celeny Ortiz; Daniel O. Maldonado-Perez; Diego A. Álvarez-Díaz; Eliana Patricia Calvo Tapiero; Hector Alejandro Ruiz-Moreno; Idabely Betancur Ortiz; Jorge E. Osorio; Juan P. Hernandez-Ortiz; Karl A Ciuoderis; Katherine Laiton-Donato; Laura Silvana Perez; Lina M. Hurtado; Marcela Mercado-Reyes; Maria Angélica Maya; Maria Stella López; Rita Almanza Payares; Sandra Ines Cano; Simón Villegas Velásquez
EPI_ISL_2651229, EPI_ISL_2651230, EPI_ISL_2651231	LABORATORIO HEMATOLOGICO	Universidad Nacional de Colombia - Laboratorio Genómico One Health	Andres F. Cardona-Rios; Carlos Franco-Muñoz; Carolina Muñoz-Arango; Celeny Ortiz; Daniel O. Maldonado-Perez; Diego A. Álvarez-Díaz; Hector Alejandro Ruiz-Moreno; Idabely Betancur Ortiz; Jorge E. Osorio; Juan P. Hernandez-Ortiz; Karl A Ciuoderis; Katherine Laiton-Donato; Laura Silvana Perez; Lina M. Hurtado; Marcela Mercado-Reyes; Maria Angélica Maya; Maria Stella López; Rita Almanza Payares; Sandra Ines Cano; Simón Villegas Velásquez
EPI_ISL_3355480	LABORATORIO IMAT	Corporacion CorpoGen-Universidad de los Andes-Universidad Central	Christian Romero; Jorge Duitama; Juan Manuel Anzola; Laura González; Maryam Chaib De Mares; María Mercedes Zambrano; Nelly Díaz; Patricia Del Portillo; Silvia Restrepo
EPI_ISL_956302, EPI_ISL_3385785, EPI_ISL_3385792, EPI_ISL_3385793, EPI_ISL_3385794, EPI_ISL_3385795, EPI_ISL_3385798, EPI_ISL_3385799, EPI_ISL_3385802, EPI_ISL_3385806, EPI_ISL_3385812, EPI_ISL_3385813, EPI_ISL_3385826, EPI_ISL_3385828, EPI_ISL_3385830, EPI_ISL_3385836			
see above	LABORATORIO IMAT	Instituto Nacional de Salud- Dirección de Investigación en Salud Pública	Carlos Franco-Muñoz; Carmen Osorio; Diana Malo; Diego A. Álvarez-Díaz; Diego Andrés Prada; Gerardo Santamaría; Hector Alejandro Ruiz-Moreno; Jhonnatan Reales-González; Jorge Rivera; Juan Camilo Martinez; Julian Naizaque; Katherine Laiton-Donato; Lisseth Pardo; Magdalena Wiesner; Marcela Mercado-Reyes; Maria T. Herrera-Sepúlveda; Marta Lopez Blanco; Martha Lucia Ospina Martinez; Mauricio Pacheco-Montealegre; Paola Rojas; Sergio Gomez; Sheryll Corchuelo; Ángela Alarcon Cruz
EPI_ISL_1673258, EPI_ISL_1673259, EPI_ISL_1673260, EPI_ISL_1673261, EPI_ISL_1673262, EPI_ISL_3275678, EPI_ISL_3275679, EPI_ISL_3275680, EPI_ISL_3275681, EPI_ISL_3275682			
see above	LABORATORIO LAS AMERICAS	Universidad Nacional de Colombia - Laboratorio Genómico One Health	Andres F. Cardona-Rios; Carlos Franco-Muñoz; Carolina Muñoz-Arango; Celeny Ortiz; Daniel O. Maldonado-Perez; Diego A. Álvarez-Díaz; Eliana Patricia Calvo Tapiero; Hector Alejandro Ruiz-Moreno; Idabely Betancur Ortiz; Jorge E. Osorio; Juan P. Hernandez-Ortiz; Karl A Ciuoderis; Katherine Laiton-Donato; Laura Silvana Perez; Lina M. Hurtado; Marcela Mercado-Reyes; Maria Angélica Maya; Maria Stella López; Rita Almanza Payares; Sandra Ines Cano; Simón Villegas Velásquez
EPI_ISL_2362579, EPI_ISL_2362580, EPI_ISL_2362581, EPI_ISL_2362582	LABORATORIO NACY FLOREZ GARCIA	Instituto Nacional de Salud- Dirección de Investigación en Salud Pública	Carlos Franco-Muñoz; Carmen Osorio; Diana Malo; Diego A. Álvarez-Díaz; Diego Andrés Prada; Gerardo Santamaría; Hector Alejandro Ruiz-Moreno; Jhonnatan Reales-González; Jorge Rivera; Juan Camilo Martinez; Julian Naizaque; Katherine Laiton-Donato; Lisseth Pardo; Magdalena Wiesner; Marcela Mercado-Reyes; Maria T. Herrera-Sepúlveda; Marta Lopez Blanco; Martha Lucia Ospina Martinez; Paola Rojas; Sergio Gomez; Sheryll Corchuelo; Ángela Alarcon Cruz
EPI_ISL_3385786, EPI_ISL_3385810, EPI_ISL_3385819, EPI_ISL_3385824	LABORATORIO NANCY FLOREZ GARCIA	Instituto Nacional de Salud- Dirección de Investigación en Salud Pública	Carlos Franco-Muñoz; Carmen Osorio; Diana Malo; Diego A. Álvarez-Díaz; Diego Andrés Prada; Gerardo Santamaría; Hector Alejandro Ruiz-Moreno; Jhonnatan Reales-González; Jorge Rivera; Juan Camilo Martinez; Julian Naizaque; Katherine Laiton-Donato; Lisseth Pardo; Magdalena Wiesner; Marcela Mercado-Reyes; Maria T. Herrera-Sepúlveda; Marta Lopez Blanco; Martha Lucia Ospina Martinez; Paola Rojas; Sergio Gomez; Sheryll Corchuelo; Ángela Alarcon Cruz
EPI_ISL_2362568, EPI_ISL_2362569, EPI_ISL_2362570	LABORATORIO OLIMPUS	Instituto Nacional de Salud- Dirección de Investigación en Salud Pública	Carlos Franco-Muñoz; Carmen Osorio; Diana Malo; Diego A. Álvarez-Díaz; Diego Andrés Prada; Gerardo Santamaría; Hector Alejandro Ruiz-Moreno; Jhonnatan Reales-González; Jorge Rivera; Juan Camilo Martinez; Julian Naizaque; Katherine Laiton-Donato; Lisseth Pardo; Magdalena Wiesner; Marcela Mercado-Reyes; Maria T. Herrera-Sepúlveda; Marta Lopez Blanco; Martha Lucia Ospina Martinez; Paola Rojas; Sergio Gomez; Sheryll Corchuelo; Ángela Alarcon Cruz
EPI_ISL_4300701	LABORATORIOS DEL VALLE S.A.S.	Instituto Nacional de Salud- Dirección de Investigación en Salud Pública	Carlos Franco-Muñoz; Carmen Osorio; Diana Malo; Diego A. Álvarez-Díaz; Diego Andrés Prada; Gerardo Santamaría; Hector Alejandro Ruiz-Moreno; Jhonnatan Reales-González; Jorge Rivera; Juan Camilo Martinez; Julian Naizaque; Katherine Laiton-Donato; Lisseth Pardo; Magdalena Wiesner; Marcela Mercado-Reyes; Maria T. Herrera-Sepúlveda; Marta Lopez Blanco; Martha Lucia Ospina Martinez; Paola Rojas; Sergio Gomez; Sheryll Corchuelo; Ángela Alarcon Cruz
EPI_ISL_1960058, EPI_ISL_3276559, EPI_ISL_3276560	LAS AMERICAS	Universidad Nacional de Colombia - Laboratorio Genómico One Health	Andres F. Cardona-Rios; Carlos Franco-Muñoz; Carolina Muñoz-Arango; Celeny Ortiz; Daniel O. Maldonado-Perez; Diego A. Álvarez-Díaz; Hector Alejandro Ruiz-Moreno; Idabely Betancur Ortiz; Jorge E. Osorio; Juan P. Hernandez-Ortiz; Karl A Ciuoderis; Katherine Laiton-Donato; Laura Silvana Perez; Lina M. Hurtado; Marcela Mercado-Reyes; Maria Angélica Maya; Maria Stella López; Rita Almanza Payares; Sandra Ines Cano; Simón Villegas Velásquez
EPI_ISL_1091784, EPI_ISL_1091785, EPI_ISL_1091786, EPI_ISL_1960051, EPI_ISL_1960052, EPI_ISL_1960057, EPI_ISL_1960065, EPI_ISL_1960068, EPI_ISL_1960075, EPI_ISL_1960077, EPI_ISL_1960078, EPI_ISL_1960081			
see above	LDSP	Universidad Nacional de Colombia - Laboratorio Genómico One Health	Andres F. Cardona-Rios; Carlos Franco-Muñoz; Carolina Muñoz-Arango; Celeny Ortiz; Daniel O. Maldonado-Perez; Diego A. Álvarez-Díaz; Hector Alejandro Ruiz-Moreno; Idabely Betancur Ortiz; Jorge E. Osorio; Juan P. Hernandez-Ortiz; Karl A Ciuoderis; Katherine Laiton-Donato; Laura Silvana Perez; Lina M. Hurtado; Marcela Mercado-Reyes; Maria Angélica Maya; Maria Stella López; Rita Almanza Payares; Sandra Ines Cano; Simón Villegas Velásquez
EPI_ISL_2827765, EPI_ISL_2827783, EPI_ISL_2827787	LDSP - CAQUETA HOSPITAL MALVINAS	Instituto Nacional de Salud- Dirección de Investigación en Salud Pública	Carlos Franco-Muñoz; Carmen Osorio; Diana Malo; Diego A. Álvarez-Díaz; Diego Andrés Prada; Gerardo Santamaría; Hector Alejandro Ruiz-Moreno; Jhonnatan Reales-González; Jorge Rivera; Juan Camilo Martinez; Julian Naizaque; Katherine Laiton-Donato; Lisseth Pardo; Magdalena Wiesner; Marcela Mercado-Reyes; Maria T. Herrera-Sepúlveda; Marta Lopez Blanco; Martha Lucia Ospina Martinez; Paola Rojas; Sergio Gomez; Sheryll Corchuelo; Ángela Alarcon Cruz
EPI_ISL_2827779	LDSP - CAQUETA HOSPITAL MARIA INMACULADA	Instituto Nacional de Salud- Dirección de Investigación en Salud Pública	Carlos Franco-Muñoz; Carmen Osorio; Diana Malo; Diego A. Álvarez-Díaz; Diego Andrés Prada; Gerardo Santamaría; Hector Alejandro Ruiz-Moreno; Jhonnatan Reales-González; Jorge Rivera; Juan Camilo Martinez; Julian Naizaque; Katherine Laiton-Donato; Lisseth Pardo; Magdalena Wiesner; Marcela Mercado-Reyes; Maria T. Herrera-Sepúlveda; Marta Lopez Blanco; Martha Lucia Ospina Martinez; Paola Rojas; Sergio Gomez; Sheryll Corchuelo; Ángela Alarcon Cruz
EPI_ISL_2827768, EPI_ISL_2827771, EPI_ISL_2827773, EPI_ISL_2827778, EPI_ISL_2827782	LDSP - LAB CLINICO ESPECIALIZADO AIDA ASCENCIO	Instituto Nacional de Salud- Dirección de Investigación en Salud Pública	Carlos Franco-Muñoz; Carmen Osorio; Diana Malo; Diego A. Álvarez-Díaz; Diego Andrés Prada; Gerardo Santamaría; Hector Alejandro Ruiz-Moreno; Jhonnatan Reales-González; Jorge Rivera; Juan Camilo Martinez; Julian Naizaque; Katherine Laiton-Donato; Lisseth Pardo; Magdalena Wiesner; Marcela Mercado-Reyes; Maria T. Herrera-Sepúlveda; Marta Lopez Blanco; Martha Lucia Ospina Martinez; Paola Rojas; Sergio Gomez; Sheryll Corchuelo; Ángela Alarcon Cruz
EPI_ISL_2155050	LDSP AMAZONAS	Instituto Nacional de Salud- Dirección de Investigación en Salud Pública	Carlos Franco-Muñoz; Carmen Osorio; Diana Malo; Diego A. Álvarez-Díaz; Diego Andrés Prada; Gerardo Santamaría; Hector Alejandro Ruiz-Moreno; Jhonnatan Reales-González; Jorge Rivera; Juan Camilo Martinez; Julian Naizaque; Katherine Laiton-Donato; Lisseth Pardo; Magdalena Wiesner; Marcela Mercado-Reyes; Maria T. Herrera-Sepúlveda; Marta Lopez Blanco; Martha Lucia Ospina Martinez; Paola Rojas; Sergio Gomez; Sheryll Corchuelo; Ángela Alarcon Cruz
EPI_ISL_2158348	LDSP ANTIOQUIA	Instituto Nacional de Salud- Dirección de Investigación en Salud Pública	Carlos Franco-Muñoz; Carmen Osorio; Diana Malo; Diego A. Álvarez-Díaz; Diego Andrés Prada; Gerardo Santamaría; Hector Alejandro Ruiz-Moreno; Jhonnatan Reales-González; Jorge Rivera; Juan Camilo Martinez; Julian Naizaque; Katherine Laiton-Donato; Lisseth Pardo; Magdalena Wiesner; Marcela Mercado-Reyes; Maria T. Herrera-Sepúlveda; Marta Lopez Blanco; Martha Lucia Ospina Martinez; Paola Rojas; Sergio Gomez; Sheryll Corchuelo; Ángela Alarcon Cruz
EPI_ISL_4300697, EPI_ISL_4300698, EPI_ISL_4300699	LDSP ARAUCA	Instituto Nacional de Salud- Dirección de Investigación en Salud Pública	Carlos Franco-Muñoz; Carmen Osorio; Diana Malo; Diego A. Álvarez-Díaz; Diego Andrés Prada; Gerardo Santamaría; Hector Alejandro Ruiz-Moreno; Jhonnatan Reales-González; Jorge Rivera; Juan Camilo Martinez; Julian Naizaque; Katherine Laiton-Donato; Lisseth Pardo; Magdalena Wiesner; Marcela Mercado-Reyes; Maria T. Herrera-Sepúlveda; Marta Lopez Blanco; Martha Lucia Ospina Martinez; Paola Rojas; Sergio Gomez; Sheryll Corchuelo; Ángela Alarcon Cruz
EPI_ISL_1632510, EPI_ISL_1632511, EPI_ISL_1820959	LDSP Amazonas	Instituto Nacional de Salud- Dirección de Investigación en Salud Pública	Carlos Franco-Muñoz; Carmen Osorio; Christian Romero; Diana Malo; Diego A. Álvarez-Díaz; Diego Andrés Prada; Gerardo Santamaría; Hector Alejandro Ruiz-Moreno; Jhonnatan Reales-González; Jorge Rivera; Juan Camilo Martinez; Julian Naizaque; Katherine Laiton-Donato; Lisseth Pardo; Magdalena Wiesner; Marcela Mercado-Reyes; Maria T. Herrera-Sepúlveda; Marta Lopez Blanco; Martha Lucia Ospina Martinez; Paola Rojas; Patricia del Portillo; Sergio Gomez; Sheryll Corchuelo; Ángela Alarcon Cruz
EPI_ISL_1582996, EPI_ISL_1632512, EPI_ISL_1820927, EPI_ISL_1820929, EPI_ISL_1820930, EPI_ISL_1820932, EPI_ISL_2009066, EPI_ISL_2861246			
see above	LDSP BARRANQUILLA	Instituto Nacional de Salud- Dirección de Investigación en Salud Pública	Carlos Franco-Muñoz; Carmen Osorio; Christian Romero; Diana Malo; Diego A. Álvarez-Díaz; Diego Andrés Prada; Gerardo Santamaría; Hector Alejandro Ruiz-Moreno; Jhonnatan Reales-González; Jorge Rivera; Juan Camilo Martinez; Julian Naizaque; Katherine Laiton-Donato; Lisseth Pardo; Magdalena Wiesner; Marcela Mercado-Reyes; Maria T. Herrera-Sepúlveda; Marta Lopez Blanco; Martha Lucia Ospina

			Martinez; Paola Rojas; Patricia del Portillo; Sergio Gomez; Sheryll Corchuelo; Ángela Alarcon Cruz
EPI_ISL_1632514, EPI_ISL_1652039, EPI_ISL_1652040, EPI_ISL_1652041, EPI_ISL_1820951	LDSP BOGOTA	Instituto Nacional de Salud- Dirección de Investigación en Salud Pública	Carlos Franco-Muñoz; Carmen Osorio; Christian Romero; Diana Malo; Diego A. Álvarez-Díaz; Diego Andrés Prada; Gerardo Santamaría; Hector Alejandro Ruiz-Moreno; Jhonnatan Reales-González; Jorge Rivera; Juan Camilo Martinez; Julian Naizaque; Katherine Laiton-Donato; Lisseth Pardo; Magdalena Wiesner; Marcela Mercado-Reyes; Maria T. Herrera-Sepúlveda; Marta Lopez Blanco; Martha Lucia Ospina Martinez; Paola Rojas; Patricia del Portillo; Sergio Gomez; Sheryll Corchuelo; Ángela Alarcon Cruz
EPI_ISL_3385804, EPI_ISL_3385827, EPI_ISL_3385833, EPI_ISL_3385841, EPI_ISL_3385852	LDSP BOYACA	Instituto Nacional de Salud- Dirección de Investigación en Salud Pública	Carlos Franco-Muñoz; Carmen Osorio; Diana Malo; Diego A. Álvarez-Díaz; Diego Andrés Prada; Gerardo Santamaría; Hector Alejandro Ruiz-Moreno; Jhonnatan Reales-González; Jorge Rivera; Juan Camilo Martinez; Julian Naizaque; Katherine Laiton-Donato; Lisseth Pardo; Magdalena Wiesner; Marcela Mercado-Reyes; Maria T. Herrera-Sepúlveda; Marta Lopez Blanco; Martha Lucia Ospina Martinez; Paola Rojas; Sergio Gomez; Sheryll Corchuelo; Ángela Alarcon Cruz
EPI_ISL_1632504, EPI_ISL_1805640, EPI_ISL_1805641, EPI_ISL_1805642, EPI_ISL_1805643, EPI_ISL_1805644, EPI_ISL_1805645, EPI_ISL_1805646, EPI_ISL_1805743, EPI_ISL_2009060, EPI_ISL_2155048, EPI_ISL_2155049, EPI_ISL_2674296			
see above	LDSP CALDAS	Instituto Nacional de Salud- Dirección de Investigación en Salud Pública	Carlos Franco-Muñoz; Carmen Osorio; Diana Malo; Diego A. Álvarez-Díaz; Diego Andrés Prada; Gerardo Santamaría; Hector Alejandro Ruiz-Moreno; Jhonnatan Reales-González; Jorge Rivera; Juan Camilo Martinez; Julian Naizaque; Katherine Laiton-Donato; Lisseth Pardo; Magdalena Wiesner; Marcela Mercado-Reyes; Maria T. Herrera-Sepúlveda; Marta Lopez Blanco; Martha Lucia Ospina Martinez; Paola Rojas; Sergio Gomez; Sheryll Corchuelo; Ángela Alarcon Cruz
EPI_ISL_3459386, EPI_ISL_3459387, EPI_ISL_3459389, EPI_ISL_3459398, EPI_ISL_3459413	LDSP CAQUETA	Instituto Nacional de Salud	Carlos Franco-Muñoz; Carmen Osorio; Diana Malo; Diego A. Álvarez-Díaz; Diego Andrés Prada; Gerardo Santamaría; Hector Alejandro Ruiz-Moreno; Jhonnatan Reales-González; Jorge Rivera; Juan Camilo Martinez; Julian Naizaque; Katherine Laiton-Donato; Lisseth Pardo; Magdalena Wiesner; Marcela Mercado-Reyes; Maria T. Herrera-Sepúlveda; Marta Lopez Blanco; Martha Lucia Ospina Martinez; Paola Rojas; Sergio Gomez; Sheryll Corchuelo; Ángela Alarcon Cruz
EPI_ISL_1652045, EPI_ISL_2362562, EPI_ISL_2827767, EPI_ISL_2827777	LDSP CASANARE	Instituto Nacional de Salud- Dirección de Investigación en Salud Pública	Carlos Franco-Muñoz; Carmen Osorio; Christian Romero; Diana Malo; Diego A. Álvarez-Díaz; Diego Andrés Prada; Gerardo Santamaría; Hector Alejandro Ruiz-Moreno; Jhonnatan Reales-González; Jorge Rivera; Juan Camilo Martinez; Julian Naizaque; Katherine Laiton-Donato; Lisseth Pardo; Magdalena Wiesner; Marcela Mercado-Reyes; Maria T. Herrera-Sepúlveda; Marta Lopez Blanco; Martha Lucia Ospina Martinez; Paola Rojas; Patricia del Portillo; Sergio Gomez; Sheryll Corchuelo; Ángela Alarcon Cruz
EPI_ISL_3459392, EPI_ISL_3459399, EPI_ISL_3459400, EPI_ISL_3459401, EPI_ISL_3462743	LDSP CESAR	Instituto Nacional de Salud	Carlos Franco-Muñoz; Carmen Osorio; Diana Malo; Diego A. Álvarez-Díaz; Diego Andrés Prada; Gerardo Santamaría; Hector Alejandro Ruiz-Moreno; Jhonnatan Reales-González; Jorge Rivera; Juan Camilo Martinez; Julian Naizaque; Katherine Laiton-Donato; Lisseth Pardo; Magdalena Wiesner; Marcela Mercado-Reyes; Maria T. Herrera-Sepúlveda; Marta Lopez Blanco; Martha Lucia Ospina Martinez; Paola Rojas; Sergio Gomez; Sheryll Corchuelo; Ángela Alarcon Cruz
EPI_ISL_2362571, EPI_ISL_2362573, EPI_ISL_2362574, EPI_ISL_2362575, EPI_ISL_2362576, EPI_ISL_2362577, EPI_ISL_2362578, EPI_ISL_2674284			
see above	LDSP CESAR	Instituto Nacional de Salud- Dirección de Investigación en Salud Pública	Carlos Franco-Muñoz; Carmen Osorio; Diana Malo; Diego A. Álvarez-Díaz; Diego Andrés Prada; Gerardo Santamaría; Hector Alejandro Ruiz-Moreno; Jhonnatan Reales-González; Jorge Rivera; Juan Camilo Martinez; Julian Naizaque; Katherine Laiton-Donato; Lisseth Pardo; Magdalena Wiesner; Marcela Mercado-Reyes; Maria T. Herrera-Sepúlveda; Marta Lopez Blanco; Martha Lucia Ospina Martinez; Paola Rojas; Sergio Gomez; Sheryll Corchuelo; Ángela Alarcon Cruz
EPI_ISL_3459375, EPI_ISL_3459381, EPI_ISL_3459383, EPI_ISL_3459391, EPI_ISL_3459402, EPI_ISL_3459403	LDSP CUNDINAMARCA	Instituto Nacional de Salud	Carlos Franco-Muñoz; Carmen Osorio; Diana Malo; Diego A. Álvarez-Díaz; Diego Andrés Prada; Gerardo Santamaría; Hector Alejandro Ruiz-Moreno; Jhonnatan Reales-González; Jorge Rivera; Juan Camilo Martinez; Julian Naizaque; Katherine Laiton-Donato; Lisseth Pardo; Magdalena Wiesner; Marcela Mercado-Reyes; Maria T. Herrera-Sepúlveda; Marta Lopez Blanco; Martha Lucia Ospina Martinez; Paola Rojas; Sergio Gomez; Sheryll Corchuelo; Ángela Alarcon Cruz
EPI_ISL_2158353, EPI_ISL_2362585, EPI_ISL_2362586, EPI_ISL_2362587, EPI_ISL_2362588, EPI_ISL_2362589, EPI_ISL_2362590, EPI_ISL_2362591, EPI_ISL_2362592, EPI_ISL_2674289, EPI_ISL_2827786			
see above	LDSP CUNDINAMARCA	Instituto Nacional de Salud- Dirección de Investigación en Salud Pública	Carlos Franco-Muñoz; Carmen Osorio; Diana Malo; Diego A. Álvarez-Díaz; Diego Andrés Prada; Gerardo Santamaría; Hector Alejandro Ruiz-Moreno; Jhonnatan Reales-González; Jorge Rivera; Juan Camilo Martinez; Julian Naizaque; Katherine Laiton-Donato; Lisseth Pardo; Magdalena Wiesner; Marcela Mercado-Reyes; Maria T. Herrera-Sepúlveda; Marta Lopez Blanco; Martha Lucia Ospina Martinez; Paola Rojas; Sergio Gomez; Sheryll Corchuelo; Ángela Alarcon Cruz
EPI_ISL_1626608, EPI_ISL_1626609	LDSP Caldas	Instituto Nacional de Salud- Dirección de Investigación en Salud Pública	Carlos Franco-Muñoz; Carmen Osorio; Diana Malo; Diego A. Álvarez-Díaz; Diego Andrés Prada; Gerardo Santamaría; Hector Alejandro Ruiz-Moreno; Jhonnatan Reales-González; Jorge Rivera; Juan Camilo Martinez; Julian Naizaque; Katherine Laiton-Donato; Lisseth Pardo; Magdalena Wiesner; Marcela Mercado-Reyes; Maria T. Herrera-Sepúlveda; Marta Lopez Blanco; Martha Lucia Ospina Martinez; Paola Rojas; Sergio Gomez; Sheryll Corchuelo; Ángela Alarcon Cruz
EPI_ISL_3319857, EPI_ISL_3320728, EPI_ISL_3320737, EPI_ISL_3320741	LDSP Casanare	Instituto Nacional de Salud	Carlos Franco-Muñoz; Carmen Osorio; Diana Malo; Diego A. Álvarez-Díaz; Diego Andrés Prada; Gerardo Santamaría; Hector Alejandro Ruiz-Moreno; Jhonnatan Reales-González; Jorge Rivera; Juan Camilo Martinez; Julian Naizaque; Katherine Laiton-Donato; Lisseth Pardo; Magdalena Wiesner; Marcela Mercado-Reyes; Maria T. Herrera-Sepúlveda; Marta Lopez Blanco; Martha Lucia Ospina Martinez; Paola Rojas; Sergio Gomez; Sheryll Corchuelo; Ángela Alarcon Cruz
EPI_ISL_3320731, EPI_ISL_3320735, EPI_ISL_3320739, EPI_ISL_3320744, EPI_ISL_3320749	LDSP Cundinamarca	Instituto Nacional de Salud	Carlos Franco-Muñoz; Carmen Osorio; Diana Malo; Diego A. Álvarez-Díaz; Diego Andrés Prada; Gerardo Santamaría; Hector Alejandro Ruiz-Moreno; Jhonnatan Reales-González; Jorge Rivera; Juan Camilo Martinez; Julian Naizaque; Katherine Laiton-Donato; Lisseth Pardo; Magdalena Wiesner; Marcela Mercado-Reyes; Maria T. Herrera-Sepúlveda; Marta Lopez Blanco; Martha Lucia Ospina Martinez; Paola Rojas; Sergio Gomez; Sheryll Corchuelo; Ángela Alarcon Cruz
EPI_ISL_1805635, EPI_ISL_1805636, EPI_ISL_1805637, EPI_ISL_1805638, EPI_ISL_1805639, EPI_ISL_1805742, EPI_ISL_1821069, EPI_ISL_2009063, EPI_ISL_2009064			
see above	LDSP FRONTERIZO	Instituto Nacional de Salud- Dirección de Investigación en Salud Pública	Carlos Franco-Muñoz; Carmen Osorio; Diana Malo; Diego A. Álvarez-Díaz; Diego Andrés Prada; Gerardo Santamaría; Hector Alejandro Ruiz-Moreno; Jhonnatan Reales-González; Jorge Rivera; Juan Camilo Martinez; Julian Naizaque; Katherine Laiton-Donato; Lisseth Pardo; Magdalena Wiesner; Marcela Mercado-Reyes; Maria T. Herrera-Sepúlveda; Marta Lopez Blanco; Martha Lucia Ospina Martinez; Paola Rojas; Sergio Gomez; Sheryll Corchuelo; Ángela Alarcon Cruz
EPI_ISL_2674301, EPI_ISL_2674302, EPI_ISL_2674303, EPI_ISL_2674304, EPI_ISL_2674323, EPI_ISL_2674324	LDSP HUILA	Instituto Nacional de Salud- Dirección de Investigación en Salud Pública	Carlos Franco-Muñoz; Carmen Osorio; Diana Malo; Diego A. Álvarez-Díaz; Diego Andrés Prada; Gerardo Santamaría; Hector Alejandro Ruiz-Moreno; Jhonnatan Reales-González; Jorge Rivera; Juan Camilo Martinez; Julian Naizaque; Katherine Laiton-Donato; Lisseth Pardo; Magdalena Wiesner; Marcela Mercado-Reyes; Maria T. Herrera-Sepúlveda; Marta Lopez Blanco; Martha Lucia Ospina Martinez; Paola Rojas; Sergio Gomez; Sheryll Corchuelo; Ángela Alarcon Cruz
EPI_ISL_2674294, EPI_ISL_2674295	LDSP LETICIA	Instituto Nacional de Salud- Dirección de Investigación en Salud Pública	Carlos Franco-Muñoz; Carmen Osorio; Diana Malo; Diego A. Álvarez-Díaz; Diego Andrés Prada; Gerardo Santamaría; Hector Alejandro Ruiz-Moreno; Jhonnatan Reales-González; Jorge Rivera; Juan Camilo Martinez; Julian Naizaque; Katherine Laiton-Donato; Lisseth Pardo; Magdalena Wiesner; Marcela Mercado-Reyes; Maria T. Herrera-Sepúlveda; Marta Lopez Blanco; Martha Lucia Ospina Martinez; Paola Rojas; Sergio Gomez; Sheryll Corchuelo; Ángela Alarcon Cruz
EPI_ISL_2009061, EPI_ISL_2674310, EPI_ISL_2674311, EPI_ISL_2674312	LDSP NARIÑO	Instituto Nacional de Salud- Dirección de Investigación en Salud Pública	Carlos Franco-Muñoz; Carmen Osorio; Diana Malo; Diego A. Álvarez-Díaz; Diego Andrés Prada; Gerardo Santamaría; Hector Alejandro Ruiz-Moreno; Jhonnatan Reales-González; Jorge Rivera; Juan Camilo Martinez; Julian Naizaque; Katherine Laiton-Donato; Lisseth Pardo; Magdalena Wiesner; Marcela Mercado-Reyes; Maria T. Herrera-Sepúlveda; Marta Lopez Blanco; Martha Lucia Ospina Martinez; Paola Rojas; Sergio Gomez; Sheryll Corchuelo; Ángela Alarcon Cruz
EPI_ISL_1582977, EPI_ISL_1582981, EPI_ISL_1652042, EPI_ISL_1652043, EPI_ISL_1820946	LDSP NORTE DE SANTANDER	Instituto Nacional de Salud- Dirección de Investigación en Salud Pública	Carlos Franco-Muñoz; Carmen Osorio; Christian Romero; Diana Malo; Diego A. Álvarez-Díaz; Diego Andrés Prada; Gerardo Santamaría; Hector Alejandro Ruiz-Moreno; Jhonnatan Reales-González; Jorge Rivera; Juan Camilo Martinez; Julian Naizaque; Katherine Laiton-Donato; Lisseth Pardo; Magdalena Wiesner; Marcela Mercado-Reyes; Maria T. Herrera-Sepúlveda; Marta Lopez Blanco; Martha Lucia Ospina Martinez; Paola Rojas; Patricia del Portillo; Sergio Gomez; Sheryll Corchuelo; Ángela Alarcon Cruz
EPI_ISL_3320745	LDSP Norte de Santander	Instituto Nacional de Salud	Carlos Franco-Muñoz; Carmen Osorio; Diana Malo; Diego A. Álvarez-Díaz; Diego Andrés Prada; Gerardo Santamaría; Hector Alejandro Ruiz-Moreno; Jhonnatan Reales-González; Jorge Rivera; Juan Camilo Martinez; Julian Naizaque; Katherine Laiton-Donato; Lisseth Pardo; Magdalena Wiesner; Marcela Mercado-Reyes; Maria T. Herrera-Sepúlveda; Marta Lopez Blanco; Martha Lucia Ospina Martinez; Paola Rojas; Sergio Gomez; Sheryll Corchuelo; Ángela Alarcon Cruz
EPI_ISL_2894631, EPI_ISL_2894632, EPI_ISL_3385797, EPI_ISL_3385809, EPI_ISL_3385843, EPI_ISL_3385853	LDSP QUINDIO	Instituto Nacional de Salud- Dirección de Investigación en Salud Pública	Carlos Franco-Muñoz; Carmen Osorio; Diana Malo; Diego A. Álvarez-Díaz; Diego Andrés Prada; Gerardo Santamaría; Hector Alejandro Ruiz-Moreno; Jhonnatan Reales-González; Jorge Rivera; Juan Camilo Martinez; Julian Naizaque; Katherine Laiton-Donato; Lisseth Pardo; Magdalena Wiesner; Marcela Mercado-Reyes; Maria T. Herrera-Sepúlveda; Marta Lopez Blanco; Martha Lucia Ospina Martinez; Paola Rojas; Sergio Gomez; Sheryll Corchuelo; Ángela Alarcon Cruz
EPI_ISL_3921330, EPI_ISL_3921332, EPI_ISL_3921333, EPI_ISL_3921335, EPI_ISL_3921336, EPI_ISL_3921338, EPI_ISL_3921339, EPI_ISL_3921341, EPI_ISL_3921343, EPI_ISL_3921344, EPI_ISL_3921346, EPI_ISL_3921347, EPI_ISL_3921349, EPI_ISL_3921350, EPI_ISL_3921352, EPI_ISL_3921354, EPI_ISL_3921355, EPI_ISL_3921357, EPI_ISL_3921358, EPI_ISL_3921361, EPI_ISL_3921363, EPI_ISL_3921365, EPI_ISL_3921366, EPI_ISL_3921368, EPI_ISL_3921370, EPI_ISL_3921371, EPI_ISL_3921373, EPI_ISL_3921374, EPI_ISL_3921376, EPI_ISL_3921377, EPI_ISL_3921379, EPI_ISL_3921380, EPI_ISL_3921382, EPI_ISL_3921383, EPI_ISL_3921385, EPI_ISL_3921386, EPI_ISL_3921388, EPI_ISL_3921389, EPI_ISL_3921391, EPI_ISL_3921392, EPI_ISL_3921394, EPI_ISL_3921395, EPI_ISL_3921396, EPI_ISL_3921398, EPI_ISL_3921399			
see above	LDSP SAN ANDRES ISLA	Instituto Nacional de Salud- Dirección de Investigación en Salud Pública	Carlos Franco-Muñoz; Carmen Osorio; Diana Malo; Diego A. Álvarez-Díaz; Diego Andrés Prada; Gerardo Santamaría; Hector Alejandro Ruiz-Moreno; Jhonnatan Reales-González; Jorge Rivera; Juan Camilo Martinez; Julian Naizaque; Katherine Laiton-Donato; Lisseth Pardo; Magdalena Wiesner; Marcela Mercado-Reyes; Maria T. Herrera-Sepúlveda; Marta Lopez Blanco; Martha Lucia Ospina Martinez; Paola Rojas-Estevez; Sergio Gomez; Sheryll Corchuelo; Ángela Alarcon Cruz
EPI_ISL_1820967, EPI_ISL_1820968, EPI_ISL_1821076, EPI_ISL_1821077, EPI_ISL_2674285, EPI_ISL_2828055, EPI_ISL_2828056			
see above	LDSP SANTANDER	Instituto Nacional de Salud- Dirección de Investigación en Salud Pública	Carlos Franco-Muñoz; Carmen Osorio; Diana Malo; Diego A. Álvarez-Díaz; Diego Andrés Prada; Gerardo Santamaría; Hector Alejandro Ruiz-Moreno; Jhonnatan Reales-González; Jorge Rivera; Juan Camilo Martinez; Julian Naizaque; Katherine Laiton-Donato; Lisseth Pardo; Magdalena Wiesner; Marcela Mercado-Reyes; Maria T. Herrera-Sepúlveda; Marta Lopez Blanco; Martha Lucia Ospina Martinez; Paola Rojas; Sergio Gomez; Sheryll Corchuelo; Ángela Alarcon Cruz
EPI_ISL_1805632, EPI_ISL_2158351, EPI_ISL_2674317, EPI_ISL_2674318, EPI_ISL_2674319	LDSP TOLIMA	Instituto Nacional de Salud- Dirección de Investigación en Salud Pública	Carlos Franco-Muñoz; Carmen Osorio; Diana Malo; Diego A. Álvarez-Díaz; Diego Andrés Prada; Gerardo Santamaría; Hector Alejandro Ruiz-Moreno; Jhonnatan Reales-González; Jorge Rivera; Juan Camilo Martinez; Julian Naizaque; Katherine Laiton-Donato; Lisseth Pardo; Magdalena Wiesner; Marcela Mercado-Reyes; Maria T. Herrera-Sepúlveda; Marta Lopez Blanco; Martha Lucia Ospina Martinez; Paola Rojas; Sergio Gomez; Sheryll Corchuelo; Ángela Alarcon Cruz
EPI_ISL_1652044	LDSP Tolima	Instituto Nacional de Salud- Dirección de Investigación en Salud Pública	Carlos Franco-Muñoz; Carmen Osorio; Christian Romero; Diana Malo; Diego A. Álvarez-Díaz; Diego Andrés Prada; Gerardo Santamaría; Hector Alejandro Ruiz-Moreno; Jhonnatan Reales-González; Jorge Rivera; Juan Camilo Martinez; Julian Naizaque; Katherine Laiton-Donato; Lisseth Pardo; Magdalena Wiesner; Marcela Mercado-Reyes; Maria T. Herrera-Sepúlveda; Marta Lopez Blanco; Martha Lucia Ospina Martinez; Paola Rojas; Patricia del Portillo; Sergio Gomez; Sheryll Corchuelo; Ángela Alarcon Cruz
EPI_ISL_3385820	LDSP VALLE DEL CAUCA	Instituto Nacional de Salud- Dirección de Investigación en Salud Pública	Carlos Franco-Muñoz; Carmen Osorio; Diana Malo; Diego A. Álvarez-Díaz; Diego Andrés Prada; Gerardo Santamaría; Hector Alejandro Ruiz-Moreno; Jhonnatan Reales-González; Jorge Rivera; Juan Camilo Martinez; Julian Naizaque; Katherine Laiton-Donato; Lisseth Pardo; Magdalena Wiesner; Marcela Mercado-Reyes; Maria T. Herrera-Sepúlveda; Marta Lopez Blanco; Martha Lucia Ospina Martinez; Paola Rojas; Sergio Gomez; Sheryll Corchuelo; Ángela Alarcon Cruz
EPI_ISL_1582987, EPI_ISL_1582997	LDSP VICHADA	Instituto Nacional de Salud- Dirección de Investigación en Salud Pública	Carlos Franco-Muñoz; Carmen Osorio; Diana Malo; Diego A. Álvarez-Díaz; Diego Andrés Prada; Gerardo Santamaría; Hector Alejandro Ruiz-Moreno; Jhonnatan Reales-González; Juan Camilo Martinez; Julian Naizaque; Katherine Laiton-Donato; Lisseth Pardo; Magdalena Wiesner; Marcela Mercado-Reyes; Maria T. Herrera-Sepúlveda; Marta Lopez Blanco; Martha Lucia Ospina Martinez; Paola Rojas; Sergio Gomez; Sheryll Corchuelo; Ángela Alarcon Cruz
EPI_ISL_3007486	LDSP VILLAVICENCIO - CLINICA PRIMAVERA DE VILLAVICENCIO	Instituto Nacional de Salud- Dirección de Investigación en Salud Pública	Carlos Franco-Muñoz; Carmen Osorio; Diana Malo; Diego A. Álvarez-Díaz; Diego Andrés Prada; Gerardo Santamaría; Hector Alejandro Ruiz-Moreno; Jhonnatan Reales-González; Jorge Rivera; Juan Camilo Martinez; Julian Naizaque; Katherine Laiton-Donato; Lisseth Pardo; Magdalena Wiesner; Marcela Mercado-Reyes; Maria T. Herrera-Sepúlveda; Marta Lopez Blanco; Martha Lucia Ospina Martinez; Paola Rojas; Sergio Gomez; Sheryll Corchuelo; Ángela Alarcon Cruz
EPI_ISL_3342147, EPI_ISL_3342148, EPI_ISL_3342149, EPI_ISL_3342150, EPI_ISL_3342151, EPI_ISL_3342152	LIME	Laboratorio Departamental de Salud Publica de Antioquia	Andres F. Cardona-Rios; Gloria Isabel Escobar; Idabely Betancur Ortiz; Juan P. Hernandez-Ortiz; Maria Stella López
EPI_ISL_794652, EPI_ISL_794653	LSP DEL TOLIMA	Instituto Nacional de Salud - Dirección de Investigación en Salud Pública	Carlos Franco-Muñoz; Diego A. Álvarez-Díaz; Diego Andrés Prada; Gerardo Santamaría; Jonathan Reales; Julian Naizaque; Katherine Laiton-Donato; Magdalena Wiesner; Marcela Mercado-Reyes; Maria T. Herrera; Martha Lucia Ospina Martinez; Mauricio Pacheco-Montealegre; Paola Muñoz-Laiton; Sheryl Corchuelo
EPI_ISL_4196882, EPI_ISL_4196883, EPI_ISL_4196884, EPI_ISL_4196885, EPI_ISL_4196886, EPI_ISL_4196887, EPI_ISL_4196888, EPI_ISL_4196889, EPI_ISL_4196890, EPI_ISL_4196891, EPI_ISL_4196892, EPI_ISL_4196893, EPI_ISL_4196894, EPI_ISL_4196895, EPI_ISL_4196896, EPI_ISL_4196897, EPI_ISL_4196898, EPI_ISL_4196899, EPI_ISL_4196900, EPI_ISL_4196901, EPI_ISL_4196902, EPI_ISL_4196903, EPI_ISL_4196904, EPI_ISL_4196905, EPI_ISL_4196906, EPI_ISL_4196907, EPI_ISL_4196908, EPI_ISL_4196909, EPI_ISL_4196910, EPI_ISL_4196911, EPI_ISL_4196912, EPI_ISL_4196913, EPI_ISL_4196914, EPI_ISL_4196915, EPI_ISL_4196916, EPI_ISL_4196917, EPI_ISL_4196921, EPI_ISL_4196922, EPI_ISL_4212128, EPI_ISL_4212129, EPI_ISL_4212130, EPI_ISL_4212131, EPI_ISL_4212132, EPI_ISL_4212133,			

EPI_ISL_4212134, EPI_ISL_4212135, EPI_ISL_4212136, EPI_ISL_4212137, EPI_ISL_4212138, EPI_ISL_4212139, EPI_ISL_4212140, EPI_ISL_4212141, EPI_ISL_4212142, EPI_ISL_4212143, EPI_ISL_4212144, EPI_ISL_4212145, EPI_ISL_4212146, EPI_ISL_4212147, EPI_ISL_4212148, EPI_ISL_4212149, EPI_ISL_4212150, EPI_ISL_4212151, EPI_ISL_4212152, EPI_ISL_4212153, EPI_ISL_4212154, EPI_ISL_4212155, EPI_ISL_4212156, EPI_ISL_4212157, EPI_ISL_4212158, EPI_ISL_4212159, EPI_ISL_4212160, EPI_ISL_4212161, EPI_ISL_4212162, EPI_ISL_4212163, EPI_ISL_4212164, EPI_ISL_4212165, EPI_ISL_4212166, EPI_ISL_4212178, EPI_ISL_4212179, EPI_ISL_4212180, EPI_ISL_4212181, EPI_ISL_4212182			
see above	LSP DISTRITAL BOGOTA	Gencore - Universidad de los Andes	Alejandro Gomez; Cristian Barrera; Gabriela Ariza; Gabriela Delgado; Johana Hernandez; Luisa Sacristan; Marcela Guevara; Marcela Mercado; Silvia Restrepo
EPI_ISL_3320736	LSP San Andres	Instituto Nacional de Salud	Carlos Franco-Muñoz; Carmen Osorio; Diana Malo; Diego A. Álvarez-Díaz; Diego Andrés Prada; Gerardo Santamaría; Hector Alejandro Ruiz-Moreno; Jhonnatan Reales-González; Jorge Rivera; Juan Camilo Martinez; Julian Naizaque; Katherine Laiton-Donato; Lisseth Pardo; Magdalena Wiesner; Marcela Mercado-Reyes; Maria T. Herrera-Sepúlveda; Marta Lopez Blanco; Martha Lucia Ospina Martinez; Paola Rojas; Sergio Gomez; Sheryll Corchuelo; Ángela Alarcon Cruz
EPI_ISL_3368471, EPI_ISL_3368473, EPI_ISL_4417563, EPI_ISL_4417564, EPI_ISL_4417565, EPI_ISL_4417566, EPI_ISL_4417567, EPI_ISL_4417568, EPI_ISL_4417569, EPI_ISL_4417570, EPI_ISL_4417571, EPI_ISL_4417572, EPI_ISL_4417573, EPI_ISL_4417574, EPI_ISL_4417575, EPI_ISL_4417576, EPI_ISL_4417577, EPI_ISL_4417578, EPI_ISL_4417579, EPI_ISL_4417580, EPI_ISL_4417585, EPI_ISL_4417586, EPI_ISL_4417587, EPI_ISL_4417588, EPI_ISL_4417589, EPI_ISL_4417590, EPI_ISL_4417591, EPI_ISL_4417592, EPI_ISL_4417593, EPI_ISL_4417594, EPI_ISL_4417595, EPI_ISL_4417596, EPI_ISL_4417597, EPI_ISL_4417598, EPI_ISL_4417599, EPI_ISL_4417600, EPI_ISL_4417612, EPI_ISL_4417614, EPI_ISL_4417616, EPI_ISL_4417617, EPI_ISL_4417620, EPI_ISL_4417623, EPI_ISL_4417625, EPI_ISL_4417630, EPI_ISL_4417632, EPI_ISL_4417633, EPI_ISL_4417634, EPI_ISL_4417635, EPI_ISL_4417637, EPI_ISL_4417638, EPI_ISL_4417639, EPI_ISL_4417640, EPI_ISL_4417643, EPI_ISL_4417644, EPI_ISL_4417647, EPI_ISL_4417649, EPI_ISL_4417652, EPI_ISL_4417653, EPI_ISL_4417659			
see above	LSP de Atlantico	Centro de Investigaciones en Microbiología y Biotecnología-UR (CIMBIUR), Facultad de Ciencias Naturales, Universidad del Rosario, Bogotá, Colombia	Angie Ramírez; Juan David Ramírez; Luz H. Patiño; Marcela Mercado-Reyes; Marina Muñoz; Nathalia Ballesteros; Nicolas Niño; Sergio Castañeda
EPI_ISL_4419176, EPI_ISL_4419177, EPI_ISL_4419178, EPI_ISL_4419179, EPI_ISL_4419180, EPI_ISL_4419181, EPI_ISL_4419182			
see above	Lab Christus Sinergia Salud -Clinica Farallones	Universidad del Valle	Programa Nacional de Caracterización Genómica de SARS-CoV-2
EPI_ISL_2657876	Lab microbiologia FVL	Instituto Nacional de Salud- Dirección de Investigación en Salud Pública	Carlos Franco-Muñoz; Carmen Osorio; Diana Malo; Diego A. Álvarez-Díaz; Diego Andrés Prada; Gerardo Santamaría; Hector Alejandro Ruiz-Moreno; Jhonnatan Reales-González; Jorge Rivera; Juan Camilo Martinez; Julian Naizaque; Katherine Laiton-Donato; Lisseth Pardo; Magdalena Wiesner; Marcela Mercado-Reyes; Maria T. Herrera-Sepúlveda; Marta Lopez Blanco; Martha Lucia Ospina Martinez; Paola Rojas; Sergio Gomez; Sheryll Corchuelo; Ángela Alarcon Cruz
EPI_ISL_2158354, EPI_ISL_2158355	Lab. Virología y Genética Universidad Simón Bolívar	Instituto Nacional de Salud- Dirección de Investigación en Salud Pública	Carlos Franco-Muñoz; Carmen Osorio; Diana Malo; Diego A. Álvarez-Díaz; Diego Andrés Prada; Gerardo Santamaría; Hector Alejandro Ruiz-Moreno; Jhonnatan Reales-González; Jorge Rivera; Juan Camilo Martinez; Julian Naizaque; Katherine Laiton-Donato; Lisseth Pardo; Magdalena Wiesner; Marcela Mercado-Reyes; Maria T. Herrera-Sepúlveda; Marta Lopez Blanco; Martha Lucia Ospina Martinez; Paola Rojas; Sergio Gomez; Sheryll Corchuelo; Ángela Alarcon Cruz
EPI_ISL_3998115, EPI_ISL_3998116, EPI_ISL_3998117, EPI_ISL_3998119, EPI_ISL_3998120, EPI_ISL_3998133, EPI_ISL_3998145, EPI_ISL_3998157, EPI_ISL_3998159			
see above	Lab. Virología y Genética Universidad Simón Bolívar	Laboratorio de Biologia Molecular, Universidad Cooperativa de Colombia, Santa Marta	Andrew S. Muñoz-Gamba; Antonio Acosta; Daniel B. Ramírez-Osorio; José A. Usme-Ciro; Paula A. Quintero-Cortés; Yesid Bello
EPI_ISL_906552	Laboratorio Angel Diagnostica	Instituto Nacional de Salud- Dirección de Investigación en Salud Pública, Universidad de los Andes- Applied genomics research group, Vicerrectoria de Investigación y Creación, Universidad de los Andes- Systems and Computing Engineering Department	Carlos Franco-Muñoz; Diego A. Álvarez-Díaz; Diego Andrés Prada; Gerardo Santamaría Jorge Duitama; Héctor Alejandro Ruiz-Moreno; Jhonnatan Reales-González; Jorge Ivan Diaz; Julian Naizaque; Katherine Laiton-Donato; Laura Natalia Gonzalez; Magdalena Wiesner; Marcela Mercado-Reyes; Maria T. Herrera-Sepúlveda; Martha Lucia Ospina Martinez; Mauricio Pacheco-Montealegre; Sheryll Corchuelo; Silvia Restrepo-Restrepo
EPI_ISL_906553, EPI_ISL_906554, EPI_ISL_906555	Laboratorio Bienestar	Instituto Nacional de Salud- Dirección de Investigación en Salud Pública, Universidad de los Andes- Applied genomics research group, Vicerrectoria de Investigación y Creación, Universidad de los Andes- Systems and Computing Engineering Department	Carlos Franco-Muñoz; Diego A. Álvarez-Díaz; Diego Andrés Prada; Gerardo Santamaría Jorge Duitama; Héctor Alejandro Ruiz-Moreno; Jhonnatan Reales-González; Jorge Ivan Diaz; Julian Naizaque; Katherine Laiton-Donato; Laura Natalia Gonzalez; Magdalena Wiesner; Marcela Mercado-Reyes; Maria T. Herrera-Sepúlveda; Martha Lucia Ospina Martinez; Mauricio Pacheco-Montealegre; Sheryll Corchuelo; Silvia Restrepo-Restrepo
EPI_ISL_4219532	Laboratorio CAFAM	Molecular Genetics and Antimicrobial Resistance - UGRA, Universidad El Bosque	Catalina Espitia; Jinnethe Reyes; Lorena Diaz; Marcela Mercado; Mauricio Pacheco; Rafael Rios; Valentina Martinez
EPI_ISL_3671009, EPI_ISL_3671014, EPI_ISL_3671015, EPI_ISL_3671018, EPI_ISL_3671048, EPI_ISL_3671055, EPI_ISL_3721590, EPI_ISL_3721615, EPI_ISL_3721623			
see above	Laboratorio Centrolab	Universidad Nacional de Colombia - Laboratorio Genómico One Health	Andres F. Cardona-Rios; Carlos Franco-Muñoz; Carolina Muñoz-Arango; Celeny Ortiz; Daniel O. Maldonado-Perez; Diego A. Álvarez-Díaz; Hector Alejandro Ruiz-Moreno; Idabely Betancur Ortiz; Jorge E. Osorio; Juan P. Hernandez-Ortiz; Karl A Ciuoderis; Katherine Laiton-Donato; Laura Silvana Perez; Lina M. Hurtado; Marcela Mercado-Reyes; Maria Angélica Maya; Maria Stella López; Rita Almanza Payares; Sandra Ines Cano; Simón Villegas Velásquez
EPI_ISL_4297526, EPI_ISL_4297527, EPI_ISL_4297528, EPI_ISL_4297529	Laboratorio Claret Ariño Garcia	Centro de Genética y Biología Molecular - Universidad del Magdalena	Andrea M. Ramírez Hernandez; Angel Oviedo Marquez; Daniel Bautista; Edison Lea-Ch; Lyda R. Castro; Maria Teresa Mojica-Ortiz
EPI_ISL_4006779, EPI_ISL_4006780, EPI_ISL_4006781, EPI_ISL_4006782, EPI_ISL_4006783, EPI_ISL_4006784	Laboratorio Claret Ariño Garcia	Corporación para Investigaciones Biológicas-CIB	Jeanneth Mosquera Rendon; Katterine Molina Hoyos; Marcela Mercado Reyes; Uriel A. Hurtado Paez
EPI_ISL_3368472, EPI_ISL_4417559, EPI_ISL_4417560, EPI_ISL_4417561, EPI_ISL_4417581, EPI_ISL_4417601, EPI_ISL_4417602, EPI_ISL_4417603, EPI_ISL_4417604, EPI_ISL_4417605, EPI_ISL_4417606, EPI_ISL_4417607, EPI_ISL_4417608, EPI_ISL_4417609, EPI_ISL_4417610, EPI_ISL_4417611, EPI_ISL_4417615, EPI_ISL_4417619, EPI_ISL_4417621, EPI_ISL_4417622, EPI_ISL_4417624, EPI_ISL_4417626, EPI_ISL_4417627, EPI_ISL_4417628, EPI_ISL_4417629, EPI_ISL_4417631, EPI_ISL_4417641, EPI_ISL_4417642, EPI_ISL_4417645, EPI_ISL_4417648, EPI_ISL_4417657, EPI_ISL_4417660			
see above	Laboratorio Clinica Iberoamericana (Sanitas)	Centro de Investigaciones en Microbiología y Biotecnología-UR (CIMBIUR), Facultad de Ciencias Naturales, Universidad del Rosario, Bogotá, Colombia	Angie Ramírez; Juan David Ramírez; Luz H. Patiño; Marcela Mercado-Reyes; Marina Muñoz; Nathalia Ballesteros; Nicolas Niño; Sergio Castañeda
EPI_ISL_3320730, EPI_ISL_3320734	Laboratorio Clinica del Norte	Instituto Nacional de Salud	Carlos Franco-Muñoz; Carmen Osorio; Diana Malo; Diego A. Álvarez-Díaz; Diego Andrés Prada; Gerardo Santamaría; Hector Alejandro Ruiz-Moreno; Jhonnatan Reales-González; Jorge Rivera; Juan Camilo Martinez; Julian Naizaque; Katherine Laiton-Donato; Lisseth Pardo; Magdalena Wiesner; Marcela Mercado-Reyes; Maria T. Herrera-Sepúlveda; Marta Lopez Blanco; Martha Lucia Ospina Martinez; Paola Rojas; Sergio Gomez; Sheryll Corchuelo; Ángela Alarcon Cruz
EPI_ISL_1220050	Laboratorio Clinico Clinica General del Norte	Instituto Nacional de Salud- Dirección de Investigación en Salud Pública	Carlos Franco-Muñoz; Diego A. Álvarez-Díaz; Diego Andrés Prada; Gerardo Santamaría; Hector Alejandro Ruiz-Moreno; Jhonnatan Reales-González; Julian Naizaque; Katherine Laiton-Donato; Magdalena Wiesner; Marcela Mercado-Reyes.; Maria T. Herrera-Sepúlveda; Martha Lucia Ospina Martínez; Sheryll Corchuelo
EPI_ISL_1220049	Laboratorio Clinico Clinica General del norte SAS	Instituto Nacional de Salud- Dirección de Investigación en Salud Pública	Carlos Franco-Muñoz; Diego A. Álvarez-Díaz; Diego Andrés Prada; Gerardo Santamaría; Hector Alejandro Ruiz-Moreno; Jhonnatan Reales-González; Julian Naizaque; Katherine Laiton-Donato; Magdalena Wiesner; Marcela Mercado-Reyes.; Maria T. Herrera-Sepúlveda; Martha Lucia Ospina Martínez; Sheryll Corchuelo
EPI_ISL_4219515, EPI_ISL_4219516, EPI_ISL_4219517, EPI_ISL_4219518, EPI_ISL_4219519, EPI_ISL_4219523, EPI_ISL_4219530, EPI_ISL_4219531, EPI_ISL_4219538, EPI_ISL_4219539, EPI_ISL_4219547, EPI_ISL_4219549			
see above	Laboratorio Clínica Colsanitas Medellín	Molecular Genetics and Antimicrobial Resistance - UGRA, Universidad El Bosque	Catalina Espitia; Jinnethe Reyes; Lorena Diaz; Marcela Mercado; Mauricio Pacheco; Rafael Rios; Valentina Martinez
EPI_ISL_2674334, EPI_ISL_2674335	Laboratorio Clínica Putumayo	Instituto Nacional de Salud- Dirección de Investigación en Salud Pública	Carlos Franco-Muñoz; Carmen Osorio; Diana Malo; Diego A. Álvarez-Díaz; Diego Andrés Prada; Gerardo Santamaría; Hector Alejandro Ruiz-Moreno; Jhonnatan Reales-González; Jorge Rivera; Juan Camilo Martinez; Julian Naizaque; Katherine Laiton-Donato; Lisseth Pardo; Magdalena Wiesner; Marcela Mercado-Reyes; Maria T. Herrera-Sepúlveda; Marta Lopez Blanco; Martha Lucia Ospina Martinez; Paola Rojas; Sergio Gomez; Sheryll Corchuelo; Ángela Alarcon Cruz
EPI_ISL_4196880, EPI_ISL_4197216, EPI_ISL_4197217, EPI_ISL_4197218, EPI_ISL_4197219, EPI_ISL_4197220, EPI_ISL_4197221, EPI_ISL_4197222, EPI_ISL_4197223, EPI_ISL_4197224, EPI_ISL_4197225, EPI_ISL_4197226, EPI_ISL_4197227, EPI_ISL_4197228, EPI_ISL_4197229, EPI_ISL_4197230, EPI_ISL_4197231, EPI_ISL_4197232, EPI_ISL_4197233, EPI_ISL_4197234, EPI_ISL_4197235, EPI_ISL_4197236, EPI_ISL_4197237, EPI_ISL_4197238, EPI_ISL_4197239, EPI_ISL_4197240, EPI_ISL_4197241, EPI_ISL_4197242, EPI_ISL_4197243, EPI_ISL_4197244, EPI_ISL_4197245, EPI_ISL_4197246, EPI_ISL_4197247, EPI_ISL_4197248, EPI_ISL_4197249, EPI_ISL_4197250, EPI_ISL_4197251, EPI_ISL_4197252, EPI_ISL_4197253, EPI_ISL_4197254, EPI_ISL_4197255, EPI_ISL_4197256, EPI_ISL_4197257, EPI_ISL_4197258, EPI_ISL_4197259, EPI_ISL_4197260, EPI_ISL_4197261, EPI_ISL_4197262			
see above	Laboratorio Clínico Analizar	Gencore - Universidad de los Andes	Cristian Barrera; David González; Gabriela Ariza; Luisa Sacristan; Marcela Guevara; Marcela Mercado; Silvia Restrepo
EPI_ISL_4207315, EPI_ISL_4207316, EPI_ISL_4207317, EPI_ISL_4207318, EPI_ISL_4207319, EPI_ISL_4207320, EPI_ISL_4207321, EPI_ISL_4207322, EPI_ISL_4207323, EPI_ISL_4207324			
see above	Laboratorio Clínico BioReferencia	Corporación para Investigaciones Biológicas-CIB	Jeanneth Mosquera Rendon; Katterine Molina Hoyos; Marcela Mercado Reyes; Uriel A. Hurtado Paez
EPI_ISL_4006785, EPI_ISL_4006786, EPI_ISL_4006787, EPI_ISL_4006788, EPI_ISL_4006789, EPI_ISL_4006790, EPI_ISL_4006791, EPI_ISL_4006792			
see above	Laboratorio Clínico Citisalud	Corporación para Investigaciones Biológicas-CIB	Jeanneth Mosquera Rendon; Katterine Molina Hoyos; Marcela Mercado Reyes; Uriel A. Hurtado Paez
EPI_ISL_3998102, EPI_ISL_3998104, EPI_ISL_3998106, EPI_ISL_3998108, EPI_ISL_3998109, EPI_ISL_3998110, EPI_ISL_3998121, EPI_ISL_3998125, EPI_ISL_3998126, EPI_ISL_3998129, EPI_ISL_3998130, EPI_ISL_3998135, EPI_ISL_3998139, EPI_ISL_3998147, EPI_ISL_3998149, EPI_ISL_3998150, EPI_ISL_3998151, EPI_ISL_3998153			
see above	Laboratorio Clínico Cristiam Gram	Laboratorio de Biologia Molecular, Universidad Cooperativa de Colombia, Santa Marta	Andrew S. Muñoz-Gamba; Daniel B. Ramírez-Osorio; Gloria A. Cuello; José A. Usme-Ciro; Katty Mindiola; Paula A. Quintero-Cortés; Yulieth L. Quiroz
EPI_ISL_4207069, EPI_ISL_4297478, EPI_ISL_4297479, EPI_ISL_4297480, EPI_ISL_4297518, EPI_ISL_4297519, EPI_ISL_4297520, EPI_ISL_4297521, EPI_ISL_4297522, EPI_ISL_4297523, EPI_ISL_4297524, EPI_ISL_4297525			
see above	Laboratorio Clínico Nancy Florez García	Centro de Genética y Biología Molecular - Universidad del Magdalena	Andrea M. Ramírez Hernandez; Angel Oviedo Marquez; Daniel Bautista; Edison Lea-Ch; Lyda R. Castro; Maria Teresa Mojica-Ortiz
EPI_ISL_4207337, EPI_ISL_4207338, EPI_ISL_4207339	Laboratorio Clínico Proanalab	Corporación para Investigaciones Biológicas-CIB	Jeanneth Mosquera Rendon; Katterine Molina Hoyos; Marcela Mercado Reyes; Uriel A. Hurtado Paez
EPI_ISL_1092007	Laboratorio Continental	Instituto Nacional de Salud- Dirección de Investigación en Salud Pública	Carlos Franco-Muñoz; Diego A. Álvarez-Díaz; Diego Andrés Prada; Gerardo Santamaría; Hector Alejandro Ruiz-Moreno; Jhonnatan Reales-González; Julian Naizaque; Katherine Laiton-Donato; Magdalena Wiesner; Marcela Mercado-Reyes; Maria T. Herrera-Sepúlveda; Martha Lucia Ospina Martinez; Sheryll Corchuelo
EPI_ISL_4297530, EPI_ISL_4297531, EPI_ISL_4297532, EPI_ISL_4297533, EPI_ISL_4297534, EPI_ISL_4297535, EPI_ISL_4297536, EPI_ISL_4297537, EPI_ISL_4297538, EPI_ISL_4297539, EPI_ISL_4297540, EPI_ISL_4297541, EPI_ISL_4297542, EPI_ISL_4297543, EPI_ISL_4297544, EPI_ISL_4297545, EPI_ISL_4297546, EPI_ISL_4297547, EPI_ISL_4297548, EPI_ISL_4297549, EPI_ISL_4297550, EPI_ISL_4297551			
see above	Laboratorio Cristian Gram	Centro de Genética y Biología Molecular - Universidad del Magdalena	Andrea M. Ramírez Hernandez; Angel Oviedo Marquez; Daniel Bautista; Edison Lea-Ch; Lyda R. Castro; Maria Teresa Mojica-Ortiz
EPI_ISL_1017702	Laboratorio Docente Asistencial-Universidad de Antioquia	Instituto Nacional de Salud- Dirección de Investigación en Salud Pública	Carlos Franco-Muñoz; Diego A. Álvarez-Díaz; Diego Andrés Prada; Gerardo Santamaría; Hector Alejandro Ruiz-Moreno; Jhonnatan Reales-González; Julian Naizaque; Katherine Laiton-Donato; Magdalena Wiesner; Marcela Mercado-Reyes; Maria T. Herrera-Sepúlveda; Martha Lucia Ospina Martinez; Sheryll Corchuelo
EPI_ISL_956301, EPI_ISL_956306	Laboratorio Gencore- Universidad de los Andes	Instituto Nacional de Salud- Dirección de Investigación en Salud Pública, Universidad de los Andes- Gencore	Carlos Franco-Muñoz; Diego A. Álvarez-Díaz; Diego Andrés Prada; Gerardo Santamaría; Hector Alejandro Ruiz-Moreno; Jhonnatan Reales-González; Julian Naizaque; Katherine Laiton-Donato; Magdalena Wiesner; Marcela Mercado-Reyes; Maria T. Herrera-Sepúlveda; Martha Lucia Ospina Martinez; Mauricio Pacheco-Montealegre; Sheryll Corchuelo
EPI_ISL_1092006	Laboratorio IMAT	Instituto Nacional de Salud- Dirección de Investigación en Salud Pública	Carlos Franco-Muñoz; Diego A. Álvarez-Díaz; Diego Andrés Prada; Gerardo Santamaría; Hector Alejandro Ruiz-Moreno; Jhonnatan Reales-González; Julian Naizaque; Katherine Laiton-Donato; Magdalena Wiesner; Marcela Mercado-Reyes; Maria T. Herrera-Sepúlveda; Martha Lucia Ospina Martinez; Sheryll Corchuelo
EPI_ISL_4219520, EPI_ISL_4219521, EPI_ISL_4219522, EPI_ISL_4219524, EPI_ISL_4219525, EPI_ISL_4219526, EPI_ISL_4219527, EPI_ISL_4219528, EPI_ISL_4219533, EPI_ISL_4219534, EPI_ISL_4219535, EPI_ISL_4219536, EPI_ISL_4219537, EPI_ISL_4219540, EPI_ISL_4219541, EPI_ISL_4219542, EPI_ISL_4219543, EPI_ISL_4219544, EPI_ISL_4219545, EPI_ISL_4219546, EPI_ISL_4219548			
see above	Laboratorio IMAT Instituto médico de alta tecnología S.A.S	Molecular Genetics and Antimicrobial Resistance - UGRA, Universidad El Bosque	Catalina Espitia; Jinnethe Reyes; Lorena Diaz; Marcela Mercado; Mauricio Pacheco; Rafael Rios; Valentina Martinez
EPI_ISL_3320719, EPI_ISL_3320724, EPI_ISL_3320729, EPI_ISL_3320746, EPI_ISL_3320750	Laboratorio IMAT SAS	Instituto Nacional de Salud	Carlos Franco-Muñoz; Carmen Osorio; Diana Malo; Diego A. Álvarez-Díaz; Diego Andrés Prada; Gerardo Santamaría; Hector Alejandro Ruiz-Moreno; Jhonnatan Reales-González; Jorge Rivera; Juan Camilo Martinez; Julian Naizaque; Katherine Laiton-Donato; Lisseth Pardo; Magdalena Wiesner; Marcela Mercado-Reyes; Maria T. Herrera-Sepúlveda; Marta Lopez Blanco; Martha Lucia Ospina Martinez; Paola Rojas; Sergio Gomez; Sheryll Corchuelo; Ángela Alarcon Cruz
EPI_ISL_3671002, EPI_ISL_3671008, EPI_ISL_3671038, EPI_ISL_3671041, EPI_ISL_3671049, EPI_ISL_3671051, EPI_ISL_3671061			
see above	Laboratorio Las Americas	Universidad Nacional de Colombia - Laboratorio Genómico One Health	Andres F. Cardona-Rios; Carlos Franco-Muñoz; Carolina Muñoz-Arango; Celeny Ortiz; Daniel O. Maldonado-Perez; Diego A. Álvarez-Díaz; Hector Alejandro Ruiz-Moreno; Idabely Betancur Ortiz; Jorge E. Osorio; Juan P. Hernandez-Ortiz; Karl A Ciuoderis; Katherine Laiton-Donato; Laura Silvana Perez; Lina M. Hurtado; Marcela Mercado-Reyes; Maria Angélica Maya; Maria Stella López; Rita Almanza Payares; Sandra Ines Cano; Simón Villegas Velásquez
EPI_ISL_4006793, EPI_ISL_4006794, EPI_ISL_4006795, EPI_ISL_4006796, EPI_ISL_4006797, EPI_ISL_4006798, EPI_ISL_4006799, EPI_ISL_4207304, EPI_ISL_4207305, EPI_ISL_4207306, EPI_ISL_4207307, EPI_ISL_4207308, EPI_ISL_4207309, EPI_ISL_4207310, EPI_ISL_4207311, EPI_ISL_4207312, EPI_ISL_4207313, EPI_ISL_4207314, EPI_ISL_4207335, EPI_ISL_4207336			
see above	Laboratorio Las Américas	Corporación para Investigaciones Biológicas-CIB	Jeanneth Mosquera Rendon; Katterine Molina Hoyos; Marcela Mercado Reyes; Uriel A. Hurtado Paez

EPI_ISL_3998113, EPI_ISL_3998114, EPI_ISL_3998131, EPI_ISL_3998134, EPI_ISL_3998137, EPI_ISL_3998138, EPI_ISL_3998142, EPI_ISL_3998143, EPI_ISL_3998158			
see above	Laboratorio Lorena Vejarano sede Barranquilla	Laboratorio de Biologia Molecular, Universidad Cooperativa de Colombia, Santa Marta	Andrew S. Muñoz-Gamba; Daniel B. Ramírez-Osorio; Danis Lora; José A. Usme-Ciro; Lissette M. Lopez; Paula A. Quintero-Cortés
EPI_ISL_4297481, EPI_ISL_4297482, EPI_ISL_4297483, EPI_ISL_4297484, EPI_ISL_4297485, EPI_ISL_4297486, EPI_ISL_4297487, EPI_ISL_4297495, EPI_ISL_4297496, EPI_ISL_4297497, EPI_ISL_4297498, EPI_ISL_4297499, EPI_ISL_4297500, EPI_ISL_4297501, EPI_ISL_4297502			
see above	Laboratorio Masvida de la Costa	Centro de Genética y Biología Molecular - Universidad del Magdalena	Andrea M. Ramírez Hernandez; Angel Oviedo Marquez; Daniel Bautista; Edison Lea-Ch; Lyda R. Castro; Maria Teresa Mojica-Ortiz
EPI_ISL_845628, EPI_ISL_845629	Laboratorio Médico Echavarria	Instituto Nacional de Salud - Dirección de Investigación en Salud Pública	Carlos Franco-Muñoz; Diego A. Álvarez-Díaz; Diego Andrés Prada; Gerardo Santamaría; Jonathan Reales; Julian Naizaque; Katherine Laiton-Donato; Magdalena Wiesner; Marcela Mercado-Reyes; Maria T. Herrera-Sepúlveda; Martha Lucia Ospina Martinez; Mauricio Pacheco-Montealegre; Paola Muñoz-Laiton; Sheryll Corchuelo
EPI_ISL_3278303, EPI_ISL_3320733, EPI_ISL_3320738, EPI_ISL_3320742, EPI_ISL_3320747, EPI_ISL_3320751	Laboratorio Olimpus	Instituto Nacional de Salud	Carlos Franco-Muñoz; Carmen Osorio; Diana Malo; Diego A. Álvarez-Díaz; Diego Andrés Prada; Gerardo Santamaría; Hector Alejandro Ruiz-Moreno; Jhonnatan Reales-González; Jorge Rivera; Juan Camilo Martinez; Julian Naizaque; Katherine Laiton-Donato; Lisseth Pardo; Magdalena Wiesner; Marcela Mercado-Reyes; Maria T. Herrera-Sepúlveda; Marta Lopez Blanco; Martha Lucia Ospina Martinez; Paola Rojas; Sergio Gomez; Sheryll Corchuelo; Ángela Alarcon Cruz
EPI_ISL_3320740	Laboratorio Previs IPS	Instituto Nacional de Salud	Carlos Franco-Muñoz; Carmen Osorio; Diana Malo; Diego A. Álvarez-Díaz; Diego Andrés Prada; Gerardo Santamaría; Hector Alejandro Ruiz-Moreno; Jhonnatan Reales-González; Jorge Rivera; Juan Camilo Martinez; Julian Naizaque; Katherine Laiton-Donato; Lisseth Pardo; Magdalena Wiesner; Marcela Mercado-Reyes; Maria T. Herrera-Sepúlveda; Marta Lopez Blanco; Martha Lucia Ospina Martinez; Paola Rojas; Sergio Gomez; Sheryll Corchuelo; Ángela Alarcon Cruz
EPI_ISL_4207325, EPI_ISL_4207326, EPI_ISL_4207327, EPI_ISL_4207328, EPI_ISL_4207329, EPI_ISL_4207330, EPI_ISL_4207331, EPI_ISL_4207332			
see above	Laboratorio Prime Diagnostics	Corporación para Investigaciones Biológicas-CIB	Jeanneth Mosquera Rendon; Katterine Molina Hoyos; Marcela Mercado Reyes; Uriel A. Hurtado Paez
EPI_ISL_4297503, EPI_ISL_4297504, EPI_ISL_4297505, EPI_ISL_4297506, EPI_ISL_4297507, EPI_ISL_4297508, EPI_ISL_4297509			
see above	Laboratorio Rey-Fals	Centro de Genética y Biología Molecular - Universidad del Magdalena	Andrea M. Ramírez Hernandez; Angel Oviedo Marquez; Daniel Bautista; Edison Lea-Ch; Lyda R. Castro; Maria Teresa Mojica-Ortiz
EPI_ISL_3307442, EPI_ISL_3307443, EPI_ISL_3307444, EPI_ISL_3307445, EPI_ISL_3307446, EPI_ISL_3307447, EPI_ISL_3307448, EPI_ISL_3307449, EPI_ISL_3307450, EPI_ISL_3307451, EPI_ISL_3307452, EPI_ISL_3307453, EPI_ISL_3307454, EPI_ISL_3307455, EPI_ISL_3307456, EPI_ISL_3307457, EPI_ISL_3307458, EPI_ISL_3332684, EPI_ISL_3332685, EPI_ISL_3332686, EPI_ISL_3332687, EPI_ISL_3332688, EPI_ISL_3332689, EPI_ISL_3332690, EPI_ISL_3332691, EPI_ISL_3332692, EPI_ISL_3332693, EPI_ISL_3332694, EPI_ISL_3332695, EPI_ISL_3332696, EPI_ISL_3332698, EPI_ISL_3332699			
see above	Laboratorio SYNLAB Colombia	Corporación para Investigaciones Biológicas-CIB	Jeanneth Mosquera Rendon; Jenny Santiago Cuesta; Marcela Mercado Reyes; Uriel A. Hurtado Paez
EPI_ISL_2674355, EPI_ISL_2674358, EPI_ISL_2674359, EPI_ISL_2674360, EPI_ISL_2674362, EPI_ISL_2674366, EPI_ISL_2674367			
see above	Laboratorio Salud Publica San Andres Islas	Instituto Nacional de Salud- Dirección de Investigación en Salud Pública	Carlos Franco-Muñoz; Carmen Osorio; Diana Malo; Diego A. Álvarez-Díaz; Diego Andrés Prada; Gerardo Santamaría; Hector Alejandro Ruiz-Moreno; Jhonnatan Reales-González; Jorge Rivera; Juan Camilo Martinez; Julian Naizaque; Katherine Laiton-Donato; Lisseth Pardo; Magdalena Wiesner; Marcela Mercado-Reyes; Maria T. Herrera-Sepúlveda; Marta Lopez Blanco; Martha Lucia Ospina Martinez; Paola Rojas; Sergio Gomez; Sheryll Corchuelo; Ángela Alarcon Cruz
EPI_ISL_2674363	Laboratorio Salud Publica- San Andres Islas	Instituto Nacional de Salud- Dirección de Investigación en Salud Pública	Carlos Franco-Muñoz; Carmen Osorio; Diana Malo; Diego A. Álvarez-Díaz; Diego Andrés Prada; Gerardo Santamaría; Hector Alejandro Ruiz-Moreno; Jhonnatan Reales-González; Jorge Rivera; Juan Camilo Martinez; Julian Naizaque; Katherine Laiton-Donato; Lisseth Pardo; Magdalena Wiesner; Marcela Mercado-Reyes; Maria T. Herrera-Sepúlveda; Marta Lopez Blanco; Martha Lucia Ospina Martinez; Paola Rojas; Sergio Gomez; Sheryll Corchuelo; Ángela Alarcon Cruz
EPI_ISL_3671003, EPI_ISL_3671019, EPI_ISL_3671036, EPI_ISL_3671039	Laboratorio UNIGEM	Universidad Nacional de Colombia - Laboratorio Genómico One Health	Andres F. Cardona-Rios; Carlos Franco-Muñoz; Carolina Muñoz-Arango; Celeny Ortiz; Daniel O. Maldonado-Perez; Diego A. Álvarez-Díaz; Hector Alejandro Ruiz-Moreno; Idabely Betancur Ortiz; Jorge E. Osorio; Juan P. Hernandez-Ortiz; Karl A Ciuoderis; Katherine Laiton-Donato; Laura Silvana Perez; Lina M. Hurtado; Marcela Mercado-Reyes; Maria Angélica Maya; Maria Stella López; Rita Almanza Payares; Sandra Ines Cano; Simón Villegas Velásquez
EPI_ISL_3671006, EPI_ISL_3671016, EPI_ISL_3671040, EPI_ISL_3671042, EPI_ISL_3671053, EPI_ISL_3671063, EPI_ISL_3671064, EPI_ISL_3721596			
see above	Laboratorio Yamina Cumplido	Universidad Nacional de Colombia - Laboratorio Genómico One Health	Andres F. Cardona-Rios; Carlos Franco-Muñoz; Carolina Muñoz-Arango; Celeny Ortiz; Daniel O. Maldonado-Perez; Diego A. Álvarez-Díaz; Hector Alejandro Ruiz-Moreno; Idabely Betancur Ortiz; Jorge E. Osorio; Juan P. Hernandez-Ortiz; Karl A Ciuoderis; Katherine Laiton-Donato; Laura Silvana Perez; Lina M. Hurtado; Marcela Mercado-Reyes; Maria Angélica Maya; Maria Stella López; Rita Almanza Payares; Sandra Ines Cano; Simón Villegas Velásquez
EPI_ISL_3342899, EPI_ISL_3376685, EPI_ISL_3376686, EPI_ISL_3376687, EPI_ISL_3376688, EPI_ISL_3376689, EPI_ISL_3376690, EPI_ISL_3376691, EPI_ISL_3376692, EPI_ISL_3376693, EPI_ISL_3376694, EPI_ISL_3376695, EPI_ISL_3376696, EPI_ISL_3376697, EPI_ISL_3376698, EPI_ISL_3376699, EPI_ISL_3376700, EPI_ISL_3376701, EPI_ISL_3376702, EPI_ISL_3376703, EPI_ISL_3376704, EPI_ISL_3376705, EPI_ISL_3376706, EPI_ISL_3376707, EPI_ISL_3376708, EPI_ISL_3376709, EPI_ISL_3376710, EPI_ISL_3376711, EPI_ISL_3376712, EPI_ISL_3376713, EPI_ISL_3376714, EPI_ISL_3376715, EPI_ISL_3376716, EPI_ISL_3376717, EPI_ISL_3376718, EPI_ISL_3376719, EPI_ISL_3376720, EPI_ISL_3376721, EPI_ISL_3376722, EPI_ISL_3376723, EPI_ISL_3376724, EPI_ISL_3376725, EPI_ISL_3376726, EPI_ISL_3376727, EPI_ISL_3376728, EPI_ISL_3376729, EPI_ISL_3376730, EPI_ISL_4297488, EPI_ISL_4297489, EPI_ISL_4297490, EPI_ISL_4297491, EPI_ISL_4297492, EPI_ISL_4297493, EPI_ISL_4297494			
see above	Laboratorio de Biología Molecular - Universidad del Magdalena	Centro de Genética y Biología Molecular - Universidad del Magdalena	Andrea M. Ramírez Hernandez; Angel Oviedo Marquez; Daniel Bautista; Edison Lea-Ch; Lyda R. Castro; Maria Teresa Mojica-Ortiz
EPI_ISL_1582984, EPI_ISL_1582985, EPI_ISL_1582986, EPI_ISL_1582988, EPI_ISL_1582989, EPI_ISL_1582990	Laboratorio de Biología Molecular – Universidad del Magdalena	Instituto Nacional de Salud- Dirección de Investigación en Salud Pública	Carlos Franco-Muñoz; Carmen Osorio; Diana Malo; Diego A. Álvarez-Díaz; Diego Andrés Prada; Gerardo Santamaría; Hector Alejandro Ruiz-Moreno; Jhonnatan Reales-González; Juan Camilo Martinez; Julian Naizaque; Katherine Laiton-Donato; Lisseth Pardo; Magdalena Wiesner; Marcela Mercado-Reyes; Maria T. Herrera-Sepúlveda; Marta Lopez Blanco; Martha Lucia Ospina Martinez; Paola Rojas; Sergio Gomez; Sheryll Corchuelo; Ángela Alarcon Cruz
EPI_ISL_2009062, EPI_ISL_2009067, EPI_ISL_2009068, EPI_ISL_2876096	Laboratorio de Especialidades Clinizad	Instituto Nacional de Salud- Dirección de Investigación en Salud Pública	Carlos Franco-Muñoz; Carmen Osorio; Diana Malo; Diego A. Álvarez-Díaz; Diego Andrés Prada; Gerardo Santamaría; Hector Alejandro Ruiz-Moreno; Jhonnatan Reales-González; Jorge Rivera; Juan Camilo Martinez; Julian Naizaque; Katherine Laiton-Donato; Lisseth Pardo; Magdalena Wiesner; Marcela Mercado-Reyes; Maria T. Herrera-Sepúlveda; Marta Lopez Blanco; Martha Lucia Ospina Martinez; Paola Rojas; Sergio Gomez; Sheryll Corchuelo; Ángela Alarcon Cruz
EPI_ISL_2674329, EPI_ISL_2674330, EPI_ISL_2674331, EPI_ISL_2674332, EPI_ISL_2674333	Laboratorio de Inmunología y Biología Molecular	Instituto Nacional de Salud- Dirección de Investigación en Salud Pública	Carlos Franco-Muñoz; Carmen Osorio; Diana Malo; Diego A. Álvarez-Díaz; Diego Andrés Prada; Gerardo Santamaría; Hector Alejandro Ruiz-Moreno; Jhonnatan Reales-González; Jorge Rivera; Juan Camilo Martinez; Julian Naizaque; Katherine Laiton-Donato; Lisseth Pardo; Magdalena Wiesner; Marcela Mercado-Reyes; Maria T. Herrera-Sepúlveda; Marta Lopez Blanco; Martha Lucia Ospina Martinez; Paola Rojas; Sergio Gomez; Sheryll Corchuelo; Ángela Alarcon Cruz
EPI_ISL_2674364, EPI_ISL_2674365	Laboratorio de Salud Publica	Instituto Nacional de Salud- Dirección de Investigación en Salud Pública	Carlos Franco-Muñoz; Carmen Osorio; Diana Malo; Diego A. Álvarez-Díaz; Diego Andrés Prada; Gerardo Santamaría; Hector Alejandro Ruiz-Moreno; Jhonnatan Reales-González; Jorge Rivera; Juan Camilo Martinez; Julian Naizaque; Katherine Laiton-Donato; Lisseth Pardo; Magdalena Wiesner; Marcela Mercado-Reyes; Maria T. Herrera-Sepúlveda; Marta Lopez Blanco; Martha Lucia Ospina Martinez; Paola Rojas; Sergio Gomez; Sheryll Corchuelo; Ángela Alarcon Cruz
EPI_ISL_1494941, EPI_ISL_1494942, EPI_ISL_1494943	Laboratorio de Salud Publica Arauca	Instituto Nacional de Salud- Dirección de Investigación en Salud Pública	Carlos Franco-Muñoz; Carmen Osorio; Diana Malo; Diego A. Álvarez-Díaz; Diego Andrés Prada; Gerardo Santamaría; Hector Alejandro Ruiz-Moreno; Jhonnatan Reales-González; Juan Camilo Martinez; Julian Naizaque; Katherine Laiton-Donato; Lisseth Pardo; Magdalena Wiesner; Marcela Mercado-Reyes; Maria T. Herrera-Sepúlveda; Marta Lopez Blanco; Martha Lucia Ospina Martinez; Paola Rojas; Sergio Gomez; Sheryll Corchuelo; Ángela Alarcon Cruz
EPI_ISL_2674353, EPI_ISL_2674354, EPI_ISL_2674361	Laboratorio de Salud Publica San Andrés Islas	Instituto Nacional de Salud- Dirección de Investigación en Salud Pública	Carlos Franco-Muñoz; Carmen Osorio; Diana Malo; Diego A. Álvarez-Díaz; Diego Andrés Prada; Gerardo Santamaría; Hector Alejandro Ruiz-Moreno; Jhonnatan Reales-González; Jorge Rivera; Juan Camilo Martinez; Julian Naizaque; Katherine Laiton-Donato; Lisseth Pardo; Magdalena Wiesner; Marcela Mercado-Reyes; Maria T. Herrera-Sepúlveda; Marta Lopez Blanco; Martha Lucia Ospina Martinez; Paola Rojas; Sergio Gomez; Sheryll Corchuelo; Ángela Alarcon Cruz
EPI_ISL_906144, EPI_ISL_906145, EPI_ISL_906534, EPI_ISL_906557, EPI_ISL_906558, EPI_ISL_906559, EPI_ISL_906560, EPI_ISL_906561			
see above	Laboratorio de Salud Publica de Amazonas	Instituto Nacional de Salud- Dirección de Investigación en Salud Pública, Universidad de los Andes- Applied genomics research group, Vicerrectoria de Investigación y Creación, Universidad de los Andes- Systems and Computing Engineering Department	Carlos Franco-Muñoz; Diego A. Álvarez-Díaz; Diego Andrés Prada; Gerardo Santamaría Jorge Duitama; Héctor Alejandro Ruiz-Moreno; Jhonnatan Reales-González; Jorge Ivan Diaz; Julian Naizaque; Katherine Laiton-Donato; Laura Natalia Gonzalez; Magdalena Wiesner; Marcela Mercado-Reyes; Maria T. Herrera-Sepúlveda; Martha Lucia Ospina Martinez; Mauricio Pacheco-Montealegre; Sheryll Corchuelo; Silvia Restrepo-Restrepo
EPI_ISL_1092008	Laboratorio de Salud Publica de Bogota	Instituto Nacional de Salud- Dirección de Investigación en Salud Pública	Carlos Franco-Muñoz; Diego A. Álvarez-Díaz; Diego Andrés Prada; Gerardo Santamaría; Hector Alejandro Ruiz-Moreno; Jhonnatan Reales-González; Julian Naizaque; Katherine Laiton-Donato; Magdalena Wiesner; Marcela Mercado-Reyes; Maria T. Herrera-Sepúlveda; Martha Lucia Ospina Martinez; Sheryll Corchuelo
EPI_ISL_1137615, EPI_ISL_1137619	Laboratorio de Salud Publica de Cauca	Instituto Nacional de Salud- Dirección de Investigación en Salud Pública	Carlos Franco-Muñoz; Diego A. Álvarez-Díaz; Diego Andrés Prada; Gerardo Santamaría; Hector Alejandro Ruiz-Moreno; Jhonnatan Reales-González; Julian Naizaque; Katherine Laiton-Donato; Magdalena Wiesner; Marcela Mercado-Reyes.; Maria T. Herrera-Sepúlveda; Martha Lucia Ospina Martínez; Sheryll Corchuelo
EPI_ISL_1092005	Laboratorio de Salud Publica de Cesar	Instituto Nacional de Salud- Dirección de Investigación en Salud Pública	Carlos Franco-Muñoz; Diego A. Álvarez-Díaz; Diego Andrés Prada; Gerardo Santamaría; Hector Alejandro Ruiz-Moreno; Jhonnatan Reales-González; Julian Naizaque; Katherine Laiton-Donato; Magdalena Wiesner; Marcela Mercado-Reyes; Maria T. Herrera-Sepúlveda; Martha Lucia Ospina Martinez; Sheryll Corchuelo
EPI_ISL_4196881	Laboratorio de Salud Publica de Cundinamarca	Gencore - Universidad de los Andes	Cristian Barrera; Gabriela Ariza; Luisa Sacristan; Marcela Guevara; Marcela Mercado; Silvia Restrepo
EPI_ISL_1494950	Laboratorio de Salud Publica de Santander	Instituto Nacional de Salud- Dirección de Investigación en Salud Pública	Carlos Franco-Muñoz; Carmen Osorio; Diana Malo; Diego A. Álvarez-Díaz; Diego Andrés Prada; Gerardo Santamaría; Hector Alejandro Ruiz-Moreno; Jhonnatan Reales-González; Juan Camilo Martinez; Julian Naizaque; Katherine Laiton-Donato; Lisseth Pardo; Magdalena Wiesner; Marcela Mercado-Reyes; Maria T. Herrera-Sepúlveda; Marta Lopez Blanco; Martha Lucia Ospina Martinez; Paola Rojas; Sergio Gomez; Sheryll Corchuelo; Ángela Alarcon Cruz
EPI_ISL_2157202, EPI_ISL_2157203, EPI_ISL_2157204, EPI_ISL_2157205, EPI_ISL_2157206, EPI_ISL_2157207, EPI_ISL_2157208, EPI_ISL_2157209, EPI_ISL_2157210, EPI_ISL_2157213			
see above	Laboratorio de Salud Pública	Gencore - Universidad de los Andes	Alejandro Gomez; David Gonzalez; Gabriela Delgado; Johana Hernandez; Luisa Sacristan; Marcela Guevara; Silvia Restrepo
EPI_ISL_1235686	Laboratorio de Salud Pública (Bogota)	Gencore- Universidad de los Andes	Alejandro Gómez; David González; Johana Hernandez Gabriela Delgado; Luisa Sacristán; Marcela Guevara-Suarez; Silvia Restrepo
EPI_ISL_845635, EPI_ISL_845636, EPI_ISL_845637, EPI_ISL_845638, EPI_ISL_845639, EPI_ISL_845640, EPI_ISL_845641, EPI_ISL_845642, EPI_ISL_845643, EPI_ISL_845644, EPI_ISL_845645, EPI_ISL_845646, EPI_ISL_845647, EPI_ISL_845648, EPI_ISL_845649, EPI_ISL_845650, EPI_ISL_845651, EPI_ISL_845652, EPI_ISL_845653, EPI_ISL_845654, EPI_ISL_845655			
see above	Laboratorio de Salud Pública - Secretaría Distrital de Salud	Instituto Nacional de Salud - Dirección de Investigación en Salud Pública	Carlos Franco-Muñoz; Diego A. Álvarez-Díaz; Diego Andrés Prada; Gerardo Santamaría; Jonathan Reales; Julian Naizaque; Katherine Laiton-Donato; Magdalena Wiesner; Marcela Mercado-Reyes; Maria T. Herrera-Sepúlveda; Martha Lucia Ospina Martinez; Mauricio Pacheco-Montealegre; Paola Muñoz-Laiton; Sheryll Corchuelo
EPI_ISL_1137478, EPI_ISL_1303378, EPI_ISL_1303379, EPI_ISL_1303380, EPI_ISL_1494959	Laboratorio de Salud Pública - Secretaría Distrital de Salud	Instituto Nacional de Salud- Dirección de Investigación en Salud Pública	Carlos Franco-Muñoz; Carmen Osorio; Diana Malo; Diego A. Álvarez-Díaz; Diego Andrés Prada; Gerardo Santamaría; Hector Alejandro Ruiz-Moreno; Jhonnatan Reales-González; Juan Camilo Martinez; Julian Naizaque; Katherine Laiton-Donato; Lisseth Pardo; Magdalena Wiesner; Marcela Mercado-Reyes; Marcela Mercado-Reyes.; Maria T. Herrera-Sepúlveda; Marta Lopez Blanco; Martha Lucia Ospina Martinez; Martha Lucia Ospina Martínez; Paola Rojas; Sergio Gomez; Sheryll Corchuelo; Ángela Alarcon Cruz
EPI_ISL_1626617, EPI_ISL_1626618, EPI_ISL_1626619, EPI_ISL_1626620, EPI_ISL_1626621, EPI_ISL_1738781, EPI_ISL_1738782, EPI_ISL_1738783, EPI_ISL_1738784, EPI_ISL_1738785, EPI_ISL_1738786, EPI_ISL_1738787, EPI_ISL_1738788, EPI_ISL_1738789, EPI_ISL_1738790			
see above	Laboratorio de Salud Pública Bogota	Gencore - Universidad de los Andes	Alejandro Gomez; Ana Maria Palacio; David Gonzalez; Gabriela Delgado; Johana Hernandez; Luisa Sacristan; Marcela Guevara; Silvia Restrepo
EPI_ISL_2657859, EPI_ISL_2657860, EPI_ISL_2657861, EPI_ISL_2657862, EPI_ISL_2657863, EPI_ISL_2657864	Laboratorio de Salud Pública Fronterizo de Arauca	Instituto Nacional de Salud- Dirección de Investigación en Salud Pública	Carlos Franco-Muñoz; Carmen Osorio; Diana Malo; Diego A. Álvarez-Díaz; Diego Andrés Prada; Gerardo Santamaría; Hector Alejandro Ruiz-Moreno; Jhonnatan Reales-González; Jorge Rivera; Juan Camilo Martinez; Julian Naizaque; Katherine Laiton-Donato; Lisseth Pardo; Magdalena Wiesner; Marcela Mercado-Reyes; Maria T. Herrera-Sepúlveda; Marta Lopez Blanco; Martha Lucia Ospina Martinez; Paola Rojas; Sergio Gomez; Sheryll Corchuelo; Ángela Alarcon Cruz
EPI_ISL_906548	Laboratorio de Salud Pública Fronterizo de Arauca	Instituto Nacional de Salud- Dirección de Investigación en Salud Pública, Universidad de los Andes- Applied genomics research group, Vicerrectoria de Investigación y Creación, Universidad de los Andes- Systems and Computing Engineering Department	Carlos Franco-Muñoz; Diego A. Álvarez-Díaz; Diego Andrés Prada; Gerardo Santamaría Jorge Duitama; Héctor Alejandro Ruiz-Moreno; Jhonnatan Reales-González; Jorge Ivan Diaz; Julian Naizaque; Katherine Laiton-Donato; Laura Natalia Gonzalez; Magdalena Wiesner; Marcela Mercado-Reyes; Maria T. Herrera-Sepúlveda; Martha Lucia Ospina Martinez; Mauricio Pacheco-Montealegre; Sheryll Corchuelo; Silvia Restrepo-Restrepo
EPI_ISL_1582995	Laboratorio de Salud Pública de Amazonas	Instituto Nacional de Salud- Dirección de Investigación en Salud Pública	Carlos Franco-Muñoz; Carmen Osorio; Diana Malo; Diego A. Álvarez-Díaz; Diego Andrés Prada; Gerardo Santamaría; Hector Alejandro Ruiz-Moreno; Jhonnatan Reales-González; Juan Camilo Martinez; Julian Naizaque; Katherine Laiton-Donato; Lisseth Pardo; Magdalena Wiesner; Marcela Mercado-Reyes; Maria T. Herrera-Sepúlveda; Marta Lopez Blanco; Martha Lucia Ospina Martinez; Paola Rojas; Sergio Gomez; Sheryll Corchuelo; Ángela Alarcon Cruz

EPI_ISL_1017703	Laboratorio de Salud Pública de Bogota	Instituto Nacional de Salud- Dirección de Investigación en Salud Pública	Carlos Franco-Muñoz; Diego A. Álvarez-Díaz; Diego Andrés Prada; Gerardo Santamaría; Hector Alejandro Ruiz-Moreno; Jhonnatan Reales-González; Julian Naizaque; Katherine Laiton-Donato; Magdalena Wiesner; Marcela Mercado-Reyes; Maria T. Herrera-Sepúlveda; Martha Lucia Ospina Martinez; Sheryll Corchuelo
EPI_ISL_1036756	Laboratorio de Salud Pública de Bogotá	Instituto Nacional de Salud- Direccion de Investigacion en Salud Publica	Carlos Franco-Muñoz; Diego A. Álvarez-Díaz; Diego Andrés Prada; Gerardo Santamaría; Hector Alejandro Ruiz-Moreno; Jhonnatan Reales-González; Julian Naizaque; Katherine Laiton-Donato; Magdalena Wiesner; Marcela Mercado-Reyes; Maria T. Herrera-Sepúlveda; Martha Lucia Ospina Martinez; Sheryll Corchuelo
EPI_ISL_3673674, EPI_ISL_3673675	Laboratorio de Salud Pública de Bogotá	Laboratorio de Salud Pública de Bogotá	Alejandro Gomez; Cristian Barrera; Gabriela Ariza; Gabriela Delgado; Johana Hernandez; Luisa Sacristan; Marcela Guevara; Silvia Restrepo
EPI_ISL_1017701	Laboratorio de Salud Pública de Boyacá	Instituto Nacional de Salud- Dirección de Investigación en Salud Pública	Carlos Franco-Muñoz; Diego A. Álvarez-Díaz; Diego Andrés Prada; Gerardo Santamaría; Hector Alejandro Ruiz-Moreno; Jhonnatan Reales-González; Julian Naizaque; Katherine Laiton-Donato; Magdalena Wiesner; Marcela Mercado-Reyes; Maria T. Herrera-Sepúlveda; Martha Lucia Ospina Martinez; Sheryll Corchuelo
EPI_ISL_906533, EPI_ISL_906538, EPI_ISL_906542, EPI_ISL_906549	Laboratorio de Virologia-Instituto Nacional de Salud	Instituto Nacional de Salud- Dirección de Investigación en Salud Pública, Universidad de los Andes- Applied genomics research group, Vicerrectoria de Investigación y Creación, Universidad de los Andes- Systems and Computing Engineering Department	Carlos Franco-Muñoz; Diego A. Álvarez-Díaz; Diego Andrés Prada; Gerardo Santamaría Jorge Duitama; Héctor Alejandro Ruiz-Moreno; Jhonnatan Reales-González; Jorge Ivan Diaz; Julian Naizaque; Katherine Laiton-Donato; Laura Natalia Gonzalez; Magdalena Wiesner; Marcela Mercado-Reyes; Maria T. Herrera-Sepúlveda; Martha Lucia Ospina Martinez; Mauricio Pacheco-Montealegre; Sheryll Corchuelo; Silvia Restrepo-Restrepo
EPI_ISL_1220054	Laboratorio de biología molecular diagnóstico de COVID-19 U Caldas	Instituto Nacional de Salud- Dirección de Investigación en Salud Pública	Carlos Franco-Muñoz; Diego A. Álvarez-Díaz; Diego Andrés Prada; Gerardo Santamaría; Hector Alejandro Ruiz-Moreno; Jhonnatan Reales-González; Julian Naizaque; Katherine Laiton-Donato; Magdalena Wiesner; Marcela Mercado-Reyes.; Maria T. Herrera-Sepúlveda; Martha Lucia Ospina Martínez; Sheryll Corchuelo
EPI_ISL_4196918, EPI_ISL_4196919, EPI_ISL_4196920, EPI_ISL_4197195, EPI_ISL_4197196, EPI_ISL_4197197, EPI_ISL_4197198, EPI_ISL_4197199, EPI_ISL_4197200, EPI_ISL_4197201, EPI_ISL_4197202, EPI_ISL_4197203, EPI_ISL_4197204, EPI_ISL_4197205, EPI_ISL_4197206, EPI_ISL_4197207, EPI_ISL_4197208, EPI_ISL_4197209, EPI_ISL_4197210, EPI_ISL_4197211, EPI_ISL_4197212, EPI_ISL_4197213, EPI_ISL_4197214, EPI_ISL_4197215, EPI_ISL_4212167, EPI_ISL_4212168, EPI_ISL_4212169, EPI_ISL_4212170, EPI_ISL_4212171, EPI_ISL_4212172, EPI_ISL_4212173, EPI_ISL_4212174, EPI_ISL_4212175, EPI_ISL_4212176, EPI_ISL_4212177			
see above	Laboratorio de la Clínica Los Nogales	Gencore - Universidad de los Andes	Cristian Barrera; David González; Gabriela Ariza; Luisa Sacristan; Marcela Guevara; Marcela Mercado; Silvia Restrepo
EPI_ISL_956299, EPI_ISL_956300	Laboratorio de salud publica Arauca	Instituto Nacional de Salud- Dirección de Investigación en Salud Pública	Carlos Franco-Muñoz; Diego A. Álvarez-Díaz; Diego Andrés Prada; Gerardo Santamaría; Hector Alejandro Ruiz-Moreno; Jhonnatan Reales-González; Julian Naizaque; Katherine Laiton-Donato; Magdalena Wiesner; Marcela Mercado-Reyes; Maria T. Herrera-Sepúlveda; Martha Lucia Ospina Martinez; Mauricio Pacheco-Montealegre; Sheryll Corchuelo
EPI_ISL_906530	Laboratorio de salud publica de Bogota	Instituto Nacional de Salud- Dirección de Investigación en Salud Pública, Universidad de los Andes- Applied genomics research group, Vicerrectoria de Investigación y Creación, Universidad de los Andes- Systems and Computing Engineering Department	Carlos Franco-Muñoz; Diego A. Álvarez-Díaz; Diego Andrés Prada; Gerardo Santamaría Jorge Duitama; Héctor Alejandro Ruiz-Moreno; Jhonnatan Reales-González; Jorge Ivan Diaz; Julian Naizaque; Katherine Laiton-Donato; Laura Natalia Gonzalez; Magdalena Wiesner; Marcela Mercado-Reyes; Maria T. Herrera-Sepúlveda; Martha Lucia Ospina Martinez; Mauricio Pacheco-Montealegre; Sheryll Corchuelo; Silvia Restrepo-Restrepo
EPI_ISL_2674357	Laboratorio de salud publica de san andres	Instituto Nacional de Salud- Dirección de Investigación en Salud Pública	Carlos Franco-Muñoz; Carmen Osorio; Diana Malo; Diego A. Álvarez-Díaz; Diego Andrés Prada; Gerardo Santamaría; Hector Alejandro Ruiz-Moreno; Jhonnatan Reales-González; Jorge Rivera; Juan Camilo Martinez; Julian Naizaque; Katherine Laiton-Donato; Lisseth Pardo; Magdalena Wiesner; Marcela Mercado-Reyes; Maria T. Herrera-Sepúlveda; Marta Lopez Blanco; Martha Lucia Ospina Martinez; Paola Rojas; Sergio Gomez; Sheryll Corchuelo; Ángela Alarcon Cruz
EPI_ISL_956298	Laboratorio de salud publica del Valle del Cauca	Instituto Nacional de Salud- Dirección de Investigación en Salud Pública	Carlos Franco-Muñoz; Diego A. Álvarez-Díaz; Diego Andrés Prada; Gerardo Santamaría; Hector Alejandro Ruiz-Moreno; Jhonnatan Reales-González; Julian Naizaque; Katherine Laiton-Donato; Magdalena Wiesner; Marcela Mercado-Reyes; Maria T. Herrera-Sepúlveda; Martha Lucia Ospina Martinez; Mauricio Pacheco-Montealegre; Sheryll Corchuelo
EPI_ISL_906531	Laboratorio de salud publica del Valle del Cauca	Instituto Nacional de Salud- Dirección de Investigación en Salud Pública, Universidad de los Andes- Applied genomics research group, Vicerrectoria de Investigación y Creación, Universidad de los Andes- Systems and Computing Engineering Department	Carlos Franco-Muñoz; Diego A. Álvarez-Díaz; Diego Andrés Prada; Gerardo Santamaría Jorge Duitama; Héctor Alejandro Ruiz-Moreno; Jhonnatan Reales-González; Jorge Ivan Diaz; Julian Naizaque; Katherine Laiton-Donato; Laura Natalia Gonzalez; Magdalena Wiesner; Marcela Mercado-Reyes; Maria T. Herrera-Sepúlveda; Martha Lucia Ospina Martinez; Mauricio Pacheco-Montealegre; Sheryll Corchuelo; Silvia Restrepo-Restrepo
EPI_ISL_2674356	Laboratorio de salud pública de San Andrés	Instituto Nacional de Salud- Dirección de Investigación en Salud Pública	Carlos Franco-Muñoz; Carmen Osorio; Diana Malo; Diego A. Álvarez-Díaz; Diego Andrés Prada; Gerardo Santamaría; Hector Alejandro Ruiz-Moreno; Jhonnatan Reales-González; Jorge Rivera; Juan Camilo Martinez; Julian Naizaque; Katherine Laiton-Donato; Lisseth Pardo; Magdalena Wiesner; Marcela Mercado-Reyes; Maria T. Herrera-Sepúlveda; Marta Lopez Blanco; Martha Lucia Ospina Martinez; Paola Rojas; Sergio Gomez; Sheryll Corchuelo; Ángela Alarcon Cruz
EPI_ISL_1091922, EPI_ISL_1091923	Laboratorio de salud público de Atlantico	Instituto Nacional de Salud- Dirección de Investigación en Salud Pública	Carlos Franco-Muñoz; Diego A. Álvarez-Díaz; Diego Andrés Prada; Gerardo Santamaría; Hector Alejandro Ruiz-Moreno; Jhonnatan Reales-González; Julian Naizaque; Katherine Laiton-Donato; Magdalena Wiesner; Marcela Mercado-Reyes; Maria T. Herrera-Sepúlveda; Martha Lucia Ospina Martinez; Sheryll Corchuelo
EPI_ISL_2657857, EPI_ISL_2657858, EPI_ISL_2657869	Laboratorios del Valle SAS	Instituto Nacional de Salud- Dirección de Investigación en Salud Pública	Carlos Franco-Muñoz; Carmen Osorio; Diana Malo; Diego A. Álvarez-Díaz; Diego Andrés Prada; Gerardo Santamaría; Hector Alejandro Ruiz-Moreno; Jhonnatan Reales-González; Jorge Rivera; Juan Camilo Martinez; Julian Naizaque; Katherine Laiton-Donato; Lisseth Pardo; Magdalena Wiesner; Marcela Mercado-Reyes; Maria T. Herrera-Sepúlveda; Marta Lopez Blanco; Martha Lucia Ospina Martinez; Paola Rojas; Sergio Gomez; Sheryll Corchuelo; Ángela Alarcon Cruz
EPI_ISL_536399	Laboratory of Immunovirology. Universidad de Antioquia	Instituto Nacional de Salud - Unidad de Secuenciación y Genómica	Carlos Franco-Muñoz; Diego Álvarez-Diaz and Marcela Mercado-Reyes; Francisco J. Díaz; Katherine Laiton-Donato; Lizdany Flórez; Wbeimar Aguilar-Jimenez
EPI_ISL_1424057, EPI_ISL_1424058	OLIMPUS MAGDALENA	Instituto Nacional de Salud- Dirección de Investigación en Salud Pública	Carlos Franco-Muñoz; Diego A. Álvarez-Díaz; Diego Andrés Prada; Gerardo Santamaría; Hector Alejandro Ruiz-Moreno; Jhonnatan Reales-González; Julian Naizaque; Katherine Laiton-Donato; Magdalena Wiesner; Marcela Mercado-Reyes.; Maria T. Herrera-Sepúlveda; Martha Lucia Ospina Martínez; Sheryll Corchuelo
EPI_ISL_2321650, EPI_ISL_2321651, EPI_ISL_2321652, EPI_ISL_2321653	One Health	Universidad Nacional de Colombia - Laboratorio Genómico One Health	Andres F. Cardona-Rios; Carlos Franco-Muñoz; Daniel O. Maldonado-Perez; Diego A. Álvarez-Díaz; Hector Alejandro Ruiz-Moreno; Jorge E. Osorio; Juan P. Hernandez-Ortiz; Karl A Ciuoderis; Katherine Laiton-Donato; Laura Silvana Perez; Lina M. Hurtado; Marcela Mercado-Reyes; Maria Stella López; Monica Palma-Cuero; Simón Villegas Velásquez
EPI_ISL_1091782, EPI_ISL_1091783, EPI_ISL_1960047, EPI_ISL_1960071, EPI_ISL_1960076	PECET	Universidad Nacional de Colombia - Laboratorio Genómico One Health	Andres F. Cardona-Rios; Carlos Franco-Muñoz; Carolina Muñoz-Arango; Celeny Ortiz; Daniel O. Maldonado-Perez; Diego A. Álvarez-Díaz; Hector Alejandro Ruiz-Moreno; Idabely Betancur Ortiz; Jorge E. Osorio; Juan P. Hernandez-Ortiz; Karl A Ciuoderis; Katherine Laiton-Donato; Laura Silvana Perez; Lina M. Hurtado; Marcela Mercado-Reyes; Maria Angélica Maya; Maria Stella López; Rita Almanza Payares; Sandra Ines Cano; Simón Villegas Velásquez
EPI_ISL_3459405, EPI_ISL_3459407, EPI_ISL_3459410, EPI_ISL_3459411, EPI_ISL_3459415	PREVIS - IPS	Instituto Nacional de Salud	Carlos Franco-Muñoz; Carmen Osorio; Diana Malo; Diego A. Álvarez-Díaz; Diego Andrés Prada; Gerardo Santamaría; Hector Alejandro Ruiz-Moreno; Jhonnatan Reales-González; Jorge Rivera; Juan Camilo Martinez; Julian Naizaque; Katherine Laiton-Donato; Lisseth Pardo; Magdalena Wiesner; Marcela Mercado-Reyes; Maria T. Herrera-Sepúlveda; Marta Lopez Blanco; Martha Lucia Ospina Martinez; Paola Rojas; Sergio Gomez; Sheryll Corchuelo; Ángela Alarcon Cruz
EPI_ISL_2621177, EPI_ISL_2621178, EPI_ISL_2621179, EPI_ISL_2621180, EPI_ISL_2621181, EPI_ISL_3275683, EPI_ISL_3275684, EPI_ISL_3275685, EPI_ISL_3275686, EPI_ISL_3275687, EPI_ISL_3275688, EPI_ISL_3275689, EPI_ISL_3275690, EPI_ISL_3275691, EPI_ISL_3275692			
see above	PRIME DIAGNOSTICS	Universidad Nacional de Colombia - Laboratorio Genómico One Health	Andres F. Cardona-Rios; Carlos Franco-Muñoz; Carolina Muñoz-Arango; Celeny Ortiz; Daniel O. Maldonado-Perez; Diego A. Álvarez-Díaz; Hector Alejandro Ruiz-Moreno; Idabely Betancur Ortiz; Jorge E. Osorio; Juan P. Hernandez-Ortiz; Karl A Ciuoderis; Katherine Laiton-Donato; Laura Silvana Perez; Lina M. Hurtado; Marcela Mercado-Reyes; Maria Angélica Maya; Maria Stella López; Rita Almanza Payares; Sandra Ines Cano; Simón Villegas Velásquez
EPI_ISL_2621198	PROANALAB	Universidad Nacional de Colombia - Laboratorio Genómico One Health	Andres F. Cardona-Rios; Carlos Franco-Muñoz; Carolina Muñoz-Arango; Celeny Ortiz; Daniel O. Maldonado-Perez; Diego A. Álvarez-Díaz; Hector Alejandro Ruiz-Moreno; Idabely Betancur Ortiz; Jorge E. Osorio; Juan P. Hernandez-Ortiz; Karl A Ciuoderis; Katherine Laiton-Donato; Laura Silvana Perez; Lina M. Hurtado; Marcela Mercado-Reyes; Maria Angélica Maya; Maria Stella López; Rita Almanza Payares; Sandra Ines Cano; Simón Villegas Velásquez
EPI_ISL_2437995, EPI_ISL_2437996, EPI_ISL_2437997	Prime Diagnostics	Universidad Nacional de Colombia - Laboratorio Genómico One Health	Andres F. Cardona-Rios; Carlos Franco-Muñoz; Carolina Muñoz-Arango; Celeny Ortiz; Daniel O. Maldonado-Perez; Diego A. Álvarez-Díaz; Hector Alejandro Ruiz-Moreno; Idabely Betancur Ortiz; Jorge E. Osorio; Juan P. Hernandez-Ortiz; Karl A Ciuoderis; Katherine Laiton-Donato; Laura Silvana Perez; Lina M. Hurtado; Marcela Mercado-Reyes; Maria Angélica Maya; Maria Stella López; Rita Almanza Payares; Sandra Ines Cano; Simón Villegas Velásquez
EPI_ISL_2321587, EPI_ISL_2321588, EPI_ISL_2621209, EPI_ISL_2621210, EPI_ISL_2621211	SAN VICENTE FUNDACION	Universidad Nacional de Colombia - Laboratorio Genómico One Health	Andres F. Cardona-Rios; Carlos Franco-Muñoz; Carolina Muñoz-Arango; Celeny Ortiz; Daniel O. Maldonado-Perez; Diego A. Álvarez-Díaz; Hector Alejandro Ruiz-Moreno; Idabely Betancur Ortiz; Jorge E. Osorio; Juan P. Hernandez-Ortiz; Karl A Ciuoderis; Katherine Laiton-Donato; Laura Silvana Perez; Lina M. Hurtado; Marcela Mercado-Reyes; Maria Angélica Maya; Maria Stella López; Rita Almanza Payares; Sandra Ines Cano; Simón Villegas Velásquez
EPI_ISL_1960064	SAN VICENTE FUNDACIÓN	Universidad Nacional de Colombia - Laboratorio Genómico One Health	Andres F. Cardona-Rios; Carlos Franco-Muñoz; Carolina Muñoz-Arango; Celeny Ortiz; Daniel O. Maldonado-Perez; Diego A. Álvarez-Díaz; Hector Alejandro Ruiz-Moreno; Idabely Betancur Ortiz; Jorge E. Osorio; Juan P. Hernandez-Ortiz; Karl A Ciuoderis; Katherine Laiton-Donato; Laura Silvana Perez; Lina M. Hurtado; Marcela Mercado-Reyes; Maria Angélica Maya; Maria Stella López; Rita Almanza Payares; Sandra Ines Cano; Simón Villegas Velásquez
EPI_ISL_2674350	SECCION SERVICIOS INTEGRALES DE SALUD Y DESARROLLO PSICOSOCIAL DE BIENESTAR UNIVERSITARIO UIS	Instituto Nacional de Salud- Dirección de Investigación en Salud Pública	Carlos Franco-Muñoz; Carmen Osorio; Diana Malo; Diego A. Álvarez-Díaz; Diego Andrés Prada; Gerardo Santamaría; Hector Alejandro Ruiz-Moreno; Jhonnatan Reales-González; Jorge Rivera; Juan Camilo Martinez; Julian Naizaque; Katherine Laiton-Donato; Lisseth Pardo; Magdalena Wiesner; Marcela Mercado-Reyes; Maria T. Herrera-Sepúlveda; Marta Lopez Blanco; Martha Lucia Ospina Martinez; Paola Rojas; Sergio Gomez; Sheryll Corchuelo; Ángela Alarcon Cruz
EPI_ISL_3459382, EPI_ISL_3459384	SECRETARIA DE SALUD	Instituto Nacional de Salud	Carlos Franco-Muñoz; Carmen Osorio; Diana Malo; Diego A. Álvarez-Díaz; Diego Andrés Prada; Gerardo Santamaría; Hector Alejandro Ruiz-Moreno; Jhonnatan Reales-González; Jorge Rivera; Juan Camilo Martinez; Julian Naizaque; Katherine Laiton-Donato; Lisseth Pardo; Magdalena Wiesner; Marcela Mercado-Reyes; Maria T. Herrera-Sepúlveda; Marta Lopez Blanco; Martha Lucia Ospina Martinez; Paola Rojas; Sergio Gomez; Sheryll Corchuelo; Ángela Alarcon Cruz
EPI_ISL_3066770	SECRETARIA DE SALUD DE SAN ANDRES ISLA	Instituto Nacional de Salud	Carlos Franco-Muñoz; Carmen Osorio; Diana Malo; Diego A. Álvarez-Díaz; Diego Andrés Prada; Gerardo Santamaría; Hector Alejandro Ruiz-Moreno; Jhonnatan Reales-González; Jorge Rivera; Juan Camilo Martinez; Julian Naizaque; Katherine Laiton-Donato; Lisseth Pardo; Magdalena Wiesner; Marcela Mercado-Reyes; Maria T.Herrera-Sepúlveda; Marta Lopez Blanco; Martha Lucia Ospina Martinez; Paola Rojas; Sergio Gomez; Sheryll Corchuelo; Ángela Alarcon Cruz
EPI_ISL_3385800	SECRETARIA DE SALUD DE SAN ANDRES ISLA	Instituto Nacional de Salud- Dirección de Investigación en Salud Pública	Carlos Franco-Muñoz; Carmen Osorio; Diana Malo; Diego A. Álvarez-Díaz; Diego Andrés Prada; Gerardo Santamaría; Hector Alejandro Ruiz-Moreno; Jhonnatan Reales-González; Jorge Rivera; Juan Camilo Martinez; Julian Naizaque; Katherine Laiton-Donato; Lisseth Pardo; Magdalena Wiesner; Marcela Mercado-Reyes; Maria T. Herrera-Sepúlveda; Marta Lopez Blanco; Martha Lucia Ospina Martinez; Paola Rojas; Sergio Gomez; Sheryll Corchuelo; Ángela Alarcon Cruz
EPI_ISL_3385776, EPI_ISL_3385803, EPI_ISL_3385821, EPI_ISL_3385835, EPI_ISL_3385840	SERVICIOS MEDICOS OLIMPUS	Instituto Nacional de Salud- Dirección de Investigación en Salud Pública	Carlos Franco-Muñoz; Carmen Osorio; Diana Malo; Diego A. Álvarez-Díaz; Diego Andrés Prada; Gerardo Santamaría; Hector Alejandro Ruiz-Moreno; Jhonnatan Reales-González; Jorge Rivera; Juan Camilo Martinez; Julian Naizaque; Katherine Laiton-Donato; Lisseth Pardo; Magdalena Wiesner; Marcela Mercado-Reyes; Maria T. Herrera-Sepúlveda; Marta Lopez Blanco; Martha Lucia Ospina Martinez; Paola Rojas; Sergio Gomez; Sheryll Corchuelo; Ángela Alarcon Cruz
EPI_ISL_1629721, EPI_ISL_1629722, EPI_ISL_1629723, EPI_ISL_1629724, EPI_ISL_1629725, EPI_ISL_1629726, EPI_ISL_1629727, EPI_ISL_1675299, EPI_ISL_1675300, EPI_ISL_1675301, EPI_ISL_1675302, EPI_ISL_1675318, EPI_ISL_1824705, EPI_ISL_1960049, EPI_ISL_2321627, EPI_ISL_2321628, EPI_ISL_2321629, EPI_ISL_2321630, EPI_ISL_2321631, EPI_ISL_2321632, EPI_ISL_2321633, EPI_ISL_2321634, EPI_ISL_2321635, EPI_ISL_2321636, EPI_ISL_2321637, EPI_ISL_2321638, EPI_ISL_2321639, EPI_ISL_2321640, EPI_ISL_2321641, EPI_ISL_2321642, EPI_ISL_2321643, EPI_ISL_2321644, EPI_ISL_2321645, EPI_ISL_2321646, EPI_ISL_2321647, EPI_ISL_2321648, EPI_ISL_2321649, EPI_ISL_3275693, EPI_ISL_3275694, EPI_ISL_3276555, EPI_ISL_3276556, EPI_ISL_3276557, EPI_ISL_3276558			
see above	SOMER	Universidad Nacional de Colombia - Laboratorio Genómico One Health	Andres F. Cardona-Rios; Carlos Franco-Muñoz; Carolina Muñoz-Arango; Celeny Ortiz; Daniel O. Maldonado-Perez; Diego A. Álvarez-Díaz; Eliana Patricia Calvo Tapiero; Hector Alejandro Ruiz-Moreno; Idabely Betancur Ortiz; Jorge E. Osorio; Juan P. Hernandez-Ortiz; Karl A Ciuoderis; Katherine Laiton-Donato; Laura Silvana Perez; Lina M. Hurtado; Marcela Mercado-Reyes; Maria Angélica Maya; Maria Stella López; Rita Almanza Payares; Sandra Ines Cano; Simón Villegas Velásquez
EPI_ISL_2674305, EPI_ISL_2674306, EPI_ISL_2674307, EPI_ISL_2674308	SURA	Instituto Nacional de Salud- Dirección de Investigación en Salud Pública	Carlos Franco-Muñoz; Carmen Osorio; Diana Malo; Diego A. Álvarez-Díaz; Diego Andrés Prada; Gerardo Santamaría; Hector Alejandro Ruiz-Moreno; Jhonnatan Reales-González; Jorge Rivera; Juan Camilo Martinez; Julian Naizaque; Katherine Laiton-Donato; Lisseth Pardo; Magdalena Wiesner; Marcela Mercado-Reyes; Maria T. Herrera-Sepúlveda; Marta Lopez Blanco; Martha Lucia Ospina Martinez; Paola Rojas; Sergio Gomez; Sheryll Corchuelo; Ángela Alarcon Cruz
EPI_ISL_1629733, EPI_ISL_1629734, EPI_ISL_1629735, EPI_ISL_1629736, EPI_ISL_1629737, EPI_ISL_1675319, EPI_ISL_1675320, EPI_ISL_1675321, EPI_ISL_1820901, EPI_ISL_1820902, EPI_ISL_1820903, EPI_ISL_1820904, EPI_ISL_1820905, EPI_ISL_1820906, EPI_ISL_1820907, EPI_ISL_1820908, EPI_ISL_1820909, EPI_ISL_1960039, EPI_ISL_1960040, EPI_ISL_1960041, EPI_ISL_1960042, EPI_ISL_1960053, EPI_ISL_1960059, EPI_ISL_1960060, EPI_ISL_1960061, EPI_ISL_1960066, EPI_ISL_1960067, EPI_ISL_1960069, EPI_ISL_1960070, EPI_ISL_1960072, EPI_ISL_2136275, EPI_ISL_2136276, EPI_ISL_2136277, EPI_ISL_2136278, EPI_ISL_2136279, EPI_ISL_2621828, EPI_ISL_2621829, EPI_ISL_2621830, EPI_ISL_2621831, EPI_ISL_2621832, EPI_ISL_2621833, EPI_ISL_2621834, EPI_ISL_2621835, EPI_ISL_2621836, EPI_ISL_2621837, EPI_ISL_2621838			
see above	SURA	Universidad Nacional de Colombia - Laboratorio Genómico One Health	Andres F. Cardona-Rios; Carlos Franco-Muñoz; Carolina Muñoz-Arango; Celeny Ortiz; Daniel O. Maldonado-Perez; Diego A. Álvarez-Díaz; Eliana Patricia Calvo Tapiero; Hector Alejandro Ruiz-Moreno; Idabely Betancur Ortiz; Jorge E. Osorio; Juan P. Hernandez-Ortiz; Karl A Ciuoderis; Katherine Laiton-Donato; Laura Silvana Perez; Lina M. Hurtado; Marcela Mercado-Reyes; Maria Angélica Maya; Maria Stella López; Rita Almanza Payares; Sandra Ines Cano; Simón Villegas Velásquez
EPI_ISL_1629748, EPI_ISL_1629749, EPI_ISL_1629750, EPI_ISL_1629751, EPI_ISL_1629752, EPI_ISL_1675310, EPI_ISL_1675311, EPI_ISL_1675312, EPI_ISL_1824701, EPI_ISL_1960048, EPI_ISL_1960055, EPI_ISL_1960080, EPI_ISL_2321576, EPI_ISL_2321577, EPI_ISL_2651235, EPI_ISL_3276547, EPI_ISL_3276548			

see above	SYNLAB	Universidad Nacional de Colombia - Laboratorio Genómico One Health	Andres F. Cardona-Rios; Carlos Franco-Muñoz; Carolina Muñoz-Arango; Celeny Ortiz; Daniel O. Maldonado-Perez; Diego A. Álvarez-Díaz; Eliana Patricia Calvo Tapiero; Hector Alejandro Ruiz-Moreno; Idabely Betancur Ortiz; Jorge E. Osorio; Juan P. Hernandez-Ortiz; Karl A Ciuoderis; Katherine Laiton-Donato; Laura Silvana Perez; Lina M. Hurtado; Marcela Mercado-Reyes; Maria Angélica Maya; Maria Stella López; Rita Almanza Payares; Sandra Ines Cano; Simón Villegas Velásquez
EPI_ISL_3355472, EPI_ISL_3355473, EPI_ISL_3355474, EPI_ISL_3355475, EPI_ISL_3355476, EPI_ISL_3355481, EPI_ISL_3355486, EPI_ISL_3355492, EPI_ISL_3355493, EPI_ISL_3355494, EPI_ISL_3355495, EPI_ISL_3355496, EPI_ISL_3355497, EPI_ISL_3355498, EPI_ISL_3355499, EPI_ISL_3355500, EPI_ISL_3355501, EPI_ISL_3355502, EPI_ISL_3355503, EPI_ISL_3355504, EPI_ISL_3355505, EPI_ISL_3355506, EPI_ISL_3355507, EPI_ISL_3355508, EPI_ISL_3355509, EPI_ISL_3355510, EPI_ISL_3355511, EPI_ISL_3355512, EPI_ISL_3355513, EPI_ISL_3369920, EPI_ISL_3369921, EPI_ISL_3369927, EPI_ISL_3369929, EPI_ISL_3369930, EPI_ISL_3369931, EPI_ISL_3369935, EPI_ISL_3369937, EPI_ISL_3369940, EPI_ISL_3369942, EPI_ISL_3369943, EPI_ISL_3369944, EPI_ISL_3369947, EPI_ISL_3369948, EPI_ISL_3369949			
see above	SYNLAB - ANGEL DIAGNOSTICA	Corporacion CorpoGen-Universidad de los Andes-Universidad Central	Christian Romero; Jorge Duitama; Juan Manuel Anzola; Laura González; Maryam Chaib De Mares; María Mercedes Zambrano; Nelly Díaz; Patricia Del Portillo; Silvia Restrepo
EPI_ISL_2362560	SYNLAB - ANGEL DIAGNOSTICA	Instituto Nacional de Salud- Dirección de Investigación en Salud Pública	Carlos Franco-Muñoz; Carmen Osorio; Diana Malo; Diego A. Álvarez-Díaz; Diego Andrés Prada; Gerardo Santamaría; Hector Alejandro Ruiz-Moreno; Jhonnatan Reales-González; Jorge Rivera; Juan Camilo Martinez; Julian Naizaque; Katherine Laiton-Donato; Lisseth Pardo; Magdalena Wiesner; Marcela Mercado-Reyes; Maria T. Herrera-Sepúlveda; Marta Lopez Blanco; Martha Lucia Ospina Martinez; Paola Rojas; Sergio Gomez; Sheryll Corchuelo; Ángela Alarcon Cruz
EPI_ISL_2155037, EPI_ISL_2155038, EPI_ISL_2155039, EPI_ISL_2155040, EPI_ISL_2158352	SYNLAB -ANGEL DIAGNOSTICA	Instituto Nacional de Salud- Dirección de Investigación en Salud Pública	Carlos Franco-Muñoz; Carmen Osorio; Diana Malo; Diego A. Álvarez-Díaz; Diego Andrés Prada; Gerardo Santamaría; Hector Alejandro Ruiz-Moreno; Jhonnatan Reales-González; Jorge Rivera; Juan Camilo Martinez; Julian Naizaque; Katherine Laiton-Donato; Lisseth Pardo; Magdalena Wiesner; Marcela Mercado-Reyes; Maria T. Herrera-Sepúlveda; Marta Lopez Blanco; Martha Lucia Ospina Martinez; Paola Rojas; Sergio Gomez; Sheryll Corchuelo; Ángela Alarcon Cruz
EPI_ISL_1632494, EPI_ISL_1632495, EPI_ISL_2009058, EPI_ISL_2009059	SYNLAB ANGEL DIAGNOSTICA	Instituto Nacional de Salud- Dirección de Investigación en Salud Pública	Carlos Franco-Muñoz; Carmen Osorio; Diana Malo; Diego A. Álvarez-Díaz; Diego Andrés Prada; Gerardo Santamaría; Hector Alejandro Ruiz-Moreno; Jhonnatan Reales-González; Jorge Rivera; Juan Camilo Martinez; Julian Naizaque; Katherine Laiton-Donato; Lisseth Pardo; Magdalena Wiesner; Marcela Mercado-Reyes; Maria T. Herrera-Sepúlveda; Marta Lopez Blanco; Martha Lucia Ospina Martinez; Paola Rojas; Sergio Gomez; Sheryll Corchuelo; Ángela Alarcon Cruz
EPI_ISL_845627	SYNLAB COLOMBIA S.A.S	Instituto Nacional de Salud - Dirección de Investigación en Salud Pública	Carlos Franco-Muñoz; Diego A. Álvarez-Díaz; Diego Andrés Prada; Gerardo Santamaría; Jonathan Reales; Julian Naizaque; Katherine Laiton-Donato; Magdalena Wiesner; Marcela Mercado-Reyes; Maria T. Herrera-Sepúlveda; Martha Lucia Ospina Martinez; Mauricio Pacheco-Montealegre; Paola Muñoz-Laiton; Sheryll Corchuelo
EPI_ISL_1632496	SYNLAB REGIONAL CALI	Instituto Nacional de Salud- Dirección de Investigación en Salud Pública	Carlos Franco-Muñoz; Carmen Osorio; Diana Malo; Diego A. Álvarez-Díaz; Diego Andrés Prada; Gerardo Santamaría; Hector Alejandro Ruiz-Moreno; Jhonnatan Reales-González; Jorge Rivera; Juan Camilo Martinez; Julian Naizaque; Katherine Laiton-Donato; Lisseth Pardo; Magdalena Wiesner; Marcela Mercado-Reyes; Maria T. Herrera-Sepúlveda; Marta Lopez Blanco; Martha Lucia Ospina Martinez; Paola Rojas; Sergio Gomez; Sheryll Corchuelo; Ángela Alarcon Cruz
EPI_ISL_794666, EPI_ISL_794667	SYNLAB REGIONAL NOROCCIDENTE	Instituto Nacional de Salud - Dirección de Investigación en Salud Pública	Carlos Franco-Muñoz; Diego A. Álvarez-Díaz; Diego Andrés Prada; Gerardo Santamaría; Jonathan Reales; Julian Naizaque; Katherine Laiton-Donato; Magdalena Wiesner; Marcela Mercado-Reyes; Maria T. Herrera; Martha Lucia Ospina Martinez; Mauricio Pacheco-Montealegre; Paola Muñoz-Laiton; Sheryll Corchuelo
EPI_ISL_3391980, EPI_ISL_3391987	Secretaria De Salud Del Guaviare	Instituto Nacional de Salud- Dirección de Investigación en Salud Pública	Carlos Franco-Muñoz; Carmen Osorio; Diana Malo; Diego A. Álvarez-Díaz; Diego Andrés Prada; Gerardo Santamaría; Hector Alejandro Ruiz-Moreno; Jhonnatan Reales-González; Jorge Rivera; Juan Camilo Martinez; Julian Naizaque; Katherine Laiton-Donato; Lisseth Pardo; Magdalena Wiesner; Marcela Mercado-Reyes; Maria T. Herrera-Sepúlveda; Marta Lopez Blanco; Martha Lucia Ospina Martinez; Paola Rojas; Sergio Gomez; Sheryll Corchuelo; Ángela Alarcon Cruz
EPI_ISL_417924	Secretaría de Salud Medellín	Instituto Nacional de Salud, Universidad Cooperativa de Colombia, Instituto Alexander von Humboldt, Imperial College-London, London School of Hygiene & Tropical Medicine	Astrid C. Flórez; Carlos Franco-Muñoz; Christian Julian Villabona-Arenas; Diana Marcela Walteros-Acero; Diego A. Álvarez-Díaz; Erika Ospitia; Gloria Puerto; Jose A. Usme-Ciro; Juliana Barbosa; Katherine Laiton-Donato; Liz Villabona-Arenas; Luz Dary Rodriguez; Mailyn A. Gonzalez; Marcela Mercado-Reyes; Martha Lucia Ospina Martinez; Nicolás D. Franco-Sierra; Sergio Gomez Rangel; Sussy Echeverria-Londoño; Zulma M. Cucunubá
EPI_ISL_2437998, EPI_ISL_2438024, EPI_ISL_2438025, EPI_ISL_2438026, EPI_ISL_2438027, EPI_ISL_2438028, EPI_ISL_2438029, EPI_ISL_2438030, EPI_ISL_2438031, EPI_ISL_2438032, EPI_ISL_2438033, EPI_ISL_2438034, EPI_ISL_2438035, EPI_ISL_2438036, EPI_ISL_2438039, EPI_ISL_2438041, EPI_ISL_2438045, EPI_ISL_2621182, EPI_ISL_2621183, EPI_ISL_2621184, EPI_ISL_2621185, EPI_ISL_2621186, EPI_ISL_2621187, EPI_ISL_2621188, EPI_ISL_2621189, EPI_ISL_2621190, EPI_ISL_2621191, EPI_ISL_2621192, EPI_ISL_2621193, EPI_ISL_2621194, EPI_ISL_2621195, EPI_ISL_2621196, EPI_ISL_2621197, EPI_ISL_2621878, EPI_ISL_2621879, EPI_ISL_2621880, EPI_ISL_2621881, EPI_ISL_2621882, EPI_ISL_2621883, EPI_ISL_2651204, EPI_ISL_2651205, EPI_ISL_2651206, EPI_ISL_2651207, EPI_ISL_2651208, EPI_ISL_2651209, EPI_ISL_2651210, EPI_ISL_2651211, EPI_ISL_2651212, EPI_ISL_2651213, EPI_ISL_2651214			
see above	Somer	Universidad Nacional de Colombia - Laboratorio Genómico One Health	Andres F. Cardona-Rios; Carlos Franco-Muñoz; Carolina Muñoz-Arango; Celeny Ortiz; Daniel O. Maldonado-Perez; Diego A. Álvarez-Díaz; Hector Alejandro Ruiz-Moreno; Idabely Betancur Ortiz; Jorge E. Osorio; Juan P. Hernandez-Ortiz; Karl A Ciuoderis; Katherine Laiton-Donato; Laura Silvana Perez; Lina M. Hurtado; Marcela Mercado-Reyes; Maria Angélica Maya; Maria Stella López; Rita Almanza Payares; Sandra Ines Cano; Simón Villegas Velásquez
EPI_ISL_2438015, EPI_ISL_2438016, EPI_ISL_2438017, EPI_ISL_2438018, EPI_ISL_2438019, EPI_ISL_2438020, EPI_ISL_2438021, EPI_ISL_2438022, EPI_ISL_2438023, EPI_ISL_2438048, EPI_ISL_2438050, EPI_ISL_2438052, EPI_ISL_2438055, EPI_ISL_2621148, EPI_ISL_2621149, EPI_ISL_2621150, EPI_ISL_2621151, EPI_ISL_2621152, EPI_ISL_2621153, EPI_ISL_2621154, EPI_ISL_2621155, EPI_ISL_2621156, EPI_ISL_2621199, EPI_ISL_2621200, EPI_ISL_2621201, EPI_ISL_2621202, EPI_ISL_2621203, EPI_ISL_2621204, EPI_ISL_2621852, EPI_ISL_2621853, EPI_ISL_2621854, EPI_ISL_2621855, EPI_ISL_2621856, EPI_ISL_2621857, EPI_ISL_2621858, EPI_ISL_2621876, EPI_ISL_2621877			
see above	Synlab	Universidad Nacional de Colombia - Laboratorio Genómico One Health	Andres F. Cardona-Rios; Carlos Franco-Muñoz; Carolina Muñoz-Arango; Celeny Ortiz; Daniel O. Maldonado-Perez; Diego A. Álvarez-Díaz; Hector Alejandro Ruiz-Moreno; Idabely Betancur Ortiz; Jorge E. Osorio; Juan P. Hernandez-Ortiz; Karl A Ciuoderis; Katherine Laiton-Donato; Laura Silvana Perez; Lina M. Hurtado; Marcela Mercado-Reyes; Maria Angélica Maya; Maria Stella López; Rita Almanza Payares; Sandra Ines Cano; Simón Villegas Velásquez
EPI_ISL_1629728, EPI_ISL_1629729, EPI_ISL_1629730, EPI_ISL_1629731, EPI_ISL_1629732, EPI_ISL_1675303, EPI_ISL_1675304, EPI_ISL_1675305, EPI_ISL_1675306, EPI_ISL_1675307, EPI_ISL_1675323, EPI_ISL_1675324, EPI_ISL_1675325, EPI_ISL_1824708, EPI_ISL_1960046, EPI_ISL_1960063, EPI_ISL_1960082, EPI_ISL_2438057, EPI_ISL_2438059, EPI_ISL_2438086, EPI_ISL_2438089, EPI_ISL_2438092			
see above	UDEA	Universidad Nacional de Colombia - Laboratorio Genómico One Health	Andres F. Cardona-Rios; Carlos Franco-Muñoz; Carolina Muñoz-Arango; Celeny Ortiz; Daniel O. Maldonado-Perez; Diego A. Álvarez-Díaz; Eliana Patricia Calvo Tapiero; Hector Alejandro Ruiz-Moreno; Idabely Betancur Ortiz; Jorge E. Osorio; Juan P. Hernandez-Ortiz; Karl A Ciuoderis; Katherine Laiton-Donato; Laura Silvana Perez; Lina M. Hurtado; Marcela Mercado-Reyes; Maria Angélica Maya; Maria Stella López; Rita Almanza Payares; Sandra Ines Cano; Simón Villegas Velásquez
EPI_ISL_3066764, EPI_ISL_3398810	UNIDAD DE DIAGNOSTICO HEMATO ONCOLOGICA	Instituto Nacional de Salud	Carlos Franco-Muñoz; Carmen Osorio; Diana Malo; Diego A. Álvarez-Díaz; Diego Andrés Prada; Gerardo Santamaría; Hector Alejandro Ruiz-Moreno; Jhonnatan Reales-González; Jorge Rivera; Juan Camilo Martinez; Julian Naizaque; Katherine Laiton-Donato; Lisseth Pardo; Magdalena Wiesner; Marcela Mercado-Reyes; Maria T.Herrera-Sepúlveda; Marta Lopez Blanco; Martha Lucia Ospina Martinez; Paola Rojas; Sergio Gomez; Sheryll Corchuelo; Ángela Alarcon Cruz
EPI_ISL_3385787, EPI_ISL_3385807, EPI_ISL_3385808, EPI_ISL_3385834, EPI_ISL_3385849	UNIDAD DE DIAGNOSTICO HEMATO ONCOLOGICA	Instituto Nacional de Salud- Dirección de Investigación en Salud Pública	Carlos Franco-Muñoz; Carmen Osorio; Diana Malo; Diego A. Álvarez-Díaz; Diego Andrés Prada; Gerardo Santamaría; Hector Alejandro Ruiz-Moreno; Jhonnatan Reales-González; Jorge Rivera; Juan Camilo Martinez; Julian Naizaque; Katherine Laiton-Donato; Lisseth Pardo; Magdalena Wiesner; Marcela Mercado-Reyes; Maria T. Herrera-Sepúlveda; Marta Lopez Blanco; Martha Lucia Ospina Martinez; Paola Rojas; Sergio Gomez; Sheryll Corchuelo; Ángela Alarcon Cruz
EPI_ISL_794656, EPI_ISL_794657	UNIDAD HEMATOLOGICA ESPECIALIZADA	Instituto Nacional de Salud - Dirección de Investigación en Salud Pública	Carlos Franco-Muñoz; Diego A. Álvarez-Díaz; Diego Andrés Prada; Gerardo Santamaría; Jonathan Reales; Julian Naizaque; Katherine Laiton-Donato; Magdalena Wiesner; Marcela Mercado-Reyes; Maria T. Herrera; Martha Lucia Ospina Martinez; Mauricio Pacheco-Montealegre; Paola Muñoz-Laiton; Sheryll Corchuelo
EPI_ISL_4300689, EPI_ISL_4300690	UNIDAD HEMATOLOGICA ESPECIALIZADA	Instituto Nacional de Salud- Dirección de Investigación en Salud Pública	Carlos Franco-Muñoz; Carmen Osorio; Diana Malo; Diego A. Álvarez-Díaz; Diego Andrés Prada; Gerardo Santamaría; Hector Alejandro Ruiz-Moreno; Jhonnatan Reales-González; Jorge Rivera; Juan Camilo Martinez; Julian Naizaque; Katherine Laiton-Donato; Lisseth Pardo; Magdalena Wiesner; Marcela Mercado-Reyes; Maria T. Herrera-Sepúlveda; Marta Lopez Blanco; Martha Lucia Ospina Martinez; Paola Rojas; Sergio Gomez; Sheryll Corchuelo; Ángela Alarcon Cruz
EPI_ISL_845656	UNIDAD HEMATOLÓGICA ESPECIALIZADA	Instituto Nacional de Salud - Dirección de Investigación en Salud Pública	Carlos Franco-Muñoz; Diego A. Álvarez-Díaz; Diego Andrés Prada; Gerardo Santamaría; Jonathan Reales; Julian Naizaque; Katherine Laiton-Donato; Magdalena Wiesner; Marcela Mercado-Reyes; Maria T. Herrera-Sepúlveda; Martha Lucia Ospina Martinez; Mauricio Pacheco-Montealegre; Paola Muñoz-Laiton; Sheryll Corchuelo
EPI_ISL_1015353, EPI_ISL_1015354, EPI_ISL_1015355, EPI_ISL_1015356, EPI_ISL_1015357, EPI_ISL_1960038, EPI_ISL_2136280, EPI_ISL_2136281, EPI_ISL_2136282, EPI_ISL_2136283, EPI_ISL_2136284, EPI_ISL_2136285, EPI_ISL_2136286, EPI_ISL_2136287, EPI_ISL_2136288, EPI_ISL_2136289, EPI_ISL_2136290, EPI_ISL_2136291, EPI_ISL_2136292, EPI_ISL_2136293, EPI_ISL_2136294, EPI_ISL_2136295, EPI_ISL_2136296, EPI_ISL_2136297, EPI_ISL_2136298, EPI_ISL_2136299, EPI_ISL_2136300, EPI_ISL_2136301, EPI_ISL_2136302, EPI_ISL_2136303, EPI_ISL_2136304, EPI_ISL_2136305, EPI_ISL_2136306, EPI_ISL_2136307, EPI_ISL_2136308, EPI_ISL_2136309, EPI_ISL_2136310, EPI_ISL_2136311, EPI_ISL_2136312, EPI_ISL_2136313, EPI_ISL_2136314, EPI_ISL_2136315, EPI_ISL_2136316, EPI_ISL_2136317, EPI_ISL_2136318, EPI_ISL_2136319, EPI_ISL_2136320, EPI_ISL_2136321, EPI_ISL_2136322, EPI_ISL_2136323, EPI_ISL_2136324, EPI_ISL_2136325, EPI_ISL_2136326, EPI_ISL_2136327, EPI_ISL_2136328, EPI_ISL_2136329, EPI_ISL_2136330, EPI_ISL_2136331, EPI_ISL_2136332, EPI_ISL_2136333, EPI_ISL_2136334, EPI_ISL_2136335, EPI_ISL_2136336, EPI_ISL_2321592, EPI_ISL_2321593, EPI_ISL_2321594, EPI_ISL_2321595, EPI_ISL_2321596, EPI_ISL_2321597, EPI_ISL_2321598, EPI_ISL_2321599, EPI_ISL_2321600, EPI_ISL_2321601, EPI_ISL_2321602, EPI_ISL_2321603, EPI_ISL_2321604, EPI_ISL_2321605, EPI_ISL_2321606, EPI_ISL_2321607, EPI_ISL_2321608, EPI_ISL_2321609, EPI_ISL_2321610, EPI_ISL_2321611, EPI_ISL_2321612, EPI_ISL_2321613, EPI_ISL_2321614, EPI_ISL_2321615, EPI_ISL_2321616, EPI_ISL_2321617, EPI_ISL_2321618, EPI_ISL_2321619, EPI_ISL_2321620, EPI_ISL_2321621, EPI_ISL_2321622, EPI_ISL_2321623, EPI_ISL_2321624, EPI_ISL_2321625, EPI_ISL_2321626, EPI_ISL_3266996, EPI_ISL_3266997, EPI_ISL_3266999, EPI_ISL_3267000, EPI_ISL_3267043			
see above	UNIGEM	Universidad Nacional de Colombia - Laboratorio Genómico One Health	Andres F. Cardona-Rios; Carlos Franco-Muñoz; Carolina Muñoz-Arango; Celeny Ortiz; Daniel O. Maldonado-Perez; Diego A. Álvarez-Díaz; Hector Alejandro Ruiz-Moreno; Idabely Betancur Ortiz; Jorge E. Osorio; Juan P. Hernandez-Ortiz; Karl A Ciuoderis; Katherine Laiton-Donato; Laura Silvana Perez; Lina M. Hurtado; Marcela Mercado-Reyes; Maria Angélica Maya; Maria Stella López; Rita Almanza Payares; Sandra Ines Cano; Simón Villegas Velásquez
EPI_ISL_3385823, EPI_ISL_3385854	UNIVERSIDAD DE CALDAS	Instituto Nacional de Salud- Dirección de Investigación en Salud Pública	Carlos Franco-Muñoz; Carmen Osorio; Diana Malo; Diego A. Álvarez-Díaz; Diego Andrés Prada; Gerardo Santamaría; Hector Alejandro Ruiz-Moreno; Jhonnatan Reales-González; Jorge Rivera; Juan Camilo Martinez; Julian Naizaque; Katherine Laiton-Donato; Lisseth Pardo; Magdalena Wiesner; Marcela Mercado-Reyes; Maria T. Herrera-Sepúlveda; Marta Lopez Blanco; Martha Lucia Ospina Martinez; Paola Rojas; Sergio Gomez; Sheryll Corchuelo; Ángela Alarcon Cruz
EPI_ISL_1632532, EPI_ISL_1820925, EPI_ISL_1820935, EPI_ISL_1820938, EPI_ISL_1820939, EPI_ISL_1820940, EPI_ISL_1820941, EPI_ISL_1820943, EPI_ISL_1820950, EPI_ISL_1820954, EPI_ISL_1820957, EPI_ISL_1820960, EPI_ISL_1821882, EPI_ISL_3385781, EPI_ISL_3385838			
see above	UNIVERSIDAD DE CARTAGENA	Instituto Nacional de Salud- Dirección de Investigación en Salud Pública	Carlos Franco-Muñoz; Carmen Osorio; Christian Romero; Diana Malo; Diego A. Álvarez-Díaz; Diego Andrés Prada; Gerardo Santamaría; Hector Alejandro Ruiz-Moreno; Jhonnatan Reales-González; Jorge Rivera; Juan Camilo Martinez; Julian Naizaque; Katherine Laiton-Donato; Lisseth Pardo; Magdalena Wiesner; Marcela Mercado-Reyes; Maria T. Herrera-Sepúlveda; Marta Lopez Blanco; Martha Lucia Ospina Martinez; Paola Rojas; Patricia del Portillo; Sergio Gomez; Sheryll Corchuelo; Ángela Alarcon Cruz
EPI_ISL_2362559	UNIVERSIDAD DE CORDOBA	Instituto Nacional de Salud- Dirección de Investigación en Salud Pública	Carlos Franco-Muñoz; Carmen Osorio; Diana Malo; Diego A. Álvarez-Díaz; Diego Andrés Prada; Gerardo Santamaría; Hector Alejandro Ruiz-Moreno; Jhonnatan Reales-González; Jorge Rivera; Juan Camilo Martinez; Julian Naizaque; Katherine Laiton-Donato; Lisseth Pardo; Magdalena Wiesner; Marcela Mercado-Reyes; Maria T. Herrera-Sepúlveda; Marta Lopez Blanco; Martha Lucia Ospina Martinez; Paola Rojas; Sergio Gomez; Sheryll Corchuelo; Ángela Alarcon Cruz
EPI_ISL_1303369, EPI_ISL_1303370, EPI_ISL_1303375	UNIVERSIDAD DE CORDOBA - INSTITUTO DE INVESTIGACIONES BIOLÓGICAS DEL TRÓPICO IIBT	Instituto Nacional de Salud- Dirección de Investigación en Salud Pública	Carlos Franco-Muñoz; Carmen Osorio; Diana Malo; Diego A. Álvarez-Díaz; Diego Andrés Prada; Gerardo Santamaría; Hector Alejandro Ruiz-Moreno; Jhonnatan Reales-González; Juan Camilo Martinez; Julian Naizaque; Katherine Laiton-Donato; Lisseth Pardo; Magdalena Wiesner; Marcela Mercado-Reyes; Maria T. Herrera-Sepúlveda; Marta Lopez Blanco; Martha Lucia Ospina Martinez; Sergio Gomez; Sheryll Corchuelo; Ángela Alarcon Cruz
EPI_ISL_1820926	UNIVERSIDAD DE Magdalena	Instituto Nacional de Salud- Dirección de Investigación en Salud Pública	Carlos Franco-Muñoz; Carmen Osorio; Christian Romero; Diana Malo; Diego A. Álvarez-Díaz; Diego Andrés Prada; Gerardo Santamaría; Hector Alejandro Ruiz-Moreno; Jhonnatan Reales-González; Jorge Rivera; Juan Camilo Martinez; Julian Naizaque; Katherine Laiton-Donato; Lisseth Pardo; Magdalena Wiesner; Marcela Mercado-Reyes; Maria T. Herrera-Sepúlveda; Marta Lopez Blanco; Martha Lucia Ospina Martinez; Paola Rojas; Patricia del Portillo; Sergio Gomez; Sheryll Corchuelo; Ángela Alarcon Cruz
EPI_ISL_3355478, EPI_ISL_3355479, EPI_ISL_3355482, EPI_ISL_3355487, EPI_ISL_3355488, EPI_ISL_3355489, EPI_ISL_3355490, EPI_ISL_3355491, EPI_ISL_3355518, EPI_ISL_3369924, EPI_ISL_3369933, EPI_ISL_3369936, EPI_ISL_3369941			
see above	UNIVERSIDAD DEL QUINDIO	Corporacion CorpoGen-Universidad de los Andes-Universidad Central	Christian Romero; Jorge Duitama; Juan Manuel Anzola; Laura González; Maryam Chaib De Mares; María Mercedes Zambrano; Nelly Díaz; Patricia Del Portillo; Silvia Restrepo
EPI_ISL_4300694	UNIVERSIDAD DEL QUINDIO	Instituto Nacional de Salud- Dirección de Investigación en Salud Pública	Carlos Franco-Muñoz; Carmen Osorio; Diana Malo; Diego A. Álvarez-Díaz; Diego Andrés Prada; Gerardo Santamaría; Hector Alejandro Ruiz-Moreno; Jhonnatan Reales-González; Jorge Rivera; Juan Camilo Martinez; Julian Naizaque; Katherine Laiton-Donato; Lisseth Pardo; Magdalena Wiesner; Marcela Mercado-Reyes; Maria T. Herrera-Sepúlveda; Marta Lopez Blanco; Martha Lucia Ospina Martinez; Paola Rojas; Sergio Gomez; Sheryll Corchuelo; Ángela Alarcon Cruz
EPI_ISL_1632529, EPI_ISL_1632530, EPI_ISL_1632531, EPI_ISL_1820931, EPI_ISL_1820936, EPI_ISL_1820942, EPI_ISL_1820948, EPI_ISL_1820952, EPI_ISL_1820953, EPI_ISL_1820956, EPI_ISL_3385777, EPI_ISL_3385778, EPI_ISL_3385779, EPI_ISL_3385783, EPI_ISL_3385784			
see above	UNIVERSIDAD DEL SINU	Instituto Nacional de Salud- Dirección de Investigación en Salud Pública	Carlos Franco-Muñoz; Carmen Osorio; Christian Romero; Diana Malo; Diego A. Álvarez-Díaz; Diego Andrés Prada; Gerardo Santamaría; Hector Alejandro Ruiz-Moreno; Jhonnatan Reales-González; Jorge Rivera; Juan Camilo Martinez; Julian Naizaque; Katherine Laiton-Donato; Lisseth Pardo; Magdalena Wiesner; Marcela Mercado-Reyes; Maria T. Herrera-Sepúlveda; Marta Lopez Blanco; Martha Lucia Ospina Martinez; Paola Rojas; Patricia del Portillo; Sergio Gomez; Sheryll Corchuelo; Ángela Alarcon Cruz
EPI_ISL_1324134, EPI_ISL_1324135, EPI_ISL_1324136, EPI_ISL_1324138, EPI_ISL_1324141, EPI_ISL_1324143, EPI_ISL_1324144, EPI_ISL_1324148			
see above	UW Virology Lab	UW Virology Lab	Alexander Greninger; Hong Xie; Keith R Jerome; Lasata Shrestha; Margaret Mills; Meei-Li Huang; Michelle Lin; Noah Baker; Pavitra Roychoudhury; Saraswathi Sathees; Sean Ellis; Shah Mohamed Bakhash
EPI_ISL_3277384, EPI_ISL_3277385, EPI_ISL_3277386, EPI_ISL_3277387, EPI_ISL_3277388, EPI_ISL_3277389, EPI_ISL_3277390, EPI_ISL_3277391, EPI_ISL_3277392, EPI_ISL_3277393, EPI_ISL_3277394			
see above	Unidad de Investigaciones Moleculares (UNIMOL) -	Centro de Genética y Biología Molecular - Universidad del Magdalena	Andrea M. Ramírez Hernandez; Angel Oviedo Marquez; Daniel Bautista; Lyda R. Castro; Maria Teresa Mojica-Ortiz

	Universidad de Cartagena		
EPI_ISL_2828019, EPI_ISL_2828020, EPI_ISL_2828021, EPI_ISL_2828022, EPI_ISL_2828023, EPI_ISL_2828024, EPI_ISL_2828025, EPI_ISL_2828026, EPI_ISL_2828027, EPI_ISL_2828028, EPI_ISL_2828029, EPI_ISL_2828030, EPI_ISL_2828031, EPI_ISL_2828032, EPI_ISL_2828033, EPI_ISL_2828034, EPI_ISL_2828035, EPI_ISL_2828036, EPI_ISL_2828037, EPI_ISL_2828038			
see above	Unidad de Investigación Molecular (UNIMOL)	Instituto Nacional de Salud- Dirección de Investigación en Salud Pública	Carlos Franco-Muñoz; Carmen Osorio; Diana Malo; Diego A. Álvarez-Díaz; Diego Andrés Prada; Gerardo Santamaría; Hector Alejandro Ruiz-Moreno; Jhonnatan Reales-González; Jorge Rivera; Juan Camilo Martinez; Julian Naizaque; Katherine Laiton-Donato; Lisseth Pardo; Magdalena Wiesner; Marcela Mercado-Reyes; Maria T. Herrera-Sepúlveda; Marta Lopez Blanco; Martha Lucia Ospina Martinez; Paola Rojas; Sergio Gomez; Sheryll Corchuelo; Ángela Alarcon Cruz
EPI_ISL_4081112, EPI_ISL_4081113	Universidad Cartagena Laboratorio UNIMOL	Instituto Nacional de Salud- Dirección de Investigación en Salud Pública	Carlos Franco-Muñoz; Carmen Osorio; Diana Malo; Diego A. Álvarez-Díaz; Diego Andrés Prada; Gerardo Santamaría; Hector Alejandro Ruiz-Moreno; Jhonnatan Reales-González; Jorge Rivera; Juan Camilo Martinez; Julian Naizaque; Katherine Laiton-Donato; Lisseth Pardo; Magdalena Wiesner; Marcela Mercado-Reyes; Maria T. Herrera-Sepúlveda; Marta Lopez Blanco; Martha Lucia Ospina Martinez; Paola Rojas; Sergio Gomez; Sheryll Corchuelo; Ángela Alarcon Cruz
EPI_ISL_1628499	Universidad Industrial de Santander (Laboratorio Central de Investigaciones - Clínica Chicamocha).	Universidad Industrial de Santander	Carlos Barrios-Hernández; Carolina S. Torres-Jiménez; Cristian E. Cadena-Caballero; Diego Rueda-Plata; Erika Lizarazo-Gutiérrez; Francisco Martinez-Perez; Lina M. Vera-Cala.; Lizeth J. Forero-Buitrago
EPI_ISL_1576835, EPI_ISL_1577026, EPI_ISL_1577182, EPI_ISL_1577390, EPI_ISL_1582589, EPI_ISL_1582590, EPI_ISL_1628474, EPI_ISL_1629710, EPI_ISL_1629711			
see above	Universidad Industrial de Santander (Laboratorio Central de Investigaciones - Clínica Chicamocha).	Universidad Industrial de Santander.	Carlos Barrios-Hernández; Carolina S. Torres-Jiménez; Cristian E. Cadena-Caballero; Diego Rueda-Plata; Erika Lizarazo-Gutiérrez; Francisco Martinez-Perez; Lina M. Vera-Cala.; Lizeth J. Forero-Buitrago
EPI_ISL_3390788, EPI_ISL_3390789, EPI_ISL_3390790, EPI_ISL_3390791, EPI_ISL_3390792, EPI_ISL_3390793, EPI_ISL_3390794, EPI_ISL_3390795, EPI_ISL_3390796, EPI_ISL_3390797, EPI_ISL_3390798, EPI_ISL_3390799, EPI_ISL_3390800, EPI_ISL_3390801, EPI_ISL_3390802, EPI_ISL_3390803, EPI_ISL_3390804, EPI_ISL_3390805, EPI_ISL_3390806, EPI_ISL_3390807, EPI_ISL_3390808, EPI_ISL_3390809, EPI_ISL_3390810, EPI_ISL_3390811, EPI_ISL_3390812, EPI_ISL_3390813, EPI_ISL_3390814, EPI_ISL_3390815, EPI_ISL_3390816, EPI_ISL_3390817, EPI_ISL_3390818, EPI_ISL_3390819, EPI_ISL_3390820, EPI_ISL_3390821, EPI_ISL_3390822, EPI_ISL_3390823, EPI_ISL_3390824, EPI_ISL_3390825, EPI_ISL_3390826, EPI_ISL_3390827, EPI_ISL_3390828, EPI_ISL_3390829, EPI_ISL_3390830, EPI_ISL_3390831, EPI_ISL_3390832, EPI_ISL_3390833, EPI_ISL_3390834, EPI_ISL_3390835, EPI_ISL_3390836, EPI_ISL_3390837, EPI_ISL_3390838, EPI_ISL_3390839, EPI_ISL_3390840, EPI_ISL_3390841, EPI_ISL_3390842, EPI_ISL_3390843, EPI_ISL_3390844, EPI_ISL_3390845, EPI_ISL_3390846, EPI_ISL_3390847, EPI_ISL_3390848, EPI_ISL_3390849, EPI_ISL_3390850, EPI_ISL_3390851, EPI_ISL_3390852, EPI_ISL_3390853, EPI_ISL_3390854, EPI_ISL_3390855, EPI_ISL_3390856, EPI_ISL_3390857, EPI_ISL_3390858, EPI_ISL_3390859, EPI_ISL_3390860, EPI_ISL_3390861, EPI_ISL_3390862, EPI_ISL_3390863, EPI_ISL_3390864, EPI_ISL_3390865, EPI_ISL_3390866, EPI_ISL_3390867, EPI_ISL_3390868, EPI_ISL_3390869, EPI_ISL_3390870, EPI_ISL_3390871, EPI_ISL_3390872, EPI_ISL_3390873, EPI_ISL_3390874, EPI_ISL_3390875, EPI_ISL_3390876, EPI_ISL_3390877, EPI_ISL_3390878, EPI_ISL_3390879, EPI_ISL_3390880, EPI_ISL_3390881, EPI_ISL_3390882, EPI_ISL_3390883, EPI_ISL_3390884, EPI_ISL_3390885, EPI_ISL_3390886, EPI_ISL_3390887, EPI_ISL_3390888, EPI_ISL_3390889, EPI_ISL_3390890, EPI_ISL_3390891, EPI_ISL_3390892, EPI_ISL_3390893, EPI_ISL_3390894, EPI_ISL_3390895, EPI_ISL_3390896, EPI_ISL_3390897, EPI_ISL_3390898, EPI_ISL_3390899, EPI_ISL_3390900, EPI_ISL_3390901, EPI_ISL_3390902, EPI_ISL_3390903, EPI_ISL_3390904, EPI_ISL_3390905, EPI_ISL_3390906, EPI_ISL_3390907			
see above	Universidad Nacional Bogota	UNAL	Universidad Nacional de Colombia
EPI_ISL_1675332, EPI_ISL_1675333, EPI_ISL_1675334, EPI_ISL_1824711, EPI_ISL_1824712, EPI_ISL_1824713, EPI_ISL_1824714, EPI_ISL_1824715, EPI_ISL_2438094, EPI_ISL_2438098, EPI_ISL_2438101, EPI_ISL_2651232, EPI_ISL_3276644, EPI_ISL_3276645, EPI_ISL_3276646, EPI_ISL_3276647, EPI_ISL_3276648, EPI_ISL_3276649, EPI_ISL_3276650, EPI_ISL_3276651, EPI_ISL_3276652, EPI_ISL_3276653			
see above	Universidad Nacional de Colombia - Laboratorio Genómico One Health	Universidad Nacional de Colombia - Laboratorio Genómico One Health	Andres F. Cardona-Rios; Carlos Franco-Muñoz; Carolina Muñoz-Arango; Celeny Ortiz; Daniel O. Maldonado-Perez; Diego A. Álvarez-Díaz; Hector Alejandro Ruiz-Moreno; Idabely Betancur Ortiz; Jorge E. Osorio; Juan P. Hernandez-Ortiz; Karl A Ciuoderis; Katherine Laiton-Donato; Laura Silvana Perez; Lina M. Hurtado; Marcela Mercado-Reyes; Maria Angélica Maya; Maria Stella López; Rita Almanza Payares; Sandra Ines Cano; Simón Villegas Velásquez
EPI_ISL_3671011, EPI_ISL_3671020, EPI_ISL_3671028, EPI_ISL_3721607, EPI_ISL_3721611	Universidad Nacional sede Medellin Laboratorio One Health	Universidad Nacional de Colombia - Laboratorio Genómico One Health	Andres F. Cardona-Rios; Carlos Franco-Muñoz; Carolina Muñoz-Arango; Celeny Ortiz; Daniel O. Maldonado-Perez; Diego A. Álvarez-Díaz; Hector Alejandro Ruiz-Moreno; Idabely Betancur Ortiz; Jorge E. Osorio; Juan P. Hernandez-Ortiz; Karl A Ciuoderis; Katherine Laiton-Donato; Laura Silvana Perez; Lina M. Hurtado; Marcela Mercado-Reyes; Maria Angélica Maya; Maria Stella López; Rita Almanza Payares; Sandra Ines Cano; Simón Villegas Velásquez
EPI_ISL_4297510, EPI_ISL_4297511, EPI_ISL_4297512, EPI_ISL_4297513, EPI_ISL_4297514, EPI_ISL_4297515, EPI_ISL_4297516, EPI_ISL_4297517			
see above	Universidad Simón Bolivar	Centro de Genética y Biología Molecular - Universidad del Magdalena	Andrea M. Ramírez Hernandez; Angel Oviedo Marquez; Daniel Bautista; Edison Lea-Ch; Lyda R. Castro; Maria Teresa Mojica-Ortiz
EPI_ISL_3671022, EPI_ISL_3671046	Universidad de Antioquia Laboratorio LIME	Universidad Nacional de Colombia - Laboratorio Genómico One Health	Andres F. Cardona-Rios; Carlos Franco-Muñoz; Carolina Muñoz-Arango; Celeny Ortiz; Daniel O. Maldonado-Perez; Diego A. Álvarez-Díaz; Hector Alejandro Ruiz-Moreno; Idabely Betancur Ortiz; Jorge E. Osorio; Juan P. Hernandez-Ortiz; Karl A Ciuoderis; Katherine Laiton-Donato; Laura Silvana Perez; Lina M. Hurtado; Marcela Mercado-Reyes; Maria Angélica Maya; Maria Stella López; Rita Almanza Payares; Sandra Ines Cano; Simón Villegas Velásquez
EPI_ISL_3721586, EPI_ISL_3721592, EPI_ISL_3721599, EPI_ISL_3721601, EPI_ISL_3721605, EPI_ISL_3721622	Universidad de Caldas	Universidad Nacional de Colombia - Laboratorio Genómico One Health	Andres F. Cardona-Rios; Carlos Franco-Muñoz; Carolina Muñoz-Arango; Celeny Ortiz; Daniel O. Maldonado-Perez; Diego A. Álvarez-Díaz; Hector Alejandro Ruiz-Moreno; Idabely Betancur Ortiz; Jorge E. Osorio; Juan P. Hernandez-Ortiz; Karl A Ciuoderis; Katherine Laiton-Donato; Laura Silvana Perez; Lina M. Hurtado; Marcela Mercado-Reyes; Maria Angélica Maya; Maria Stella López; Rita Almanza Payares; Sandra Ines Cano; Simón Villegas Velásquez
EPI_ISL_2339856, EPI_ISL_2339857, EPI_ISL_2339858, EPI_ISL_2339859, EPI_ISL_2339860, EPI_ISL_2339861, EPI_ISL_2339862, EPI_ISL_2339863, EPI_ISL_2339864, EPI_ISL_2339865, EPI_ISL_2339866, EPI_ISL_2339867, EPI_ISL_2339868, EPI_ISL_2339869, EPI_ISL_2339870, EPI_ISL_2339871, EPI_ISL_2339872, EPI_ISL_2339873, EPI_ISL_2339874, EPI_ISL_2603124, EPI_ISL_2603125, EPI_ISL_2603126, EPI_ISL_2603127, EPI_ISL_2603128, EPI_ISL_2603129, EPI_ISL_2603130			
see above	Universidad de Cordoba, Montería, Colombia	Centro de Investigaciones en Microbiología y Biotecnología-UR (CIMBIUR), Facultad de Ciencias Naturales, Universidad del Rosario, Bogotá, Colombia	Ader Alemán; Alejandra Garcia; Alfonso Calderón; Andrés Diaz; Bertha Gastelbondo; Camilo Guzmán; Caty Martinez; Evelin Garay; Germán Arrieta; Hector Contreras; Hector Serrano; Jorge Miranda; José Berrocal; Juan David Ramírez; Ketty Galeano; Luz H. Patiño; Maria Auxiliadora Badillo; Marina Muñoz; Nathalia Ballesteros; Ricardo Rivero; Salim Mattar; Sergio Castañeda; Veronica Contreras; Yesica Botero; Yesica Lopez
EPI_ISL_1220045	Universidad de Magdalena	Instituto Nacional de Salud- Dirección de Investigación en Salud Pública	Carlos Franco-Muñoz; Diego A. Álvarez-Díaz; Diego Andrés Prada; Gerardo Santamaría; Hector Alejandro Ruiz-Moreno; Jhonnatan Reales-González; Julian Naizaque; Katherine Laiton-Donato; Magdalena Wiesner; Marcela Mercado-Reyes.; Maria T. Herrera-Sepúlveda; Martha Lucia Ospina Martínez; Sheryll Corchuelo
EPI_ISL_3671010, EPI_ISL_3671024, EPI_ISL_3671025, EPI_ISL_3671044, EPI_ISL_3671045, EPI_ISL_3671047, EPI_ISL_3671059			
see above	Universidad de Sucre	Universidad Nacional de Colombia - Laboratorio Genómico One Health	Andres F. Cardona-Rios; Carlos Franco-Muñoz; Carolina Muñoz-Arango; Celeny Ortiz; Daniel O. Maldonado-Perez; Diego A. Álvarez-Díaz; Hector Alejandro Ruiz-Moreno; Idabely Betancur Ortiz; Jorge E. Osorio; Juan P. Hernandez-Ortiz; Karl A Ciuoderis; Katherine Laiton-Donato; Laura Silvana Perez; Lina M. Hurtado; Marcela Mercado-Reyes; Maria Angélica Maya; Maria Stella López; Rita Almanza Payares; Sandra Ines Cano; Simón Villegas Velásquez
EPI_ISL_1582991, EPI_ISL_1582992, EPI_ISL_1582993, EPI_ISL_1582994	Universidad del Atlántico Laboratorio de Investigación en Biología Molecular	Instituto Nacional de Salud- Dirección de Investigación en Salud Pública	Carlos Franco-Muñoz; Carmen Osorio; Diana Malo; Diego A. Álvarez-Díaz; Diego Andrés Prada; Gerardo Santamaría; Hector Alejandro Ruiz-Moreno; Jhonnatan Reales-González; Juan Camilo Martinez; Julian Naizaque; Katherine Laiton-Donato; Lisseth Pardo; Magdalena Wiesner; Marcela Mercado-Reyes; Maria T. Herrera-Sepúlveda; Marta Lopez Blanco; Martha Lucia Ospina Martinez; Paola Rojas; Sergio Gomez; Sheryll Corchuelo; Ángela Alarcon Cruz
EPI_ISL_3998105, EPI_ISL_3998111, EPI_ISL_3998122, EPI_ISL_3998124, EPI_ISL_3998127, EPI_ISL_3998140, EPI_ISL_3998144, EPI_ISL_3998146, EPI_ISL_3998154, EPI_ISL_3998155			
see above	Universidad del Atlántico Laboratorio de Investigación en Biología Molecular	Laboratorio de Biologia Molecular, Universidad Cooperativa de Colombia, Santa Marta	Andrew S. Muñoz-Gamba; Daniel B. Ramírez-Osorio; José A. Usme-Ciro; Paula A. Quintero-Cortés; Roberto Garcia
EPI_ISL_1424054, EPI_ISL_1424055, EPI_ISL_1424056	Universidad del Magdalena	Instituto Nacional de Salud- Dirección de Investigación en Salud Pública	Carlos Franco-Muñoz; Diego A. Álvarez-Díaz; Diego Andrés Prada; Gerardo Santamaría; Hector Alejandro Ruiz-Moreno; Jhonnatan Reales-González; Julian Naizaque; Katherine Laiton-Donato; Magdalena Wiesner; Marcela Mercado-Reyes.; Maria T. Herrera-Sepúlveda; Martha Lucia Ospina Martínez; Sheryll Corchuelo
EPI_ISL_3320720, EPI_ISL_3320725, EPI_ISL_3320743, EPI_ISL_3320748, EPI_ISL_3320752	Universidad del Quindio	Instituto Nacional de Salud	Carlos Franco-Muñoz; Carmen Osorio; Diana Malo; Diego A. Álvarez-Díaz; Diego Andrés Prada; Gerardo Santamaría; Hector Alejandro Ruiz-Moreno; Jhonnatan Reales-González; Jorge Rivera; Juan Camilo Martinez; Julian Naizaque; Katherine Laiton-Donato; Lisseth Pardo; Magdalena Wiesner; Marcela Mercado-Reyes; Maria T. Herrera-Sepúlveda; Marta Lopez Blanco; Martha Lucia Ospina Martinez; Paola Rojas; Sergio Gomez; Sheryll Corchuelo; Ángela Alarcon Cruz
EPI_ISL_4205067, EPI_ISL_4205068, EPI_ISL_4205069, EPI_ISL_4205070, EPI_ISL_4205071, EPI_ISL_4205072, EPI_ISL_4205073, EPI_ISL_4205074, EPI_ISL_4205075, EPI_ISL_4205076, EPI_ISL_4205077, EPI_ISL_4205078, EPI_ISL_4205079, EPI_ISL_4205080, EPI_ISL_4205081, EPI_ISL_4205082, EPI_ISL_4205083, EPI_ISL_4254657, EPI_ISL_4254658			
see above	Universidad del Rosario	Gencell Pharma	Diego Andrés Otero-Rodríguez; Fabio Andrés Zapata-Gómez; Henry Mauricio Chaparro-Solano; Juan Mauricio Pardo-Oviedo; Jubby Marcela Gálvez-Bermúdez; Ludwig Luis Antonio Albornoz; Nicolás Molano-González; Ángela María Pinzón-Rondón; Ángela María Ruiz-Sternberg
EPI_ISL_445219	Universidad del Valle, Laboratorio de Microbiologia, VIREM	Universidad del Valle, Universidad Nacional de Colombia-Sede Palmira, International Center for Tropical Agriculture	Beatriz Parra; Diana López-Alvarez; Wilmer J. Cuellar
EPI_ISL_3898951, EPI_ISL_3898953, EPI_ISL_3898955, EPI_ISL_3914566, EPI_ISL_3914567, EPI_ISL_3914568, EPI_ISL_3914569, EPI_ISL_3914570, EPI_ISL_3914571, EPI_ISL_3914572, EPI_ISL_3914573, EPI_ISL_3914574, EPI_ISL_3914575, EPI_ISL_3914576, EPI_ISL_3914577, EPI_ISL_3914578, EPI_ISL_3914579, EPI_ISL_3914580, EPI_ISL_3914581, EPI_ISL_3914582, EPI_ISL_3914583, EPI_ISL_3914584, EPI_ISL_3914585, EPI_ISL_3914586, EPI_ISL_3914587, EPI_ISL_3914588, EPI_ISL_3914589, EPI_ISL_3914590, EPI_ISL_3914591, EPI_ISL_3914592, EPI_ISL_3914593, EPI_ISL_3914594, EPI_ISL_3914595, EPI_ISL_3914596, EPI_ISL_3914597, EPI_ISL_3914598, EPI_ISL_3914599, EPI_ISL_3914600, EPI_ISL_3914601, EPI_ISL_3914602, EPI_ISL_3914603, EPI_ISL_3914604, EPI_ISL_3914605, EPI_ISL_3914606, EPI_ISL_3941427, EPI_ISL_3941428, EPI_ISL_3941429, EPI_ISL_4419134, EPI_ISL_4419135, EPI_ISL_4419136, EPI_ISL_4419137, EPI_ISL_4419138, EPI_ISL_4419139, EPI_ISL_4419140, EPI_ISL_4419141, EPI_ISL_4419142, EPI_ISL_4419143, EPI_ISL_4419144, EPI_ISL_4419145, EPI_ISL_4419146, EPI_ISL_4419147, EPI_ISL_4419148, EPI_ISL_4419149, EPI_ISL_4419150, EPI_ISL_4419151, EPI_ISL_4419152, EPI_ISL_4419153, EPI_ISL_4419154, EPI_ISL_4419155, EPI_ISL_4419156, EPI_ISL_4419157, EPI_ISL_4419158, EPI_ISL_4419159, EPI_ISL_4419160, EPI_ISL_4419161, EPI_ISL_4419162, EPI_ISL_4419163, EPI_ISL_4419164, EPI_ISL_4419165, EPI_ISL_4419166, EPI_ISL_4419167, EPI_ISL_4419168, EPI_ISL_4419169, EPI_ISL_4419170, EPI_ISL_4419171, EPI_ISL_4419172, EPI_ISL_4419173, EPI_ISL_4419174, EPI_ISL_4419175, EPI_ISL_4419184			
see above	Universidad del Valle, LDAB-Laboratorio de Diagnostico de Agentes Biologicos	Universidad del Valle	Programa Nacional de Caracterización Genómica de SARS-CoV-2
EPI_ISL_3216903, EPI_ISL_3216904, EPI_ISL_3216905, EPI_ISL_3216906, EPI_ISL_3216907, EPI_ISL_3216908, EPI_ISL_3216909, EPI_ISL_3216910, EPI_ISL_3216911, EPI_ISL_3477560, EPI_ISL_3477561, EPI_ISL_3477562, EPI_ISL_3477563, EPI_ISL_3477564, EPI_ISL_3477565, EPI_ISL_3477566, EPI_ISL_3477567, EPI_ISL_3477568, EPI_ISL_3477569, EPI_ISL_3477570, EPI_ISL_3477571, EPI_ISL_3477572, EPI_ISL_3477573			
see above	Universidad del Valle, LDAB-Laboratorio de Diagnostico de Agentes Biologicos	Universidad del Valle, TAO-Lab, VIREM, NEAS Network	Andres Castillo; Beatriz Parra & COVID-19 Team Univalle; Diana López-Alvarez; Nelson Rivera Franco
EPI_ISL_3398821, EPI_ISL_3398822, EPI_ISL_3398823	VIROLOGIA INS DRSP	Instituto Nacional de Salud	Carlos Franco-Muñoz; Carmen Osorio; Diana Malo; Diego A. Álvarez-Díaz; Diego Andrés Prada; Gerardo Santamaría; Hector Alejandro Ruiz-Moreno; Jhonnatan Reales-González; Jorge Rivera; Juan Camilo Martinez; Julian Naizaque; Katherine Laiton-Donato; Lisseth Pardo; Magdalena Wiesner; Marcela Mercado-Reyes; Maria T.Herrera-Sepúlveda; Marta Lopez Blanco; Martha Lucia Ospina Martinez; Paola Rojas; Sergio Gomez; Sheryll Corchuelo; Ángela Alarcon Cruz
EPI_ISL_1652046, EPI_ISL_1652047, EPI_ISL_1652048, EPI_ISL_1820937, EPI_ISL_1820944, EPI_ISL_1820945, EPI_ISL_1820947, EPI_ISL_1820949, EPI_ISL_1820955, EPI_ISL_2674281			
see above	VIROLOGIA INS DRSP	Instituto Nacional de Salud- Dirección de Investigación en Salud Pública	Carlos Franco-Muñoz; Carmen Osorio; Christian Romero; Diana Malo; Diego A. Álvarez-Díaz; Diego Andrés Prada; Gerardo Santamaría; Hector Alejandro Ruiz-Moreno; Jhonnatan Reales-González; Jorge Rivera; Juan Camilo Martinez; Julian Naizaque; Katherine Laiton-Donato; Lisseth Pardo; Magdalena Wiesner; Marcela Mercado-Reyes; Maria T. Herrera-Sepúlveda; Marta Lopez Blanco; Martha Lucia Ospina Martinez; Paola Rojas; Patricia del Portillo; Sergio Gomez; Sheryll Corchuelo; Ángela Alarcon Cruz
EPI_ISL_445085	Virology Unit, Agrobiodiversity and Biotechnology Project, CIAT - International Center for Tropical Agriculture	Virology Unit, Agrobiodiversity and Biotechnology Project, CIAT - International Center for Tropical Agriculture	B. and Cuellar; Lopez, D.; Parra; W.J.
EPI_ISL_845620	compensar calle 63	Instituto Nacional de Salud - Dirección de Investigación en Salud Pública	Carlos Franco-Muñoz; Diego A. Álvarez-Díaz; Diego Andrés Prada; Gerardo Santamaría; Jonathan Reales; Julian Naizaque; Katherine Laiton-Donato; Magdalena Wiesner; Marcela Mercado-Reyes; Maria T. Herrera-Sepúlveda; Martha Lucia Ospina Martinez; Mauricio Pacheco-Montealegre; Paola Muñoz-Laiton; Sheryll Corchuelo
EPI_ISL_4219529	lab Christus Sinergia Salud -Clin Farallones	Molecular Genetics and Antimicrobial Resistance - UGRA, Universidad El Bosque	Catalina Espitia; Jinnethe Reyes; Lorena Diaz; Marcela Mercado; Mauricio Pacheco; Rafael Rios; Valentina Martinez
EPI_ISL_2657875	laboratorio de salud publica de cundinamarca	Instituto Nacional de Salud- Dirección de Investigación en Salud Pública	Carlos Franco-Muñoz; Carmen Osorio; Diana Malo; Diego A. Álvarez-Díaz; Diego Andrés Prada; Gerardo Santamaría; Hector Alejandro Ruiz-Moreno; Jhonnatan Reales-González; Jorge Rivera; Juan Camilo Martinez; Julian Naizaque; Katherine Laiton-Donato; Lisseth Pardo; Magdalena Wiesner; Marcela Mercado-Reyes; Maria T. Herrera-Sepúlveda; Marta Lopez Blanco; Martha Lucia Ospina Martinez; Paola Rojas; Sergio Gomez; Sheryll Corchuelo; Ángela Alarcon Cruz
EPI_ISL_1235687	laboratorio de salud pública (Bogotá)	Gencore- Universidad de los Andes	Alejandro Gómez; David González; Johana Hernandez Gabriela Delgado; Luisa Sacristán; Marcela Guevara-Suarez; Silvia Restrepo
EPI_ISL_2828054	laboratorio departamental de salud publica - santander	Instituto Nacional de Salud- Dirección de Investigación en Salud Pública	Carlos Franco-Muñoz; Carmen Osorio; Diana Malo; Diego A. Álvarez-Díaz; Diego Andrés Prada; Gerardo Santamaría; Hector Alejandro Ruiz-Moreno; Jhonnatan Reales-González; Jorge Rivera; Juan Camilo Martinez; Julian Naizaque; Katherine Laiton-Donato; Lisseth Pardo; Magdalena Wiesner; Marcela Mercado-Reyes; Maria T. Herrera-Sepúlveda; Marta Lopez Blanco; Martha Lucia Ospina Martinez; Paola Rojas; Sergio Gomez; Sheryll Corchuelo; Ángela Alarcon Cruz
