# Supplementary material for: Genomic epidemiology of SARS-CoV-2 variants during the first two years of the pandemic in Colombia
Source: Commun Med (Lond). 2023 Jul 13;3:97. doi: 10.1038/s43856-023-00328-3 (PMC10344885; doi:10.1038/s43856-023-00328-3)
Supplement: Supplementary file 4 — Supplementary Data 2 [file 43856_2023_328_MOESM4_ESM.pdf]

Supplementary Data 2. Access number of the novel SARS-CoV-2 sequences from Colombia included in this genome sequences used in this study, 124 genomes sequenced in this study and 104 downloaded from GISAI in the same time frame as the social protest are highlighted in gra

All submitters of data may be contacted directly via www.gisaid.org

Virus name	Accession ID
hCoV-19/Colombia/VAC-A149/2021	EPI_ISL_7476675
hCoV-19/Colombia/VAC-A148/2021	EPI_ISL_7476671
hCoV-19/Colombia/VAC-A157/2021|EPI_ISL_2603182|2021-04-05	EPI_ISL_2603182
hCoV-19/Colombia/VAC-A147/2021|EPI_ISL_2584557|2021-04-05	EPI_ISL_2584557
hCoV-19/Colombia/VAC-A150/2021|EPI_ISL_2584559|2021-04-05	EPI_ISL_2584559
hCoV-19/Colombia/VAC-A156/2021|EPI_ISL_2584561|2021-04-05	EPI_ISL_2584561
hCoV-19/Colombia/VAC-A151/2021|EPI_ISL_2603177|2021-04-05	EPI_ISL_2603177
hCoV-19/Colombia/VAC-A153/2021|EPI_ISL_2603179|2021-04-05	EPI_ISL_2603179
hCoV-19/Colombia/VAC-A154/2021|EPI_ISL_2603180|2021-04-05	EPI_ISL_2603180
hCoV-19/Colombia/VAC-A155/2021|EPI_ISL_2603181|2021-04-05	EPI_ISL_2603181
hCoV-19/Colombia/VAC-INS-VG-2047/2021|EPI_ISL_2009058|2021-04-08	EPI_ISL_2009058
hCoV-19/Colombia/VAC-A043/2021|EPI_ISL_2339835|2021-04-09	EPI_ISL_2339835
hCoV-19/Colombia/VAC-A039/2021|EPI_ISL_2339831|2021-04-09	EPI_ISL_2339831
hCoV-19/Colombia/VAC-A040/2021|EPI_ISL_2339832|2021-04-09	EPI_ISL_2339832
hCoV-19/Colombia/VAC-INS-VG-2285/2021|EPI_ISL_2158345|2021-04-09	EPI_ISL_2158345
hCoV-19/Colombia/VAC-A037/2021|EPI_ISL_2339830|2021-04-09	EPI_ISL_2339830
hCoV-19/Colombia/VAC-A042/2021|EPI_ISL_2339834|2021-04-09	EPI_ISL_2339834
hCoV-19/Colombia/VAC-K9/2021	EPI_ISL_7476481
hCoV-19/Colombia/VAC-K8/2021	EPI_ISL_7476530
hCoV-19/Colombia/VAC-A014/2021|EPI_ISL_2339808|2021-04-12	EPI_ISL_2339808
hCoV-19/Colombia/VAC-A011/2021|EPI_ISL_2339805|2021-04-12	EPI_ISL_2339805
hCoV-19/Colombia/VAC-A015/2021|EPI_ISL_2339809|2021-04-12	EPI_ISL_2339809
hCoV-19/Colombia/VAC-A016/2021|EPI_ISL_2339810|2021-04-12	EPI_ISL_2339810
hCoV-19/Colombia/VAC-INS-VG-2073/2021|EPI_ISL_2009059|2021-04-12	EPI_ISL_2009059
hCoV-19/Colombia/VAC-INS-VG-2074/2021|EPI_ISL_2155039|2021-04-12	EPI_ISL_2155039
hCoV-19/Colombia/VAC-A012/2021|EPI_ISL_2339806|2021-04-12	EPI_ISL_2339806
hCoV-19/Colombia/VAC-A013/2021|EPI_ISL_2339807|2021-04-12	EPI_ISL_2339807
hCoV-19/Colombia/VAC-A031/2021|EPI_ISL_2339824|2021-04-12	EPI_ISL_2339824
hCoV-19/Colombia/VAC-A032/2021|EPI_ISL_2339825|2021-04-12	EPI_ISL_2339825
hCoV-19/Colombia/COV_23933/2021|EPI_ISL_3216903|2021-04-14	EPI_ISL_3216903
hCoV-19/Colombia/VAC-K4/2021	EPI_ISL_7476195
hCoV-19/Colombia/VAC-K1/2021	EPI_ISL_7476429
hCoV-19/Colombia/VAC-K10/2021	EPI_ISL_7476532
hCoV-19/Colombia/VAC-K11/2021	EPI_ISL_7476776
hCoV-19/Colombia/VAC-K3/2021	EPI_ISL_7476266
hCoV-19/Colombia/VAC-K5/2021	EPI_ISL_7476273
hCoV-19/Colombia/VAC-K6/2021	EPI_ISL_7476401
hCoV-19/Colombia/VAC-K7/2021	EPI_ISL_7476602
hCoV-19/Colombia/VAC-K2/2021	EPI_ISL_7476639

hCoV-19/Colombia/VAC-K12/2021	EPI_ISL_7476873
hCoV-19/Colombia/VAC-INS-VG-4112/2021|EPI_ISL_3385807|2021-04-24	EPI_ISL_3385807
hCoV-19/Colombia/VAC-K41/2021	EPI_ISL_7476549
hCoV-19/Colombia/VAC-K18/2021	EPI_ISL_7476877
hCoV-19/Colombia/VAC-K19/2021	EPI_ISL_7476887
hCoV-19/Colombia/VAC-INS-VG-2523/2021|EPI_ISL_2362560|2021-04-26	EPI_ISL_2362560
hCoV-19/Colombia/VAC-INS-VG-2850/2021|EPI_ISL_2674313|2021-04-26	EPI_ISL_2674313
hCoV-19/Colombia/VAC-INS-VG-2851/2021|EPI_ISL_2674314|2021-04-26	EPI_ISL_2674314
hCoV-19/Colombia/VAC-INS-VG-2852/2021|EPI_ISL_2674315|2021-04-26	EPI_ISL_2674315
hCoV-19/Colombia/VAC-INS-VG-2853/2021|EPI_ISL_2674316|2021-04-26	EPI_ISL_2674316
hCoV-19/Colombia/VAC-INS-VG-3526/2021|EPI_ISL_3385815|2021-04-26	EPI_ISL_3385815
hCoV-19/Colombia/VAC-K17/2021	EPI_ISL_7476457
hCoV-19/Colombia/VAC-K16/2021	EPI_ISL_7476542
hCoV-19/Colombia/VAC-COV_24419/2021|EPI_ISL_3477560|2021-04-28	EPI_ISL_3477560
hCoV-19/Colombia/VAC-K13/2021	EPI_ISL_7476805
hCoV-19/Colombia/VAC-K20/2021	EPI_ISL_7476335
hCoV-19/Colombia/VAC-K22/2021	EPI_ISL_7476461
hCoV-19/Colombia/VAC-K21/2021	EPI_ISL_7476741
hCoV-19/Colombia/VAC-K23/2021	EPI_ISL_7476811
hCoV-19/Colombia/VAC-K24/2021	EPI_ISL_7476815
hCoV-19/Colombia/COV_24547/2021|EPI_ISL_3216904|2021-05-06	EPI_ISL_3216904
hCoV-19/Colombia/VAC-COV_24539/2021|EPI_ISL_3477561|2021-05-06	EPI_ISL_3477561
hCoV-19/Colombia/VAC-INS-VG-4111/2021|EPI_ISL_3385834|2021-05-07	EPI_ISL_3385834
hCoV-19/Colombia/VAC-K29/2021	EPI_ISL_7476525
hCoV-19/Colombia/VAC-K32/2021	EPI_ISL_7476656
hCoV-19/Colombia/VAC-K30/2021	EPI_ISL_7476697
hCoV-19/Colombia/VAC-K31/2021	EPI_ISL_7476704
hCoV-19/Colombia/VAC-K33/2021	EPI_ISL_7476711
hCoV-19/Colombia/VAC-K34/2021	EPI_ISL_7476717
hCoV-19/Colombia/VAC-K25/2021	EPI_ISL_7476823
hCoV-19/Colombia/VAC-K26/2021	EPI_ISL_7476833
hCoV-19/Colombia/VAC-K28/2021	EPI_ISL_7476843
hCoV-19/Colombia/VAC-K27/2021	EPI_ISL_7476836
hCoV-19/Colombia/VAC-COV_24589/2021|EPI_ISL_3477562|2021-05-10	EPI_ISL_3477562
hCoV-19/Colombia/COV_24623/2021|EPI_ISL_3216905|2021-05-11	EPI_ISL_3216905
hCoV-19/Colombia/COV_24635/2021|EPI_ISL_3216906|2021-05-11	EPI_ISL_3216906
hCoV-19/Colombia/COV_24656/2021|EPI_ISL_3216907|2021-05-13	EPI_ISL_3216907
hCoV-19/Colombia/VAC-COV_24670/2021|EPI_ISL_3477563|2021-05-13	EPI_ISL_3477563
hCoV-19/Colombia/VAC-K43/2021	EPI_ISL_7476423
hCoV-19/Colombia/VAC-K47/2021	EPI_ISL_7476562
hCoV-19/Colombia/VAC-K44/2021	EPI_ISL_7476582
hCoV-19/Colombia/VAC-K35/2021	EPI_ISL_7476846
hCoV-19/Colombia/VAC-K36/2021	EPI_ISL_7476853
hCoV-19/Colombia/VAC-K37/2021	EPI_ISL_7476860
hCoV-19/Colombia/COV_24770/2021|EPI_ISL_3216908|2021-05-19	EPI_ISL_3216908
hCoV-19/Colombia/VAC-COV_24759/2021|EPI_ISL_3477565|2021-05-19	EPI_ISL_3477565
hCoV-19/Colombia/VAC-COV_24796/2021|EPI_ISL_3477566|2021-05-20	EPI_ISL_3477566

hCoV-19/Colombia/VAC-K45/2021	EPI_ISL_7476326
hCoV-19/Colombia/VAC-K46/2021	EPI_ISL_7476345
hCoV-19/Colombia/VAC-K42/2021	EPI_ISL_7476627
hCoV-19/Colombia/VAC-K51/2021	EPI_ISL_7476480
hCoV-19/Colombia/VAC-COV_24846/2021|EPI_ISL_3477567|2021-05-24	EPI_ISL_3477567
hCoV-19/Colombia/VAC-COV_24867/2021|EPI_ISL_3477568|2021-05-24	EPI_ISL_3477568
hCoV-19/Colombia/VAC-K52/2021	EPI_ISL_7476288
hCoV-19/Colombia/COV_24923/2021|EPI_ISL_3216909|2021-05-26	EPI_ISL_3216909
hCoV-19/Colombia/VAC-INS-VG-4113/2021|EPI_ISL_3385787|2021-05-26	EPI_ISL_3385787
hCoV-19/Colombia/VAC-K58/2021	EPI_ISL_7476244
hCoV-19/Colombia/VAC-K53/2021	EPI_ISL_7476285
hCoV-19/Colombia/VAC-K49/2021	EPI_ISL_7476302
hCoV-19/Colombia/VAC-K57/2021	EPI_ISL_7476365
hCoV-19/Colombia/VAC-K55/2021	EPI_ISL_7476408
hCoV-19/Colombia/VAC-K54/2021	EPI_ISL_7476482
hCoV-19/Colombia/VAC-K50/2021	EPI_ISL_7476680
hCoV-19/Colombia/VAC-K56/2021	EPI_ISL_7476796
hCoV-19/Colombia/VAC-K65/2021	EPI_ISL_7476591
hCoV-19/Colombia/VAC-K60/2021	EPI_ISL_7476282
hCoV-19/Colombia/VAC-K68/2021	EPI_ISL_7476635
hCoV-19/Colombia/VAC-COV_25057/2021|EPI_ISL_3477569|2021-06-02	EPI_ISL_3477569
hCoV-19/Colombia/VAC-COV_25067/2021|EPI_ISL_3477570|2021-06-02	EPI_ISL_3477570
hCoV-19/Colombia/VAC-K62/2021	EPI_ISL_7476181
hCoV-19/Colombia/VAC-K61/2021	EPI_ISL_7476340
hCoV-19/Colombia/VAC-K63/2021	EPI_ISL_7476357
hCoV-19/Colombia/VAC-K59/2021	EPI_ISL_7476556
hCoV-19/Colombia/VAC-K66/2021	EPI_ISL_7476597
hCoV-19/Colombia/VAC-K67/2021	EPI_ISL_7476646
hCoV-19/Colombia/VAC-K64/2021	EPI_ISL_7476651
hCoV-19/Colombia/VAC-COV-VG-3805/2021|EPI_ISL_2834726|2021-06-04	EPI_ISL_2834726
hCoV-19/Colombia/VAC-K73/2021	EPI_ISL_7476218
hCoV-19/Colombia/VAC-K70/2021	EPI_ISL_7476351
hCoV-19/Colombia/VAC-K72/2021	EPI_ISL_7476417
hCoV-19/Colombia/VAC-K74/2021	EPI_ISL_7476228
hCoV-19/Colombia/VAC-K76/2021	EPI_ISL_7476237
hCoV-19/Colombia/VAC-K75/2021	EPI_ISL_7476536
hCoV-19/Colombia/VAC-INS-VG-1916/2021|EPI_ISL_3385820|2021-06-10	EPI_ISL_3385820
hCoV-19/Colombia/VAC-K69/2021	EPI_ISL_7476585
hCoV-19/Colombia/VAC-K71/2021	EPI_ISL_7476615
hCoV-19/Colombia/VAC-K77/2021	EPI_ISL_7476622
hCoV-19/Colombia/VAC-INS-VG-4114/2021|EPI_ISL_3385808|2021-06-12	EPI_ISL_3385808
hCoV-19/Colombia/VAC-INS-VG-4115/2021|EPI_ISL_3385849|2021-06-12	EPI_ISL_3385849
hCoV-19/Colombia/VAC-K88/2021	EPI_ISL_7476308
hCoV-19/Colombia/VAC-K85/2021	EPI_ISL_7476319
hCoV-19/Colombia/VAC-K80/2021	EPI_ISL_7476368
hCoV-19/Colombia/VAC-K87/2021	EPI_ISL_7476771
hCoV-19/Colombia/VAC-K81/2021	EPI_ISL_7476380

hCoV-19/Colombia/VAC-K86/2021	EPI_ISL_7476395
hCoV-19/Colombia/VAC-K84/2021	EPI_ISL_7476483
hCoV-19/Colombia/VAC-K82/2021	EPI_ISL_7476200
hCoV-19/Colombia/VAC-K79/2021	EPI_ISL_7476391
hCoV-19/Colombia/VAC-K83/2021	EPI_ISL_7476501
hCoV-19/Colombia/VAC-K95/2021	EPI_ISL_7476174
hCoV-19/Colombia/VAC-K97/2021	EPI_ISL_7476300
hCoV-19/Colombia/VAC-K96/2021	EPI_ISL_7476449
hCoV-19/Colombia/VAC-K89/2021	EPI_ISL_7476866
hCoV-19/Colombia/VAC-K94/2021	EPI_ISL_7476900
hCoV-19/Colombia/VAC-K93/2021	EPI_ISL_7476330
hCoV-19/Colombia/VAC-K90/2021	EPI_ISL_7476370
hCoV-19/Colombia/VAC-K98/2021	EPI_ISL_7476376
hCoV-19/Colombia/VAC-K92/2021	EPI_ISL_7476661
hCoV-19/Colombia/VAC-K91/2021	EPI_ISL_7476794
hCoV-19/Colombia/VAC-K106/2021	EPI_ISL_7476491
hCoV-19/Colombia/VAC-K100/2021	EPI_ISL_7476690
hCoV-19/Colombia/VAC-COV_25774/2021|EPI_ISL_3477571|2021-06-28	EPI_ISL_3477571
hCoV-19/Colombia/VAC-COV_25776/2021|EPI_ISL_3477572|2021-06-28	EPI_ISL_3477572
hCoV-19/Colombia/VAC-COV_25810/2021|EPI_ISL_3477573|2021-06-28	EPI_ISL_3477573
hCoV-19/Colombia/COV_25796/2021|EPI_ISL_3216910|2021-06-29	EPI_ISL_3216910
hCoV-19/Colombia/COV_25875/2021|EPI_ISL_3216911|2021-06-29	EPI_ISL_3216911
hCoV-19/Colombia/VAC-COV_25884/2021|EPI_ISL_3477564|2021-06-29	EPI_ISL_3477564
hCoV-19/Colombia/VAC-K104/2021	EPI_ISL_7476259
hCoV-19/Colombia/VAC-K105/2021	EPI_ISL_7476475
hCoV-19/Colombia/VAC-K103/2021	EPI_ISL_7476510
hCoV-19/Colombia/VAC-K107/2021	EPI_ISL_7476523
hCoV-19/Colombia/VAC-K102/2021	EPI_ISL_7476733
hCoV-19/Colombia/VAC-K101/2021	EPI_ISL_7476801
hCoV-19/Colombia/VAC-K99/2021	EPI_ISL_7476894
hCoV-19/Colombia/VAC-UV-20025918/2021|EPI_ISL_4419151|2021-06-30	EPI_ISL_4419151
hCoV-19/Colombia/VAC-UV-20025921/2021|EPI_ISL_4419152|2021-06-30	EPI_ISL_4419152
hCoV-19/Colombia/VAC-UV-20025938/2021|EPI_ISL_4419153|2021-06-30	EPI_ISL_4419153
hCoV-19/Colombia/VAC-UV-20025944/2021|EPI_ISL_4419154|2021-06-30	EPI_ISL_4419154
hCoV-19/Colombia/VAC-UV-20025962/2021|EPI_ISL_4419155|2021-07-01	EPI_ISL_4419155
hCoV-19/Colombia/VAC-UV-20025981/2021|EPI_ISL_4419156|2021-07-01	EPI_ISL_4419156
hCoV-19/Colombia/VAC-K108/2021	EPI_ISL_7476467
hCoV-19/Colombia/CO-UV-26067/2021|EPI_ISL_3914569|2021-07-02	EPI_ISL_3914569
hCoV-19/Colombia/VAC-SCOV-019/2021|EPI_ISL_3065505|2021-07-03	EPI_ISL_3065505
hCoV-19/Colombia/VAC-K110/2021	EPI_ISL_7476188
hCoV-19/Colombia/VAC-K116/2021	EPI_ISL_7476442
hCoV-19/Colombia/VAC-K109/2021	EPI_ISL_7476485
hCoV-19/Colombia/VAC-K115/2021	EPI_ISL_7476570
hCoV-19/Colombia/VAC-K113/2021	EPI_ISL_7476610
hCoV-19/Colombia/VAC-K111/2021	EPI_ISL_7476748
hCoV-19/Colombia/VAC-K112/2021	EPI_ISL_7476750
hCoV-19/Colombia/VAC-K114/2021	EPI_ISL_7476758

hCoV-19/Colombia/VAC-UV-20026043/2021|EPI_ISL_4419157|2021-07-06	EPI_ISL_4419157
hCoV-19/Colombia/VAC-UV-20026047/2021|EPI_ISL_4419158|2021-07-06	EPI_ISL_4419158
hCoV-19/Colombia/CO-UV-26250/2021|EPI_ISL_3914570|2021-07-08	EPI_ISL_3914570
hCoV-19/Colombia/CO-UV-26254/2021|EPI_ISL_3914571|2021-07-08	EPI_ISL_3914571
hCoV-19/Colombia/CO-UV-20026301/2021|EPI_ISL_3914566|2021-07-11	EPI_ISL_3914566
hCoV-19/Colombia/VAC-K126/2021	EPI_ISL_7476314
hCoV-19/Colombia/VAC-K120/2021	EPI_ISL_7476484
hCoV-19/Colombia/VAC-K124/2021	EPI_ISL_7476518
hCoV-19/Colombia/VAC-K117/2021	EPI_ISL_7476687
hCoV-19/Colombia/VAC-K119/2021	EPI_ISL_7476727
hCoV-19/Colombia/VAC-K118/2021	EPI_ISL_7476765
hCoV-19/Colombia/CO-UV-26362/2021|EPI_ISL_3914573|2021-07-13	EPI_ISL_3914573
hCoV-19/Colombia/VAC-UV-20026390/2021|EPI_ISL_4419159|2021-07-13	EPI_ISL_4419159
hCoV-19/Colombia/VAC-UV-20026397/2021|EPI_ISL_4419160|2021-07-13	EPI_ISL_4419160
hCoV-19/Colombia/VAC-UV-20026398/2021|EPI_ISL_4419161|2021-07-13	EPI_ISL_4419161
hCoV-19/Colombia/VAC-UV-26361/2021|EPI_ISL_4419184|2021-07-13	EPI_ISL_4419184
hCoV-19/Colombia/CO-UV-20026419/2021|EPI_ISL_3914567|2021-07-14	EPI_ISL_3914567
hCoV-19/Colombia/CO-UV-26439/2021|EPI_ISL_3914574|2021-07-14	EPI_ISL_3914574
hCoV-19/Colombia/VAC-UV-20026403/2021|EPI_ISL_4419162|2021-07-14	EPI_ISL_4419162
hCoV-19/Colombia/VAC-UV-20026423/2021|EPI_ISL_4419163|2021-07-14	EPI_ISL_4419163
hCoV-19/Colombia/CO-UV-20026422/2021|EPI_ISL_3898951|2021-07-15	EPI_ISL_3898951
hCoV-19/Colombia/VAC-UNIANDES-G027946/2021|EPI_ISL_4566188|2021-07-16	EPI_ISL_4566188
hCoV-19/Colombia/VAC-UNIANDES-G027947/2021|EPI_ISL_4566190|2021-07-16	EPI_ISL_4566190
hCoV-19/Colombia/VAC-UV-20026515/2021|EPI_ISL_4419165|2021-07-17	EPI_ISL_4419165
hCoV-19/Colombia/CO-UV-26548/2021|EPI_ISL_3914578|2021-07-19	EPI_ISL_3914578
hCoV-19/Colombia/CO-UV-26561/2021|EPI_ISL_3914576|2021-07-19	EPI_ISL_3914576
hCoV-19/Colombia/VAC-UV-20026511/2021|EPI_ISL_4419164|2021-07-19	EPI_ISL_4419164
hCoV-19/Colombia/VAC-UV-20026546/2021|EPI_ISL_4419166|2021-07-19	EPI_ISL_4419166
hCoV-19/Colombia/VAC-UV-20026550/2021|EPI_ISL_4419167|2021-07-19	EPI_ISL_4419167
hCoV-19/Colombia/VAC-UV-20026567/2021|EPI_ISL_4419168|2021-07-20	EPI_ISL_4419168
hCoV-19/Colombia/VAC-UV-20026612/2021|EPI_ISL_4419170|2021-07-21	EPI_ISL_4419170
hCoV-19/Colombia/VAC-UV-20026620/2021|EPI_ISL_4419171|2021-07-21	EPI_ISL_4419171
hCoV-19/Colombia/VAC-UV-20026634/2021|EPI_ISL_4419172|2021-07-21	EPI_ISL_4419172
hCoV-19/Colombia/VAC-K128/2021	EPI_ISL_7476210
hCoV-19/Colombia/VAC-K133/2021	EPI_ISL_7476437
hCoV-19/Colombia/VAC-K127/2021	EPI_ISL_7476784
hCoV-19/Colombia/VAC-K129/2021	EPI_ISL_7476790
hCoV-19/Colombia/VAC-UV-20026671/2021|EPI_ISL_4419173|2021-07-23	EPI_ISL_4419173
hCoV-19/Colombia/CO-UV-26723/2021|EPI_ISL_3914579|2021-07-26	EPI_ISL_3914579
hCoV-19/Colombia/CO-UV-26747/2021|EPI_ISL_3914581|2021-07-26	EPI_ISL_3914581
hCoV-19/Colombia/CO-UV-26748/2021|EPI_ISL_3941427|2021-07-26	EPI_ISL_3941427
hCoV-19/Colombia/VAC-UV-20026750/2021|EPI_ISL_4419174|2021-07-26	EPI_ISL_4419174
hCoV-19/Colombia/VAC-K38/2021	EPI_ISL_7476253
hCoV-19/Colombia/VAC-K135/2021	EPI_ISL_7476293
hCoV-19/Colombia/VAC-K134/2021	EPI_ISL_7476576
hCoV-19/Colombia/CO-UV-26847/2021|EPI_ISL_3914582|2021-07-29	EPI_ISL_3914582
hCoV-19/Colombia/VAC-UV-20026844/2021|EPI_ISL_4419175|2021-07-29	EPI_ISL_4419175

hCoV-19/Colombia/VAC-HUV-202108010198.30/2021|EPI_ISL_4419183|2021-07-31	EPI_ISL_4419183

study. Currently status until June-2022: 228
D. The genomes with a sample collection date
ay.

Date	Status
2021-04-05	Sequenced for the study
2021-04-05	Sequenced for the study
2021-04-05	Downloaded from GISAID
2021-04-05	Downloaded from GISAID
2021-04-05	Downloaded from GISAID
2021-04-05	Downloaded from GISAID
2021-04-05	Downloaded from GISAID
2021-04-05	Downloaded from GISAID
2021-04-05	Downloaded from GISAID
2021-04-05	Downloaded from GISAID
2021-04-08	Downloaded from GISAID
2021-04-09	Downloaded from GISAID
2021-04-09	Downloaded from GISAID
2021-04-09	Downloaded from GISAID
2021-04-09	Downloaded from GISAID
2021-04-09	Downloaded from GISAID
2021-04-09	Downloaded from GISAID
2021-04-12	Sequenced for the study
2021-04-12	Sequenced for the study
2021-04-12	Downloaded from GISAID
2021-04-12	Downloaded from GISAID
2021-04-12	Downloaded from GISAID
2021-04-12	Downloaded from GISAID
2021-04-12	Downloaded from GISAID
2021-04-12	Downloaded from GISAID
2021-04-12	Downloaded from GISAID
2021-04-12	Downloaded from GISAID
2021-04-12	Downloaded from GISAID
2021-04-12	Downloaded from GISAID
2021-04-14	Downloaded from GISAID
2021-04-19	Sequenced for the study
2021-04-19	Sequenced for the study
2021-04-19	Sequenced for the study
2021-04-19	Sequenced for the study
2021-04-23	Sequenced for the study
2021-04-23	Sequenced for the study
2021-04-23	Sequenced for the study
2021-04-23	Sequenced for the study
2021-04-23	Sequenced for the study

2021-04-23	Sequenced for the study
2021-04-24	Downloaded from GISAID
2021-04-26	Sequenced for the study
2021-04-26	Sequenced for the study
2021-04-26	Sequenced for the study
2021-04-26	Downloaded from GISAID
2021-04-26	Downloaded from GISAID
2021-04-26	Downloaded from GISAID
2021-04-26	Downloaded from GISAID
2021-04-26	Downloaded from GISAID
2021-04-26	Downloaded from GISAID
2021-04-28	Sequenced for the study
2021-04-28	Sequenced for the study
2021-04-28	Downloaded from GISAID
2021-05-01	Sequenced for the study
2021-05-04	Sequenced for the study
2021-05-04	Sequenced for the study
2021-05-04	Sequenced for the study
2021-05-04	Sequenced for the study
2021-05-04	Sequenced for the study
2021-05-06	Downloaded from GISAID
2021-05-06	Downloaded from GISAID
2021-05-07	Downloaded from GISAID
2021-05-10	Sequenced for the study
2021-05-10	Sequenced for the study
2021-05-10	Sequenced for the study
2021-05-10	Sequenced for the study
2021-05-10	Sequenced for the study
2021-05-10	Sequenced for the study
2021-05-10	Sequenced for the study
2021-05-10	Sequenced for the study
2021-05-10	Sequenced for the study
2021-05-10	Sequenced for the study
2021-05-10	Downloaded from GISAID
2021-05-11	Downloaded from GISAID
2021-05-11	Downloaded from GISAID
2021-05-13	Downloaded from GISAID
2021-05-13	Downloaded from GISAID
2021-05-18	Sequenced for the study
2021-05-18	Sequenced for the study
2021-05-18	Sequenced for the study
2021-05-18	Sequenced for the study
2021-05-18	Sequenced for the study
2021-05-18	Sequenced for the study
2021-05-19	Downloaded from GISAID
2021-05-19	Downloaded from GISAID
2021-05-20	Downloaded from GISAID

2021-05-21	Sequenced for the study
2021-05-21	Sequenced for the study
2021-05-21	Sequenced for the study
2021-05-24	Sequenced for the study
2021-05-24	Downloaded from GISAID
2021-05-24	Downloaded from GISAID
2021-05-26	Sequenced for the study
2021-05-26	Downloaded from GISAID
2021-05-26	Downloaded from GISAID
2021-05-29	Sequenced for the study
2021-05-29	Sequenced for the study
2021-05-29	Sequenced for the study
2021-05-29	Sequenced for the study
2021-05-29	Sequenced for the study
2021-05-29	Sequenced for the study
2021-05-29	Sequenced for the study
2021-05-29	Sequenced for the study
2021-05-31	Sequenced for the study
2021-06-02	Sequenced for the study
2021-06-02	Sequenced for the study
2021-06-02	Downloaded from GISAID
2021-06-02	Downloaded from GISAID
2021-06-04	Sequenced for the study
2021-06-04	Sequenced for the study
2021-06-04	Sequenced for the study
2021-06-04	Sequenced for the study
2021-06-04	Sequenced for the study
2021-06-04	Sequenced for the study
2021-06-04	Sequenced for the study
2021-06-04	Downloaded from GISAID
2021-06-08	Sequenced for the study
2021-06-08	Sequenced for the study
2021-06-08	Sequenced for the study
2021-06-09	Sequenced for the study
2021-06-09	Sequenced for the study
2021-06-09	Sequenced for the study
2021-06-10	Downloaded from GISAID
2021-06-11	Sequenced for the study
2021-06-11	Sequenced for the study
2021-06-11	Sequenced for the study
2021-06-12	Downloaded from GISAID
2021-06-12	Downloaded from GISAID
2021-06-15	Sequenced for the study
2021-06-15	Sequenced for the study
2021-06-15	Sequenced for the study
2021-06-15	Sequenced for the study
2021-06-16	Sequenced for the study

2021-06-16	Sequenced for the study
2021-06-16	Sequenced for the study
2021-06-17	Sequenced for the study
2021-06-17	Sequenced for the study
2021-06-17	Sequenced for the study
2021-06-21	Sequenced for the study
2021-06-21	Sequenced for the study
2021-06-21	Sequenced for the study
2021-06-21	Sequenced for the study
2021-06-21	Sequenced for the study
2021-06-23	Sequenced for the study
2021-06-23	Sequenced for the study
2021-06-23	Sequenced for the study
2021-06-23	Sequenced for the study
2021-06-23	Sequenced for the study
2021-06-28	Sequenced for the study
2021-06-28	Sequenced for the study
2021-06-28	Downloaded from GISAID
2021-06-28	Downloaded from GISAID
2021-06-28	Downloaded from GISAID
2021-06-29	Downloaded from GISAID
2021-06-29	Downloaded from GISAID
2021-06-29	Downloaded from GISAID
2021-06-30	Sequenced for the study
2021-06-30	Sequenced for the study
2021-06-30	Sequenced for the study
2021-06-30	Sequenced for the study
2021-06-30	Sequenced for the study
2021-06-30	Sequenced for the study
2021-06-30	Sequenced for the study
2021-06-30	Downloaded from GISAID
2021-06-30	Downloaded from GISAID
2021-06-30	Downloaded from GISAID
2021-06-30	Downloaded from GISAID
2021-07-01	Downloaded from GISAID
2021-07-01	Downloaded from GISAID
2021-07-02	Sequenced for the study
2021-07-02	Downloaded from GISAID
2021-07-03	Downloaded from GISAID
2021-07-06	Sequenced for the study
2021-07-06	Sequenced for the study
2021-07-06	Sequenced for the study
2021-07-06	Sequenced for the study
2021-07-06	Sequenced for the study
2021-07-06	Sequenced for the study
2021-07-06	Sequenced for the study
2021-07-06	Sequenced for the study

2021-07-06	Downloaded from GISAID
2021-07-06	Downloaded from GISAID
2021-07-08	Downloaded from GISAID
2021-07-08	Downloaded from GISAID
2021-07-11	Downloaded from GISAID
2021-07-12	Sequenced for the study
2021-07-12	Sequenced for the study
2021-07-12	Sequenced for the study
2021-07-12	Sequenced for the study
2021-07-12	Sequenced for the study
2021-07-12	Sequenced for the study
2021-07-13	Downloaded from GISAID
2021-07-13	Downloaded from GISAID
2021-07-13	Downloaded from GISAID
2021-07-13	Downloaded from GISAID
2021-07-13	Downloaded from GISAID
2021-07-14	Downloaded from GISAID
2021-07-14	Downloaded from GISAID
2021-07-14	Downloaded from GISAID
2021-07-14	Downloaded from GISAID
2021-07-15	Downloaded from GISAID
2021-07-16	Downloaded from GISAID
2021-07-16	Downloaded from GISAID
2021-07-17	Downloaded from GISAID
2021-07-19	Downloaded from GISAID
2021-07-19	Downloaded from GISAID
2021-07-19	Downloaded from GISAID
2021-07-19	Downloaded from GISAID
2021-07-19	Downloaded from GISAID
2021-07-20	Downloaded from GISAID
2021-07-21	Downloaded from GISAID
2021-07-21	Downloaded from GISAID
2021-07-21	Downloaded from GISAID
2021-07-22	Sequenced for the study
2021-07-22	Sequenced for the study
2021-07-22	Sequenced for the study
2021-07-22	Sequenced for the study
2021-07-23	Downloaded from GISAID
2021-07-26	Downloaded from GISAID
2021-07-26	Downloaded from GISAID
2021-07-26	Downloaded from GISAID
2021-07-26	Downloaded from GISAID
2021-07-28	Sequenced for the study
2021-07-28	Sequenced for the study
2021-07-28	Sequenced for the study
2021-07-29	Downloaded from GISAID
2021-07-29	Downloaded from GISAID

2021-07-31	Downloaded from GISAID
