# Supplementary material for: Genomic epidemiology of SARS-CoV-2 variants during the first two years of the pandemic in Colombia
Source: Commun Med (Lond). 2023 Jul 13;3:97. doi: 10.1038/s43856-023-00328-3 (PMC10344885; doi:10.1038/s43856-023-00328-3)
Supplement: Supplementary file 5 — Reporting Summary [file 43856_2023_328_MOESM5_ESM.pdf]

nature research

Corresponding author(s): COMMSMED-22-0216-T

Last updated by author(s): 2023/06/26

Reporting Summary

Nature Research wishes to improve the reproducibility of the work that we publish. This form provides structure for consistency and transparency in reporting. For further information on Nature Research policies, see our Editorial Policies and the Editorial Policy Checklist.

Statistics

For all statistical analyses, confirm that the following items are present in the figure legend, table legend, main text, or Methods section.

n/a	Confirmed	
☐	☒	The exact sample size (*n*) for each experimental group/condition, given as a discrete number and unit of measurement
☐	☒	A statement on whether measurements were taken from distinct samples or whether the same sample was measured repeatedly
☐	☒	The statistical test(s) used AND whether they are one- or two-sided<br>*Only common tests should be described solely by name; describe more complex techniques in the Methods section.*
☐	☒	A description of all covariates tested
☐	☒	A description of any assumptions or corrections, such as tests of normality and adjustment for multiple comparisons
☐	☒	A full description of the statistical parameters including central tendency (e.g. means) or other basic estimates (e.g. regression coefficient) AND variation (e.g. standard deviation) or associated estimates of uncertainty (e.g. confidence intervals)
☐	☒	For null hypothesis testing, the test statistic (e.g. *F*, *t*, *r*) with confidence intervals, effect sizes, degrees of freedom and *P* value noted<br>*Give P values as exact values whenever suitable.*
☐	☒	For Bayesian analysis, information on the choice of priors and Markov chain Monte Carlo settings
☒	☐	For hierarchical and complex designs, identification of the appropriate level for tests and full reporting of outcomes
☐	☒	Estimates of effect sizes (e.g. Cohen's *d*, Pearson's *r*), indicating how they were calculated

Our web collection on statistics for biologists contains articles on many of the points above.

Software and code

Policy information about availability of computer code

Data collection	We retrieved all SARS-CoV-2 genome sequences from Colombia shared via GISAID (N=14,049, last accessed on 2022-02-02) and combined them with the novel genome sequences.
Data analysis	We used Open-source software in this study. We excluded sequences with bad quality based on six different control metrics implemented in Nextclade. Epidemiological parameters and statistical test were estimated using different packages (nnet package v.7.3-17, emtrends v.1.7.3, ape package v.5.6-2, bdskytools package, ggtree v3.4.0, and stats v4.3.0) in R. v.3.5.0, likewise, plotting was perform with this platform. We aligned the sequence data of each major variant using MAFFT v7. We performed all bayesian phylodynamic and phylogeography analysis using BEAST v2.6.7. We diagnosed the MCMC samples using Tracer v1.7.2. we used Figtree v1.4.4 to visualize trees and outputs.

For manuscripts utilizing custom algorithms or software that are central to the research but not yet described in published literature, software must be made available to editors and reviewers. We strongly encourage code deposition in a community repository (e.g. GitHub). See the Nature Research guidelines for submitting code & software for further information.

Data

Policy information about availability of data

All manuscripts must include a data availability statement. This statement should provide the following information, where applicable:

- Accession codes, unique identifiers, or web links for publicly available datasets
- A list of figures that have associated raw data
- A description of any restrictions on data availability

Accession codes for all sequencing data utilized in this study, as well as any other raw datasets, are available in the supplementary material accompanying this paper. Additionally, all source data for the figures presented in the main manuscript, including the numerical results underlying the graphs and charts, are provided as supplementary files in a machine-readable format. These source data files can also be accessed online at (https://github.com/cinthylorein/Colombia-COVID-19-

phylodynamics.git/). Researchers and readers interested in accessing the data are encouraged to refer to the supplementary material for detailed instructions on data retrieval and utilization. For any further inquiries or assistance, the corresponding author can be contacted via email. Please note that access to certain datasets or raw data may require approval from the appropriate ethics committee and adherence to relevant data sharing agreements.

Field-specific reporting

Please select the one below that is the best fit for your research. If you are not sure, read the appropriate sections before making your selection.

☐ Life sciences ☐ Behavioural & social sciences ☒ Ecological, evolutionary & environmental sciences

For a reference copy of the document with all sections, see nature.com/documents/nr-reporting-summary-flat.pdf

Ecological, evolutionary & environmental sciences study design

All studies must disclose on these points even when the disclosure is negative.

Study description	To provide a better understanding of the epidemiology of SARS-Cov-2 variants in Colombia during the first two years of the pandemic, we used 14,049 complete SARS-CoV-2 genomes from the 32 states of Colombia, and performed Bayesian phylodynamic analyses to estimate the time of variants introduction, their respective effective reproductive number, and effective population size, and the impact of disease control measures.
Research sample	We collected Nasopharyngeal swabs from 10,674 residents from: Bogotá (the Capital District), Cali (the Capital of Valle del Cauca state) and Córdoba state (The Capital city and small towns) in Colombia. We sequenced 610 samples: 86 samples from Córdoba, 122 from Cali, and 402 from Bogotá. We retrieved all SARS-CoV-2 genome sequences from Colombia shared via GISAID (N=14,049, last accessed on 2022-02-02) and combined them with the novel genome sequences.
Sampling strategy	For the novel sequences, we sequenced positive samples with the following data available: travel history (the latest country of travel), patient status (Asymptomatic, mild, severe, critic, and fatal), sample collection date, and vaccination status. Our selection criteria resulted in 610 samples. Consequently, we retrieved all SARS-CoV-2 genome sequences from Colombia shared via GISAID (N=14,049, last accessed on 2022-02-02) and combined them with the novel genome sequences. We down-sampled the alignments by variant and homogeneously through the time (to have at least one sequence per day); any variant with greater than or equal to 100 samples was considered a major variant. This down-sampling resulted in 1662 sequences distributed in 10 different alignments (Table 4).
Data collection	We retrieved all SARS-CoV-2 genome sequences from Colombia shared via GISAID (N=14,049, last accessed on 2022-02-02) and combined them with the novel genome sequences.
Timing and spatial scale	The novel Colombian genome sequences were from three localities in Colombia and from 2021-04-05 to 2021-07-31. all SARS-CoV-2 genome sequences from Colombia collected via GISAID were from March 2020 to February 2022.
Data exclusions	We excluded sequences with bad quality based on six different control metrics implemented in Nextclade: no more than 10\% ambiguous characters, no more than ten mixed sites, no more than 10\% of missing data (Ns > 3000), no more than two mutation clusters, number of insertions or deletions that are not a multiple of three and number of stop codons that occur in unexpected places (2 stop codons are bad), and any outlier sequence as reported by Nextstrain. We also removed sequences with incongruent lineage classification between Pangolin and Nextclade.
Reproducibility	For the phylodynamics analysis, we performed different models to test congruence in estimating the parameters. Also, we used three independent Markov Chain Monte Carlo with 400 million iterations using the CoupledMCMC package (MC3) v1.0.2.
Randomization	We down-sampled the alignments by variant and homogeneously through the time (to have at least one sequence per day).
Blinding	Blinding was not relevant for this study. The goal was to collect positive SARS-CoV-2 samples from three different Colombia locations.

Did the study involve field work? ☐ Yes ☒ No

Reporting for specific materials, systems and methods

We require information from authors about some types of materials, experimental systems and methods used in many studies. Here, indicate whether each material, system or method listed is relevant to your study. If you are not sure if a list item applies to your research, read the appropriate section before selecting a response.

Materials & experimental systems

n/a	Involved in the study	
☒	☐	Antibodies
☒	☐	Eukaryotic cell lines
☒	☐	Palaeontology and archaeology
☒	☐	Animals and other organisms
☐	☒	Human research participants
☒	☐	Clinical data
☒	☐	Dual use research of concern

Methods

n/a	Involved in the study	
☒	☐	ChIP-seq
☒	☐	Flow cytometry
☒	☐	MRI-based neuroimaging

Human research participants

Policy information about studies involving human research participants

Population characteristics	We collected samples from 610 participants from Cali (the Capital city of Valle del Cauca state), Bogotá (the Capital city of Cundinamarca state), and municipalities from Córdoba state in Colombia. All the participants had confirmed RT-qPCR diagnostic of SARS-CoV-2 infection with Cycle threshold values (Ct) ≤30, with an age range from 0 to 95 years and a median age of 39 years (CI95: 38-40 years). Females were 50.16%, and males were 49.84% of the total participants. We sequenced positive samples with the following data available:Travel history(the latest country of travel)Patient status (Asymptomatic, mild, severe, critic, and fatal)Sample collection dateVaccination status
Recruitment	Instituto de Investigaciones Biologicas del Trópico and Centro de Investigaciones en Microbiología y Biotecnología are part of the authorized laboratories for the diagnostic and genomic surveillance of SARS-CoV-2 led by Colombia's Health Ministry. Healthcare institutions remitted samples, informed consent, and epidemiological registers of patients with COVID-19 symptoms.
Ethics oversight	Sample collection in Córdoba was approved by the Ethics committee of Universidad de Córdoba/IIBT (Acta N° 0410-2020) in compliance with CDC's guidelines for safe work practices in human diagnostic. Sample collection in Bogotá and Cali was approved by Universidad del Rosario's Research Ethics committee (DVO005 1550-CV1400) in compliance with Helsinki's declaration. Informed consent was obtained from all patients.

Note that full information on the approval of the study protocol must also be provided in the manuscript.
